# Dissecting the Conformational Stability of a Glycan Hairpin

**DOI:** 10.1021/jacs.4c00423

**Published:** 2024-02-20

**Authors:** Nishu Yadav, Surusch Djalali, Ana Poveda, Manuel G. Ricardo, Peter H. Seeberger, Jesús Jiménez-Barbero, Martina Delbianco

**Affiliations:** † Department of Biomolecular Systems, Max Planck Institute of Colloids and Interfaces, Am Mühlenberg 1, Potsdam 14476, Germany; ‡ Department of Chemistry and Biochemistry, Freie Universität Berlin, Arnimallee 22, Berlin 14195, Germany; § CIC bioGUNE, Basque Research and Technology Alliance, Derio 48160, Spain; ∥ Ikerbasque, Basque Foundation for Science, Bilbao 48009, Spain; ⊥ Department of Inorganic & Organic Chemistry, Faculty of Science and Technology, University of the Basque Country, EHU-UPV, Leioa 48940, Spain; # Centro de Investigación Biomedica en Red de Enfermedades Respiratorias, Madrid 28029, Spain

## Abstract

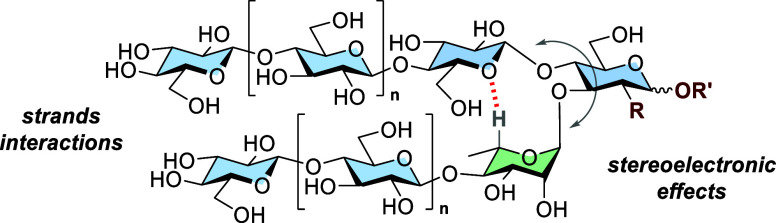

Systematic structural studies of model oligopeptides revealed important aspects of protein folding and offered design principles to access non-natural materials. In the same way, the rules that regulate glycan folding could be established by studying synthetic oligosaccharide models. However, their analysis is often limited due to the synthetic and analytical complexity. By utilizing a glycan capable of spontaneously folding into a hairpin conformation as a model system, we investigated the factors that contribute to its conformational stability in aqueous solution. The modular design of the hairpin model featured a trisaccharide turn unit and two β-1,4-oligoglucoside stacking strands that allowed for systematic chemical modifications of the glycan sequence, including the introduction of NMR labels and staples. Nuclear magnetic resonance assisted by molecular dynamics simulations revealed that stereoelectronic effects and multiple glycan–glycan interactions are the major determinants of folding stabilization. Chemical modifications in the glycan primary sequence (e.g., strand elongation) can be employed to fine-tune the rigidity of structural motifs distant from the modification sites. These results could inspire the design of other glycan architectures, with implications in glycobiology and material sciences.

## Introduction

**Figure 1:**
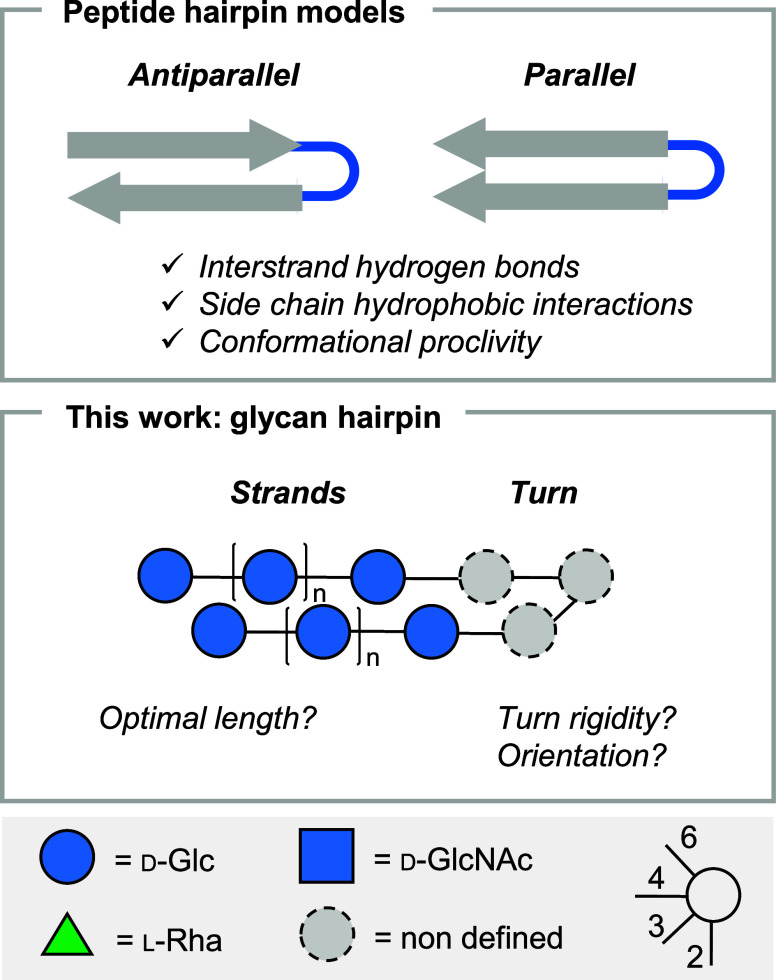
Comparison between peptide and glycan hairpins: Autonomously folding antiparallel or parallel peptide hairpin model systems have been widely explored in terms of conformational stability, whereas less is known about the rules that regulate glycan folding. Herein, we explore the main determinants of the conformational stability of a model glycan hairpin. The following abbreviations are used for monosaccharides: Glc = glucose (blue circle), GlcNAc = *N*-acetyl glucosamine (blue square), and Rha = rhamnose (green triangle). The monosaccharide residues are represented following the Symbol Nomenclature for Glycans (SNFG).^25^

Synthetic oligomers capable of folding into defined conformations (i.e., foldamers) are valuable models to dissect the intricate rules that regulate biopolymer folding.^1,2^ These discrete molecules offer well-defined chemical structures and modularity of synthesis and allow for systematic chemical modifications to establish correlations between the primary sequence and secondary structures.^3^ For example, synthetic peptide hairpins improved our understanding of the factors influencing folding,^2,4^ stability,^5^ and aggregation^6^ of beta-sheet containing proteins (e.g., amyloids^7^). Parallel^8^ or antiparallel^4^ hairpins (Figure 1) as well as more complex three-strand beta-sheets systems^9^ were designed, and their conformational stability was dissected through systematic chemical modifications. These systems revealed the origin of the beta-sheet stability as a complex combination of interstrand hydrogen bonds,^10^ van der Waals and hydrophobic interactions,^11^ and conformational proclivity of the backbone (i.e., stereoelectronic effects and entropic factors).^12^ These works also offered design principles to construct synthetic analogues capable of spanning non-natural geometries.^13,14^

In contrast to peptides, the rules that regulate glycan folding are much more obscure, because these compounds exist in a dynamic ensemble of conformers separated by low energy barriers.^15^ In addition, the lack of synthetic standards and the complexity of glycan analysis have hampered systematic structural studies.^16,17^ Nevertheless, insights into the fundamentals of glycan folding are extremely valuable to understand glycan properties^18^ and design synthetic analogues capable of performing a particular function.^19^

Recently, we have designed and synthesized a glycan sequence, capable of spontaneously folding into a hairpin conformation (Figure 1).^20^ The design featured a rigid trisaccharide turn motif substituted with two β-1,4-oligoglucoside stacking strands. NMR analysis unequivocally confirmed its folded conformation in an aqueous solution. The modular design of the glycan hairpin offers an ideal model system to systematically investigate the determinants of its conformational stability and identify design principles to access new folded architectures.

Herein, we dissect the underlying forces that stabilize glycan folding by using a glycan hairpin model. We prepared a collection of synthetic hairpin analogues to systematically analyze (i) the chemical structure of the turn unit, (ii) the interstrand interactions between the β-1,4-glucan strands, and (iii) the strands’ length influence on conformational stability (Figure 1). All hairpin analogues were synthesized with automated glycan assembly (AGA)^21,22^; the collection featured ^13^C-labeled analogues^16,23^ as well as a stapled glycan hairpin to serve as a “fully folded” reference structure.^24^ Structural analysis was performed with nuclear magnetic resonance (NMR) spectroscopy, assisted by molecular dynamics (MD) simulations. We reveal that glycan–glycan interactions can be exploited to rigidify glycan sequences and that simple chemical modifications can dramatically influence the stability of glycan motifs remote from the modification site. These rules can inspire the creation of novel glycan foldamers as well as glycomimetics with increased conformational stability.^19^

## Results and Discussion

A hairpin can be conceptualized as a turn unit substituted with two stacking strands. Herein, we discuss how systematic modifications of these structural motives affect hairpin conformational stability to pinpoint the major determinants of folding stabilization.

All hairpins were synthesized by AGA on solid support (see Supporting Information Section 3) in an overnight run using previously reported conditions.^20,21^ Post-AGA manipulations included solid-phase methanolysis, photocleavage from the solid support, and hydrogenolysis (see Supporting Information Section 3). A single purification step afforded the target hairpin analogues in overall yields of 4–55%. Atomistic MD simulations were carried out to probe the tendency of the designed glycans to adopt a hairpin conformation. All the modeled structures were simulated for 500 ns unless otherwise specified, employing a modified version of the GLYCAM06^26^ carbohydrate force field. The systems were solvated with the TIP5P^27^ water model to avoid excessive interactions between the monomers.^28^ A systematic terminology to standardize the name and representation of the glycan hairpins discussed in this paper can be found in the SI (Figures S2 and S3). Labeling of protons in a monosaccharide is done as follows: e.g., the proton attached to C-1 of Rha is named “Rha-1”.

### The Effect of the Turn

**Figure 2:**
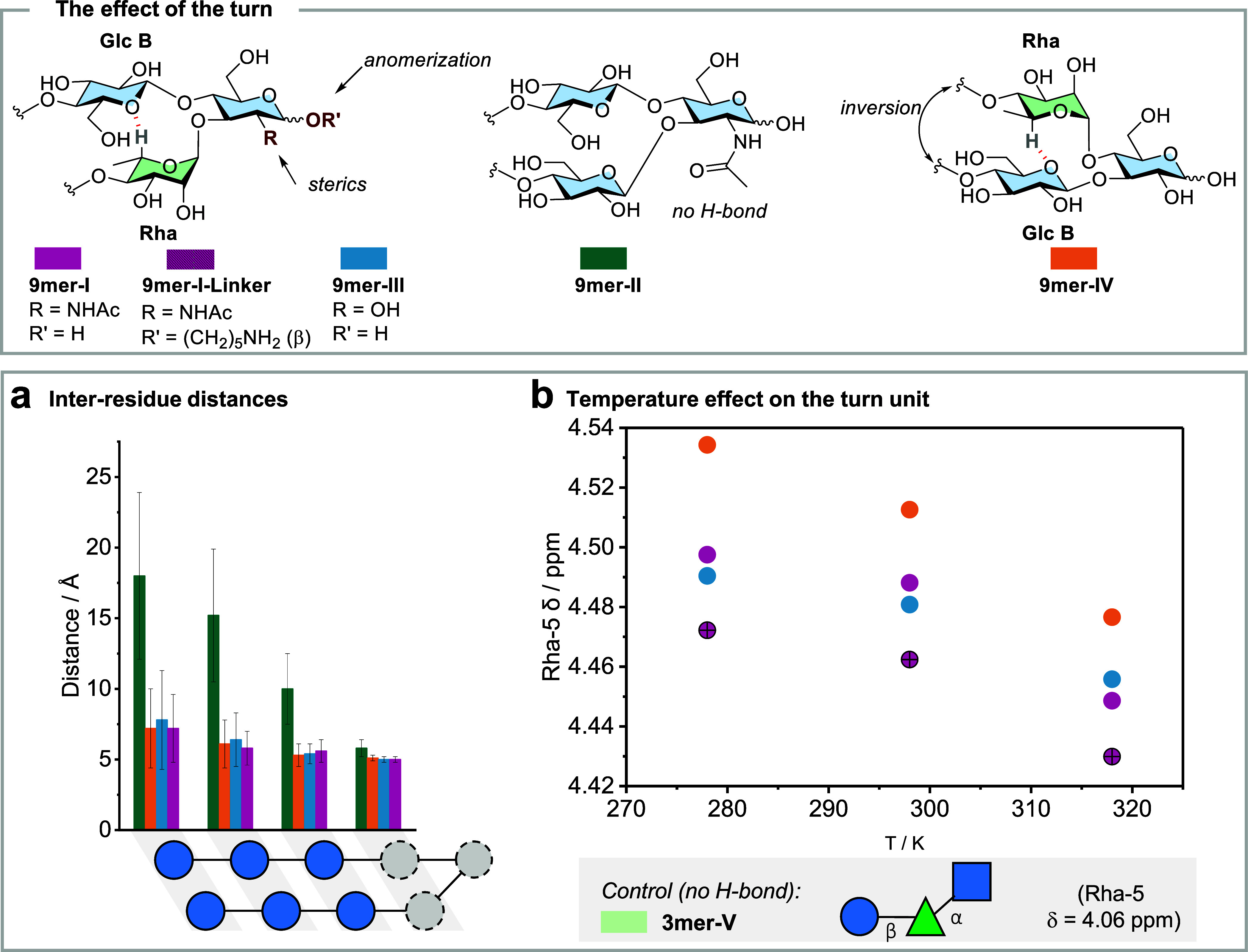
The effect of the turn: Representation of the structures of the 9mer hairpins with the different turn units analyzed in this work (for synthetic details, see the SI). (a) Average inter-residue distances calculated from the MD simulation trajectories (25,001 frames, 500 ns), showing an overall closed conformation for all 9mer apart from **9mer-II**, a control structure lacking the Rha unit. Error bars represent the standard deviation. (b) Temperature-dependent ^1^H NMR analysis of **9mer-I**, **9mer-I-Linker**, **9mer-III**, and **9mer-IV**. The chemical shifts of Rha-5 were extracted from selective 1D TOCSY (278, 298, 318 K, 700 MHz, d9 200 ms, D_2_O) showing that the nonconventional H-bond is preserved over a wide range of temperatures.

The turn unit of **9mer-I** is inspired by the Le^x^ trisaccharide^29^ and contains a reducing d-GlcNAc branching unit substituted with a β-d-Glc unit at C-4 and an α-l-Rha unit at C-3 (Figure 2). A nonconventional CH···O hydrogen bond between the H-5 of l-Rha (Rha-5) and the O-5 pyranose oxygen of Glc-B stabilizes the turn, forming a 10-membered ring.^30^ This weak interaction is complemented by stereoelectronics of the glycosidic bonds (i.e., exoanomeric effect), the van der Waals and hydrophobic interactions between the methyl group of Rha and the hydrophobic face of Glc, and a steric contribution connected to the amide moiety of GlcNAc.^30,31^ This turn proved suitable to hold two cellulose branches in an ideal parallel orientation resulting in a folded hairpin conformation.^20^

We designed and synthesized new hairpin analogues carrying the same cellulose strands to investigate the effect of specific chemical changes at the turn unit on the overall hairpin conformational stability (Figure 2 and see Supporting Information Section 3). **9mer-I-Linker** carries an amino-pentanol linker^32^ at the reducing end preventing anomerization. **9mer-II** is a control structure missing the Rha unit (i.e., lacking the unconventional H-bond). **9mer-III** is based on a reducing d-Glc branching unit (instead of GlcNAc) lacking the stabilizing acetyl amido group. In **9mer-IV**, the position of the branches (l-Rha and d-Glc) is inverted to promote a different orientation of the two stacking strands. The inversion of l-Rha and d-Glc should preserve the close conformation of the turn, as shown for the trisaccharide regio-isomers Le^x^ and Le^a^.^31^

All compounds were modeled computationally by MD simulations. For each structure, the average distance between residues on the opposite strands was measured, plotted, and compared with the values obtained for **9mer-II**, which adopts an open conformation.^20^ MD simulations indicated no significant differences for the four hairpins containing a Rha unit (Figure 2a and Figures S7–S10), all adopting the closed conformation for most of the simulation time.

The chemical shift deviation (Δδ) of Rha-5 served as an experimental marker of the closed conformation.^30,33^ We monitored the chemical shift of Rha-5 for all 9mer hairpins and compared it to a control trisaccharide lacking the H-bond **3mer-V** (δ = 4.06 ppm). All compounds preserved the nonconventional H-bond over a wide temperature range, as indicated by the downfield shift of Rha-5 (Δδ ∼ 0.4 ppm) (Figure 2b and Table S4). Moreover, the presence of a key inter-residue NOE Rha-6/Glc B-2 confirmed the conformational stability of all turn motives (Figures S44, S49, and S52).

Overall, the similar results obtained for **9mer-I** and **9mer-III** suggested the limited contribution of the amido group to hairpin stabilization. Similarly, a comparison between **9mer-I** and **9mer-I-Linker** demonstrated that anomerization is not a source of flexibility. The largest chemical shift deviation was detected for **9mer-IV**. This observation indicated a higher conformational stability for the **9mer-IV** analogue, in agreement with the reported conformational stability of Le^a^ vs Le^x^.^31^ Analysis of the MD trajectories indicated a twisted conformation of the two strands with opposite directionality for **9mer-III** and **9mer-IV** (Figure S12). Further investigations are needed to confirm such helical propensity and its impact on hairpin stabilization.

More drastic differences were detected when comparing shorter hairpins (**5mer-I** vs **5mer-III**, Figure S33), indicating a major contribution of strand–strand interactions to the overall hairpin conformation (see Supporting Information Section 4.3.2 for a detailed comparison). Thus, we moved on to analyze the stabilizing effect of the strands.

### The Effect of the Strands

**Figure 3:**
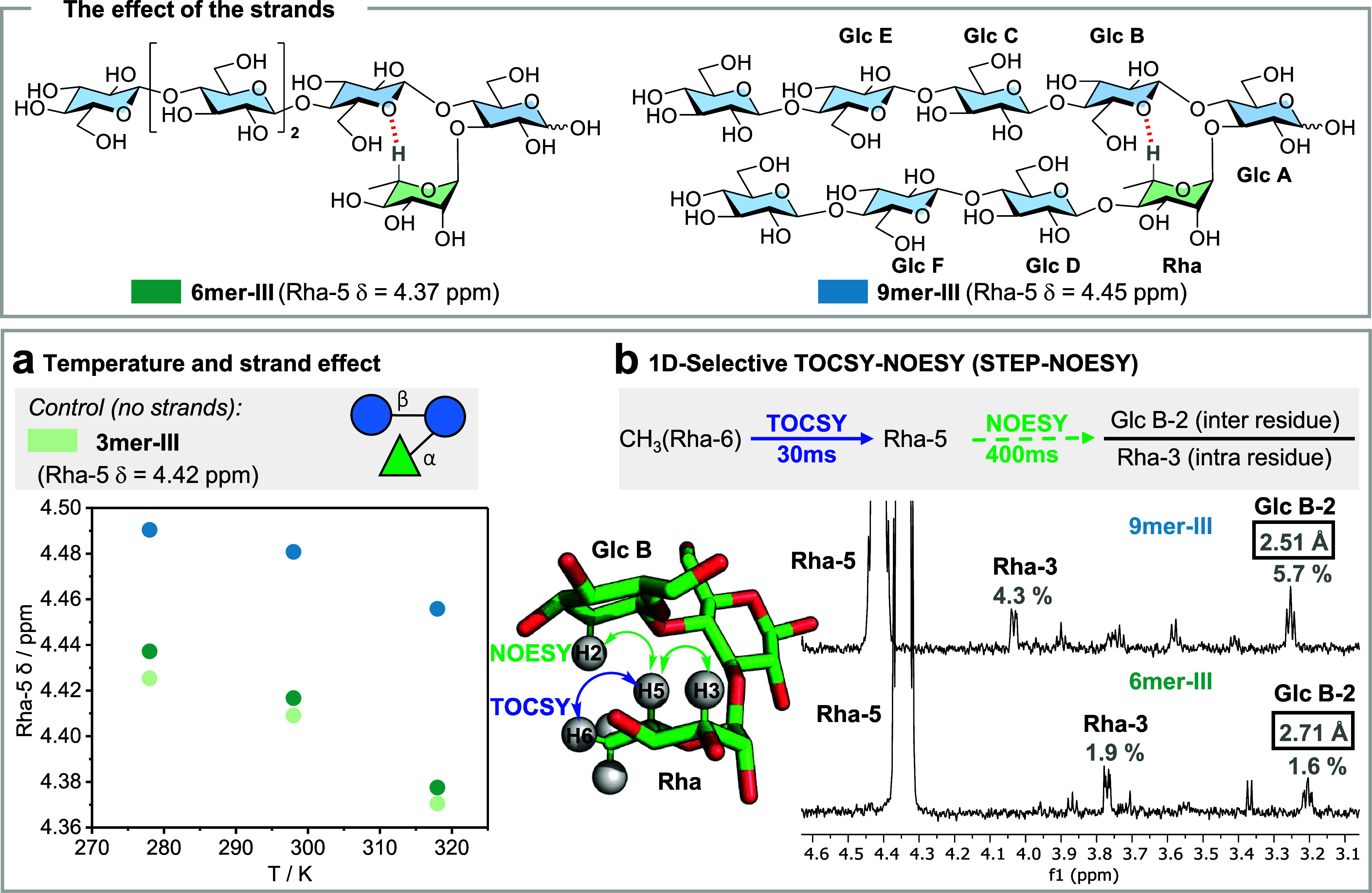
The effect of the strands: Chemical structure of the half hairpin **6mer-III** lacking one strand and **9mer-III**. (a) Temperature-dependent ^1^H NMR analysis of **9mer-III**, **6mer-III**, and **3mer-III**. The chemical shifts of Rha-5 were extracted from selective 1D TOCSY (278, 298, and 318 K, mixing time 200 ms, D_2_O, 700 MHz). The downfield shift of Rha-5 in **9mer-III** compared to **6mer-III** and **3mer-III** shows increased conformational stability due to strand–strand interactions. (b) Excerpt of the NOEs from 1D-selective TOCSY-NOESY (STEP-NOESY) NMR of **9mer-III** and **6mer-III** showing the intraresidue (Rha-5/Rha-3) NOEs and the inter-residue (Rha-5/Glc B-2) NOEs of the turn (298 K, mixing time 400 ms, D_2_O, 800 MHz).

To analyze the stabilizing effect of strand–strand interactions, experimental NMR data for **9mer-III** and **6mer-III** were compared. The latter is a truncated hairpin composed of a turn unit but lacking one of the strands. The Rha-5 chemical shift was used as an indicator of turn rigidity, showing a higher value for **9mer-III** (δ = 4.45 ppm) in comparison to **6mer-III** (δ = 4.37 ppm) (Table S4). The difference between the two compounds became even more evident at a higher temperature as the gap between Rha-5 chemical shifts increased (Figure 3a). In contrast, the behavior of **6mer-III** was virtually identical to that of **3mer-III**, the control compound lacking both strands. 1D-selective TOCSY-NOESY experiments were performed to further corroborate this observation (Figure 3b). Selective irradiation of Rha-6 followed by TOCSY transfer of magnetization to Rha-5 and then NOESY from Rha-5 permitted us to measure the intraresidue Rha-5/Rha-3 (as reference) and the inter-residue Rha-5/Glc B-2 NOEs for **6mer-III** and **9mer-III**.

The NOE data confirmed the shorter Rha-5/Glc B-2 distance in **9mer-III** (2.51 Å) than that in **6mer-III** (2.71 Å) (Figure 3b), demonstrating that strand–strand interactions rigidify the turn motif. Besides the turn, the close proximity between the two strands is encoded in the Glc C-1 chemical shift (Figure S72). Upfield Glc C-1 shifts (δ ≤ 4.40 ppm) are observed only when residue Glc D on the opposite strand was present, while for the **6mer-III**, it was significantly downfield shifted (δ 4.45 ppm).

Ramachandran plots for a Glc–Glc glycosidic bond within the strands were compared for **9mer-III**, **6mer-III**, and a cellulose hexamer as control. While the linkage in **6mer-III** showed a similar dihedral distribution with the cellulose hexamer, **9mer-III** displayed a substantial shift toward negative Ψ values (Figure S13). This indicated that interactions between strands promoted the flattening of the structure, thus favoring the formation of the conventional H-bond between Glc residues.^34^ Overall, these experimental results confirmed the important contribution of strands’ interactions in the stabilization of the hairpin conformation.

### The Effect of Strand Length

Having proved the contribution of the strand–strand interactions on the overall conformational stability of the hairpin, we moved on to analyze the effect of the strands’ length. A length-dependent stabilization has been demonstrated for specific peptide sequences folding into a hairpin conformation. In anti-parallel beta-sheet models, strands composed of five to seven residues provided optimal stabilization and further elongation did not lead to an increased stabilization but rather a loss of conformational rigidity.^35^ This behavior was not observed in parallel-β-sheet peptide models, which become steadily more stable as the strands are lengthened.^36^

To analyze the correlation between conformational stability and strand length, we prepared a collection of hairpins based on **3mer-III** substituted with strands of increasing length (**5mer-III** to **17mer-III**). This series was complemented with a stapled glycan hairpin (**5mer-III-Closed**) where the nonreducing ends were covalently connected with an aliphatic chain. This compound served as a “fully folded” reference structure as commonly done for the structural analysis of peptide hairpins.^24,37^

**5mer-III-Closed** was prepared by AGA following a ring closing metathesis (RCM) protocol.^38^ MD simulations of **5mer-III** were analyzed to determine the most suitable hydroxyl groups for the introduction of the alkene moieties.

To constrain our hairpin in the closed conformation, we aimed to staple two hydroxyl groups pointing in the same orientation for most of the simulation time. Analysis of the dihedral angles O_top,*n*_ – C_top,*n*_ – C_bottom,*m*_ – O_bottom,*m*_ and the average distance of the corresponding oxygen atoms identified the hydroxyl groups at C_top,4_ and C_bottom,4_ as ideal positions for stapling (see Supporting Information Section 4.3.3 for detailed analysis). Thus, **5mer-III-Closed** was prepared using the corresponding C-4 alkene-functionalized BBs (Figure S1 and see Supporting Information Section 3).

**Figure 4:**
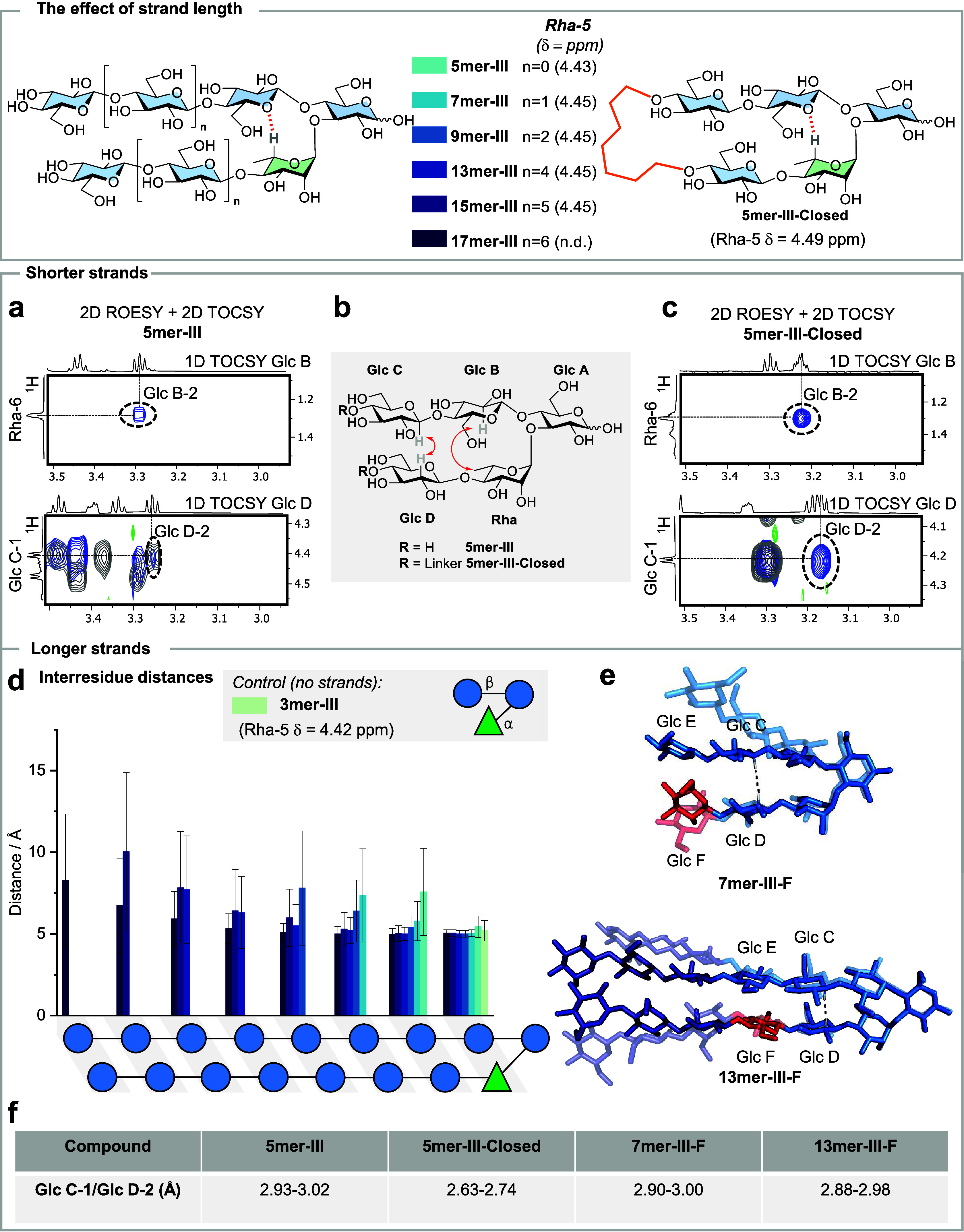
The effect of strand length: A collection of hairpin analogues with increasing strand length from **5mer-III** to **17mer-III** and a stapled glycan hairpin **5mer-III-Closed** as a “fully folded” reference. (a) Superimposed 2D ROESY (blue) and 2D TOCSY (grey) NMR of **5mer-III** showing the key interstrand NOEs with calculated distances (293 K, mixing times p15 300 ms and d9 160 ms, D_2_O, 700 MHz). (b) Experimental interstrand NOEs extracted form ROESY NMR experiments for **5mer-III** and **5mer-III-Closed** (red arrows). (c) Overimposed 2D ROESY and 2D TOCSY NMR of **5mer-III-Closed** (293 K, mixing times d9 150 ms and p15 300 ms, D_2_O, 700 MHz). (d) Average inter-residue distances calculated by MD showing a trend of increased stabilization for hairpin core with increasing strands length. (e) Overimposition of two representative snapshots extracted from MD simulation showing that the cores (light blue) of the longer hairpin (**13mer-III-F**) are more rigid than those of the shorter hairpin (**7mer-III**). The highlighted (red) F residue indicates the ^13^C-labeled unit. (f) Comparison of interstrand NOE (Glc C-1/Glc D-2) distance for **5mer-III**, **5mer-III-Closed**, **7mer-III-F**, and **13mer-III-F**. The calculated interstrand NOE distance for the **5mer-III-Closed** hairpin is closer than that for **5mer-III**, showing a certain degree of flexibility in shorter hairpin, while stability increases with strand elongation.

With this collection in hand, we set to identify whether the conformational stabilization increases linearly or whether there is an optimal length after which stabilization is lost. First, we focused on hairpins with shorter strands. The conformation of **5mer-III** and **5mer-III-Closed** in aqueous solution was compared using NMR spectroscopy (Figures S28 and S38). A downfield shift was observed for Rha-5 of **5mer-III-Closed** (4.49 ppm) as compared to **5mer-III** (4.43 ppm) (Figure 4 and Table S4). The downfield shift for Rha-5 was linearly correlated with a high field ^1^H NMR chemical shift for Glc C-1 (Figure S72). 2D ROESY and TOCSY NMR experiments were performed to confirm the spatial proximity between key inter-residue protons of the turn and the strands. The analysis of ROESY^39^ spectra showed inter-residue NOE signals (Glc B-2/Rha-6 and Glc C-1/Glc D-2) for both structures, albeit of different intensities (Figure 4b). Indeed, a closer interstrand distance was calculated for **5mer-III-Closed** (2.63–2.74 Å, Figure 4c,f) in comparison to **5mer-III** (2.93–3.02 Å, Figure 4a,f). Overall, these results indicated a certain degree of flexibility for hairpins with short strands.

Next, we explored the effect of longer strands on the overall hairpin conformation. The chemical shift of Rha-5 (4.45 ppm for structures longer than **7mer**) showed stabilization of the turn motif with strand elongation (Figure 4 and Table S4). Similarly, the comparison of inter-residue distances for all the simulated hairpin-III structures identified an increased conformational rigidity for the hairpin cores with longer strands (Figure 4d). While **5mer-III** was in equilibrium between closed and a few open forms, longer analogues were closed for the majority of the simulation time (Figures S20–S23). To support the MD simulation results with solution-state NMR, we looked for the interresidue NOEs in short vs long hairpin structures. To simplify the NMR analysis, we introduced a ^13^C-labeled residue^40^ (highlighted in red color, Figure 4e) in the same position of the bottom strand in a short and in a long hairpin (**7mer-III-F** and **13mer-III-F**). The use of a ^13^C-labeled unit (Glc F) allowed us to identify the NMR signals for its contiguous Glc D residue and to calculate the interstrand distance between Glc C-1 and Glc D-2 (Figure 4f). A shorter distance was estimated for the long **13mer-III-F** (2.88–2.98 Å) than for the short **7mer-III-F** (2.90–3.00 Å). Moreover, the presence of ^13^C-labels in residue F was instrumental to detecting a key interstrand NOE between Glc F-6 and Glc E-2. This NOE was detected only for the long **13mer-III-F** (Figure S69). In contrast, the analogous NOE (between Glc F-6 and Glc E-2) could not be detected in the shorter **7mer-III** analogue, likely due to the existence of internal motions of the E and F units. Given the dependence of the NOEs on the effective correlation times, these fast motions at the edges of **7mer-III** result in smaller and undetectable NOEs. Taken together, these analyses indicated that the glycan hairpin becomes steadily more stable as the strands are lengthened.

**Figure 5:**
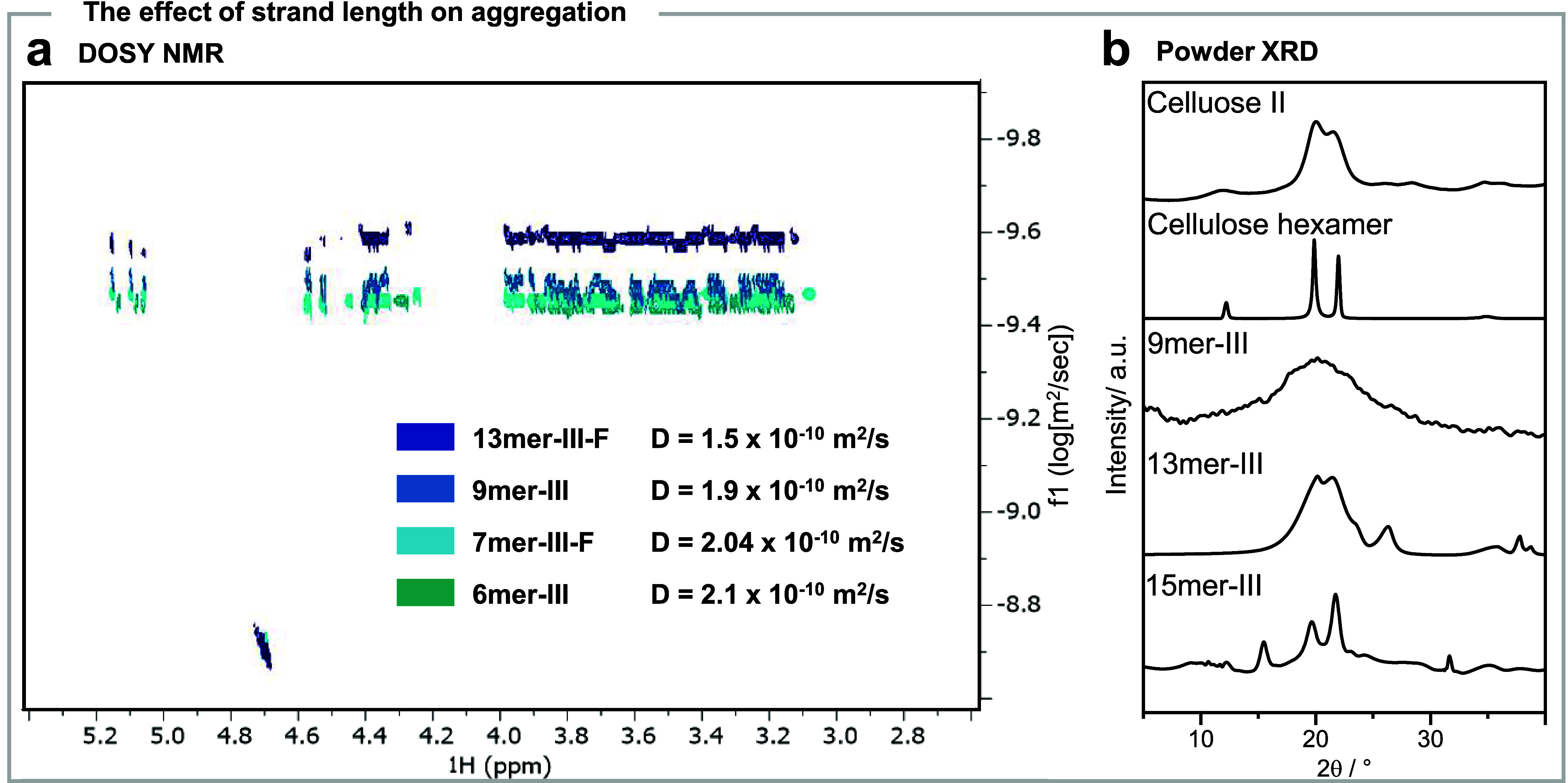
The effect of strand length on aggregation. (a) Superimposed DOSY spectra of **6mer-III**, **7mer-III-F**, **9mer-III**, and **13mer-III-F** (298 K, D_2_O, 800 MHz). (b) Powder X-ray diffraction (XRD) profiles for hairpin analogues show that **9mer-III** is amorphous, **13mer-III-F** has a cellulose-II molecular packing, and **15mer-III** is a mixture of cellulose allomorphs (cellulose II and IV_II_).

Besides increasing conformational stability, long cellulose strands could engage in intermolecular interactions.^41^ DOSY analysis confirmed the aggregation tendency for longer hairpin analogues (Figure 5a). While the diffusion coefficient decreased linearly for short structures, a significant jump was detected for **13mer-III**, suggesting the presence of certain aggregation. This result is in agreement with the observation of a precipitate forming during the synthesis, drastically decreasing the yield of the long analogues (**17mer-III**). Powder XRD analysis showed a crystalline packing for analogues longer than **9mer**, with **13mer-III** resembling the cellulose II crystal structure^42^ and **15mer-III** showing a mixture of two allomorphs (i.e., cellulose II and cellulose IV_II_)^43^ (Figure 5b). This tendency may be exploited in the future for the controlled formation of carbohydrate assemblies.^44^

### Conclusions

We analyzed the factors that influence the conformational stability of a glycan hairpin. The modularity of the hairpin design allowed us to systematically scrutinize the effect of chemical changes in the turn or in the strand length. We discovered that the conformational proclivity of the turn motif is fundamental to promoting folding but that some modifications (e.g., inversion of the strand orientation or functionalization of the reducing end) are tolerated and could be exploited to tune the hairpin geometry or for further functionalization. We identified the substantial contribution of strand–strand interactions to the overall hairpin conformational stability, which increased with the strand length. These results demonstrate that weak glycan–glycan interactions can be exploited to rigidify glycan sequences, normally considered to be highly flexible molecules. This finding is valuable for the design of rigid glycomimetics, capable of minimizing the entropic penalty of binding.^45^ Our systematic analysis also revealed the aggregation tendency and formation of crystalline materials for hairpins longer than 13mer. This feature could inspire the design of carbohydrate materials with tunable geometries,^44^ as extensively explored for peptide hairpins.^13,46^

## Data Availability

The authors declare that all data supporting the findings of this study are available within the article and in the Supporting Information files. Raw data for NMR analysis and MD simulations can be downloaded from 10.17617/3.ZCGFCC, Edmond. Data are also available from the corresponding author upon request.

## Supporting Information Available

General materials and methods; building blocks; automated glycan assembly; and structural analysis (PDF)
