# Supplementary material for: Dissecting the Conformational Stability of a Glycan Hairpin
Source: J Am Chem Soc. 2024 Feb 20;146(9):6369–76. doi: 10.1021/jacs.4c00423 (PMC10921397; doi:10.1021/jacs.4c00423)
Supplement: Supplementary file 1 — ja4c00423_si_001.pdf [file ja4c00423_si_001.pdf]

Supporting Information

Dissecting the conformational stability of a glycan hairpin

Nishu Yadav,[1,2] Surusch Djalali,[1,2] Ana Poveda,[3] Manuel G. Ricardo,[1] Peter H. Seeberger,[1,2] Jesús Jiménez-Barbero,[3,4,5,6] and Martina Delbianco[1*]

[1]Department of Biomolecular Systems, Max Planck Institute of Colloids and Interfaces, Am Mühlenberg 1, 14476, Potsdam, Germany

[2]Department of Chemistry and Biochemistry, Freie Universität Berlin, Arnimallee 22, 14195, Berlin, Germany

[3]CICbioGUNE, Basque Research and Technology Alliance, 48160, Derio, Spain

[4]Ikerbasque, Basque Foundation for Science, 48009, Bilbao, Spain

[5]Department of Inorganic & Organic Chemistry, Faculty of Science and Technology, University of the Basque Country, EHU-UPV, 48940, Leioa, Spain

[6]Centro de Investigación Biomedica En Red de Enfermedades Respiratorias, 28029, Madrid, Spain

Table of contents

1 General materials and methods **4**

2 Building blocks **5**

3 Automated glycan assembly **6**

3.1 General materials and methods 6

3.2 Preparation of stock solutions 6

3.3 Modules for automated synthesis 6

3.3.1 Module A: Resin preparation 6

3.3.2 Module B: Acidic wash with TMSOTf solution (20 min) 6

3.3.3 Module C1: Thioglycoside glycosylation (35 min-55 min) (NIS : 0.15 mmol/mL) 7

3.3.4 Module C2: Thioglycoside glycosylation (35 min-55 min) (NIS : 0.09 mmol/mL) 7

3.3.5 Module C3: Glycosyl phosphate glycosylation (45 min) 8

3.3.6 Module D: Capping (30 min) 8

3.3.7 Module E1: Fmoc deprotection (9 min) 9

3.3.8 Module E2: Lev deprotection (90 min) 9

3.4 Post-AGA manipulations 10

3.4.1 Module F: On-resin cyclisation 10

3.4.2 Module G: On-resin methanolysis 10

3.4.3 Module H1: Cleavage from solid support 10

3.4.4 Module H2: Micro-cleavage from solid support 10

3.4.5 Module I1: Hydrogenolysis 10

3.4.6 Module I2: Hydrogenolysis at ambient pressure 10

3.4.7 Module J: Purification 10

3.5 Terminology 12

3.6 Oligosaccharide synthesis 13

3.6.1 5mer-III 14

3.6.2 5mer-III-closed 18

3.6.3 9mer-I-Linker 23

3.6.4 9mer-III 27

3.6.5 9mer-IV 35

3.6.6 13mer-III 40

3.6.7 15mer-III 44

3.6.8 17mer-III 49

3.6.9 7mer-III-F 53

3.6.10 13mer-III-F 57

4 Structural analysis **61**

4.1 General materials and methods for Molecular dynamics simulations 61

4.2 General materials and methods for NMR 61

4.3 Molecular dynamics 62

4.3.1 9mers and 6mer-III 62

4.3.2 5mer-III ... 69
4.3.3 5mer-III-Closed ... 74
4.3.4 Length comparision ... 75
4.4 NMR studies ... 79
4.4.1 NMR characterization of 5mer-III ... 79
4.4.2 5mer-I vs 5mer-III ... 85
4.4.3 NMR characterization of 5mer-III-Closed ... 86
4.4.4 NMR characterization of 9mer-I-Linker ... 92
4.4.5 NMR characterization of 9mer-III ... 94
4.4.6 NMR characterization of 9mer-IV ... 97
4.4.7 Effect of temperature, turn unit and strand length on hairpin stability ... 99
4.4.8 Determination of the non-conventional H-bond ... 101
4.4.9 NMR characterization of 7mer-III-F ... 102
4.4.10 NMR characterization of 13mer-III-F ... 107
4.4.11 Comparative analysis of the 6mer-III, 7mer-III-F, 9mer-III and 13mer-III-F analogues ... 112
4.4.12 Diffusion experiments of the 6mer-III, 7mer-III-F, 9mer-III and 13mer-III-F analogues 113
4.4.13 Chemical shift perturbation (CSP) analysis for Rha-5 and Glc C-1 ... 114
4.4.14 Summary of NMR analysis ... 115
5 References ... 116

1 General materials and methods

All chemicals used were reagent grade and used as supplied unless otherwise noted. The automated syntheses were performed on a home-built synthesizer developed at the Max Planck Institute of Colloids and Interfaces.[1]. Analysis and purification by normal and reverse phase HPLC were performed by using an Agilent 1200 series. Products were lyophilized using a Christ Alpha 2-4 LD plus freeze dryer. ^{1}H, ^{13}C and HSQC NMR spectra were recorded on a Varian 400-MR (400 MHz), Varian 600-NMR (600 MHz), Bruker Biospin AVANCE700 (700 MHz) and Bruker AVANCE III 800 (800 MHz) spectrometer. Spectra were recorded in $CDCl_3$ by using the solvent residual peak chemical shift as the internal standard ($CDCl_3$: 7.26 ppm ^{1}H, 77.0 ppm ^{13}C) or in D_2O using the solvent as the internal standard in ^{1}H NMR (D_2O: 4.79 ppm ^{1}H). ^{1}H NMR spectra for all compounds were recorded without ^{13}C decoupling. Weak intensity ^{13}C resonances were derived from the respective HSQC crosspeaks. ^{1}H NMR integrals of the resonances corresponding to residues at the reducing end are reported as non-integer numbers and the sum of the integrals of α and β anomers is set to 1. High resolution mass spectra were obtained using a 6210 ESI-TOF mass spectrometer (Agilent) and a MALDI-TOF autoflexTM (Bruker).

2 Building blocks

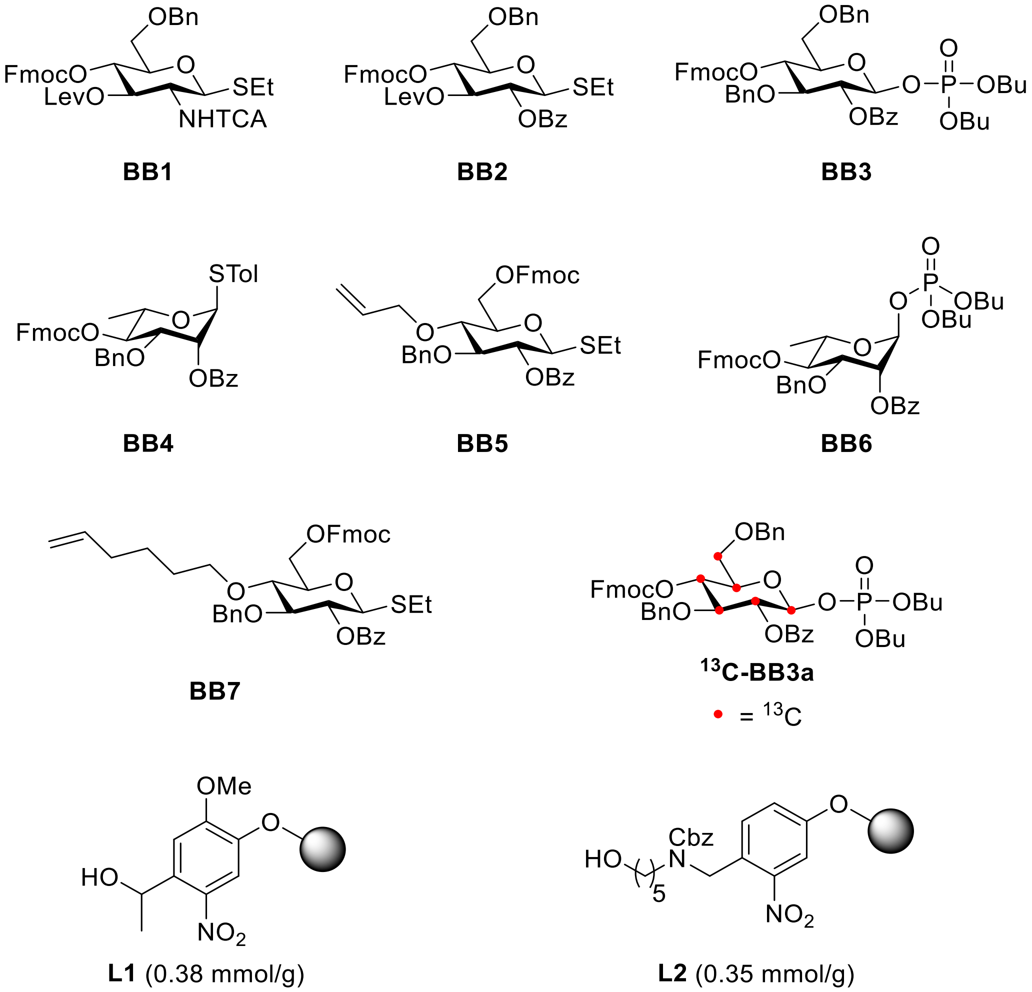

Figure S1 BBs and solid supports used in this work. Loading of **L1** and **L2** is reported in parenthesis.

BB1 was purchased from GlycoUniverse (Germany). **BB2**, **^{13}C-BB3a,** and **BB4** were synthesized according to previously reported procedures.[2] **BB3** and **BB6** was synthesized according to previously reported procedure.[3] **BB5** and **BB7** were synthesized according to previously reported procedure.[4] Merrifield resin equipped with photocleavable linkers **L1** (loading 0.38 mmol/g) or **L2** (loading 0.35 mmol/g) was prepared according to previously reported procedures.[5]

3 Automated glycan assembly

3.1 General materials and methods

The automated syntheses were performed on a home-built synthesizer developed at the Max Planck Institute of Colloids and Interfaces.[1] All solvents used were HPLC-grade. The solvents used for the building blocks, activator, TMSOTf and capping solutions were taken from an anhydrous solvent system (J.C. Meyer). The building blocks were co-evaporated three times with toluene and dried for 1 h on high vacuum before use. Oven-heated, argon-flushed flasks were used to prepare all moisture-sensitive solutions. Activator, capping, deprotection, acidic wash and building block solutions were freshly prepared and kept under argon during the automation run. All yields of products obtained by AGA were calculated on the basis of resin loading. Resin loading was determined following previously established procedures.[6]

3.2 Preparation of stock solutions

- **Building block solution**: Between 0.06 and 0.10 mmol of building block (depending on the BB, see Module C1 and C2) was dissolved in DCM (1 mL).
- **NIS/TfOH activator solution**: 1.35 g (6.0 mmol) of recrystallized NIS was dissolved in 40 mL of a 2:1 v/v mixture of anhydrous DCM and anhydrous dioxane. Then triflic acid (55 µL, 0.6 mmol) was added. The solution was kept at 0 °C (ice bath) for the duration of the automation run.
- **Fmoc deprotection solution**: A solution of 20%$_{v/v}$ piperidine in DMF was prepared.
- **Lev deprotection solution**: Hydrazine acetate (550 mg, 5.97 mmol) was dissolved in pyridine/AcOH/H_2O (40mL, v/v, 32:8:2) and sonicated for 10 min.
- **TMSOTf solution**: TMSOTf (0.45 mL, 2.49 mmol) was added to DCM (40 mL).
- **Capping solution**: A solution of 10%$_{v/v}$ acetic anhydride and 2%$_{v/v}$ methanesulfunic acid in DCM was prepared.

3.3 Modules for automated synthesis

3.3.1 Module A: Resin preparation

All automated syntheses were performed on 0.0125 mmol scale. Resin (**L1** or **L2**) is placed in the reaction vessel and swollen in DCM for 20 min at room temperature prior to the synthesis. During this time, all reagent lines needed for the synthesis are washed and primed. After the swelling, the resin is washed with DMF, THF, and DCM (three times each with 2 mL for 25 s).

3.3.2 Module B: Acidic wash with TMSOTf solution (20 min)

The resin is swollen in 2 mL DCM and the temperature of the reaction vessel adjusted to -20 °C. Upon reaching the low temperature, TMSOTf solution (1 mL) is added dropwise to the reaction vessel. After bubbling for 3 min, the acidic solution is drained and the resin washed with 2 mL DCM for 25 s.

Action	Cycles	Solution	Amount	T (°C)	Incubation time
**Cooling**	-	-	-	-20	(15 min)*
**Deliver**	1	DCM	2 mL	-20	-
**Deliver**	1	TMSOTf solution	1 mL	-20	3 min
**Wash**	1	DCM	2 mL	-20	25 sec

*Time required to reach the desired temperature.

3.3.3 Module C1: Thioglycoside glycosylation (35 min-55 min) (NIS: 0.15 mmol/mL)

The building block solution (0.10 mmol of BB in 1 mL of DCM per glycosylation) is delivered to the reaction vessel. After the set temperature is reached, the reaction is started by dropwise addition of the NIS/TfOH activator solution (1.0 mL, 0.15 mmol). The glycosylation conditions (T_1, T_2, t_1, and t_2) are building block dependent and are reported in a table below. After completion of the reaction, the solution is drained and the resin was washed with DCM, DCM:dioxane (1:2, 3 mL for 20 s) and DCM (two times, each with 2 mL for 25 s). The temperature of the reaction vessel is increased to 25 °C for the next module. In case of a double cycle (C1*, *Double cycle), module C1 is repeated twice.

Action	Cycles	Solution	Amount	T (°C)	Incubation time
**Cooling**	-	-	-	T_1	-
**Deliver**	1	BB solution	1 mL	T_1	-
**Deliver**	1	NIS/TfOH activator solution	1 mL	T_1	-
**Reaction time (BB dependent)**	1	-	-	T_1 to T_2	t_1 t_2
**Wash**	1	DCM	2 mL	T_2	5 sec
**Wash**	1	DCM : Dioxane (1:2)	2 mL	T_2	20 sec
**Heating**	-	-	-	25	-
**Wash**	2	DCM	2 mL	> 0	25 sec

BB	Equiv.	t_1 (min)	T_1 (°C)	t_2 (min)	T_2 (°C)
**BB1**	6.5	5	-20	40	0
**BB2**	6.5	5	-20	20	0
**BB4**	6.5	5	-20	20	0
**BB5**	6.5	5	-20	20	0

3.3.4 Module C2: Thioglycoside glycosylation (35 min-55 min) (NIS: 0.09 mmol/mL)

The building block solution (0.10 mmol of BB in 1 mL of DCM per glycosylation) is delivered to the reaction vessel. After the set temperature is reached, the reaction is started by dropwise addition of the NIS/TfOH activator solution (1.0 mL, 0.09 mmol). The glycosylation conditions (T_1, T_2, t_1, and t_2) are building block dependent and are reported in a table below. After completion of the reaction, the solution is drained and the resin was washed with DCM, DCM:dioxane (1:2, 3 mL for 20 s) and DCM (two times, each with 2 mL for 25 s). The temperature of the reaction vessel is increased to 25 °C for the next module. In case of a double cycle (C2*, *Double cycle), module C2 is repeated twice.

Action	Cycles	Solution	Amount	T (°C)	Incubation time
**Cooling**	-	-	-	T_1	-
**Deliver**	1	BB solution	1 mL	T_1	-

**Deliver**	1	NIS/TfOH activator solution	1 mL	T_1	-
**Reaction time (BB dependent)**	1	-	-	T_1 to T_2	t_1 t_2
**Wash**	1	DCM	2 mL	T_2	5 sec
**Wash**	1	DCM : Dioxane (1:2)	2 mL	T_2	20 sec
**Heating**	-	-	-	25	-
**Wash**	2	DCM	2 mL	> 0	25 sec

BB	Equiv.	t_1 (min)	T_1 (°C)	t_2 (min)	T_2 (°C)
**BB7**	6.5	25	-25	10	-10

3.3.5 Module C3: Glycosyl phosphate glycosylation (45 min)

The building block solution (0.06 mmol of BB in 1 mL of DCM per glycosylation) is delivered to the reaction vessel. After the set temperature is reached, the reaction is started by dropwise addition of the TMSOTf solution (1.0 mL, stoichiometric). After completion of the reaction, the solution is drained and the resin washed with DCM (six times, each with 2 mL for 25 s). The temperature of the reaction vessel is increased to 25 °C for the next module. In case of a double cycle (C3*, *Double cycle), module C3 is repeated twice.

Action	Cycles	Solution	Amount	T (°C)	Incubation time
**Cooling**	-	-	-	-30	-
**Deliver**	1	BB solution	1 mL	-30	-
**Deliver**	1	TMSOTf solution	1 mL	-30	-
**Reaction time (BB dependent)**	1	-	-	-30 to -10	5 min 40 min
**Wash**	1	DCM	2 mL	-10	5 sec
**Heating**	-	-	-	25	-
**Wash**	6	DCM	2 mL	> 0	25 sec

BB	Equiv.	t1 (min)	T1 (°C)	t2 (min)	T2 (°C)
**BB3**	5	5	-30	40	-10
**BB3a**	5	5	-30	40	-10
**BB6**	5	5	-30	40	-10

3.3.6 Module D: Capping (30 min)

The resin is washed with DMF (two times with 2 mL for 25 s) and the temperature of the reaction vessel adjusted to 25 °C. A pyridine solution (2 mL, 10%$_{v/v}$ in DMF) is delivered into the reaction vessel. After 1 min, the reaction solution is drained and the resin washed with DCM (three times with 3 mL for 25 s).

Capping solution (4 mL) is delivered into the reaction vessel. After 20 min, the reaction solution is drained and the resin washed with DCM (three times with 3 mL for 25 s).

Action	Cycles	Solution	Amount	T (°C)	Incubation time
**Heating**	-	-	-	25	(5 min)*
**Wash**	2	DMF	2 mL	25	25 sec
**Deliver**	1	10% Pyridine in DMF	2 mL	25	1 min
**Wash**	3	DCM	2 mL	25	25 sec
**Deliver**	1	Capping Solution	4 mL	25	20 min
**Wash**	3	DCM	2 mL	25	25 sec

*Time required to reach the desired temperature.

3.3.7 Module E1: Fmoc deprotection (9 min)

The resin is washed with DMF (three times with 2 mL for 25 s) and the temperature of the reaction vessel adjusted to 25 °C. Fmoc deprotection solution (2mL) is delivered to the reaction vessel and kept under Ar bubbling. After 5 min, the reaction solution is drained and the resin washed with DMF (three times with 3 mL for 25 s) and DCM (five times each with 2 mL for 25 s). The temperature of the reaction vessel is decreased to -20 °C for the next module.

Action	Cycles	Solution	Amount	T (°C)	Incubation time
**Wash**	3	DMF	2 mL	25	25 sec
**Deliver**	1	Fmoc depr. solution	2 mL	25	5 min
**Wash**	1	DMF	2 mL		
**Cooling**	-	-	-	-20	-
**Wash**	3	DMF	2 mL	< 25	25 sec
**Wash**	5	DCM	2 mL	< 25	25 sec

3.3.8 Module E2: Lev deprotection (90 min)

The resin is washed with DCM (three times with 2 mL for 25 s). DCM (1.3 mL) is delivered to the reaction vessel and the temperature of the reaction vessel is adjusted to 30 °C. Lev deprotection solution (2mL) is delivered to the reaction vessel, kept under pulsed Ar bubbling for 30 min. This procedure is repeated twice. The reaction solution is drained and the resin washed with DMF (three times with 3 mL for 25 s) and DCM (five times each with 2 mL for 25 s).

Action	Cycles	Solution	Amount	T (°C)	Incubation time
**Wash**	3	DMF	2 mL	25	25 sec
**Deliver**	2	Lev depr. solution	2 mL	25	30 min
**Wash**	1	DMF	2 mL	-	-
**Cooling**	-	-	-	-20	-

**Wash**	3	DMF	2 mL	< 25	25 sec
**Wash**	5	DCM	2 mL	< 25	25 sec

3.4 Post-AGA manipulations

3.4.1 Module F: On resin cyclization

This step is performed on a microwave peptide synthesizer Liberty Blue (CEM). The resin is washed with DCE (three times with 2 mL for 25 s) and the temperature of the reaction vessel is adjusted to 50 °C by MW heating. Grubbs 2nd gen. catalyst solution (2 µmol, 15 mol% respect to the resin loading, in 2 mL of DCE) is delivered to the reaction vessel and kept under Ar bubbling at 50 °C for 10 min. The reaction solution is drained and the procedure repeated three times. The reaction solution is drained and the resin is washed sequentially with DCE, DME, THF, and DCM (twice with 2mL for 25 s each).

3.4.2 Module G: On-resin methanolysis

The resin is suspended in THF (4 mL). MeONa in MeOH (0.5 M, 0.4 mL) is added and the suspension is gently shaken at room temperature. After micro-cleavage (see *Module H2*) indicates the complete removal of all ester groups, the resin is repeatedly washed with MeOH (3 x 2 mL) and DCM (3 x 2 mL).

3.4.3 Module H1: Cleavage from solid support

The oligosaccharides are cleaved from the solid support using a continuous-flow photoreactor as described previously.[7]

3.4.4 Module H2: Micro-cleavage from solid support

Trace amount of resin (around 20 beads) is dispersed in DCM (0.1 mL) and irradiated with a UV lamp (6 W, 356 nm) for 10 minutes. ACN (10 µL) is then added to the resin and the resulting solution analyzed by MALDI.

3.4.5 Module I1: Hydrogenolysis

The crude compound obtained from *Module H1* is dissolved in 2 mL of EtOAc:*t*BuOH:H_2O (2:1:1). 100% by weight Pd/C (10%$_w$) or $Pd(OH)_2$/C (10-20%$_w$, moistened with water) is added and the reaction stirred in a pressurized reactor under H_2 pressure (4 bar). The reaction progress is monitored to avoid undesired side products formation (*i.e.* degradation of reducing end).[8] Upon completion, the reaction is filtered (PTFE 0.45 µm 25 mm syringe filter, Fisher scientific) and washed with EtOAc, H_2O, and ACN (4 mL each). The filtrates are concentrated *in vacuo.*

3.4.6 Module I2: Hydrogenolysis at ambient pressure

The crude compound obtained from *Module H1* is dissolved in 2 mL of EtOAc:*t*BuOH:H_2O (2:1:1). 100% by weight Pd/C (10%$_w$) is added to the stirred flask, the reaction purged for 5 min with a N_2 balloon, and equipped with a H_2 balloon. The reaction progress is monitored to avoid undesired side products formation (*i.e.* degradation of reducing end).[8] Upon completion, the reaction is filtered (PTFE 0.45 µm 25 mm syringe filter, Fisher scientific) and washed with EtOAc, H_2O, and ACN (4 mL each). The filtrates are concentrated *in vacuo.*

3.4.7 Module J: Purification

The final compounds are analyzed using analytical reversed phase HPLC (Agilent 1200 Series, Methods A1, B1, and C1). The purification of the crudes is conducted using reversed phase HPLC (Agilent 1200 Series, Method B2, and C2).

- **Method A1**: (Synergi Hydro RP18 column, Phenomenex, 250 x 4.6 mm), flow rate of 1.0 mL/min with H_2O (0.1% formic acid) and ACN as eluents [isocratic (5 min), linear gradient to 20% ACN (60 min), linear gradient to 100% ACN (5 min), isocratic 100% ACN (5 min)].

- **Method A2 (Prep)**: (Synergi Hydro RP18 column, Phenomenex, 250 x 10 mm) flow rate of 4.0 mL/min with H_2O (0.1% formic acid) and ACN as eluents [isocratic (5 min), linear gradient to 20% ACN (60 min), linear gradient to 100% ACN (5 min), isocratic 100% ACN (5 min)].
- **Method B1**: (Synergi Hydro RP18 column, Phenomenex, 250 x 4.6 mm), flow rate of 1.0 mL/min with H_2O (0.1% formic acid) and ACN as eluents [isocratic (5 min), linear gradient to 30% ACN (30 min), linear gradient to 100% ACN (5 min), isocratic 100% ACN (5 min)].
- **Method B2 (Prep)**: (Synergi Hydro RP18 column, Phenomenex, 250 x 10 mm) flow rate of 4.0 mL/min with H_2O (0.1% formic acid) and ACN as eluents [isocratic (5 min), linear gradient to 30% ACN (30 min), linear gradient to 100% ACN (5 min), isocratic 100% ACN (5 min)].
- **Method C1**: (Hypercarb column, ThermoFisher scientific, 150 x 4.6 mm, 3 μm) flow rate of 0.7 mL/min with H_2O (0.1% formic acid) and ACN as eluents [isocratic (5 min), linear gradient to 60% ACN (30 min), linear gradient to 100% ACN (5 min), isocratic 100% ACN (5 min)].
- **Method C2 (Prep)**: (Hypercarb column, ThermoFisher scientific, 150 x 10 mm, 5 μm), flow rate of 3 mL/min with H_2O (0.1% formic acid) and ACN as eluents [isocratic (5 min), linear gradient to 60% ACN (30 min), linear gradient to 100% ACN (5 min), isocratic 100% ACN (5 min)].
- **Method D2 (Prep)**: (Hypercarb column, ThermoFisher scientific, 150 x 10 mm, 5 μm), flow rate of 3 mL/min with H_2O (0.1% formic acid) and ACN as eluents [isocratic (5 min), linear gradient to 50% ACN (50 min), linear gradient to 100% ACN (5 min), isocratic 100% ACN (5 min)].

Following final purification, all deprotected products are lyophilized on a Christ Alpha 2-4 LD plus freeze dryer prior to characterization.

3.5 Terminology

To standardize the name and representation of the glycan hairpins, we developed a systematic terminology. Each hairpin name describes the length, type of turn unit, and modification, giving direct information about the key features of the glycan. Each name is associated with a color code where the shade refers to the length, the color to the type of turn unit, and the pattern to the modification.

Length	Turn I	Turn III	Turn IV
3mer	#F0BBA2	#CEE6CA	#F8E87A
5mer	#EC8F96	#91D0CE	#F5D162
7mer	#E55F88	#56B9D2	#F1B054
9mer	#BD4488	#3F97C2	#EC8F43
11mer	#8D3090	#3371AA	#E66C33
13mer	#63218F	#274B93	#D35028
15mer	#4A186A	#1B277C	#96422A
17mer	#321049	#131A55	#5B372E

	Control
3mer-II	#B2DFDB
3mer-V	#4DB6AC
6mer-III	#009688
9mer-II	#00796B

	Modifier
Linker	
Alpha	
Closed	

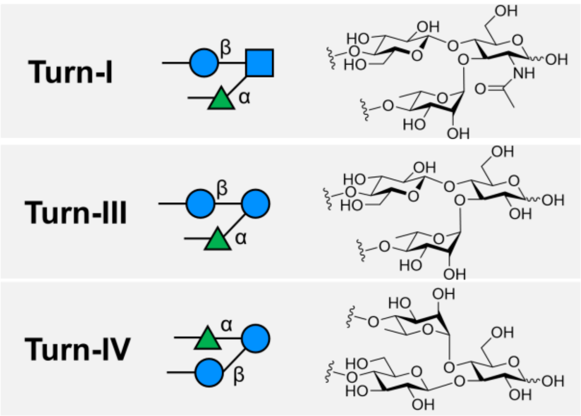

Figure S2 Left: Color code including shade gradient, color, and pattern. Right: Definition of **turn-I**, **turn-III**, and **turn-IV**.

**Example**	9mer	-	I	-	Linker
**Name code**	Length	-	Turn	-	Modifier
**Color code**	Shade	-	Color	-	Pattern
**Color**					

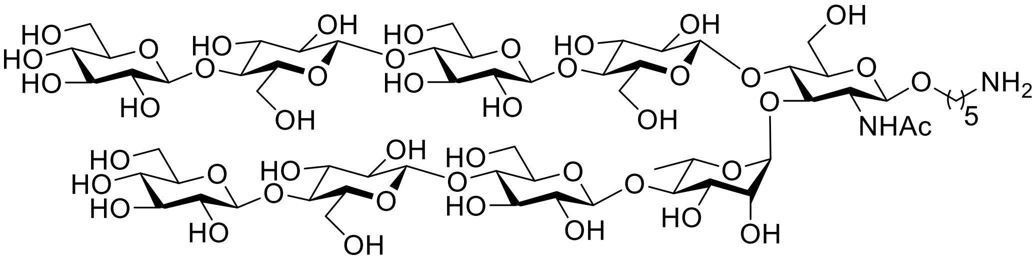

Figure S3 Terminology, corresponding color code and chemical structure for **9mer-I-Linker** as an example.

3.6 Oligosaccharide synthesis

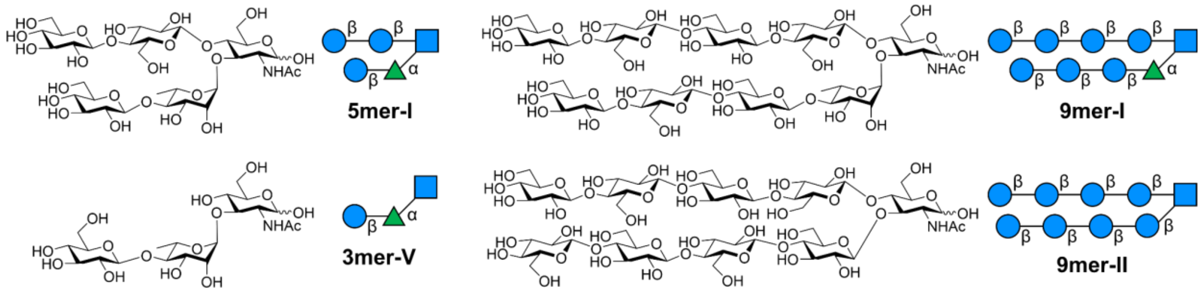

Figure S4 Oligosaccharides synthesized by AGA in previous work.[2]

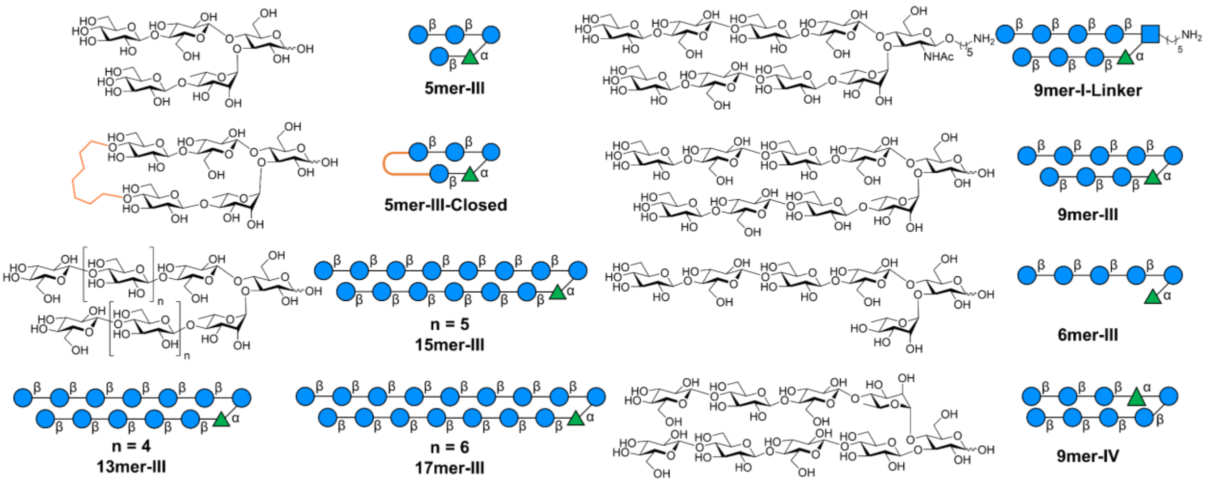

Figure S5 Oligosaccharides synthesized by AGA in this work.

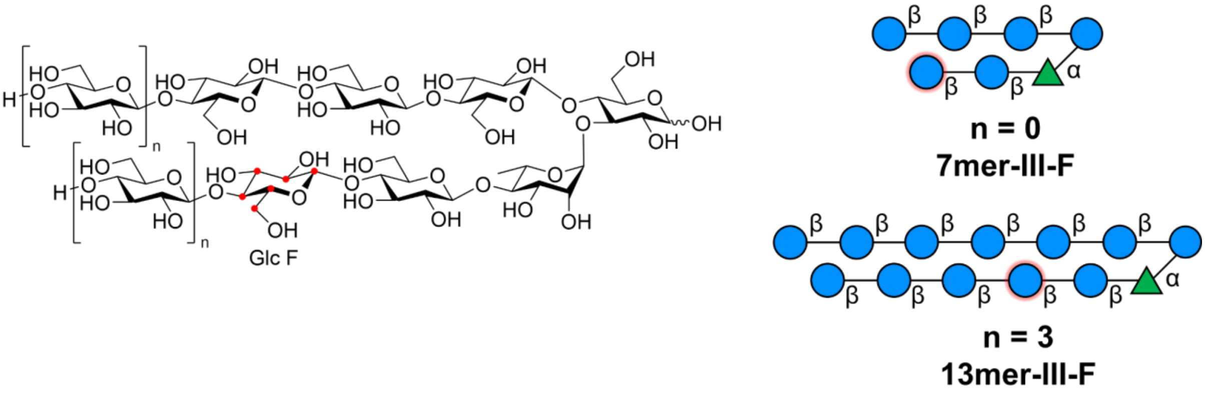

Figure S6 ^{13}C-labelled oligosaccharides synthesized by AGA in this work.

3.6.1 5mer-III

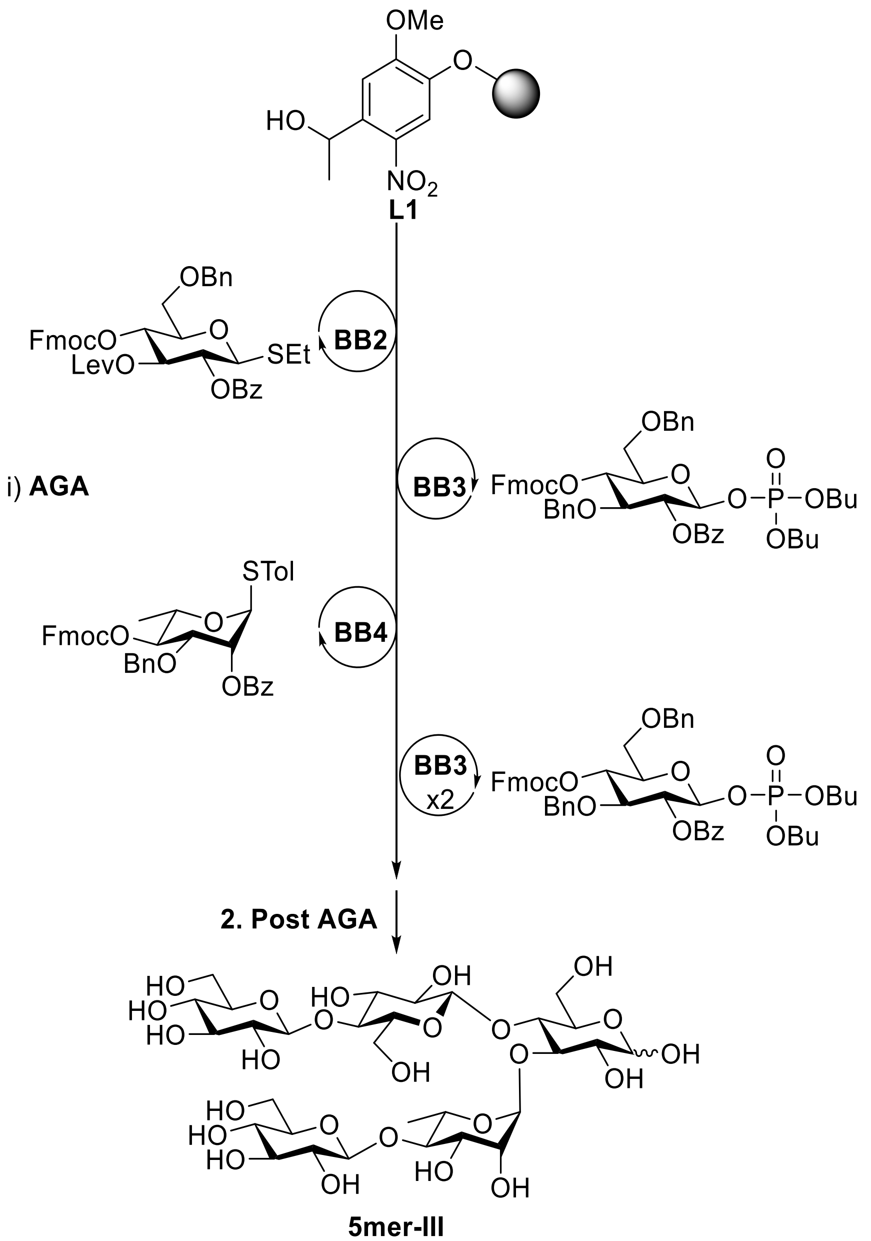

Step	BB	Modules	Notes
AGA	-	**A**	**L1** swelling
	**BB2**	**B**, **C1**, **D**, **E1**	**C1**: (**BB2**, -20 °C for 5 min, 0 °C for 20 min)
	**BB3**	**B**, **C3***, **D**<br>**E2**	**C3***: (**BB3**, -30 °C for 5 min, -10 °C for 40 min) *Double cycle
	**BB4**	**B**, **C1**, **D**, **E1**	**C1**: (**BB4**, -20 °C for 5 min, 0 °C for 20 min)
	(**BB3**)x2	(**B**, **C3***)x2<br>**D**, **E1**	**C3***: (**BB3**, -30 °C for 5 min, -10 °C for 40 min) *Double cycle
Post-AGA	-	**G**, **H1**, **I2**, **J**	**G**: (12 h)<br>**I2**: (4 h)<br>**J**: (Method C2: 16.8 min)

Automated synthesis, global deprotection, and purification afforded **5mer-III** as a white solid (3.4 mg, 32% overall yield).

^{1}H NMR (700 MHz, D_2O) δ 5.24 (s, 0.6H, H-1β Rha), 5.19 (s, 0.4H, H-1α Rha), 5.15 (d, *J* = 3.8Hz, 0.4H, H-1α Glc), 4.65 (d, *J* = 8.3 Hz, 1H, H-1 Glc), 4.62 (d, *J* = 8.0 Hz, 0.6H, H-1β Glc), 4.48 (d, *J* = 7.9 Hz, 1H, H-1 Glc), 4.44 (td, *J* = 8.4, 4.0 Hz, 1H, H-5 Rha), 4.41 (d, *J* = 7.8 Hz, 1H, H-1 Glc), 4.06 (dd, *J* = 9.7, 2.6 Hz, 1H), 4.03 (dd, *J* = 12.3, 2.4 Hz, 1H), 4.00 (d, *J* = 3.1 Hz, 1H), 3.91 (dd, *J* = 13.7, 3.3 Hz, 1H), 3.87 (d, *J* = 12.3 Hz, 2H), 3.82 (d, *J* = 4.8 Hz, 1H), 3.78 (q, *J* = 9.1 Hz, 3H), 3.69 (td, *J* = 11.6, 5.5 Hz, 2H), 3.65 – 3.62 (m, 1H), 3.59 (t, *J* = 10.3 Hz, 1H), 3.53 (tt, *J* = 7.4, 3.3 Hz, 2H), 3.50 – 3.46 (m, 3H), 3.43 (t, *J* = 9.3 Hz, 1H), 3.39 (t, *J* = 11.2 Hz, 2H), 3.37 – 3.31 (m, 2H), 3.30 – 3.24 (m, 3H), 1.28 (dd, *J* = 5.8, 2.5 Hz, 3H, CH_3-6 Rha). ^{13}C NMR (176 MHz, D_2O) δ 103.68 (C-1 Glc), 103.67, 102.81 (C-1 Glc), 101.04 (C-1 Glc), 101.01, 100.20 (C-1α Rha), 100.07 (C-1β Rha) , 95.71 (C-1β Glc), 92.00 (C-1α Glc), 81.57, 81.52, 80.07, 80.02, 77.40, 75.97, 75.96, 75.89, 75.85, 75.29, 75.27, 75.18, 75.16, 75.10, 74.30, 74.28, 74.17, 73.13, 73.09, 72.48, 70.79, 70.14, 70.09, 69.95, 69.57, 69.24, 66.96 (C-5 Rha), 66.90 , 60.95, 60.92, 60.59, 60.48, 59.50, 59.41, 16.60 (C-6 Rha). ESI-HRMS m/z 835.2692 [M+Na]$^+$ ($C_{30}H_{52}O_{25}Na$ requires 835.2695).

RP-HPLC of 5mer-III (ELSD trace, Method C1, t_R = 17.5, 18.0 min)

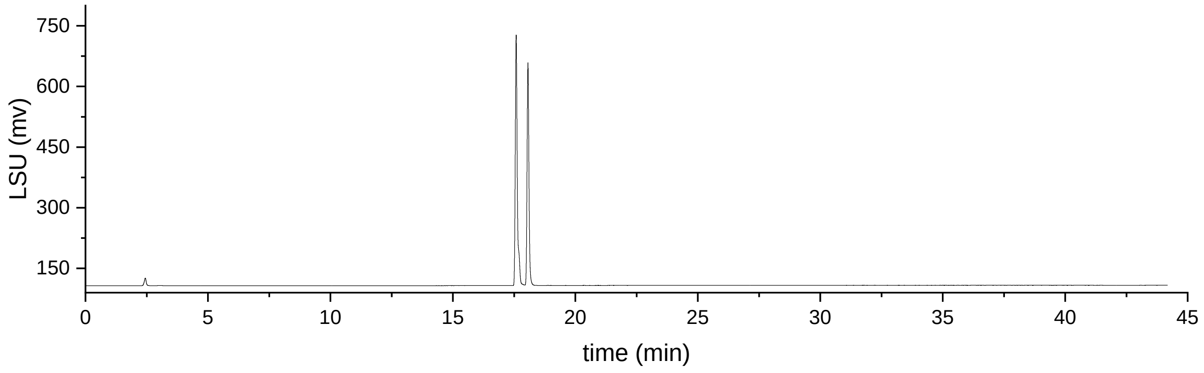

^{1}H NMR of 5mer-III (700 MHz, D_2O)

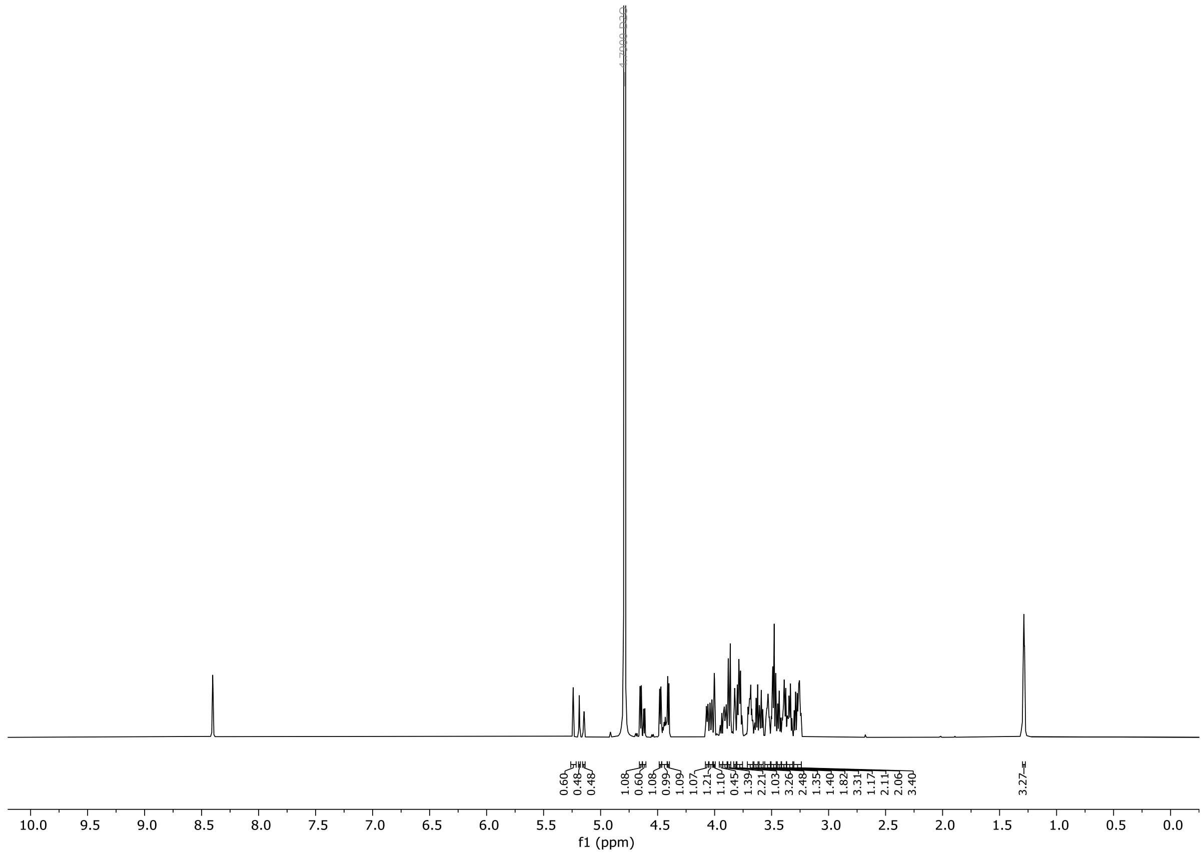

^{13}C NMR of 5mer-III (176 MHz, D_2O)

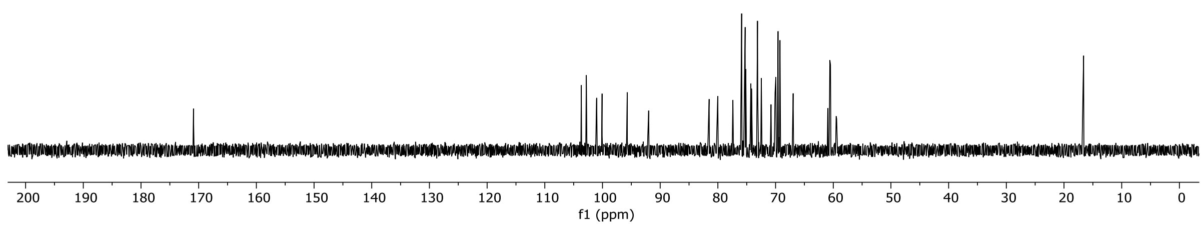

COSY NMR of 5mer-III (D_2O)

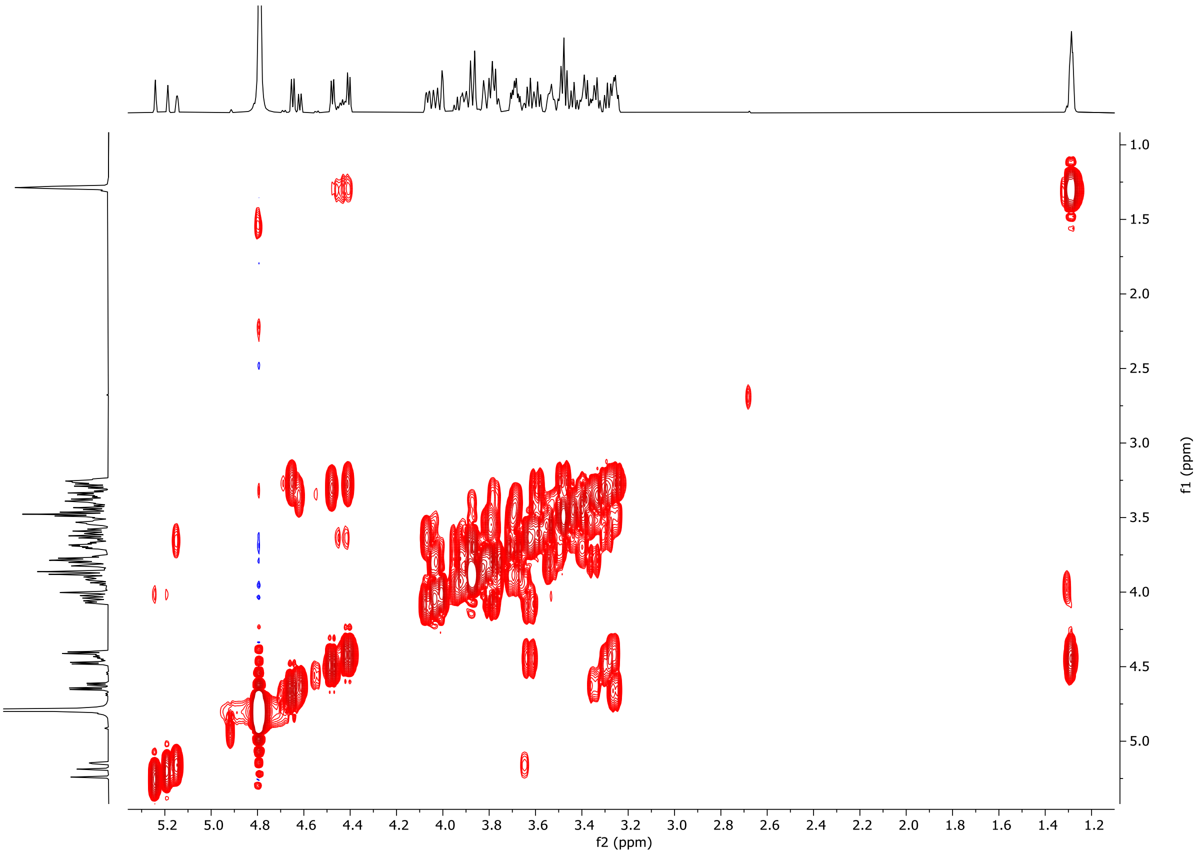

HSQC NMR of 5mer-III (D_2O)

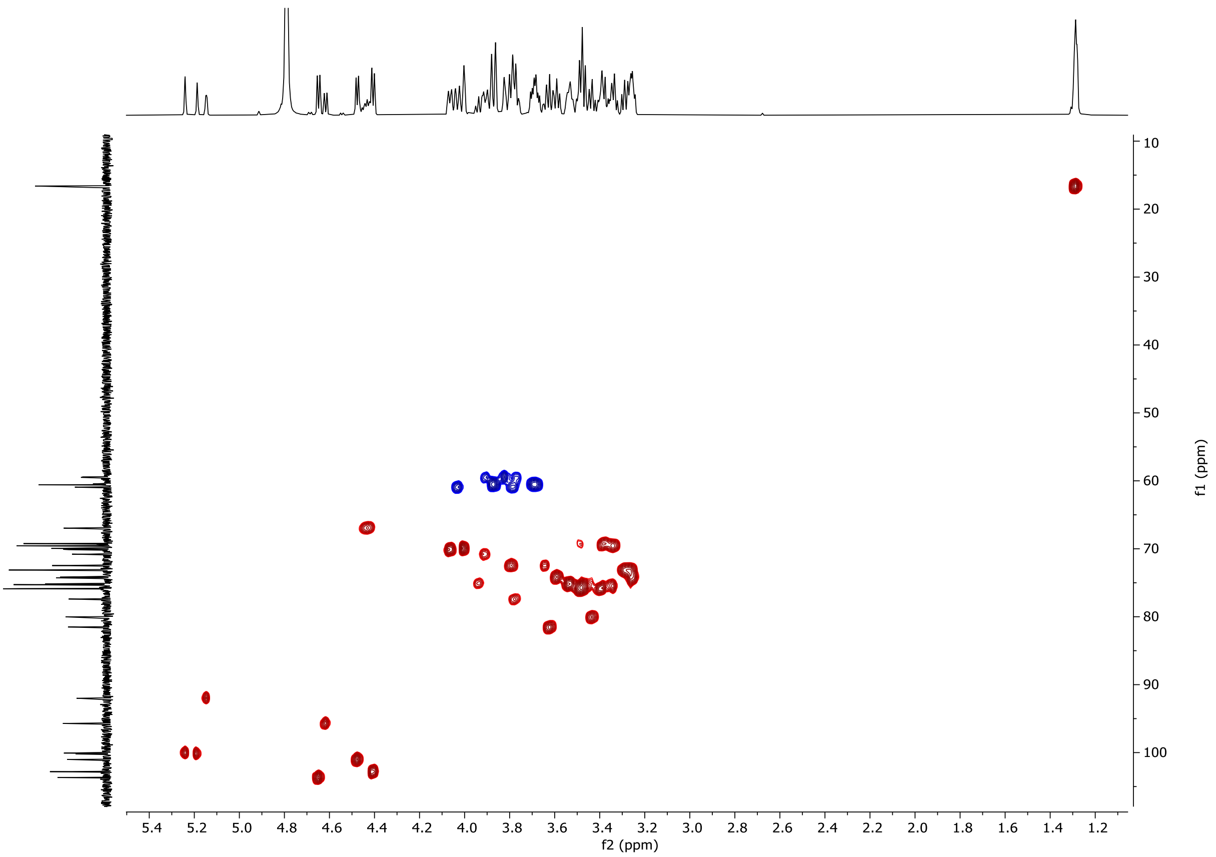

3.6.2 5mer-III-Closed

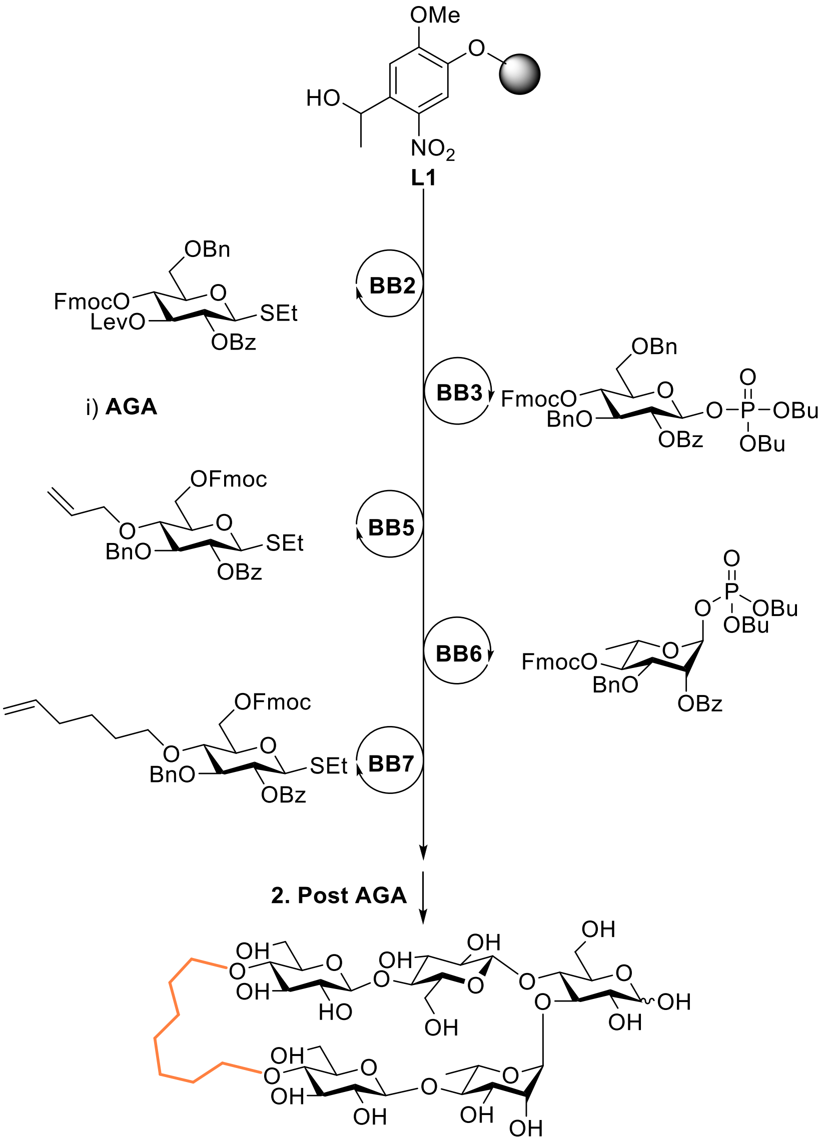

5mer-III-Closed

Step	BB	Modules	Notes
AGA	-	**A**	**L1** swelling
	**BB2**	**B, C1, D, E1**	**C1**: (**BB2**, -20 °C for 5 min, 0 °C for 20 min)
	**BB3**	**B, C3***, **D, E1**	**C3***: (**BB3**, -30 °C for 5 min, -10 °C for 40 min) *Double cycle
	**BB5**	**B, C1, D, E1**<br>**D, E2**	**C1**: (**BB5**, -20 °C for 5 min, 0 °C for 20 min)<br>-
	**BB6**	**B, C3, D, E1**	**C3**: (**BB6**, -30 °C for 5 min, -10 °C for 40 min)
	**BB7**	**B, C2***, **D, E1**	**C2***: (**BB7**, -25 °C for 25 min, -10 °C for 10 min) *Double cycle
Post-AGA	-	**F, G, H1, I1, J**	**G**: (15 h)<br>**I1**: (12 h)<br>**I**: (Method B2: 27.4 min)

Automated synthesis, global deprotection, and purification afforded **5mer-III-Closed** as a white solid (0.7 mg, 6% overall yield).

^{1}H NMR (700 MHz, D_2O) δ 5.21 (s, 0.6H, H-1β Rha), 5.16 (s, 0.4H, H-1α Rha), 5.15 (d, *J* = 3.7 Hz, 0.4H, H-1α Glc), 4.61 (t, *J* = 8.3 Hz, 1x H-1 Glc, 0.6H, H-1β Glc), 4.49 (m, *J* = 13.3, 7.5 Hz, 1x H-1 Glc, 1x H-5 Rha), 4.21 (d, *J* = 8.0 Hz, 1H, H-1 Glc), 4.06 – 4.01 (m, 2H), 4.00 (dd, *J* = 9.4, 3.3 Hz, 1H), 3.92 – 3.89 (m, 2H), 3.87 (d, *J* = 12.2 Hz, 1H), 3.83 (td, *J* = 8.4, 3.5 Hz, 2H), 3.77 (td, *J* = 10.0, 5.9 Hz, 5H), 3.66 – 3.62 (m, 3H), 3.56 (ddt, *J* = 26.1, 22.3, 8.8 Hz, 7H), 3.36 – 3.28 (m, 5H), 3.22 (td, *J* = 8.8, 4.1 Hz, 1H), 3.18 (dt, *J* = 14.4, 9.1 Hz, 2H), 1.64 – 1.56 (m, 3H), 1.51 (s, 1H), 1.43 – 1.34 (m, 4H), 1.30 (dd, *J* = 6.6, 3.9 Hz, 5H). ^{13}C NMR (176 MHz, D_2O) δ 104.46 (C-1 Glc), 103.15 (C-1 Glc), 100.98 (C-1 Glc), 100.48 (C-1 Rha), 95.65 (C-1β Glc), 91.99 (C-1α Glc), 83.52, 83.46, 79.92, 78.35, 78.01, 76.82, 75.70, 75.38, 75.20, 75.15, 75.13, 75.01, 75.01, 74.95, 74.95, 73.76, 73.57, 73.24, 72.88, 72.52, 72.32, 71.31, 70.80, 70.30, 70.25, 69.71, 66.83 (C-5 Rha), 60.96, 60.42, 60.20, 59.44, 59.40, 28.26, 27.12, 26.16, 24.17, 22.64, 16.52 (C-6 Rha). ESI-HRMS m/z 931.3652 $[M+Na]^+$ ($C_{37}H_{64}O_{25}Na$ requires 931.3634).

RP-HPLC of 5mer-III-Closed (ELSD trace, Method C1, t_R = 27.9, 28.0 min)

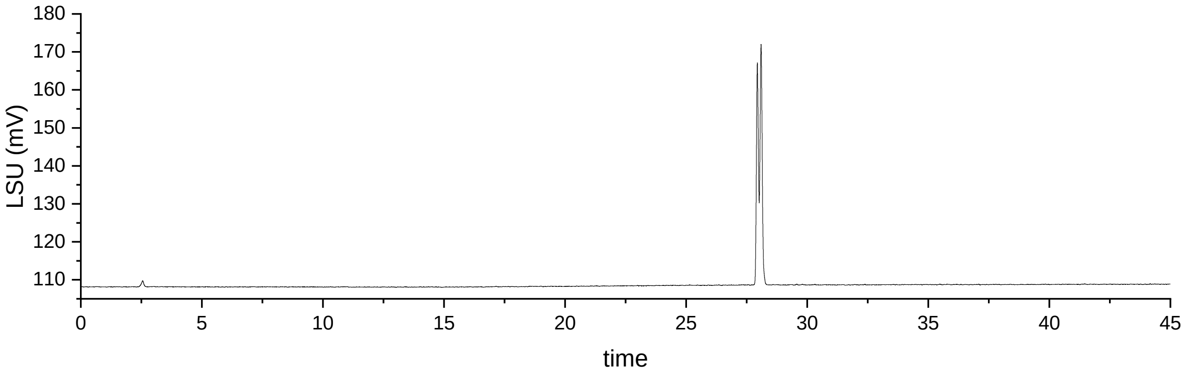

^{1}H NMR of 5mer-III-Closed (700 MHz, D_2O)

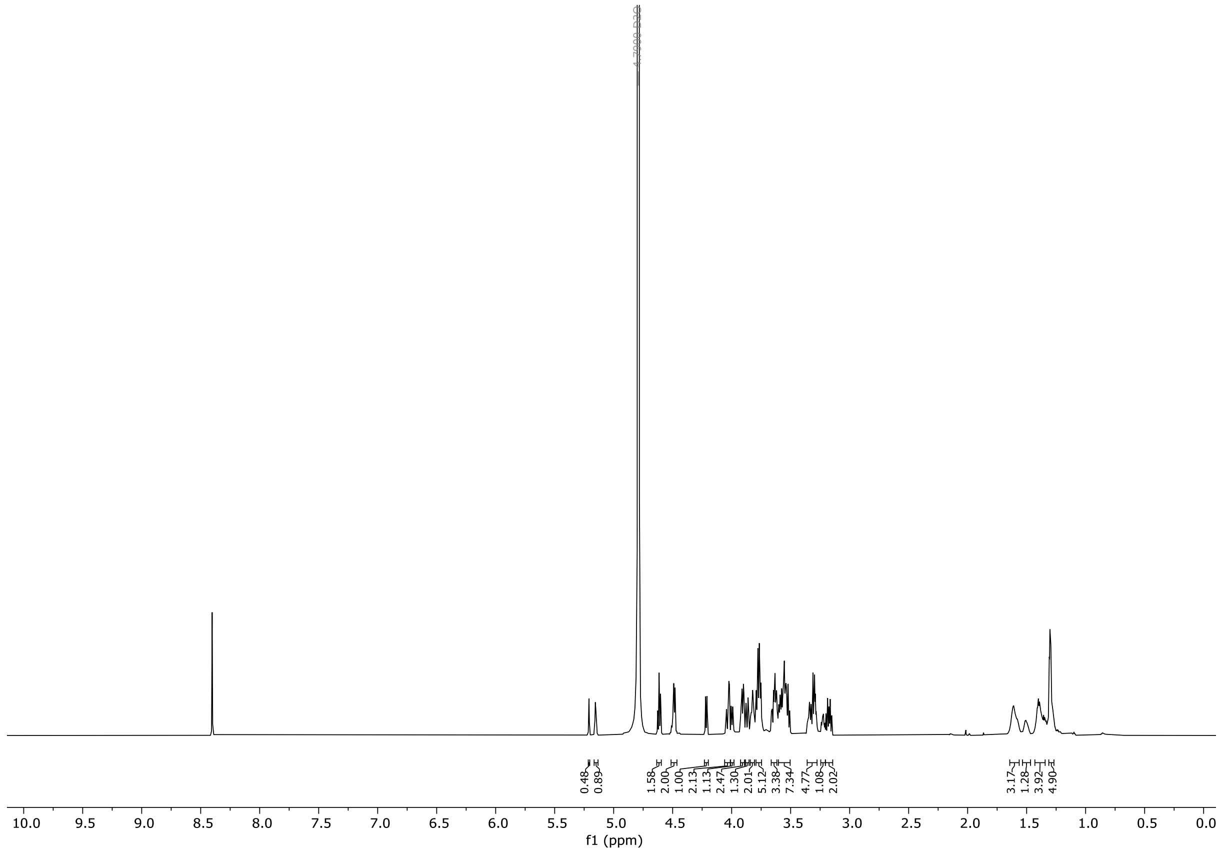

^{13}C NMR of 5mer-III-Closed (176 MHz, D_2O)

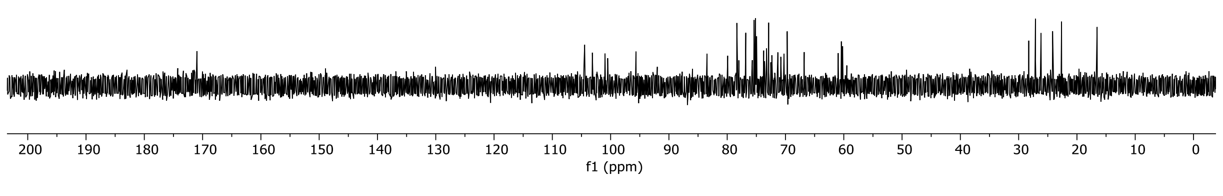

^{13}C-APT NMR of 5mer-III-Closed (176 MHz, D_2O)

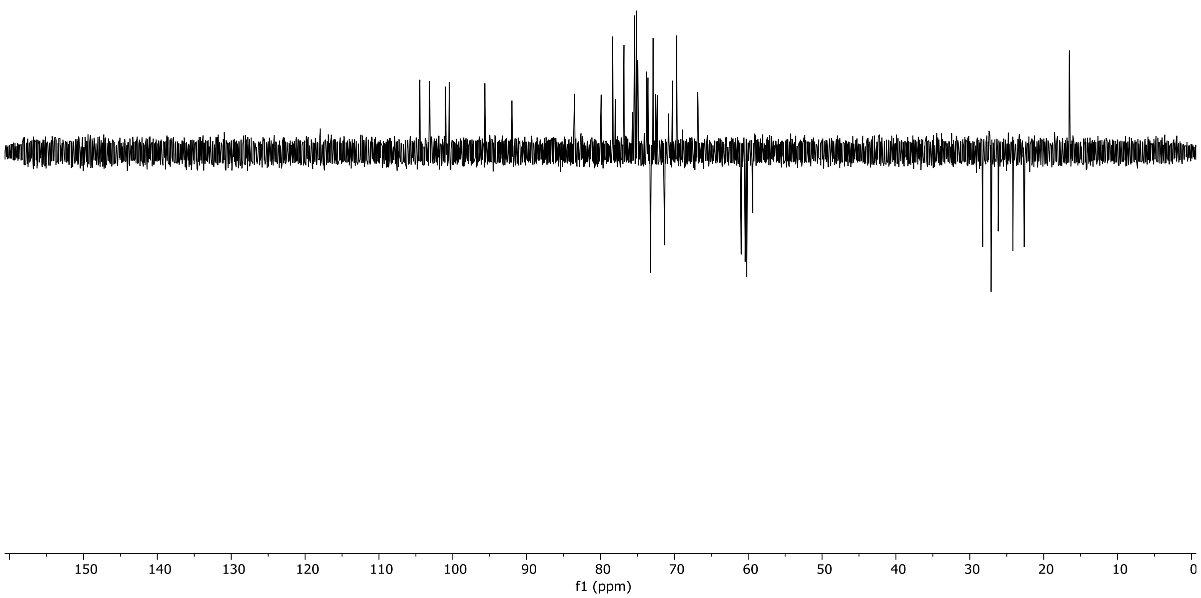

COSY NMR of 5mer-III-Closed (D_2O)

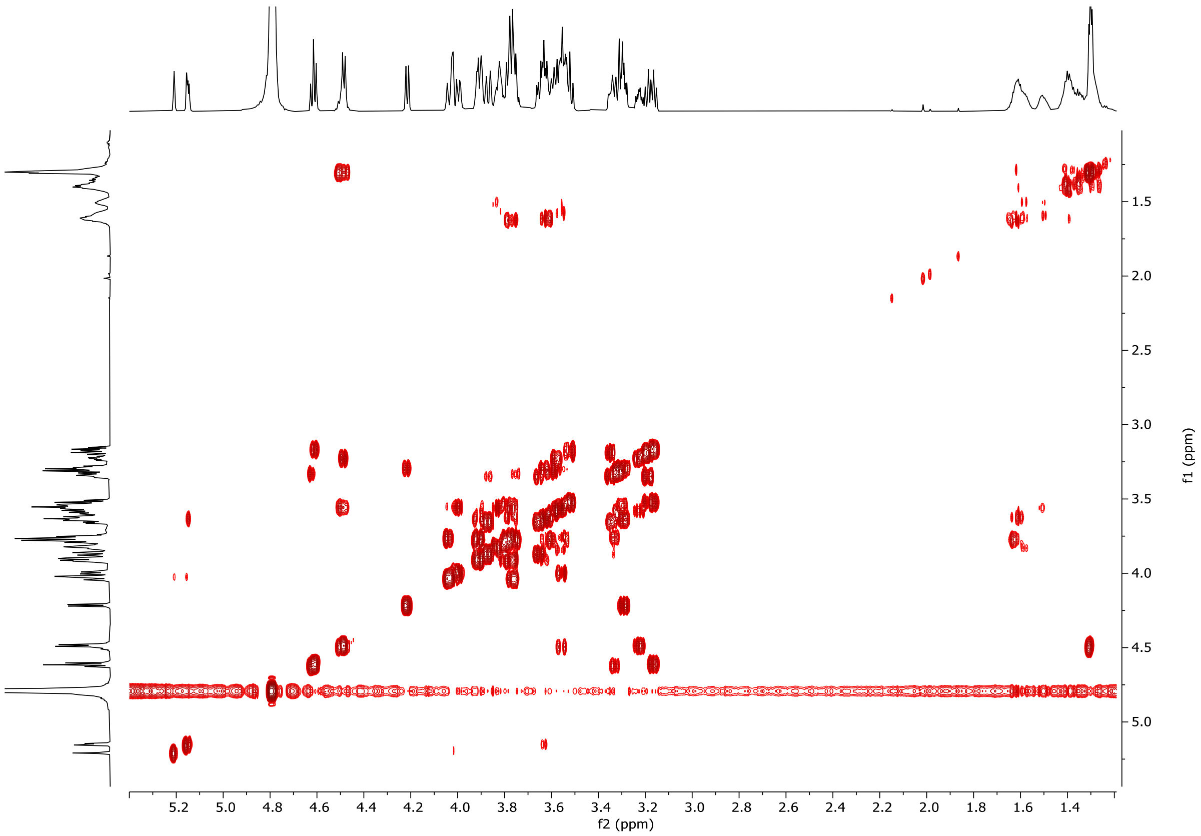

HSQC NMR of 5mer-III-Closed (D_2O)

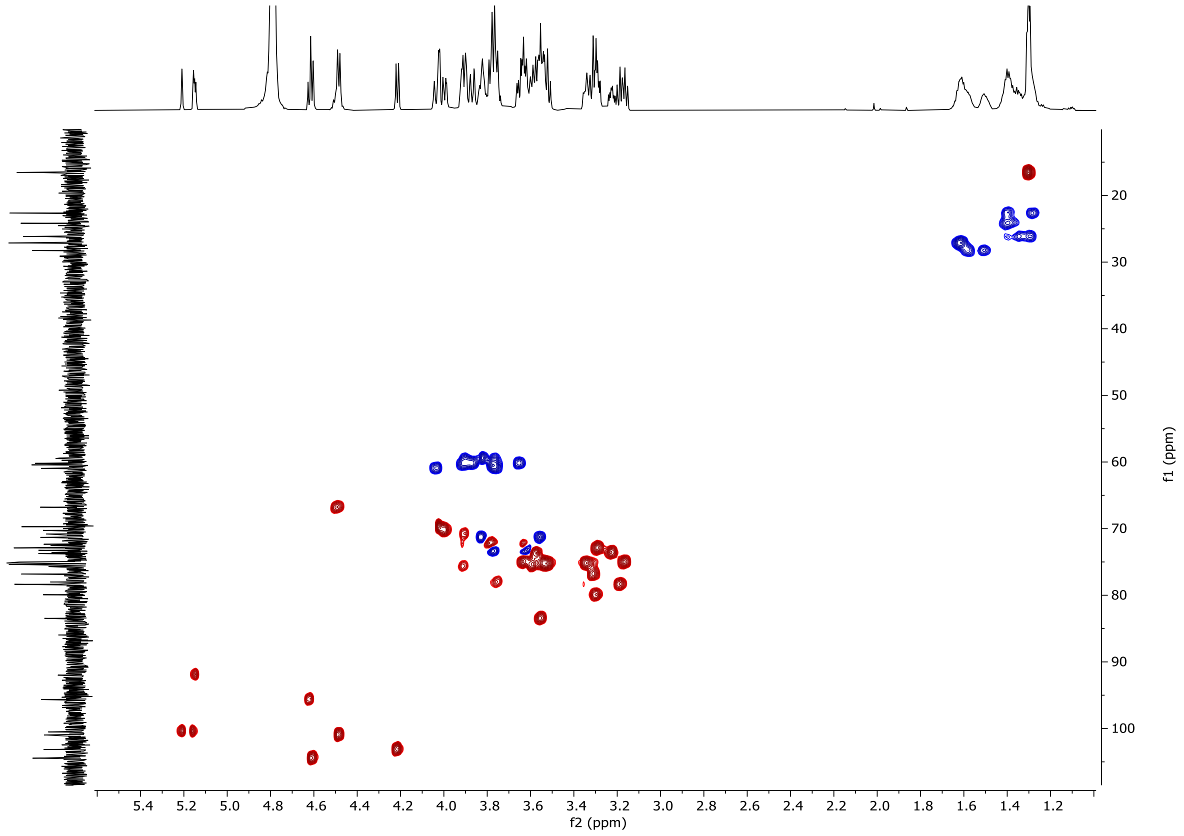

3.6.3 9mer-I-Linker

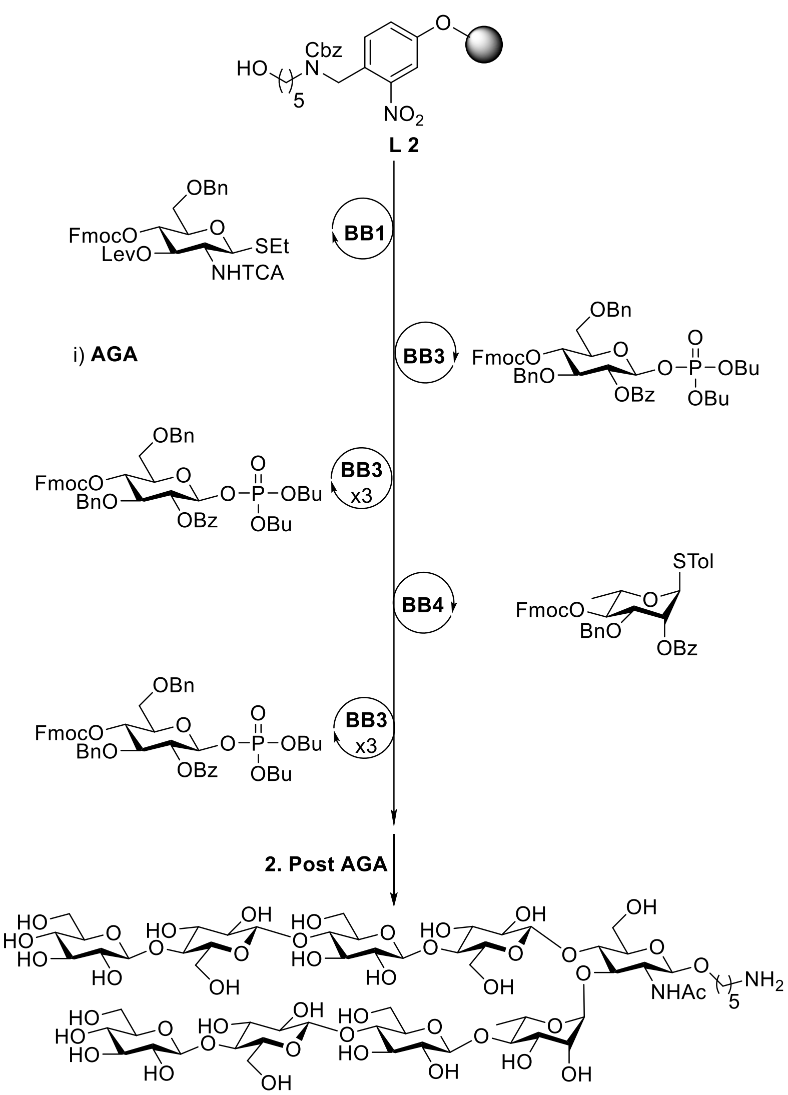

9mer-I-Linker

Step	BB	Modules	Notes
AGA	-	**A**	**L2** swelling
	**BB1**	**B**, **C1***, **D**, **E1**	**C1***: (**BB1**, -20 °C for 5 min, 0 °C for 40 min) *Double cycle
	**BB3**	**B**, **C3***, **D**, **E1**	**C3***: (**BB3**, -30 °C for 5 min, -10 °C for 40 min) *Double cycle
	(**BB3**)x3	(**B**, **C3**, **D**, **E1**)x3	**C3**: (**BB3**, -30 °C for 5 min, -10 °C for 40 min)
	-	**D**, **E2**	-
	**BB4**	**B**, **C1**, **D**, **E1**	**C1**: (**BB4**, -20 °C for 5 min, 0 °C for 20 min)
	(**BB3**)x3	(**B**, **C3**, **D**, **E1**)x3	**C3**: (**BB3**, -30 °C for 5 min, -10 °C for 40 min)
Post-AGA	-	**G**, **H1**, **I1**, **J**	**G**: (24 h)<br>**I1**: (12 h)<br>**J**: (Method D2: 39.4 min)

Automated synthesis, global deprotection, and purification afforded **9mer-I-Linker** as a white solid (5.7 mg, 30% overall yield).

^{1}H NMR (600 MHz, D_2O) δ 4.94 (d, *J* = 1.8 Hz, 1H, H-1 Rha), 4.68 (d, *J* = 7.9 Hz, 1H, H-1 Glc), 4.58 – 4.42 (m, 8H, 6x H-1 Glc, H-1 GlcNAc, H-5 Rha), 4.08 – 4.04 (m, 1H), 4.03 (dd, *J* = 9.4, 3.3 Hz, 1H), 3.99 – 3.93 (m, 5H), 3.93 – 3.87 (m, 4H), 3.82 (tdd, *J* = 11.0, 7.8, 3.2 Hz, 9H), 3.72 (dd, *J* = 12.4, 5.8 Hz, 2H), 3.68 – 3.53 (m, 17H), 3.52 – 3.45 (m, 5H), 3.43 – 3.38 (m, 2H), 3.37 – 3.28 (m, 7H), 3.00 – 2.94 (m, 2H), 2.04 (s, 3H), 1.66 (p, *J* = 7.7 Hz, 2H), 1.59 (p, *J* = 6.5 Hz, 2H), 1.43 – 1.35 (m, 2H), 1.31 (d, *J* = 6.3 Hz, 3H, CH_3-6 Rha). ^{13}C NMR (151 MHz, D_2O) δ 103.59 (C-1 Glc), 102.84 (C-1 Glc), 102.60 (C-1 Glc), 102.55 (C-1 Glc), 102.43 (C-1 Glc), 102.27 (C-1 Glc), 100.96 (C-1 Glc), 100.52 (C-1 GlcNAc), 100.10 (C-1 Rha) 78.71, 78.46, 75.93, 75.50, 75.41, 75.16, 74.69, 74.47, 74.09, 74.00, 73.10, 73.01, 72.83, 70.15, 69.39, 67.09 (C-5 Rha), 60.51, 59.90, 39.26, 28.05, 26.35, 22.10, 22.05 (CH_3 Ac GlcNAc), 16.77 (C-6 Rha). ESI-HRMS m/z 1587.617 $[M+H]^+$ ($C_{61}H_{107}N_2O_{45}$ requires 1587.615).

RP-HPLC of 9mer-I-Linker (ELSD trace, Method C1, t_R = 23.5 min)

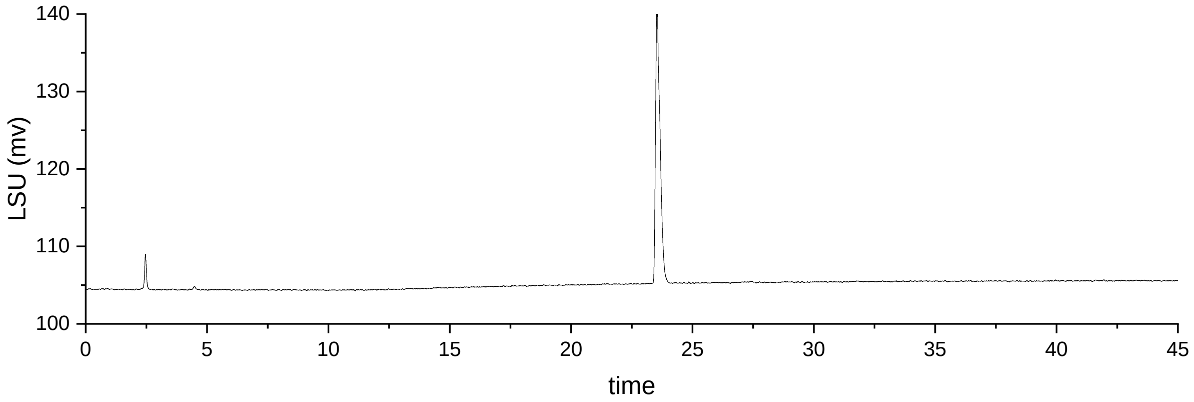

^{1}H NMR of 9mer-I-Linker (600 MHz, D_2O)

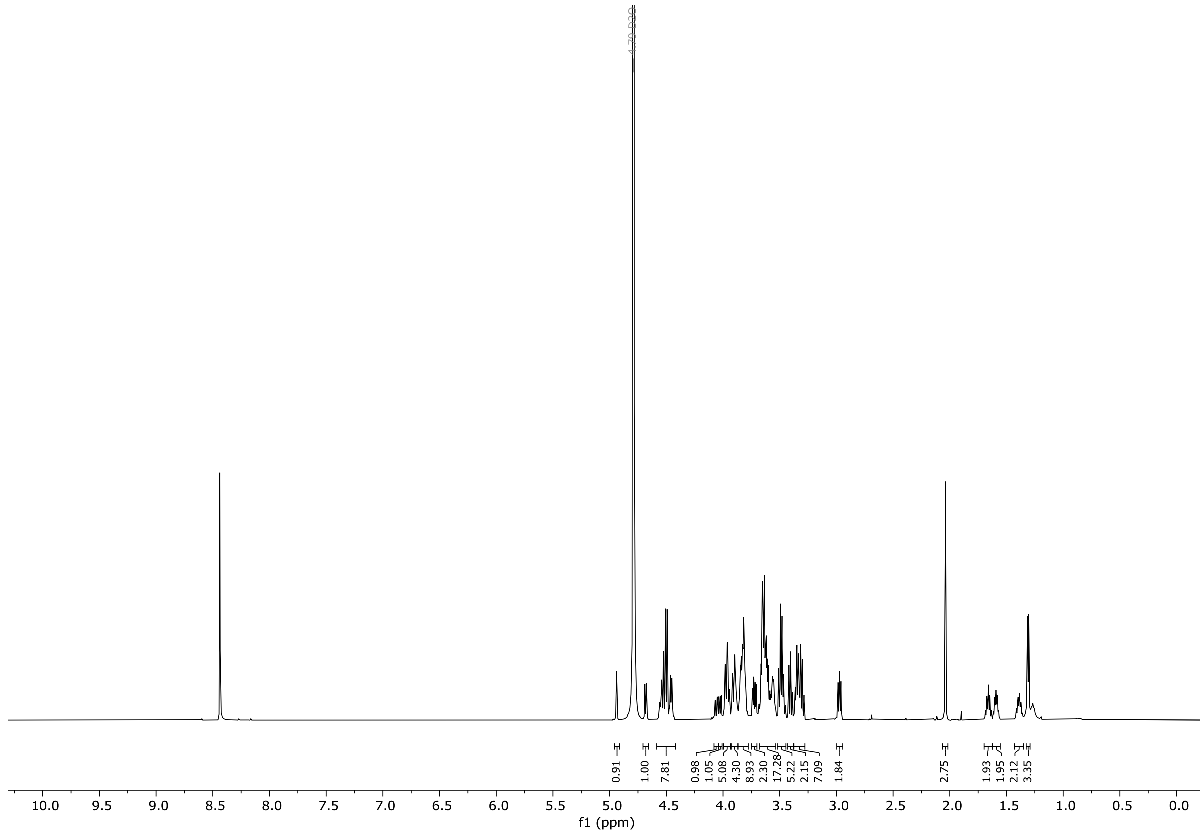

^{13}C NMR of 9mer-I-Linker (151 MHz, D_2O)

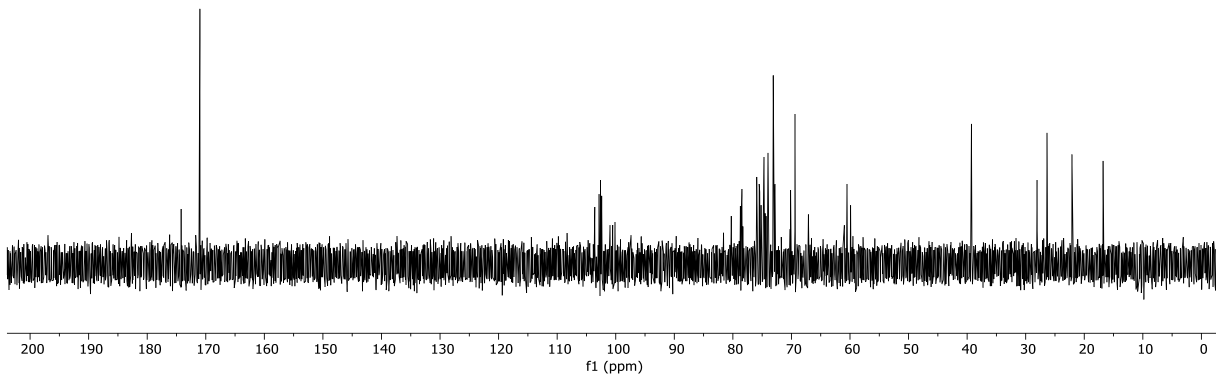

COSY NMR of 9mer-I-Linker (D_2O)

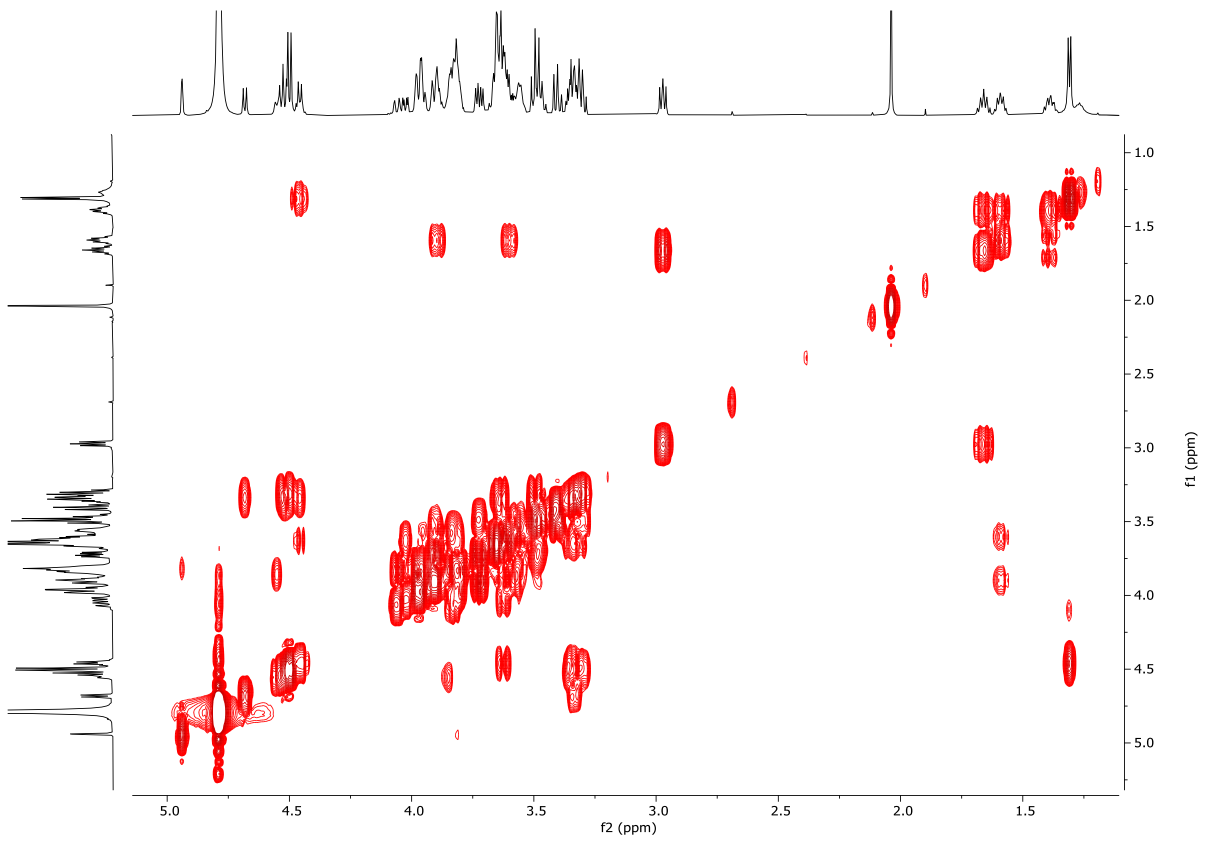

HSQC NMR of 9mer-I-Linker (D_2O)

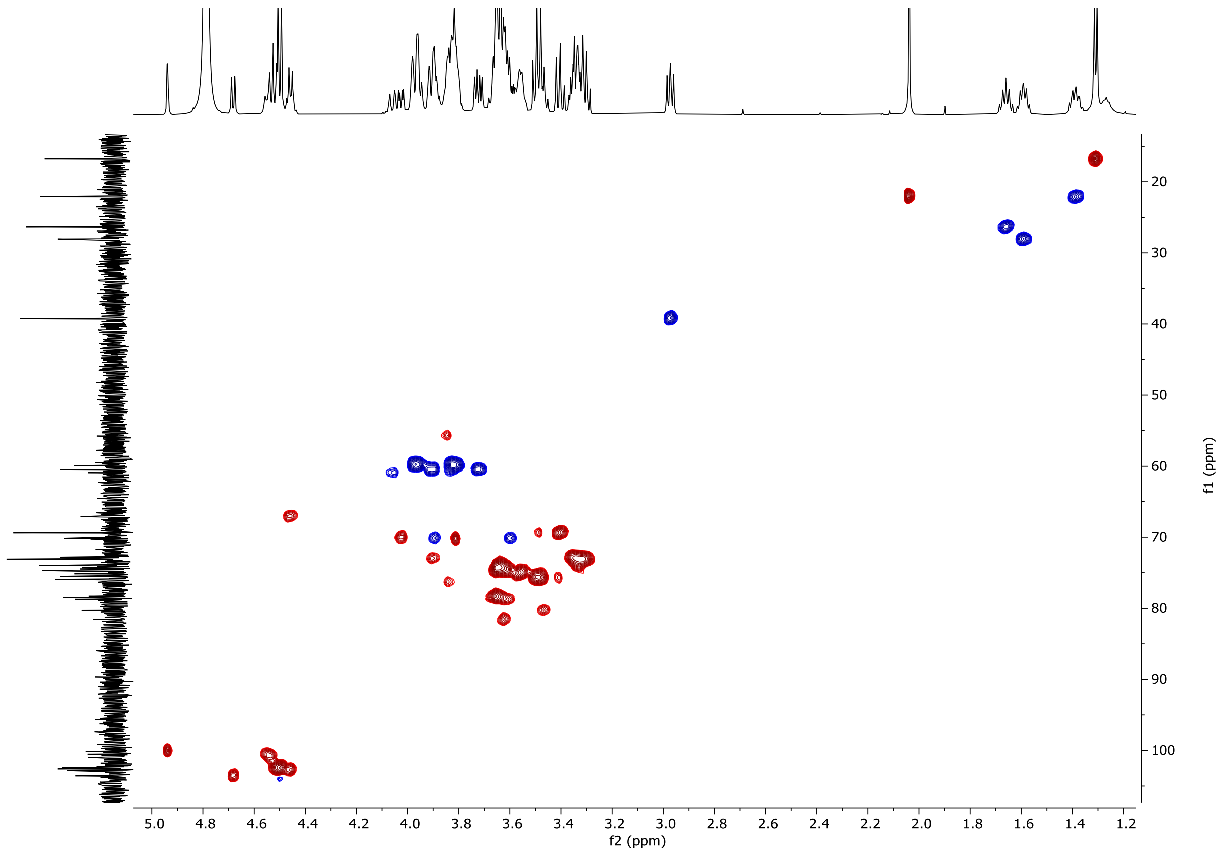

3.6.4 9mer-III

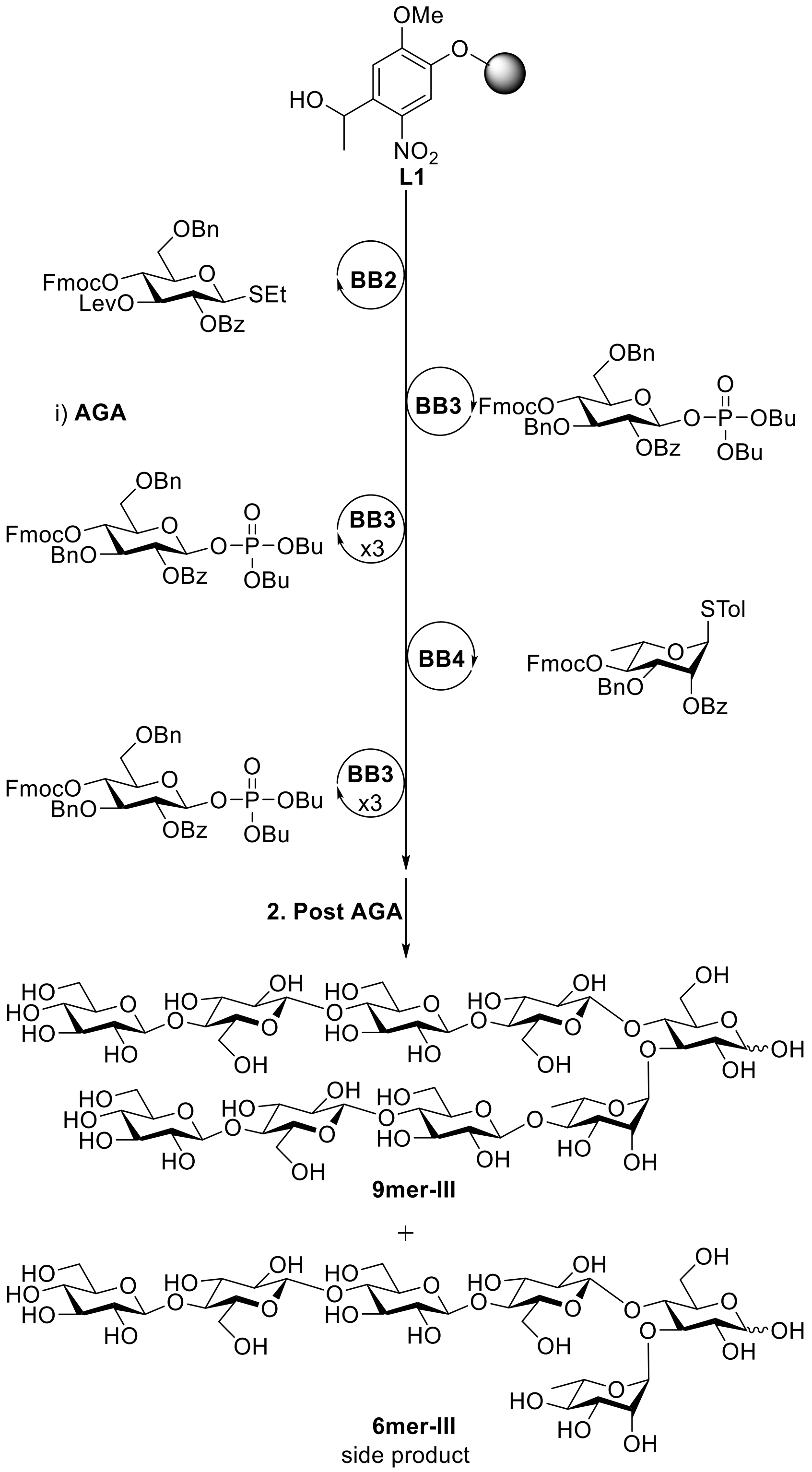
OMe
HO
NO_2
L1
OBn
FmocO
LevO
O
SEt
OBz
BB2
BB3
FmocO
BnO
OBz
O-P-OBu
OBu
i) AGA
BB3
x3
BB4
STol
BnO
BB3
x3
2. Post AGA
HO
OH
9mer-III
+
6mer-III
side product

Step	BB	Modules	Notes
AGA	-	**A**	**L1** swelling
	**BB2**	**B**, **C1**, **D**, **E1**	**C1**: (**BB2**, -20 °C for 5 min, 0 °C for 20 min)
	**BB3**	**B**, **C3***, **D**, **E1**	**C3***: (**BB3**, -30 °C for 5 min, -10 °C for 40 min) *Double cycle
	(**BB3**)x3	(**B**, **C3**, **D**, **E1**)x3	**C3**: (**BB3**, -30 °C for 5 min, -10 °C for 40 min)
	-	**D**, **E2**	-
	**BB4**	**B**, **C1**, **D**, **E1**	**C1**: (**BB4**, -20 °C for 5 min, 0 °C for 20 min)
	(**BB3**)x3	(**B**, **C3**, **D**, **E1**)x3	**C3**: (**BB3**, -30 °C for 5 min, -10 °C for 40 min)
Post-AGA	-	**G**, **H1**, **I2**, **J**	**G**: (24 h)<br>**I2**: (6 h)<br>**J**: (Method C2: 24.6 min)

Automated synthesis, global deprotection, and purification afforded **9mer-III** as a white solid (4.01 mg, 22% overall yield) and **6mer-III** side product (1.8 mg, 15% overall yield) due to incomplete glycosylation between Rha (solid-bound acceptor) and Glc **(BB3)**.

RP-HPLC of crude 9mer-III (ELSD trace, Method C1)

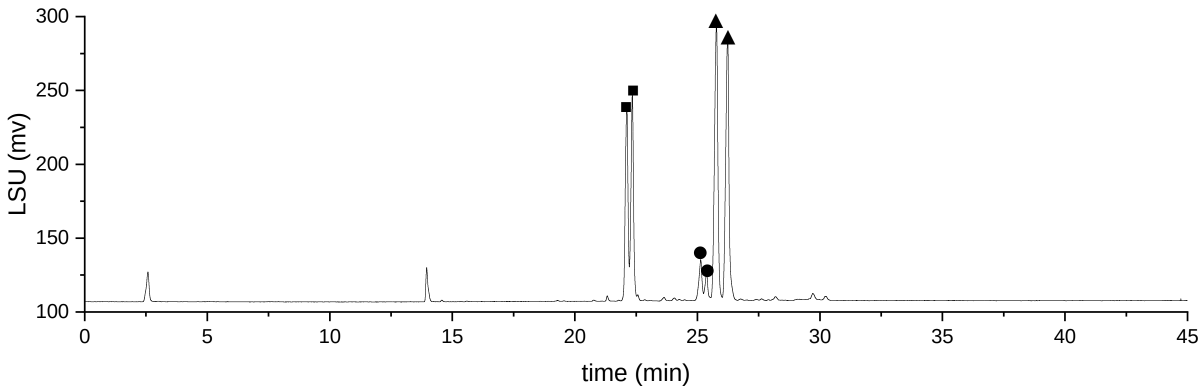

▲**9mer-III** (α and β anomers, m/z 1461 [M+H]$^+$). ■ 6mer-III side product (α and β anomers, m/z 975 [M+H]$^+$). ● 5mer side product due to incomplete deprotection of the Lev group (α and β anomers, m/z 835 [M+Na]$^+$).

Characterization of 9mer-III

^{1}H NMR (600 MHz, D_2O) δ 5.24 (d, *J* = 1.7 Hz, 0.6H, H-1β Rha), 5.19 (d, *J* = 1.7 Hz, 0.4H, H-1α Rha), 5.15 (d, *J* = 3.7 Hz, 0.4H, H-1α Glc), 4.66 (d, *J* = 7.9 Hz, 1H, H-1 Glc), 4.62 (d, *J* = 8.0 Hz, 0.6H, H-1β Glc), 4.50 – 4.42 (m, 7H, 6x H-1 Glc, H-5 Rha), 4.08 – 4.02 (m, 2H), 4.01 – 3.98 (m, 1H), 3.96 – 3.91 (m, 5H), 3.89 – 3.87 (m, 1H), 3.86 (t, *J* = 2.8 Hz, 1H), 3.83 – 3.76 (m, 8H), 3.69 (dd, *J* = 12.4, 5.8 Hz, 2H), 3.60 (dtd, *J* = 17.4, 9.3, 3.9 Hz, 14H), 3.53 (ddq, *J* = 6.8, 4.6, 2.7 Hz, 3H), 3.48 – 3.43 (m, 5H), 3.39 – 3.35 (m, 2H), 3.34 – 3.28 (m, 6H), 3.28 – 3.25 (m, 1H), 1.29 (dd, *J* = 6.3, 3.5 Hz, 3H, CH_3-6 Rha). ^{13}C NMR (151 MHz, D_2O) δ 103.60 (C-1 Glc), 102.77 (C-1 Glc), 102.56 (C-1 Glc), 102.51 (C-1 Glc), 102.37 (C-1 Glc), 100.99 (C-1 Glc), 100.1 (C-1α Rha), 99.9 (C-1β Rha), 95.72 (C-1β Glc), 91.9 (C-1α Glc), 81.81, 78.65, 78.46, 78.39, 78.21, 75.89, 75.44, 75.36, 75.13, 74.74, 74.64, 74.45, 74.22, 74.03, 73.93, 73.05, 72.98, 72.79, 72.43, 70.03, 69.88, 69.34, 66.8 (C-5 Rha), 60.46, 59.85, 16.56 (C-6 Rha). ESI-HRMS m/z 1461.499 $[M+H]^+$ ($C_{54}H_{93}O_{45}$ requires 1461.499).

RP-HPLC of 9mer-III (ELSD trace, Method C1, t_R = 25.3, 25.7 min)

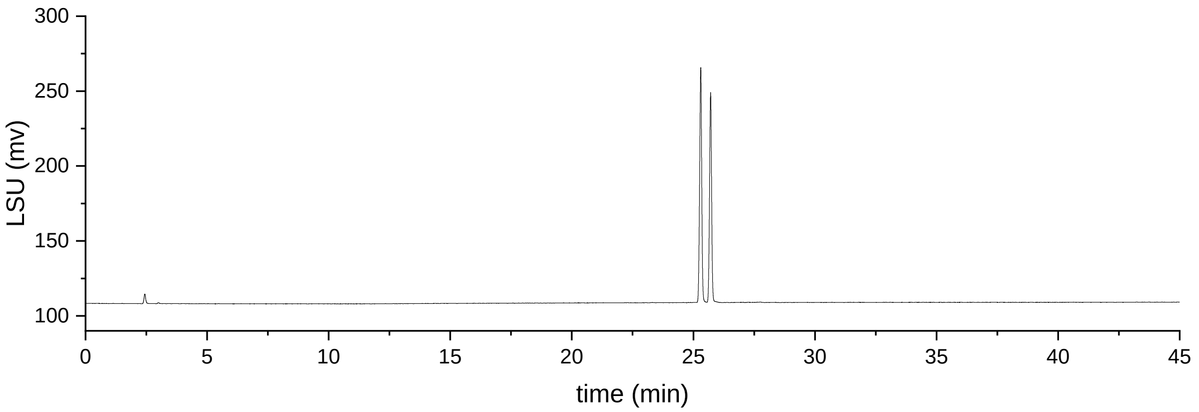

^{1}H NMR of 9mer-III (600 MHz, D_2O)

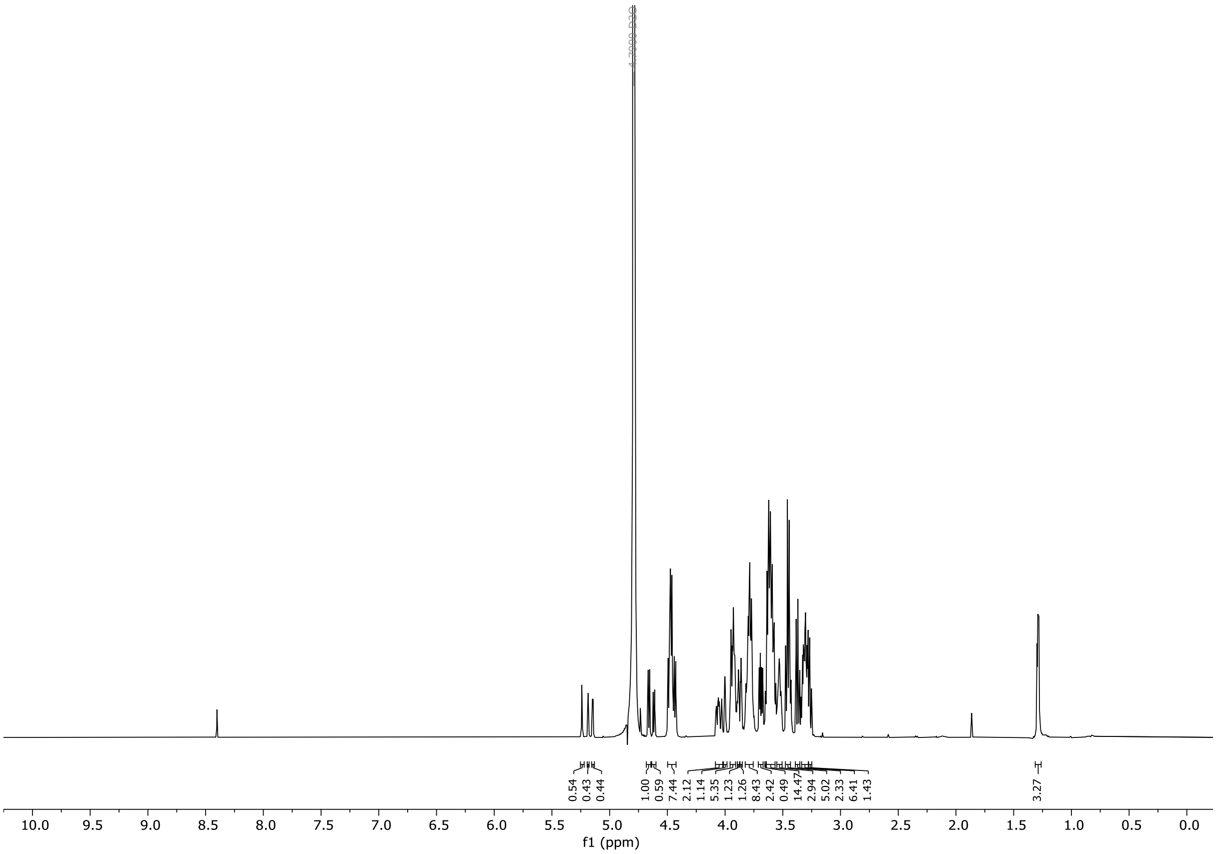

^{13}C NMR of 9mer-III (151 MHz, D_2O)

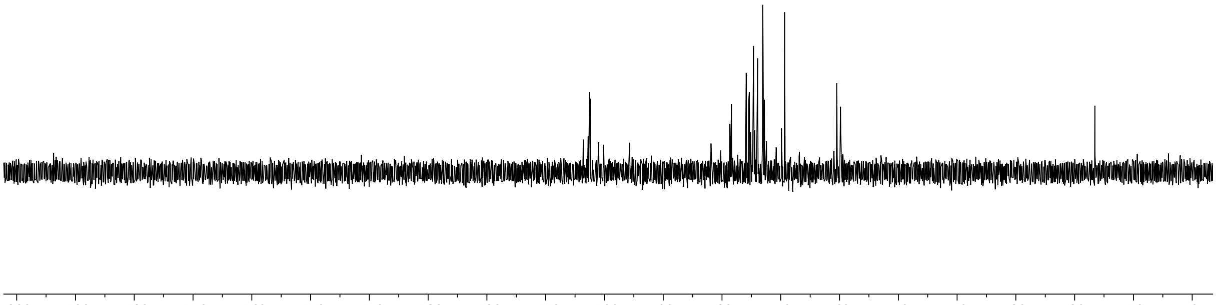

COSY NMR of 9mer-III (D_2O)

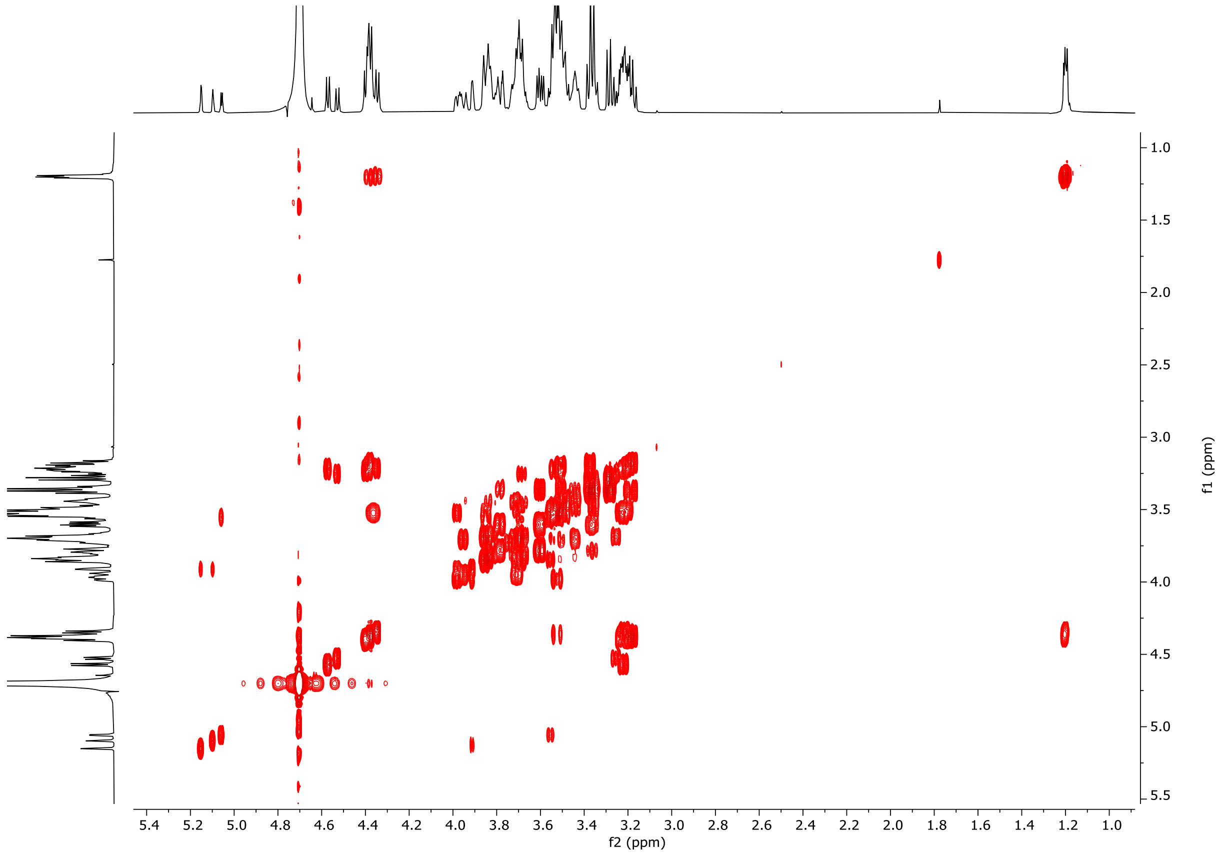

HSQC NMR of 9mer-III (D_2O)

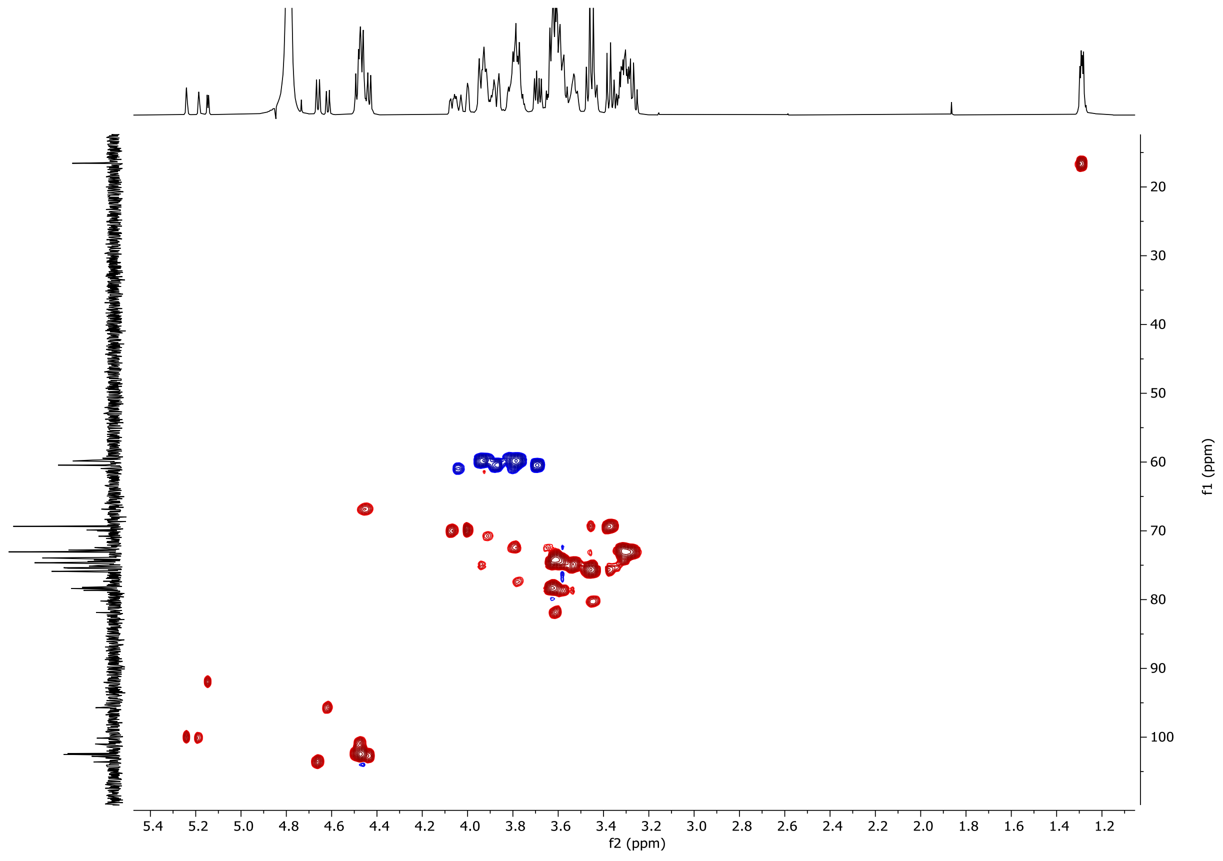

Characterization of 6mer-III

^{1}H NMR (400 MHz, D_2O) δ 5.20 (d, *J* = 1.7 Hz, 0.6H, H-1β Rha), 5.15 (d, *J* = 1.7 Hz, 0.4H, H-1α Rha), 5.13 (d, *J* = 3.7 Hz, 0.4H, H-1α Glc), 4.60 (d, *J* = 8.0 Hz, 0.6H, H-1β Glc), 4.49 – 4.43 (m, 4H, 4x H-1 Glc), 4.42 – 4.32 (m, 1H, H-5 Rha), 3.98 (dd, *J* = 3.4, 1.7 Hz, 1H), 3.93 (dd, *J* = 5.3, 2.8 Hz, 1H), 3.90 (dq, *J* = 4.6, 2.7 Hz, 2H), 3.87 (d, *J* = 2.7 Hz, 1H), 3.85 – 3.81 (m, 2H), 3.81 – 3.72 (m, 6H), 3.70 – 3.66 (m, 1H), 3.65 – 3.62 (m, 1H), 3.61 – 3.50 (m, 9H), 3.45 – 3.39 (m, 2H), 3.37 (d, *J* = 2.6 Hz, 1H), 3.36 – 3.33 (m, 1H), 3.33 – 3.26 (m, 3H), 3.26 – 3.18 (m, 2H), 1.20 (dd, *J* = 6.3, 1.6 Hz, 3H, CH_3-6 Rha). ^{13}C NMR (101 MHz, D_2O) δ102.45 (C-1 Glc), 102.24 (C-1 Glc), 101.39 (C-1 Glc), 100.67 (C-1α Rha), 100.54 (C-1β Rha), 95.66 (C-1β Glc), 91.93 (C-1α Glc), 81.58, 78.47, 78.19, 78.04, 75.86, 75.33, 75.19, 74.96, 74.70, 73.89, 73.02, 72.81, 71.85, 70.78, 69.99, 69.31, 68.38 (C-5 Rha), 60.43, 60.21, 59.73, 16.39 (CH_3-6 Rha). ESI-HRMS m/z 975.3407 $[M+H]^+$ ($C_{36}H_{63}O_{30}$ requires 975.3404).

RP-HPLC of 6mer-III (ELSD trace, Method C1, t_R = 21.8, 22.0 min)

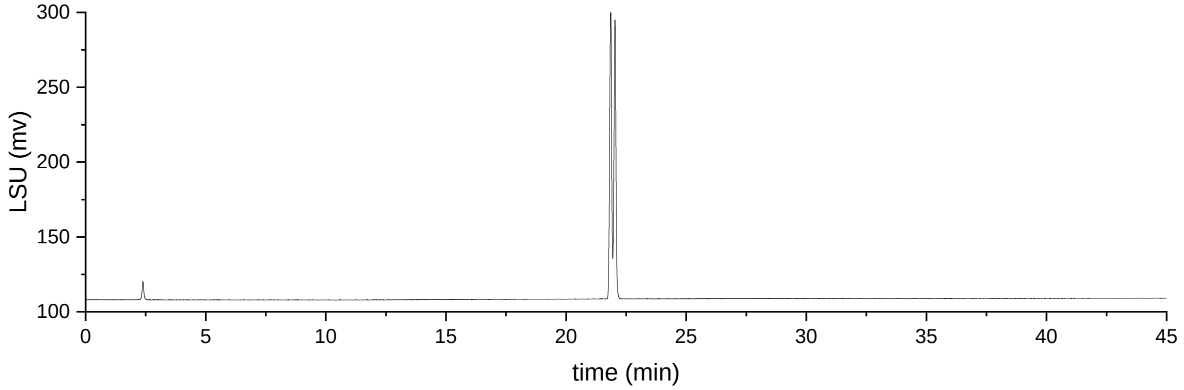

^{1}H NMR of 6mer-III (400 MHz, D_2O)

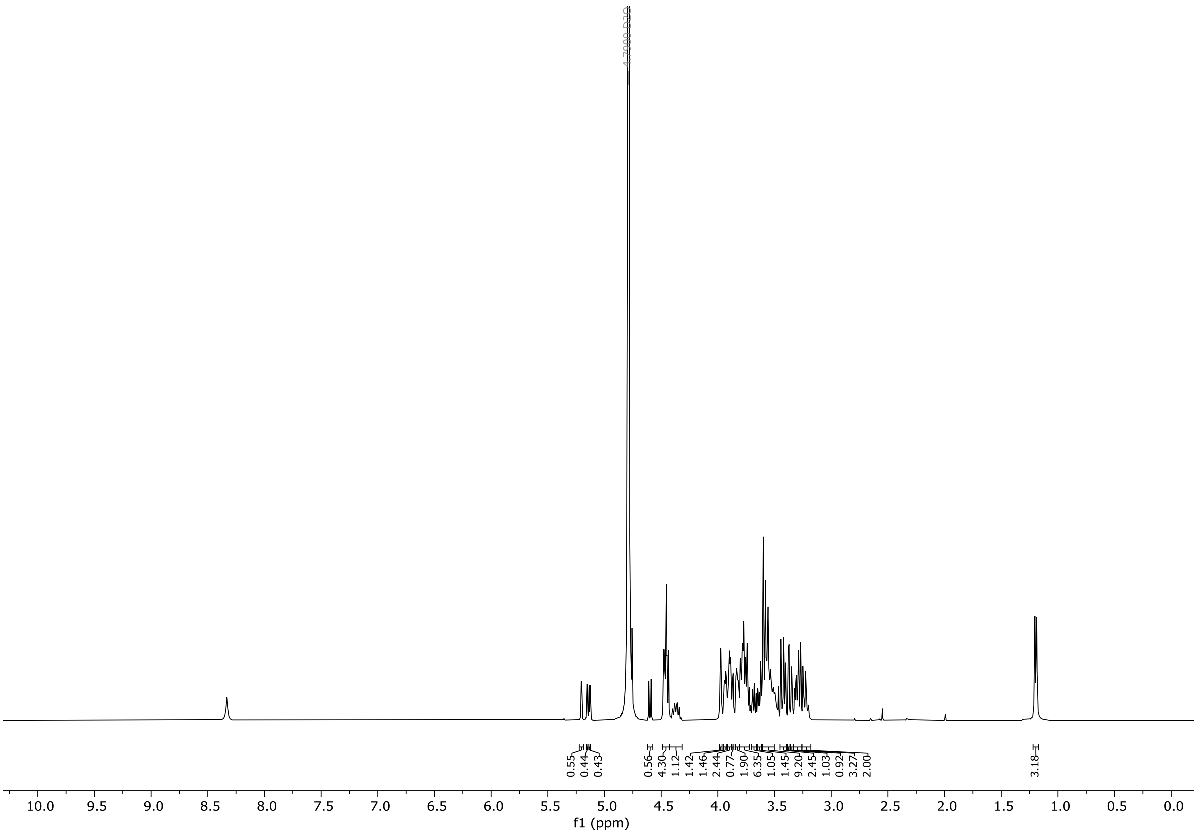

^{13}C NMR of 6mer-III (101 MHz, D_2O)

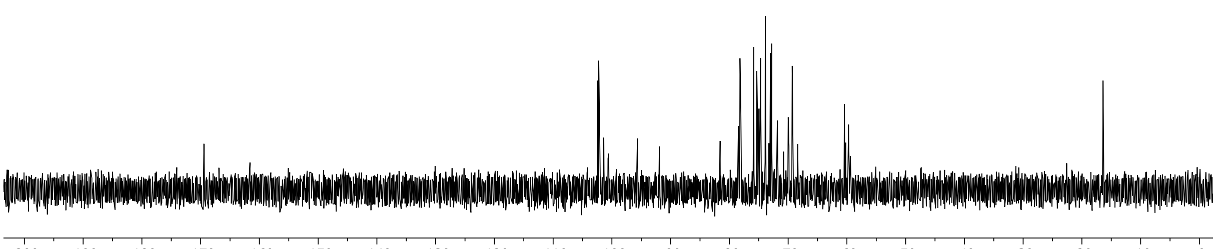

COSY NMR of 6mer-III (D_2O)

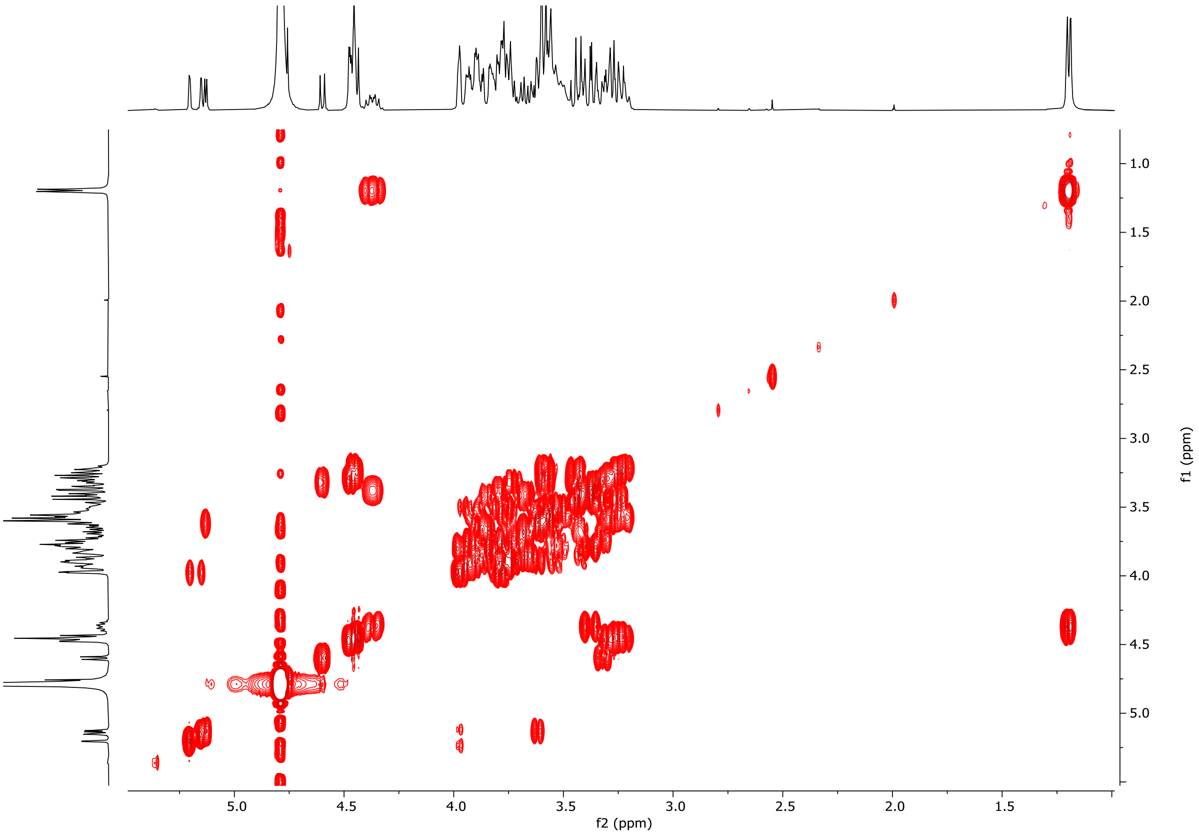

HSQC NMR of 6mer-III (D_2O)

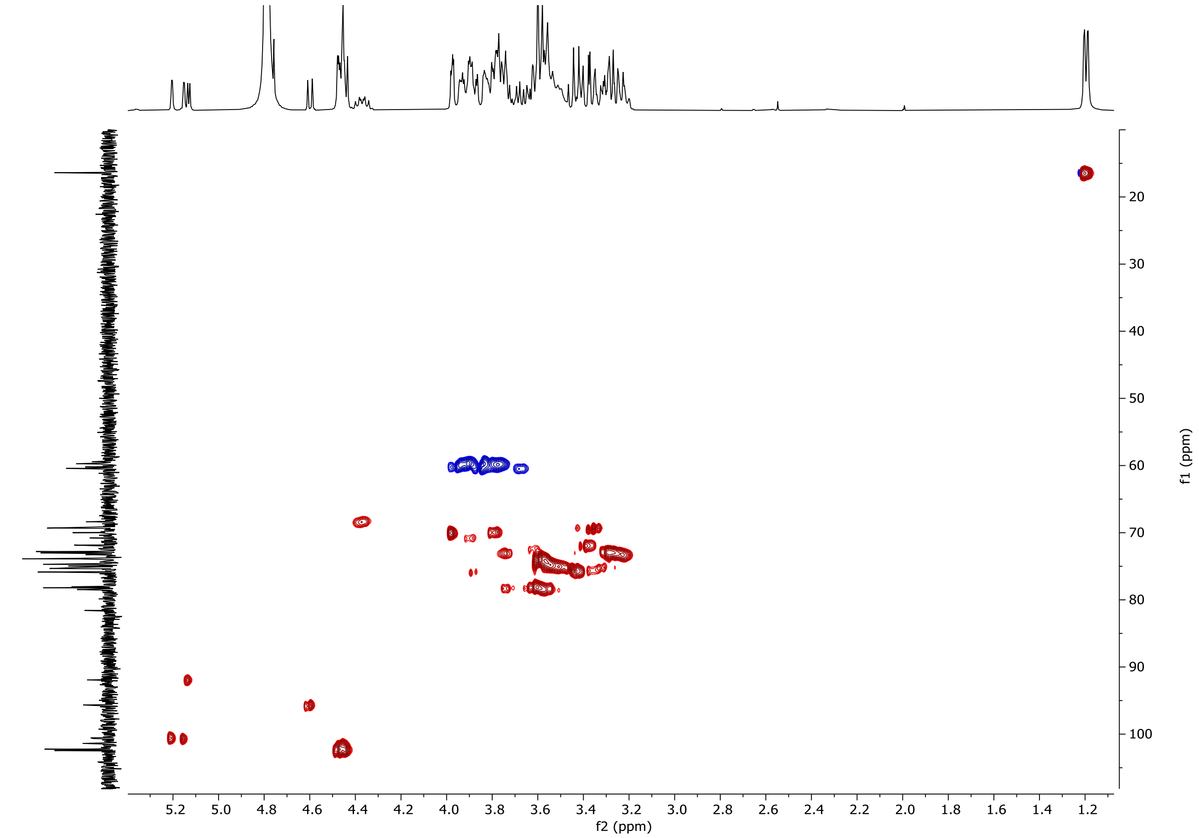

3.6.5 9mer-IV

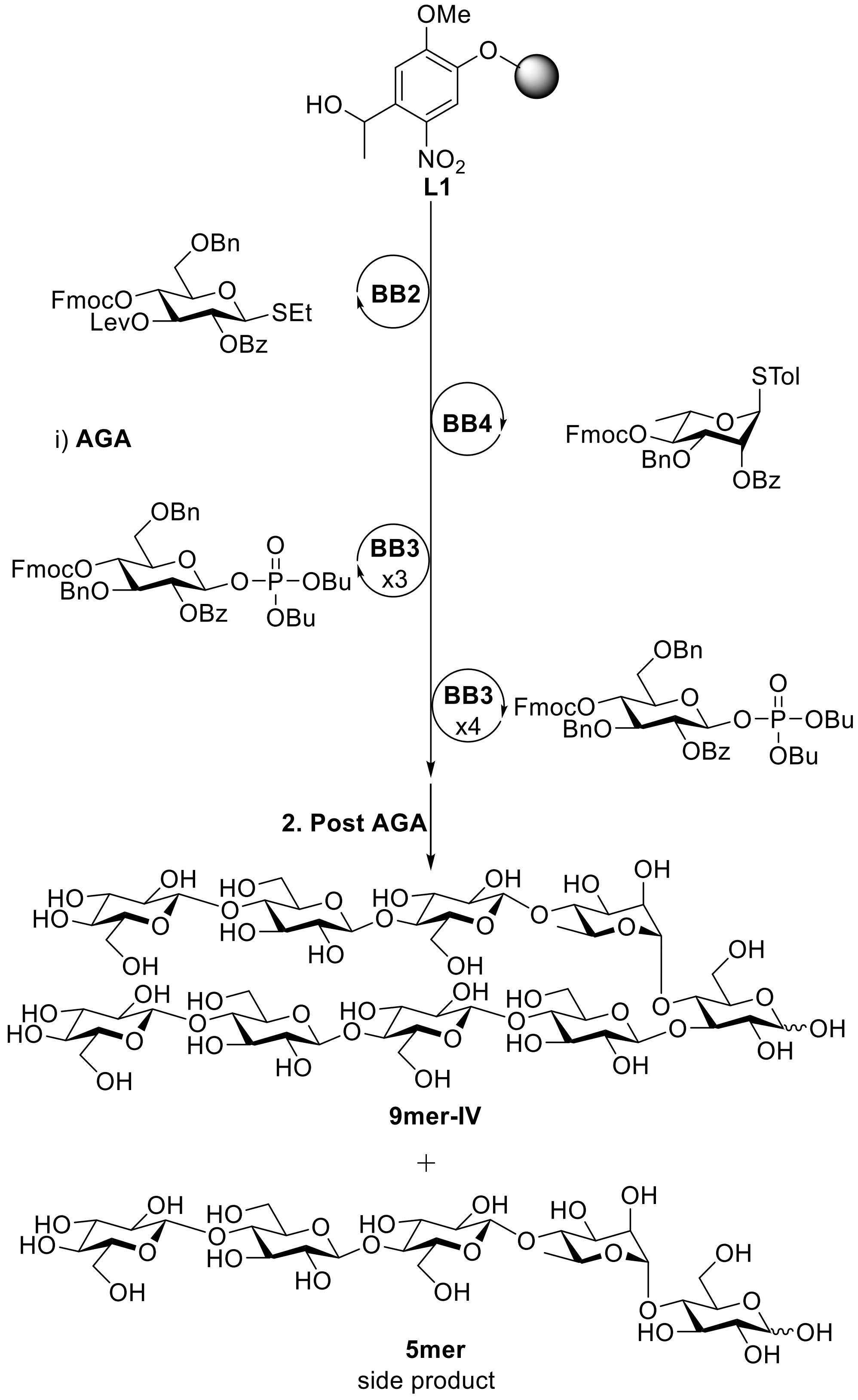
L1
BB2
BB4
i) AGA
BB3
x3
BB3
x4
2. Post AGA
9mer-IV
+
5mer
side product

Step	BB	Modules	Notes
AGA	-	**A**	**L1** swelling
	**BB2**	**B**, **C1**, **D**, **E1**	**C1**: (**BB2**, -20 °C for 5 min, 0 °C for 20 min)
	**BB4**	**B**, **C1**, **D**, **E1**	**C1**: (**BB4**, -20 °C for 5 min, 0 °C for 20 min)
	(**BB3**)x3	(**B**, **C3**, **D**, **E1**)x3 **D,E2**	**C3**: (**BB3**, -30 °C for 5 min, -10 °C for 40 min)
	(**BB3**)x4	(**B**, **C3**, **D**, **E1**)x4	**C3**: (**BB3**, -30 °C for 5 min, -10 °C for 40 min)
Post-AGA	-	**G**, **H1**, **I2**, **J**	**G**: (18 h)<br>**I2**: (6 h)<br>**J**: (Method B2: 15.1 min)

Automated synthesis, global deprotection, and purification afforded **9mer-IV** as a white solid (3.7 mg, 20% yield). A **5mer** side product was also formed (4.2 mg, 40% yield) due to incomplete glycosylation of the 1,3-Glc glycosidic bond.

RP-HPLC of crude 9mer-IV (ELSD trace, Method C1)

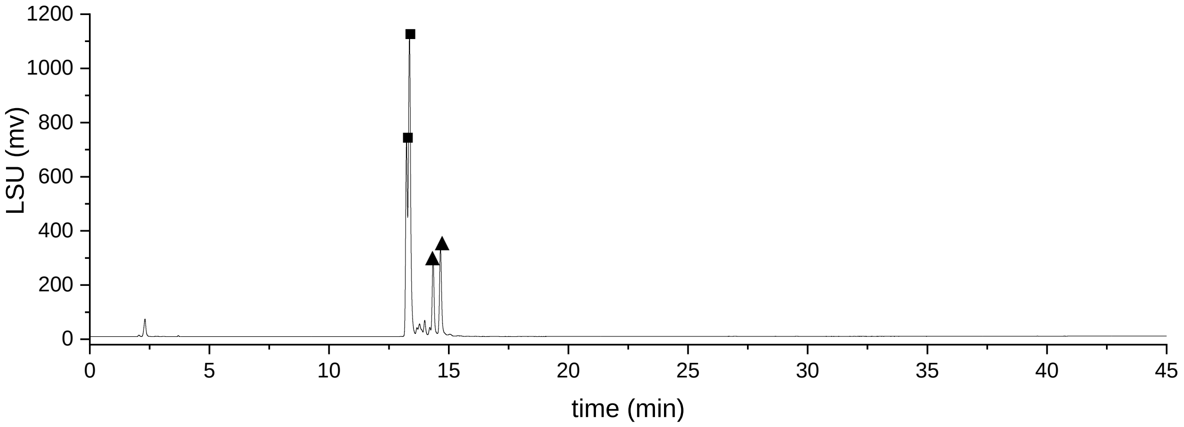

▲**9mer-IV** (α and β anomers, m/z 1461 [M+H]$^+$). ■ **5mer** deletion side product formed (α and β anomers, m/z 835 [M+Na]$^+$) due to incomplete glycosylation of Glc-1,3 glycosidic bond.

Characterization of 9mer-IV

^{1}H NMR (600 MHz, D_2O) δ 5.18 (d, *J* = 3.8 Hz, 0.4H, H-1α Glc), 4.94 – 4.87 (m, 2H, 1x H-1 Glc, 1x H-1 Rha), 4.70 (dd, *J* = 8.0, 2.0 Hz, 1H, H-1 Glc), 4.63 (d, *J* = 8.1 Hz, 0.6H, H-1β Glc), 4.55 – 4.45 (m, 6H, 5x H-1 Glc, H-5 Rha), 4.09 (d, *J* = 9.6 Hz, 0.5H), 4.05 (dt, *J* = 12.5, 2.9 Hz, 1H), 4.01 (dd, *J* = 9.3, 3.4 Hz, 1H), 3.97 (dd, *J* = 12.4, 4.8 Hz, 5H), 3.93 – 3.88 (m, 3H), 3.82 (dq, *J* = 11.9, 4.4 Hz, 5.5H), 3.77 – 3.70 (m, 4.5H), 3.69 (d, *J* = 2.7 Hz, 0.5H) 3.68 – 3.59 (m, 13H), 3.55 (dq, *J* = 6.2, 3.1 Hz, 2H), 3.53 – 3.46 (m, 6H), 3.40 (t, *J* = 9.4 Hz, 2H), 3.35 (ddd, *J* = 10.7, 5.5, 2.5 Hz, 5H), 3.30 (dd, *J* = 9.4, 8.1 Hz, 2H), 1.33 (d, *J* = 6.3 Hz, 3H, CH_3-6 Rha). ^{13}C NMR (151 MHz, D_2O) δ103.49 (C-1 Glc), 102.75 (C-1 Glc), 102.58 (C-1 Glc) , 102.54 (C-1 Glc), 102.40 (C-1 Glc), 102.36 (C-1 Glc), 101.84 (C-1 Rha), 101.72 (C-1 Rha), 99.53 (C-1 Glc), 95.75 (C-1β Glc), 91.94 (C-1α Glc), 81.55, 78.44, 78.26, 75.93, 75.48, 75.41, 74.71, 74.07, 73.99, 73.10, 73.01, 72.85, 69.39, 67.22 (C-5 Rha), , 60.51, 59.89, 16.88 (C-6 Rha). ESI-HRMS m/z 1461.499 $[M+H]^+$ ($C_{54}H_{93}O_{45}$ requires 1461.499).

RP-HPLC of 9mer-IV (ELSD trace, Method B1, t_R = 14.3, 14.6 min)

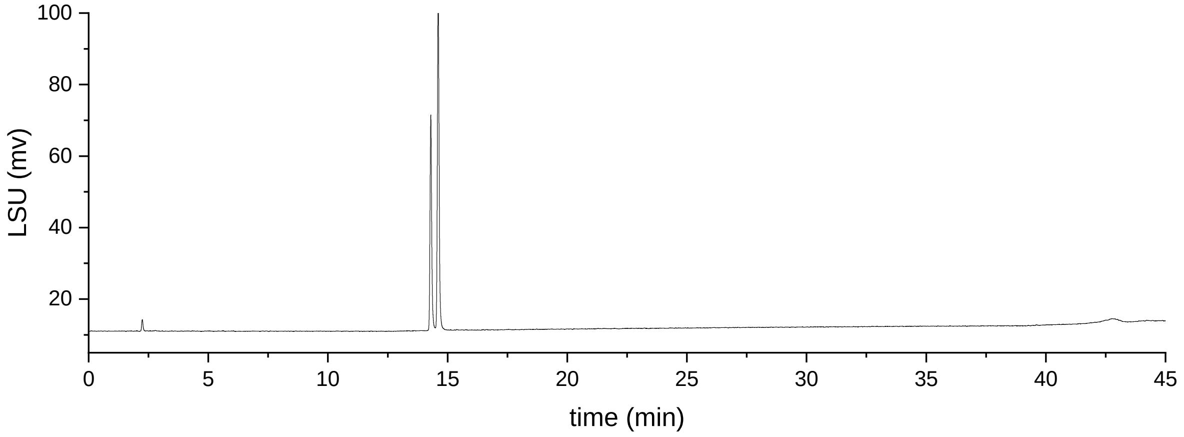

1H NMR of 9mer-IV (600 MHz, D_2O)

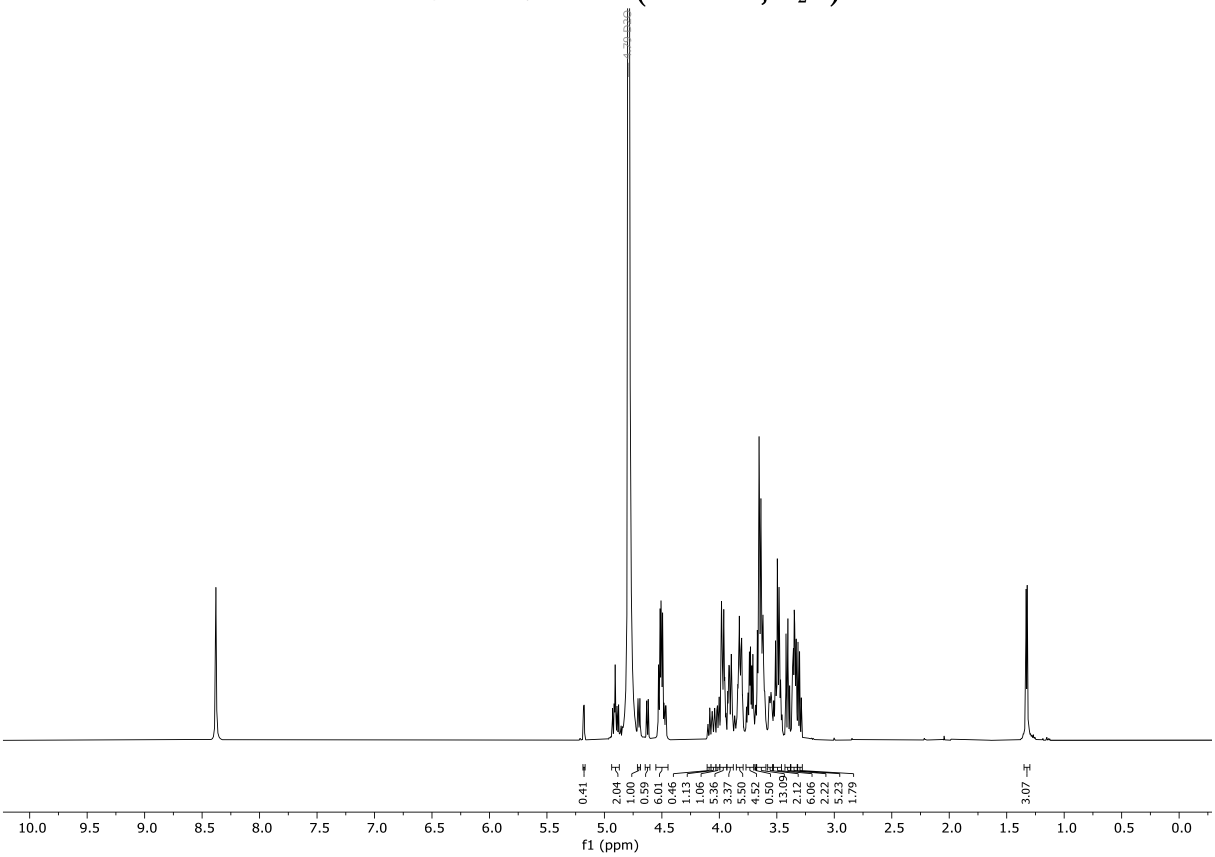

^{13}C NMR of 9mer-IV (151 MHz, D_2O)

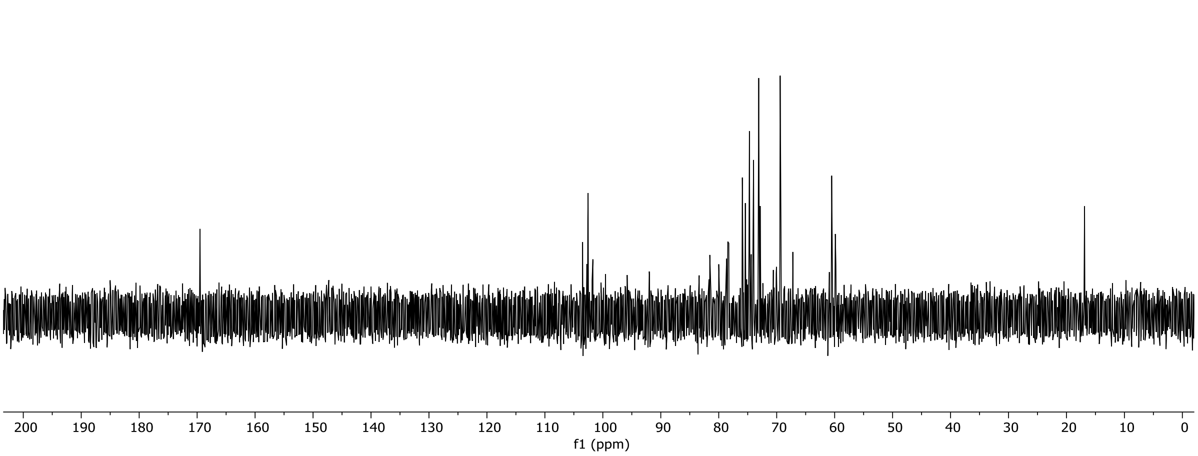

COSY NMR of 9mer-IV (D_2O)

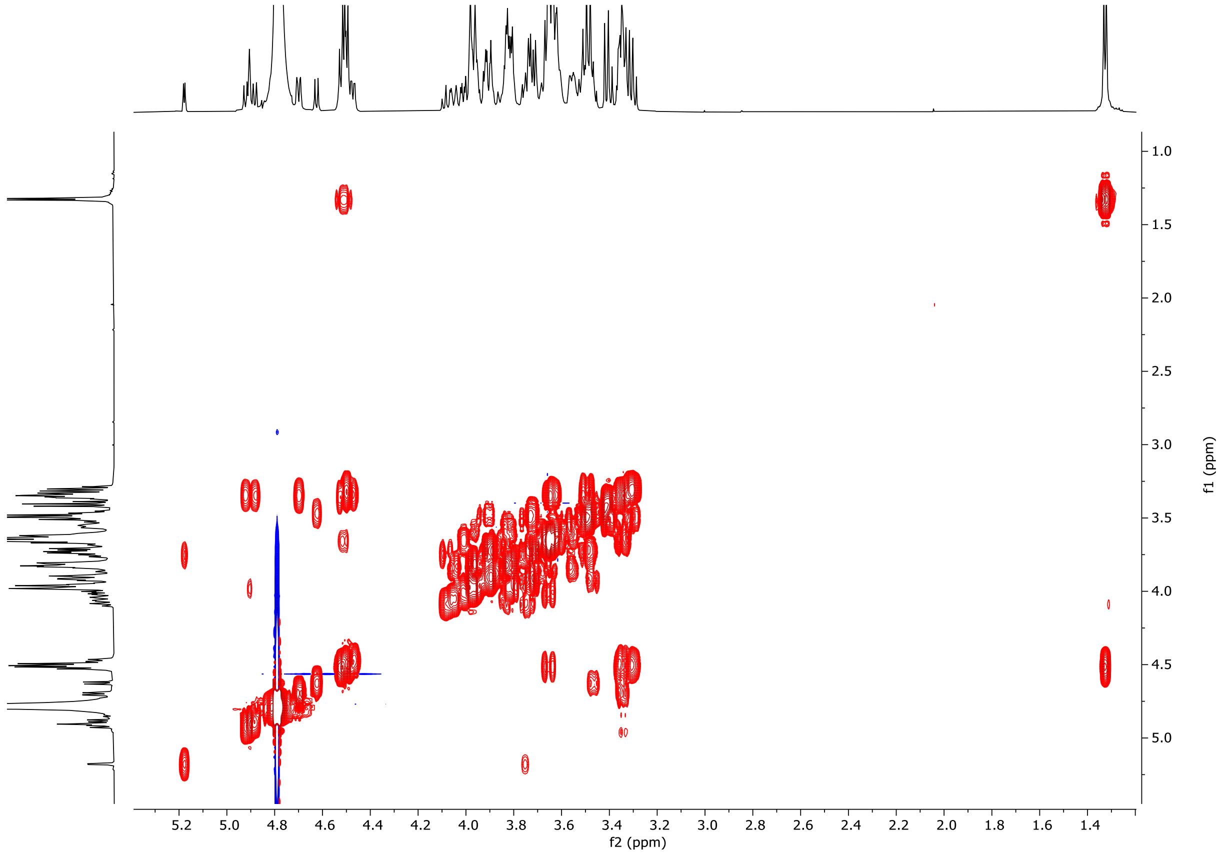

HSQC NMR of 9mer-IV (D_2O)

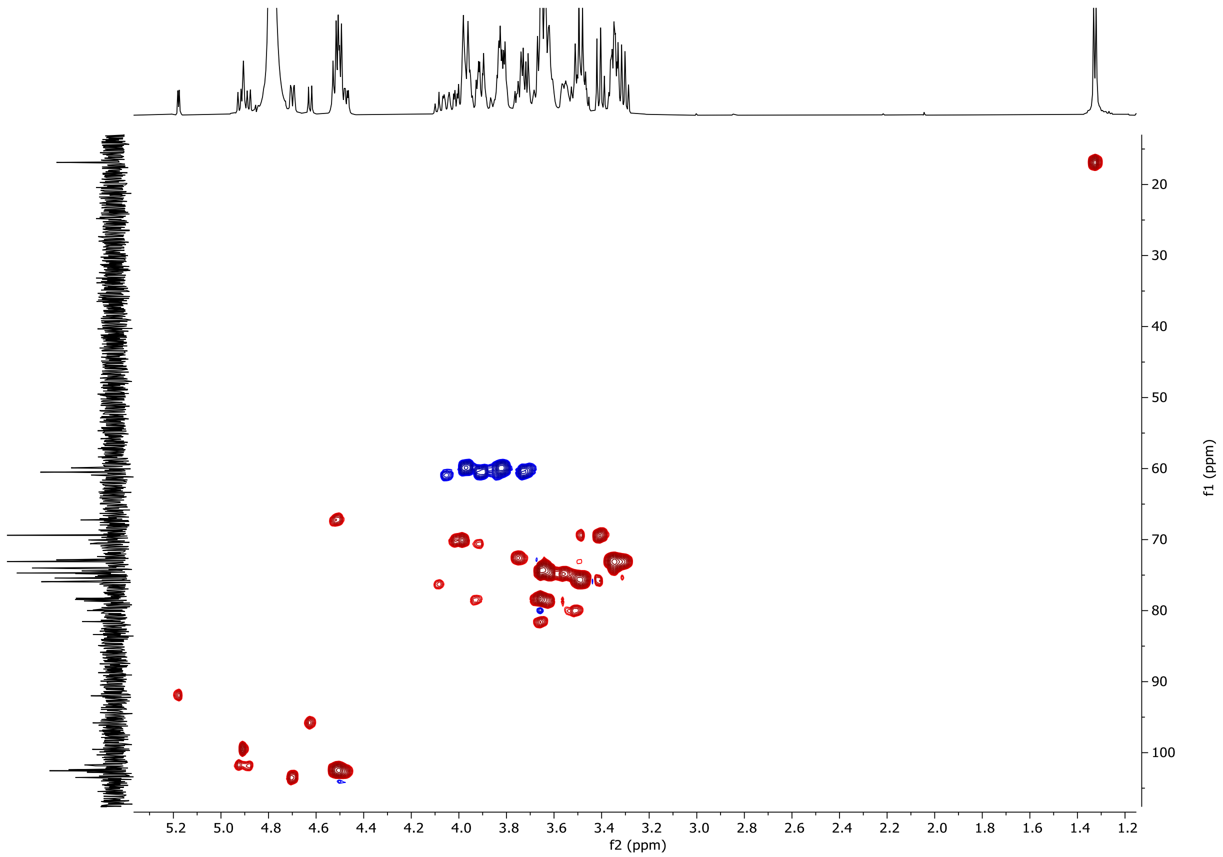

3.6.6 13mer-III

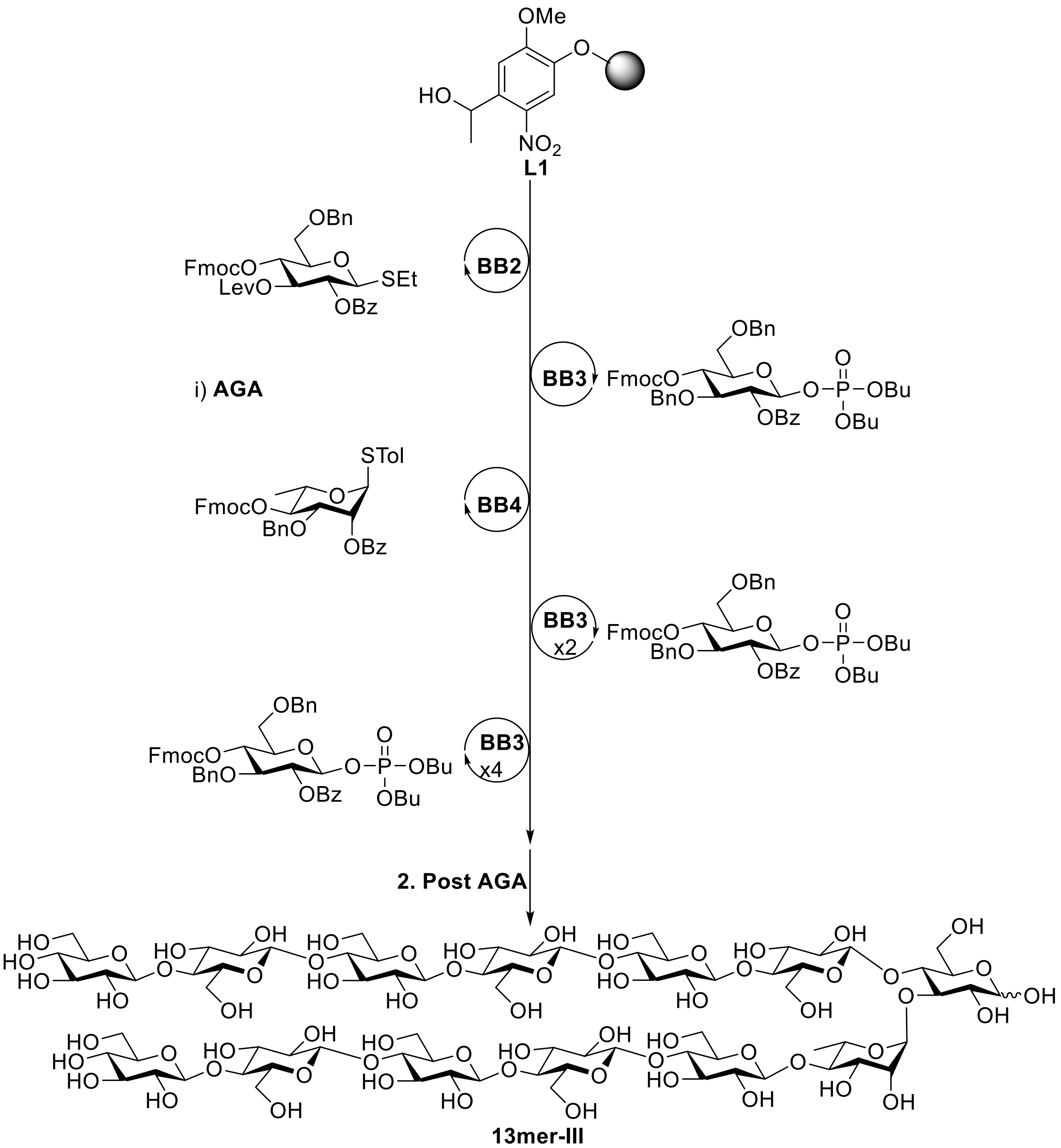

Step	BB	Modules	Notes
AGA	-	**A**	**L1** swelling
	**BB2**	**B**, **C1**, **D**, **E1**	**C1**: (**BB2**, -20 °C for 5 min, 0 °C for 20 min)
	**BB3**	**B**, **C3***, **D**, **E2**	**C3***: (**BB3**, -30 °C for 5 min, -10 °C for 40 min) *Double cycle
	**BB4**	**B**, **C1**, **D**, **E1**	**C1**: (**BB4**, -20 °C for 5 min, -10 °C for 20 min)
	(**BB3**)x2	(**B**, **C3***)x2 **D, E1**	**C3***: (**BB3**, -30 °C for 5 min, -10 °C for 40 min) *Double cycle
	(**BB3**)x4	(**B**, **C3***, **D**, **E1**)x4	**C3***: (**BB3**, -30 °C for 5 min, -10 °C for 40 min) *Double cycle
Post-AGA	-	**G**, **H1**, **I1**, **J**	**G**: (24 h) **I1**: (10 h) **J**: (Method B2: 14.7 min)

Automated synthesis, global deprotection, and purification afforded **13mer-III** as a white solid (3.9 mg, 15% overall yield).

^{1}H NMR (700 MHz, D_2O) δ 5.24 (s, 0.5H, H-1β Rha), 5.19 (s, 0.5H, H-1α Rha), 5.15 (d, *J* = 3.7 Hz, 0.4H, H-1α Glc), 4.66 (d, *J* = 7.9 Hz, 1H, H-1 Glc), 4.62 (d, *J* = 7.9 Hz, 0.6H, H-1β Glc), 4.47 (m, *J* = 20.1, 9.9 Hz, 11H, 10x H-1 Glc, H-5 Rha), 4.05 (dd, *J* = 19.3, 10.5 Hz, 2H), 4.00 (s, 1H), 3.93 (d, *J* = 11.3 Hz, 9H), 3.87 (d, *J* = 12.1 Hz, 2H), 3.78 (d, *J* = 12.2 Hz, 12H), 3.69 (dd, *J* = 12.4, 5.8 Hz, 2H), 3.65 – 3.56 (m, 26H), 3.53 (s, 3H), 3.45 (q, *J* = 9.7 Hz, 5H), 3.37 (t, *J* = 9.6 Hz, 2H), 3.29 (dt, *J* = 30.9, 9.3 Hz, 11H), 1.30 – 1.27 (m, 3H, CH_3-6 Rha). ^{13}C NMR (176 MHz, D_2O) δ 103.53 (C-1 Glc), 102.48 (C-1 Glc), 102.29 (C-1 Glc), 100.96 (C-1 Glc), 100.03 (C-1α Rha), 99.89 (C-1β Rha), 95.6 (C-1β Glc), 91.85 (C-1α Glc), 78.64, 78.24, 75.89, 75.37, 75.13, 74.72, 74.64, 74.45, 73.99, 73.93, 73.88, 73.05, 72.83, 70.03, 69.87, 69.34, 66.81 (C-5 Rha), 60.46, 59.73, 16.57 (C-6 Rha). ESI-HRMS m/z 2131.690 [M+Na]$^+$ ($C_{78}H_{132}O_{65}Na$ requires 2131.692)

RP-HPLC of 13mer-III (ELSD trace, Method B1, t_R = 15.0 min)

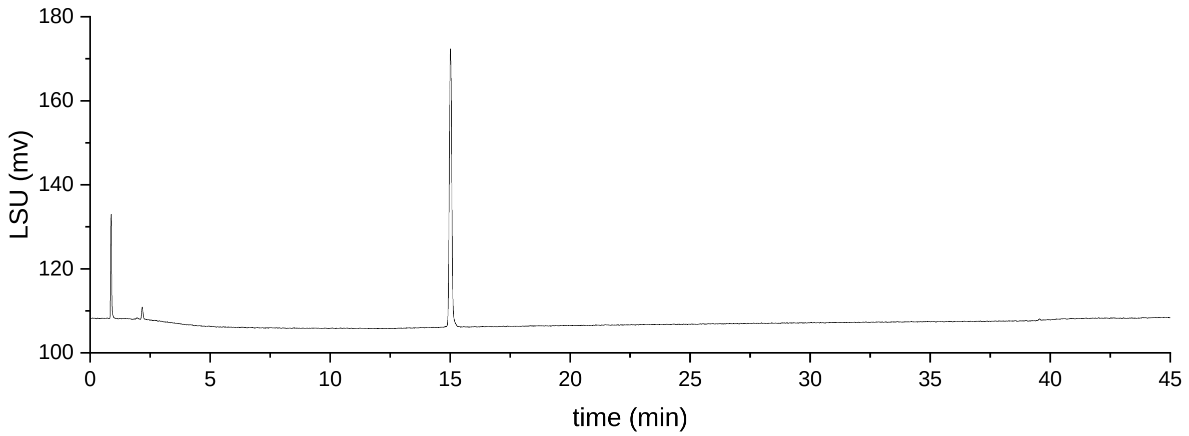

^{1}H NMR of 13mer-III (700 MHz, D_2O)

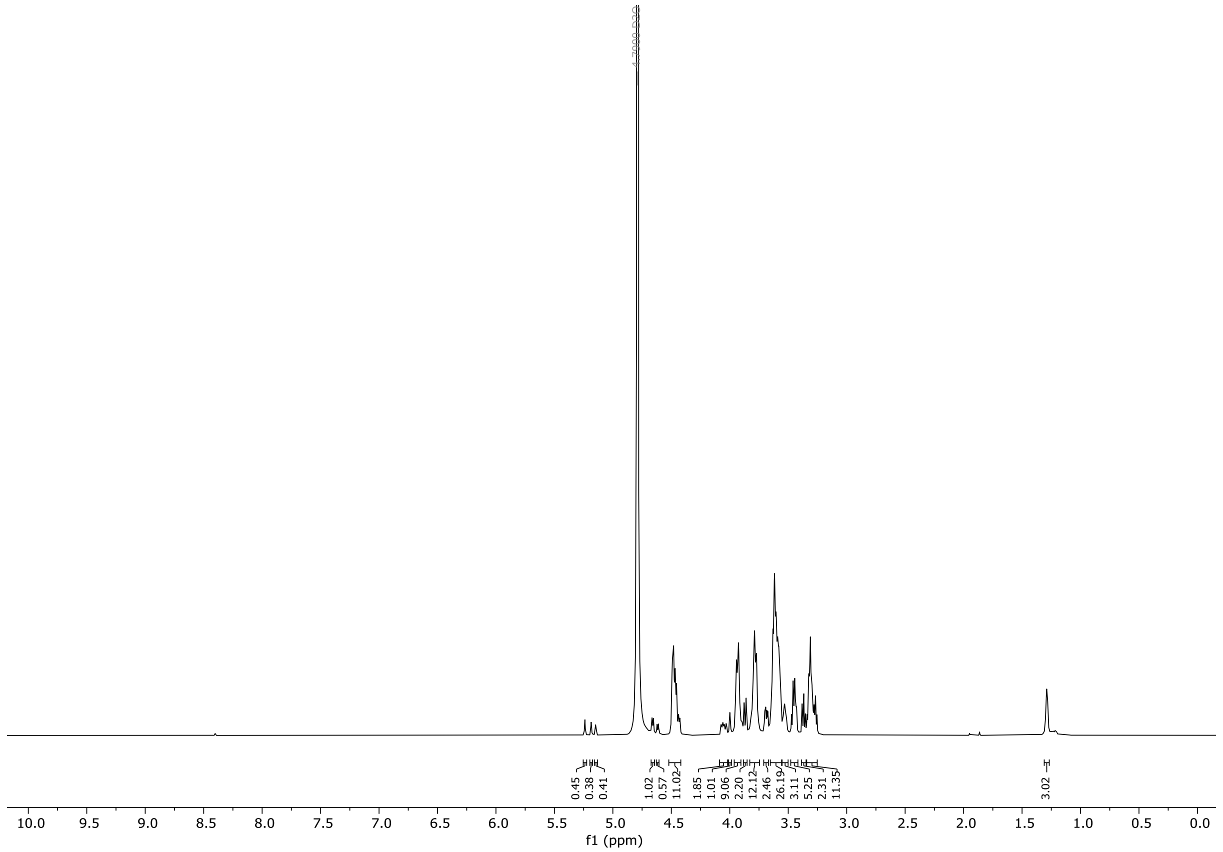

^{13}C NMR of 13mer-III (176 MHz, D_2O)

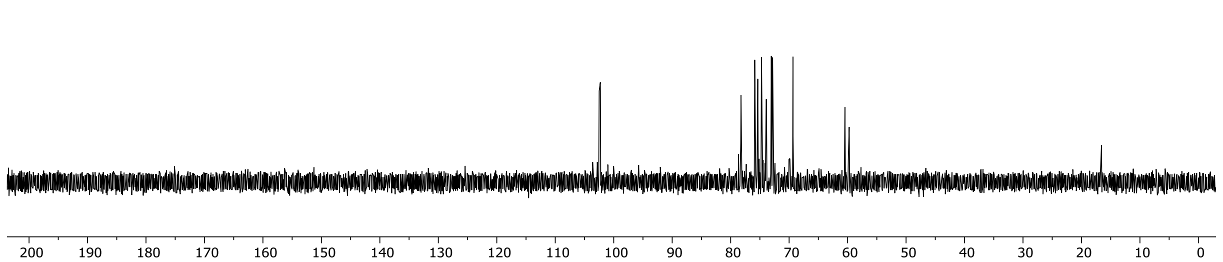

COSY NMR of 13mer-III (D_2O)

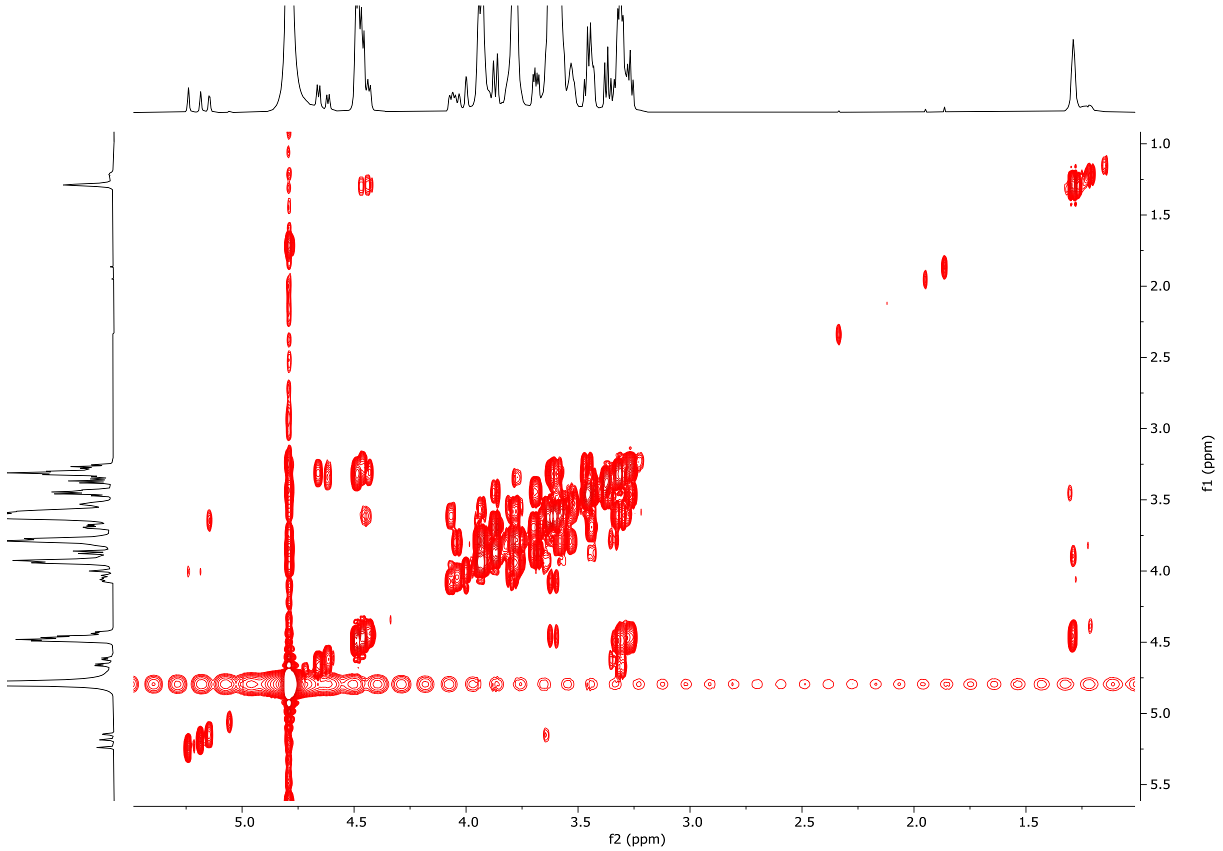

HSQC NMR of 13mer-III (D_2O)

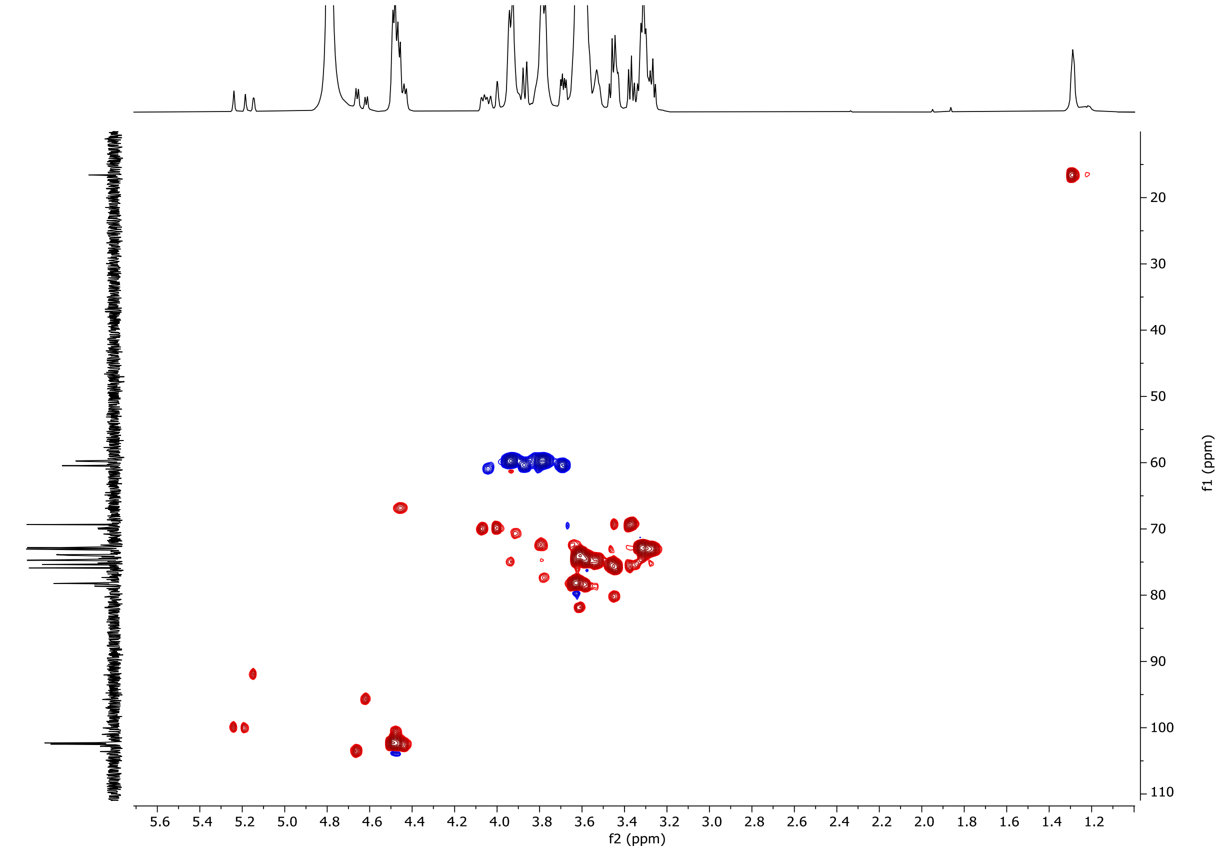

3.6.7 15mer-III

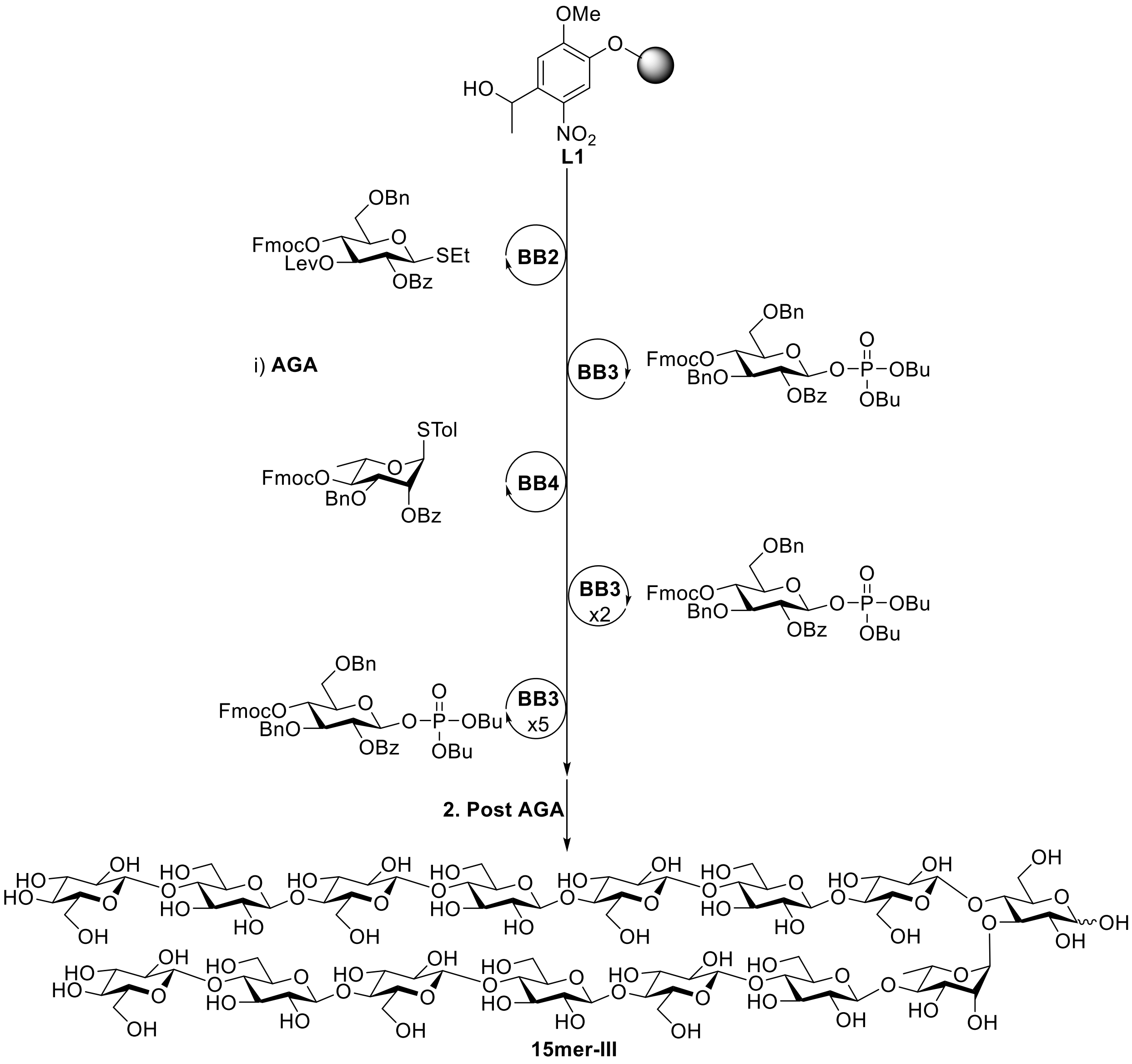

Step	BB	Modules	Notes
AGA	-	**A**	**L1** swelling
	**BB2**	**B**, **C1**, **D**, **E1**	**C1**: (**BB2**, -20 °C for 5 min, 0 °C for 20 min)
	**BB3**	**B**, **C2***, **D**, **E2**	**C2***: (**BB3**, -30 °C for 5 min, -10 °C for 40 min) *Double cycle
	**BB4**	**B**, **C1**, **D**, **E1**	**C1**: (**BB4**, -20 °C for 5 min, -10 °C for 20 min)
	(**BB3**) x2	(**B**, **C2***)x2 **D, E1**	**C2***: (**BB3**, -30 °C for 5 min, -10 °C for 40 min) *Double cycle
	(**BB3**)x5	(**B**, **C2***, **D**, **E1**)x5	**C2***: (**BB3**, -30 °C for 5 min, -10 °C for 40 min) *Double cycle
Post-AGA	-	**G**, **H1**, **I1**, **J**	**G**: (36 h)<br>**I1**: (14 h)<br>**J**: (Method B2: 15.9 min)

Automated synthesis, global deprotection, and purification afforded **15mer-III** as a white solid (2.02 mg, 7% overall yield).

^{1}H NMR (700 MHz, D_2O) δ 5.23 (s, 0.5H, H-1β Rha), 5.18 (s, 0.5H, H-1α Rha), 5.14 (d, *J* = 3.7 Hz, 0.4H, H-1α Glc), 4.65 (d, *J* = 7.9 Hz, 1H, H-1 Glc), 4.61 (d, *J* = 8.0 Hz, 0.6H, H-1β Glc), 4.47 (dd, *J* = 17.3, 7.9 Hz, 13H, 12x H-1 Glc, H-5 Rha), 4.06 (dd, *J* = 8.5, 5.6 Hz, 1H), 4.03 (s, 1H), 4.00 (s, 1H), 3.93 (d, *J* = 12.0 Hz, 11H), 3.88 – 3.85 (m, 2H), 3.77 (d, *J* = 13.4 Hz, 13H), 3.68 (dd, *J* = 12.4, 5.8 Hz, 2H), 3.65 – 3.55 (m, 34H), 3.52 (s, 3H), 3.47 – 3.42 (m, 5H), 3.36 (t, *J* = 9.5 Hz, 2H), 3.33 (s, 1H), 3.30 (t, *J* = 8.4 Hz, 11H), 3.26 (t, *J* = 8.6 Hz, 2H), 1.30 – 1.27 (m, 3H, CH_3-6 Rha). ^{13}C NMR (176 MHz, D_2O) δ 106.60 (C-1 Glc), 103.18 (C-1α Rha), 103.10 (C-1β Rha), 102.48, 102.27, 75.89, 75.36, 74.72, 72.83, 70.33 (C-5 Rha), 69.34, 68.40, 19.94 (C-6 Rha). MALDI-TOF m/z 2455.3 $[M+Na]^+$ ($C_{90}H_{152}O_{75}Na$ requires 2455.7).

RP-HPLC of 15mer-III (ELSD trace, Method B1, t_R = 15.5 min)

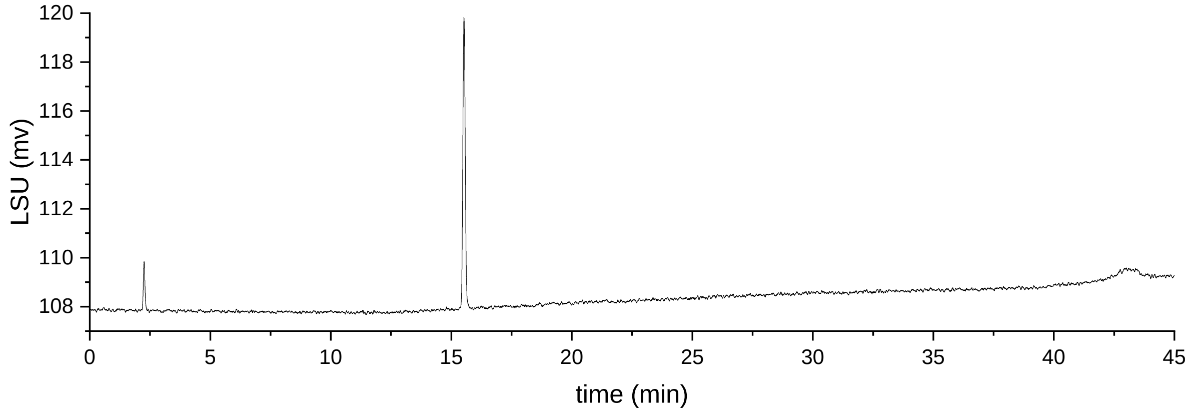

MALDI-TOF of 15mer-III

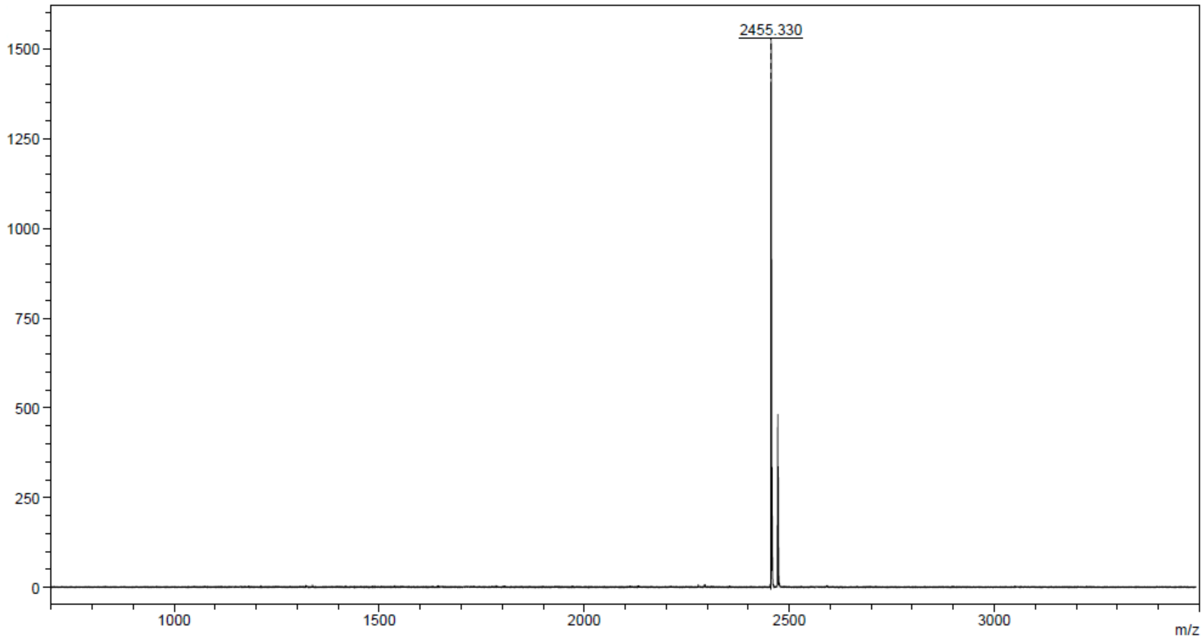

^{1}H NMR of 15mer-III (700 MHz, D_2O)

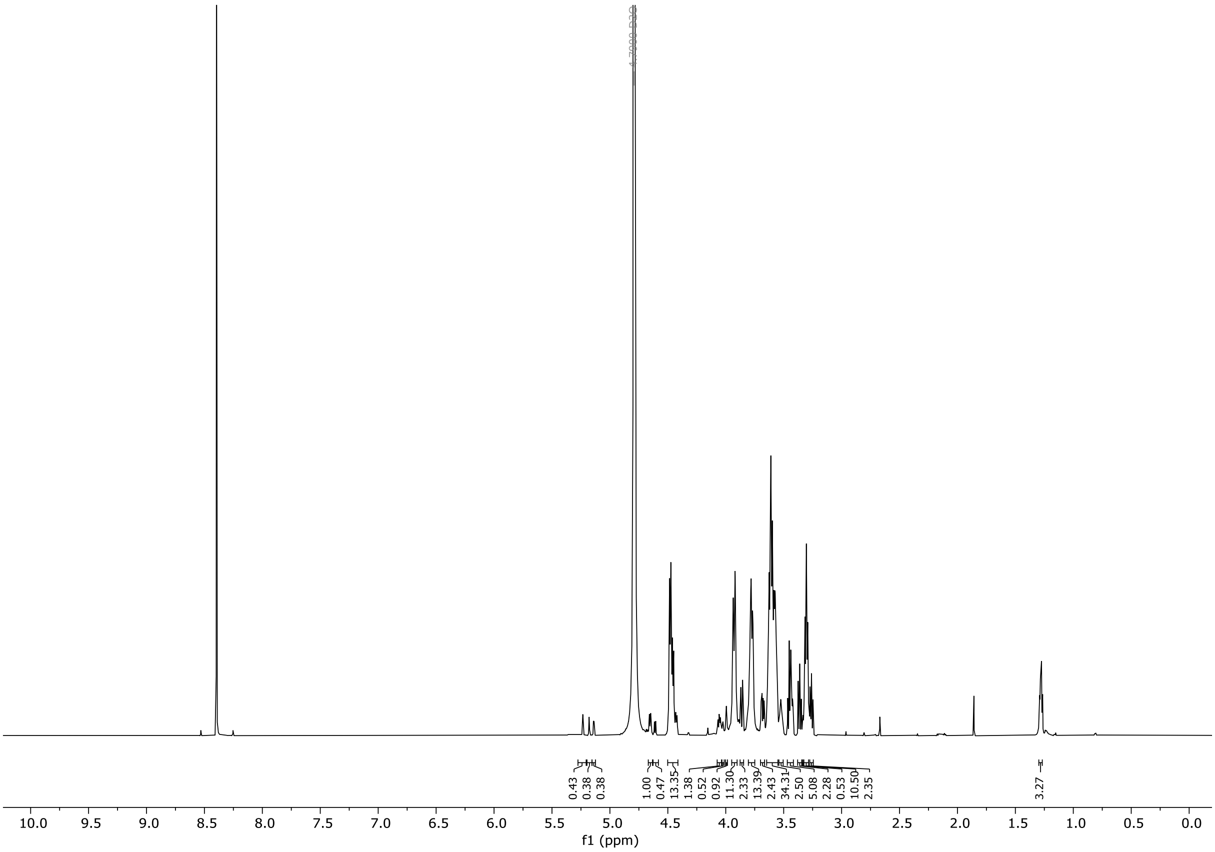

^{13}C NMR of 15mer-III (176 MHz, D_2O)

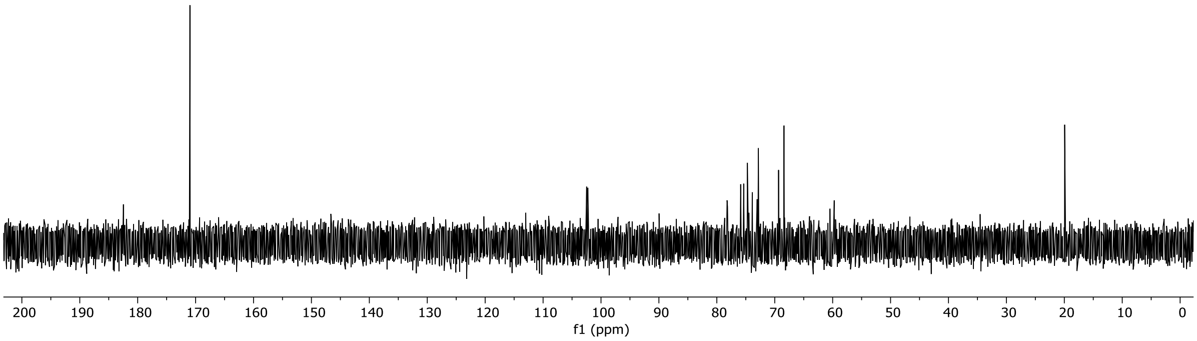

COSY NMR of 15mer-III (D_2O)

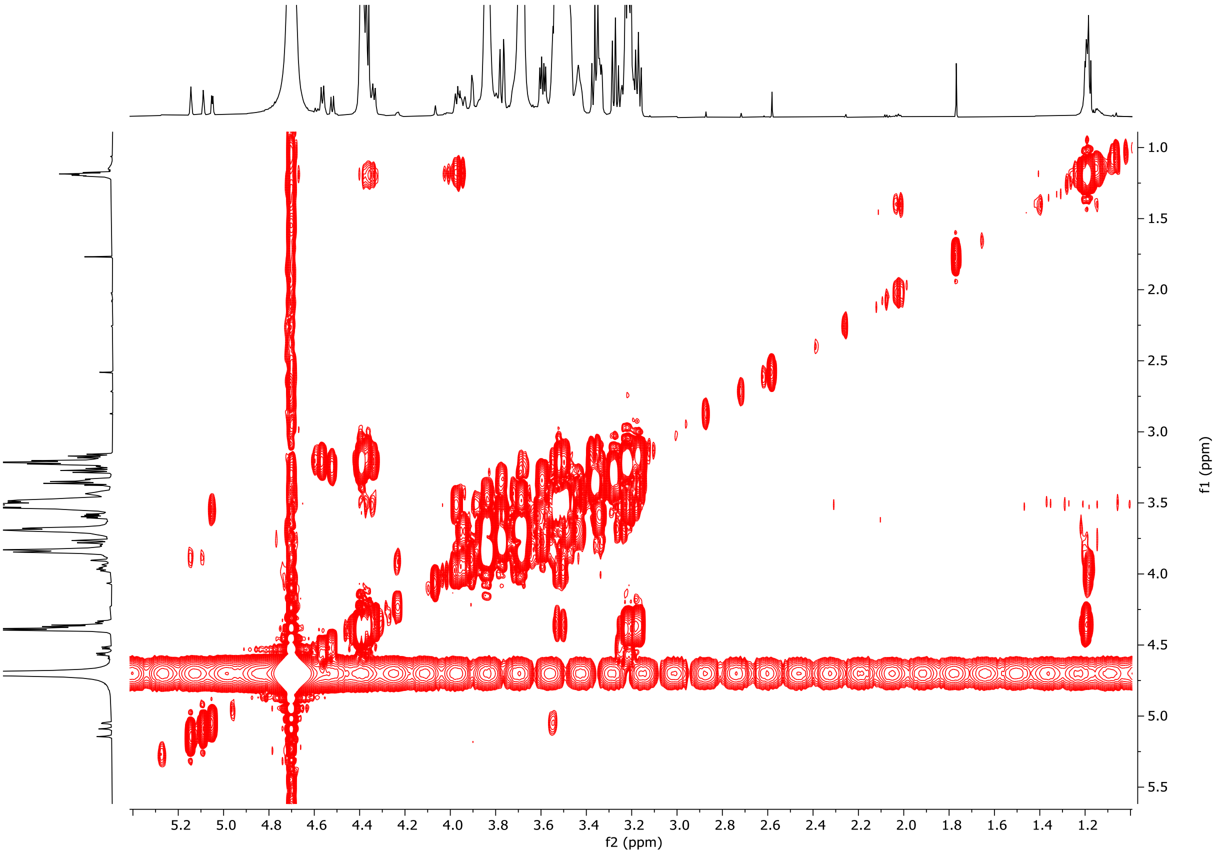

HSQC NMR of 15mer-III (D_2O)

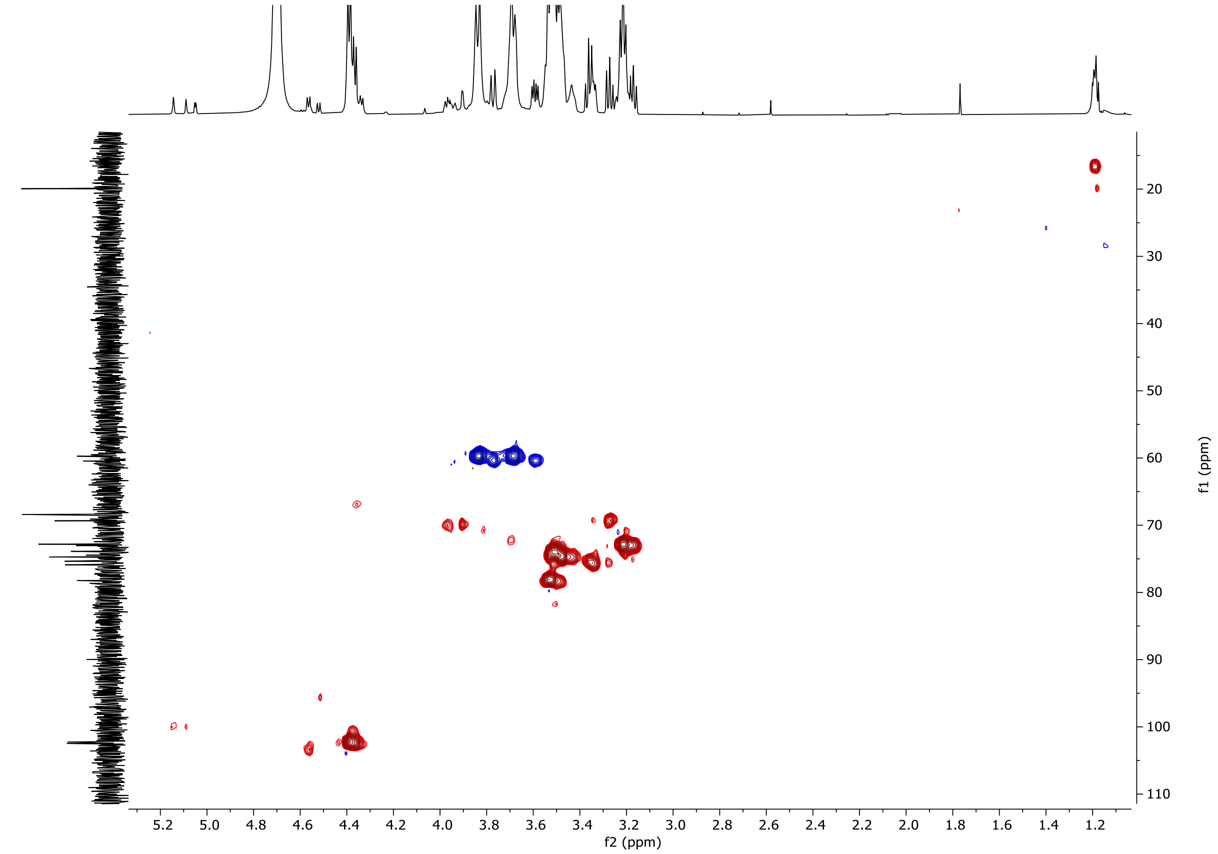

3.6.8 17mer-III

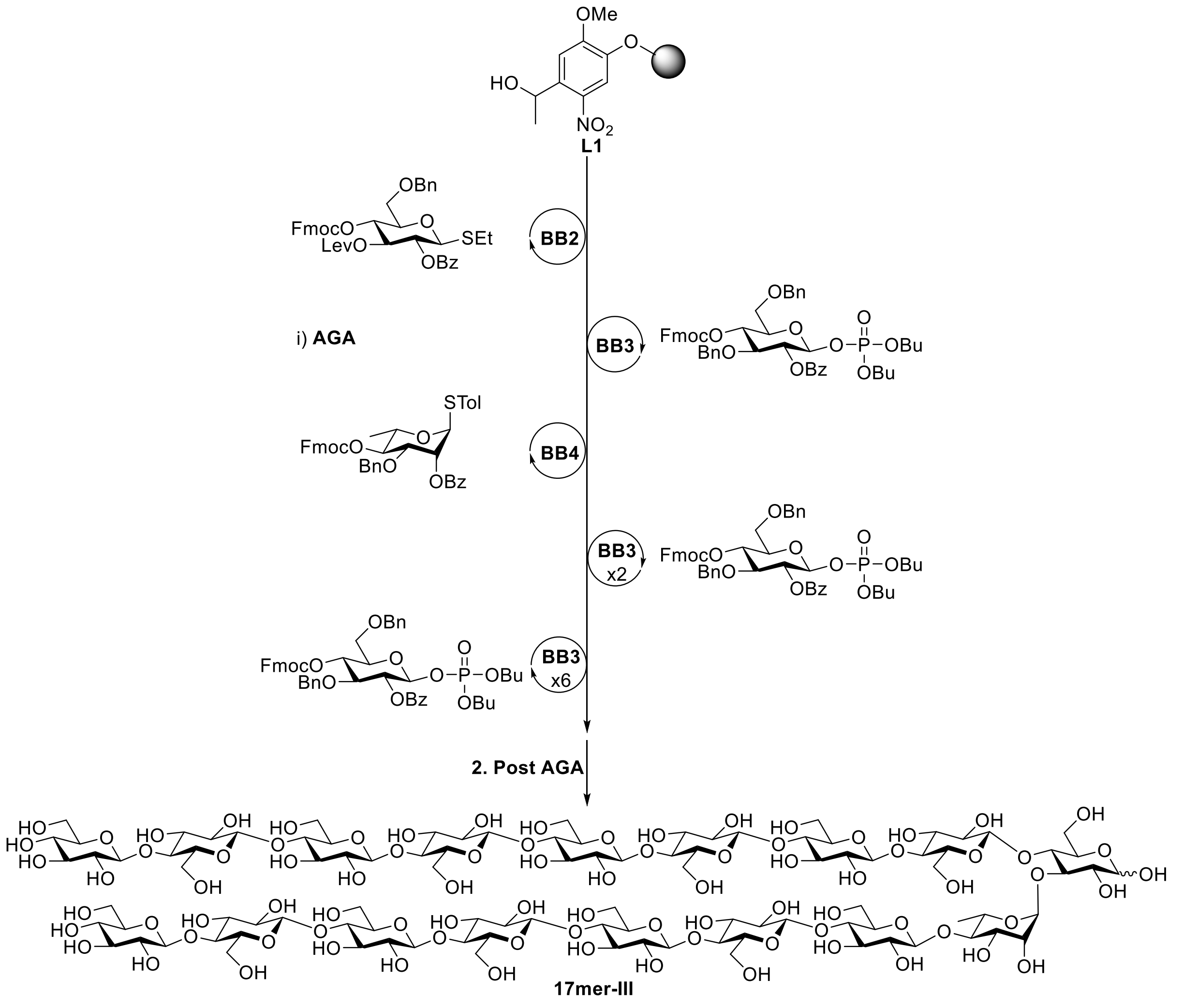

Step	BB	Modules	Notes
AGA	-	**A**	**L1** swelling
	**BB2**	**B**, **C1**, **D**, **E1**	**C1**: (**BB2**, -20 °C for 5 min, 0 °C for 20 min)
	**BB3**	**B**, **C3***, **D**, **E2**	**C3***: (**BB3**, -30 °C for 5 min, -10 °C for 40 min) *Double cycle
	**BB4**	**B**, **C1**, **D**, **E1**	**C1**: (**BB4**, -20 °C for 5 min, -10 °C for 20 min)
	(**BB3**)x2	(**B**, **C3***)x2<br>**D, E1**	**C3***: (**BB3**, -30 °C for 5 min, -10 °C for 40 min) *Double cycle
	(**BB3**)x6	(**B**, **C3***, **D**, **E1**)x6	**C3***: (**BB3**, -30 °C for 5 min, -10 °C for 40 min) *Double cycle
Post-AGA	-	**G**, **H1**, **I1**, **J**	**G**: (40 h)<br>**I1**: (13 h)<br>**J**: (Method A2: 27.4 min)

Automated synthesis, global deprotection, and purification afforded **17mer-III** as a white solid (1.3 mg, 4% overall yield).

^{1}H NMR (600 MHz, D_2O) δ 5.27 (s, 0.5H, H-1 Rha), 5.22 (s, 0.5H, H-1 Rha), 5.18 (d, *J* = 3.8 Hz, 0.4H, H-1α Glc), 4.69 (d, *J* = 7.5 Hz, H-1 Glc), 4.65 (d, *J* = 7.9 Hz, 0.6H, H-1β Glc), 4.51 (dd, *J* = 14.6, 7.9 Hz, 15H, 14x H-1 Glc, H-5 Rha), 4.12 – 4.05 (m, 2H), 4.04 (s, 1H), 3.97 (d, *J* = 12.0 Hz, 12H), 3.90 (d, *J* = 11.7 Hz, 2H), 3.88 – 3.76 (m, 15H), 3.75 – 3.70 (m, 3H), 3.65 (td, *J* = 13.7, 6.2 Hz, 37H), 3.57 (s, 2H), 3.49 (t, *J* = 9.2 Hz, 5H), 3.40 (t, *J* = 9.5 Hz, 2H), 3.38 – 3.32 (m, 14H), 3.30 (t, *J* = 8.7 Hz, 3H), 1.32 (d, *J* = 6.8 Hz, 3H, CH_3-6 Rha). ^{13}C NMR was not recorded due to the low solubility of **17mer-III** in D_2O. MALDI-TOF m/z 2779.0 $[M+Na]^+$ ($C_{102}H_{172}O_{85}Na$ requires 2779.9).

RP-HPLC of 17mer-III (ELSD trace, Method A1, t_R = 26.5 min)

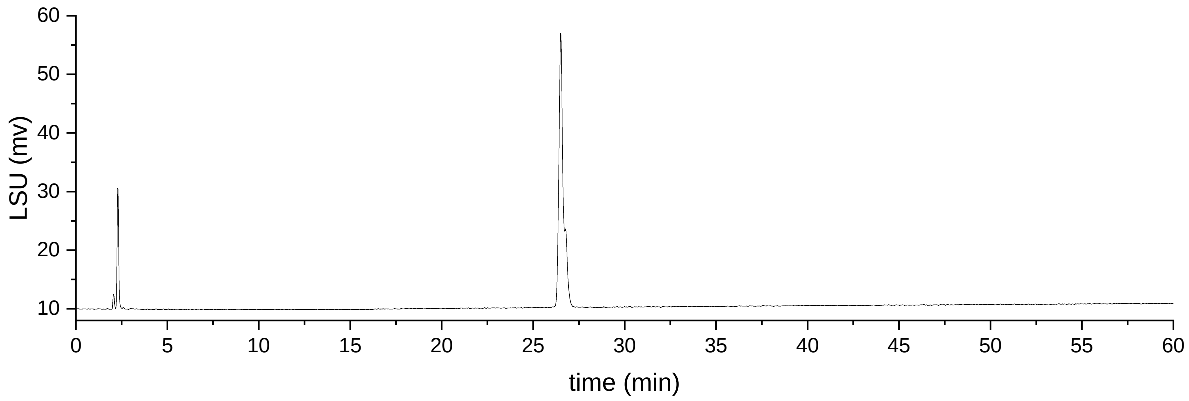

MALDI-TOF of 17mer-III

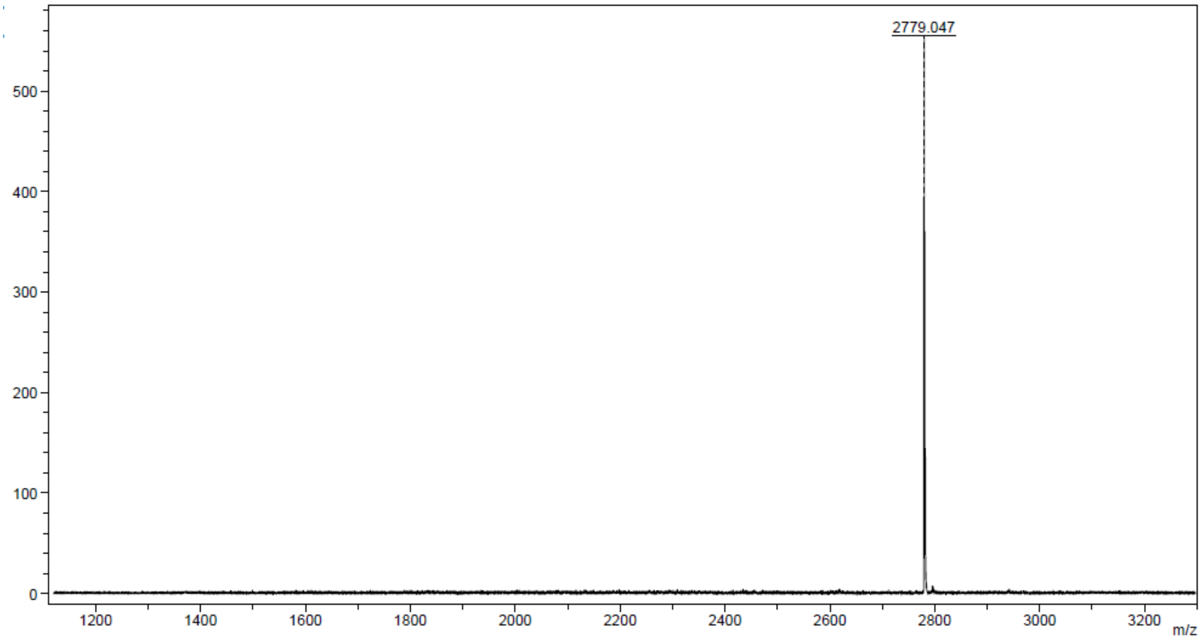

^{1}H NMR of 17mer-III (600 MHz, D_2O)

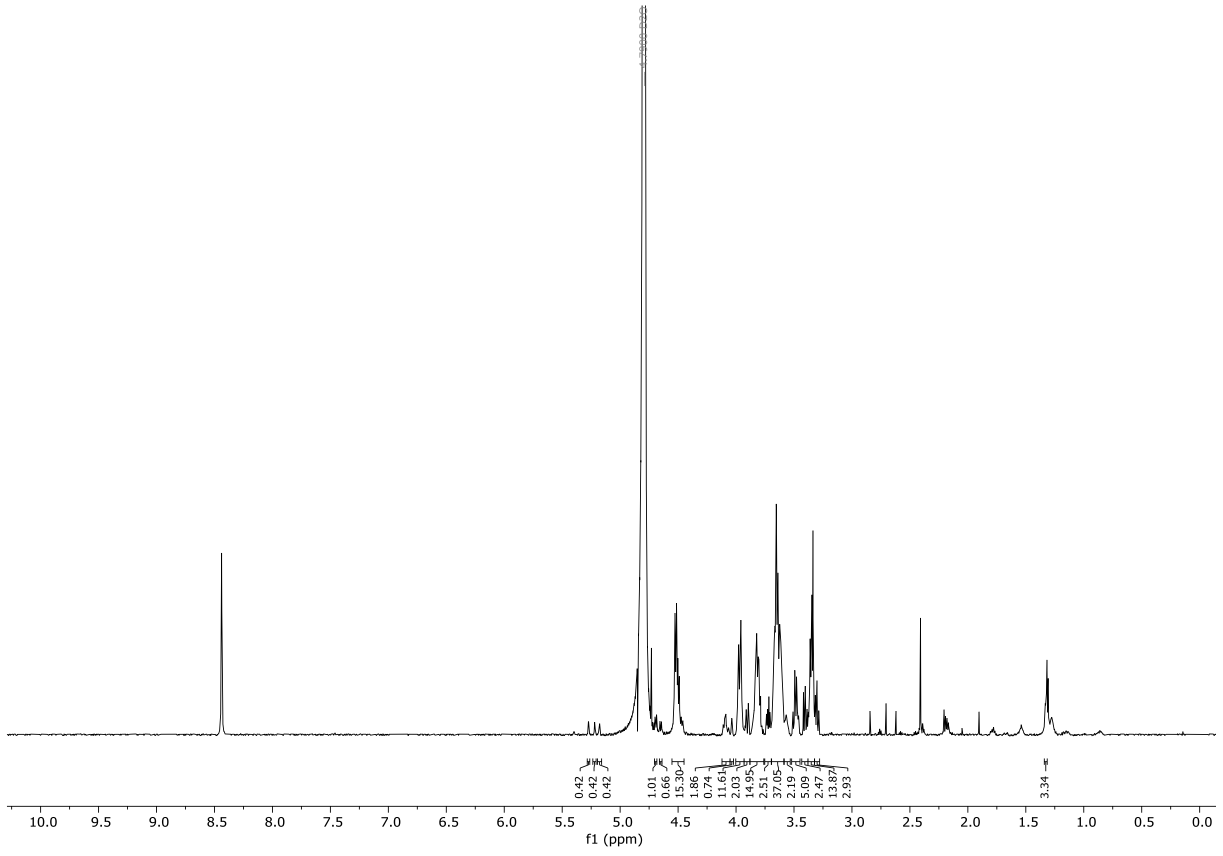

3.6.9 7mer-III-F

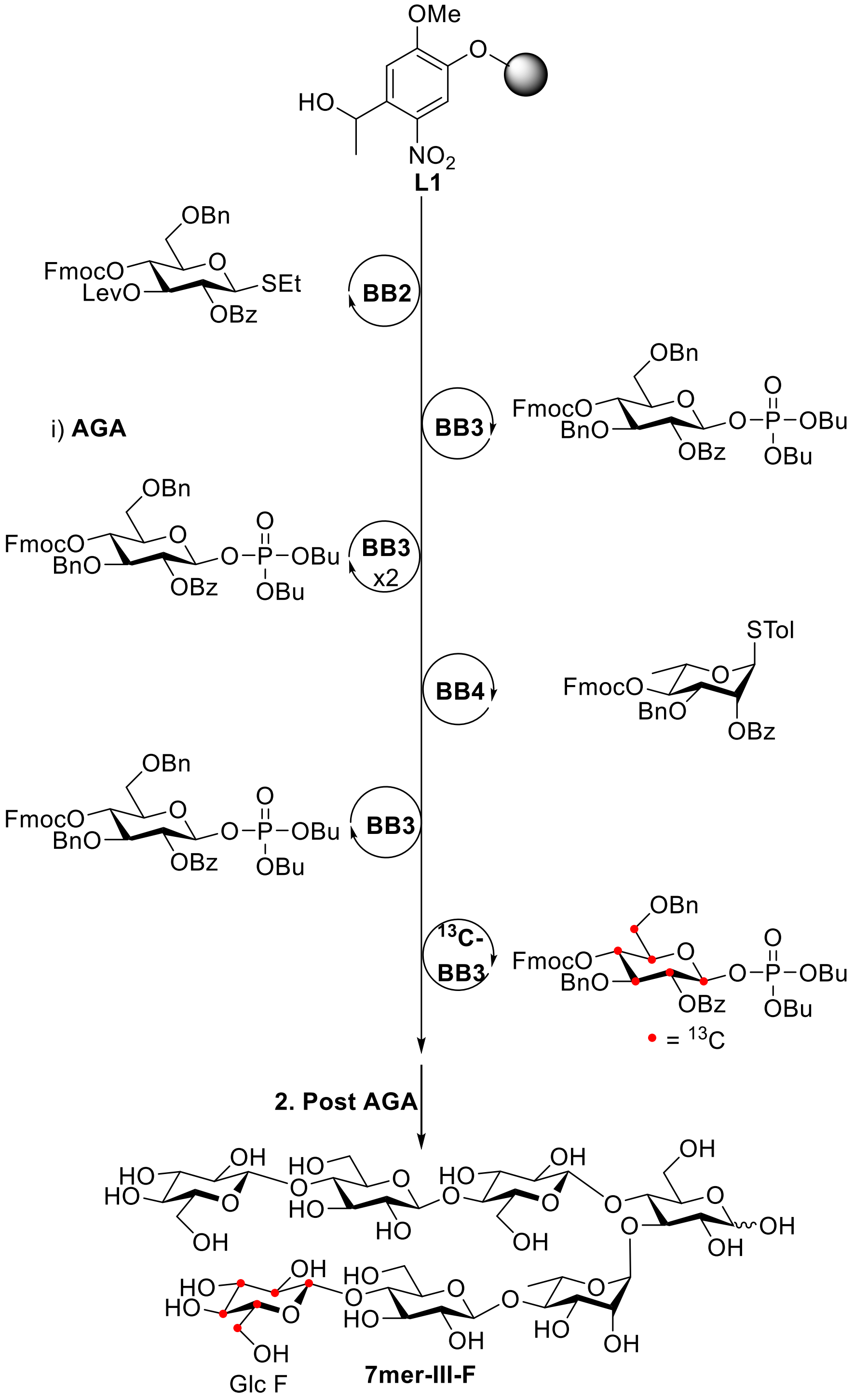

Step	BB	Modules	Notes
AGA	-	**A**	**L1** swelling
	**BB2**	**B**, **C1**, **D**, **E1**	**C1**: (**BB2**, -20 °C for 5 min, 0 °C for 20 min)
	**BB3**	**B**, **C3***, **D**, **E1**	**C3***: (**BB3**, -30 °C for 5 min, -10 °C for 40 min) *Double cycle
	(**BB3**)x2	(**B**, **C3**, **D**, **E1**)x2<br>**D**, **E2**	**C3**: (**BB3**, -30 °C for 5 min, -10 °C for 40 min)
	**BB4**	**B**, **C1**, **D**, **E1**	**C1**: (**BB4**, -20 °C for 5 min, 0 °C for 20 min)
	**BB3**	**B, C3*, B, C2, D, E1**	**C3***: (**BB3**, -30 °C for 5 min, -10 °C for 40 min) *Double cycle
	**^{13}C-BB3**	**B**, **C3**, **D**, **E1**	**C3**: (**^{13}C-BB3**, -30 °C for 5 min, -10 °C for 40 min)
Post-AGA	-	**G, H1, I1, J**	**G**: (24 h)<br>**I2**: (5 h)<br>**J**: (Method C2: 23.2 min)

Automated synthesis, global deprotection, and purification afforded **7mer-III-F** as a white solid (7.8 mg, 55% overall yield).

^{1}H NMR (700 MHz, D_2O) δ 5.24 (d, *J* = 1.8 Hz, 0.6 H, H-1β Rha), 5.18 (d, *J* = 1.8 Hz, 0.4H, H-1α Rha), 5.15 (d, *J* = 3.7 Hz, 0.4H, H-1α Glc), 4.66 (d, *J* = 8.0 Hz, 1H, H-1 Glc), 4.62 (d, *J* = 8.0 Hz, 0.6H, H-1β Glc), 4.55 (d, *J* = 7.7 Hz, 0.5H, H-1 ^{13}C-Glc), 4.50 – 4.42 (m, 4H, 3x H-1 Glc, H-5 Rha), 4.32 (d, *J* = 7.7 Hz, 0.5H, H-1 ^{13}C-Glc), 4.08 – 4.02 (m, 2H), 4.00 (dt, *J* = 3.1, 1.2 Hz, 1H), 3.97 (d, *J* = 12.2 Hz, 0.5H), 3.95 – 3.89 (m, 3H), 3.87 (dd, *J* = 12.4, 2.2 Hz, 1H), 3.83 – 3.75 (m, 7H), 3.68 (dd, *J* = 12.4, 6.0 Hz, 1H), 3.65 – 3.51 (m, 12H), 3.47 – 3.42 (m, 3H), 3.39 – 3.33 (m, 3H), 3.32 – 3.26 (m, 4H), 3.17 (dq, *J* = 13.3, 7.1 Hz, 0.5H), 1.29 (dd, *J* = 6.4, 4.1 Hz, 3H, CH_3-6 Rha). ^{13}C NMR (176 MHz, D_2O) δ 103.56 (d, *J* = 4.8 Hz), δ 102.63, δ 102.58 (d, *J* = 46.9 Hz), 100.99 (d, *J* = 5.0 Hz), 100.14, 100.02, 95.70, 92.00, 81.84 (d, *J* = 12.3 Hz), 80.25 (d, *J* = 10.2 Hz), 78.75, 78.39, 77.33, 75.92, 75.59 (dt, *J* = 79.0, 39.9 Hz), 74.62, 74.45, 74.19 (d, *J* = 3.5 Hz), 74.10 (d, *J* = 4.7 Hz), 73.95, 72.96 (ddd, *J* = 47.1, 39.1, 2.7 Hz), 72.48, 72.43 (d, *J* = 3.2 Hz), 70.78, 70.00 (d, *J* = 7.9 Hz), 69.38, 69.52 – 68.95 (m), 66.87 (d, *J* = 9.5 Hz), 60.95 (d, *J* = 6.2 Hz), 60.50 (d, *J* = 42.9 Hz), 59.79 (d, *J* = 20.9 Hz), 59.45 (d, *J* = 14.8 Hz), δ 16.56 (d, *J* = 3.3 Hz). ESI-HRMS m/z 1165.400 [M+Na]$^+$ ($C_{36}{}^{13}C_6H_{72}O_{35}Na$ requires 1165.395).

RP-HPLC of 7mer-III-E (ELSD trace, Method C1, t_R = 24.0, 24.3 min)

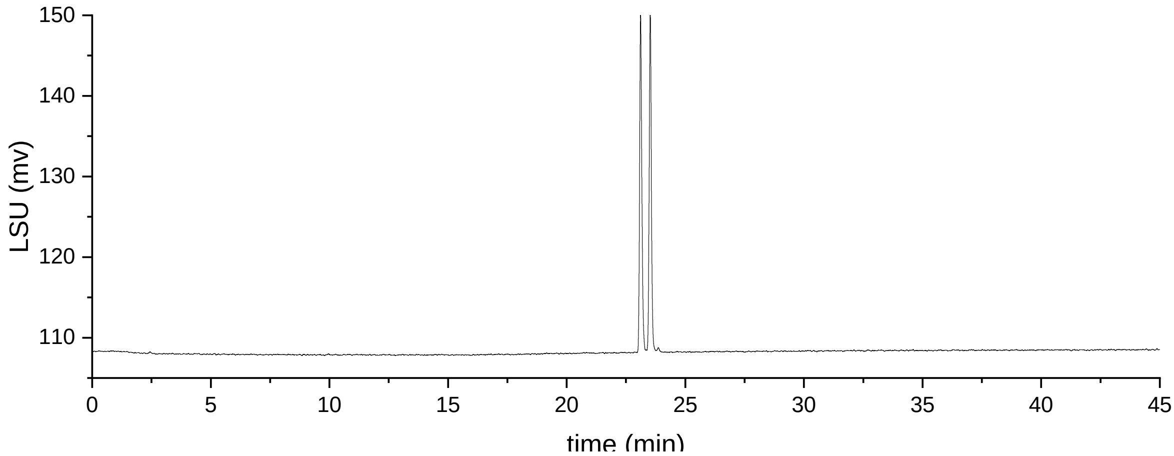

^{1}H NMR of 7mer-III-F (700 MHz, D_2O)

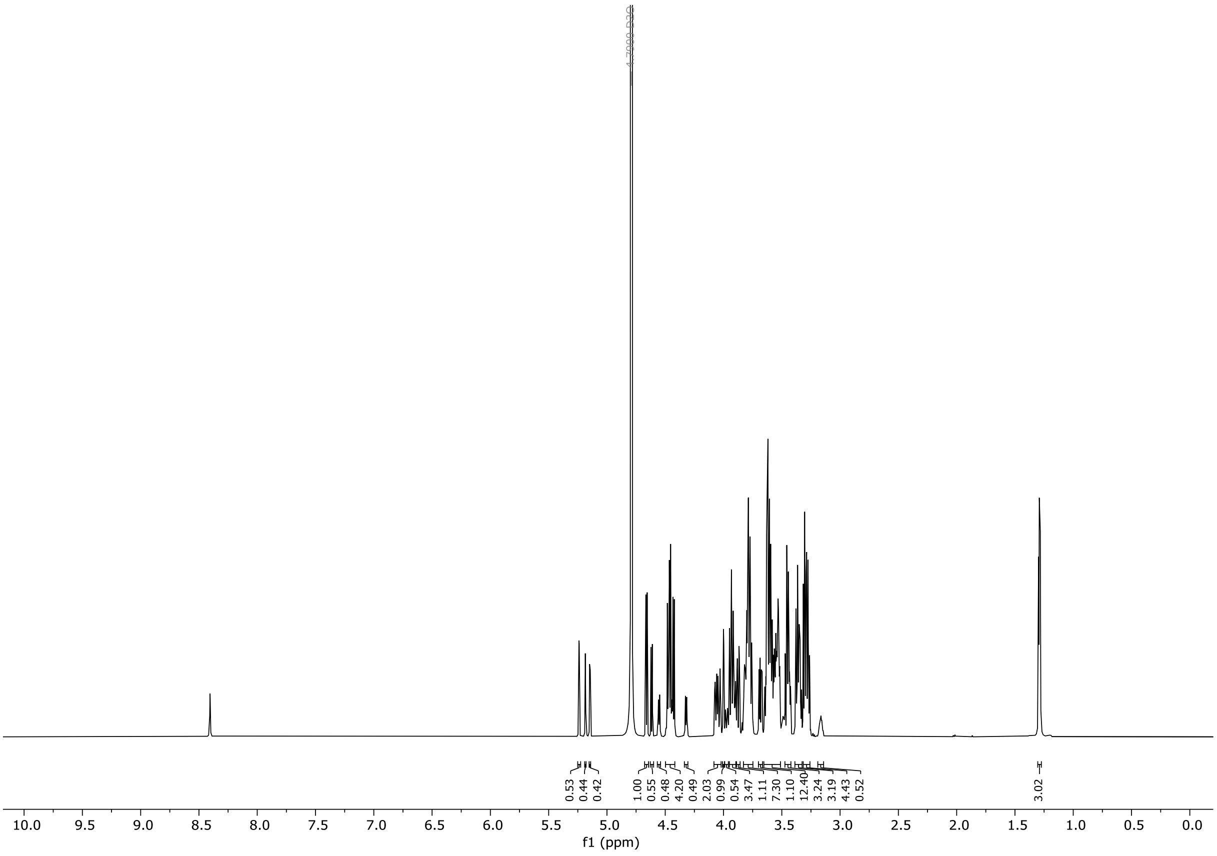

^{13}C NMR of 7mer-III-F (176 MHz, D_2O)

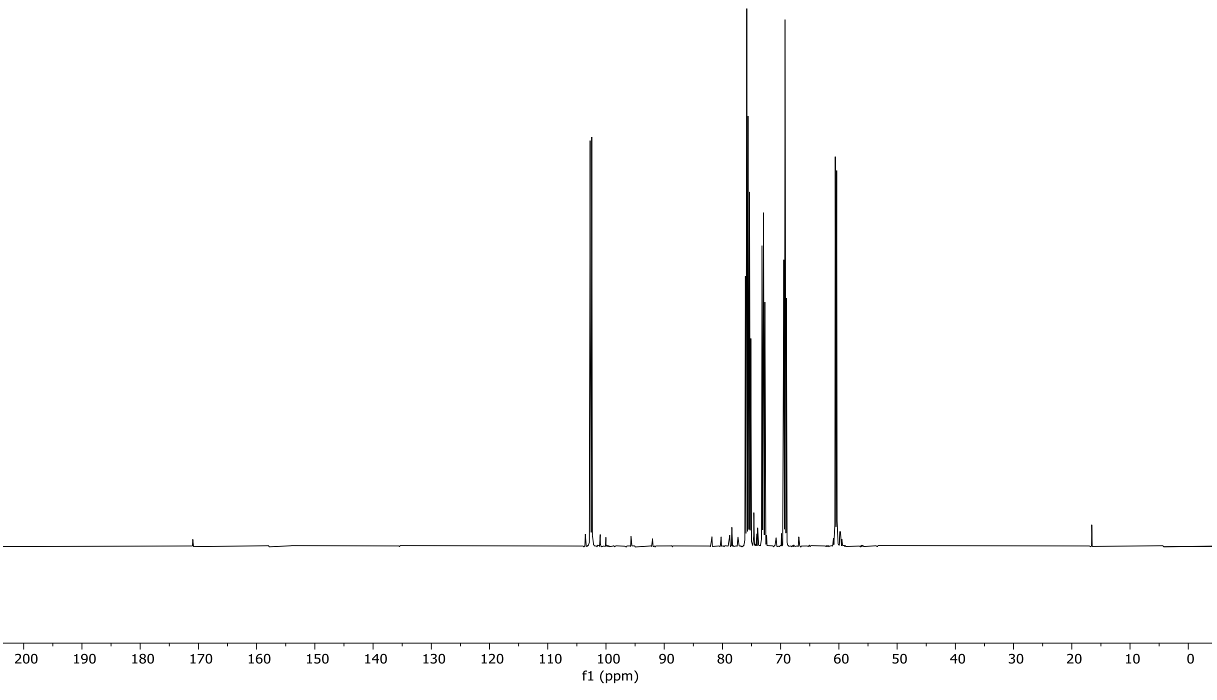

COSY NMR of 7mer-III-F (D_2O)

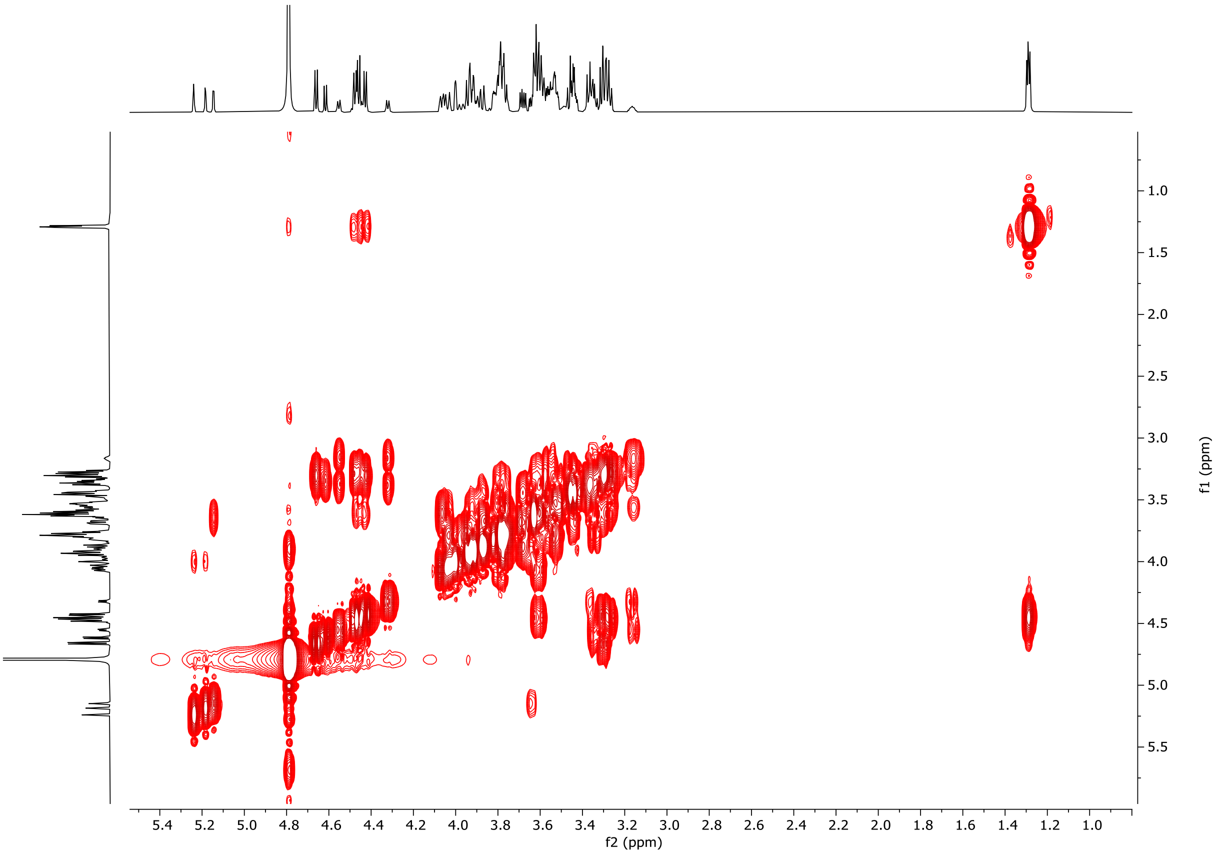

HSQC NMR of 7mer-III-F (D_2O)

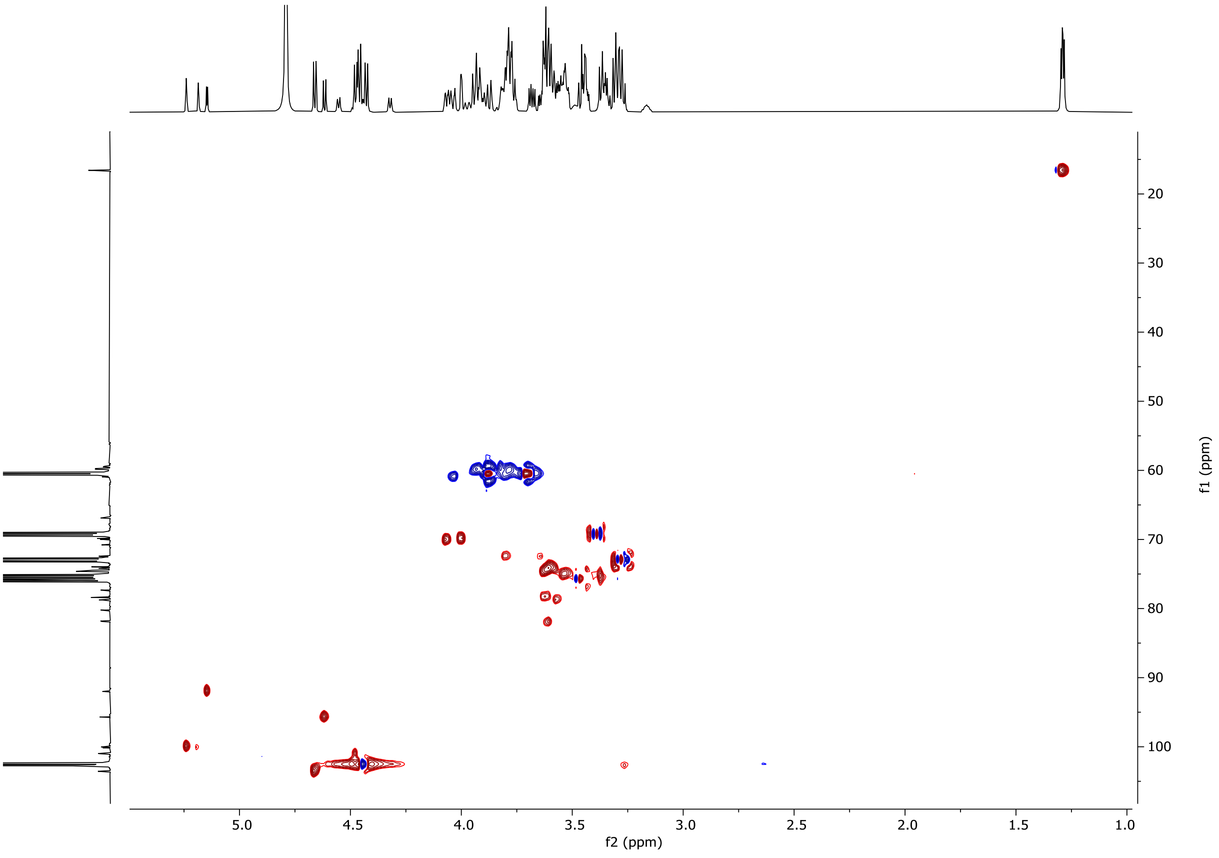

3.6.10 13mer-III-F

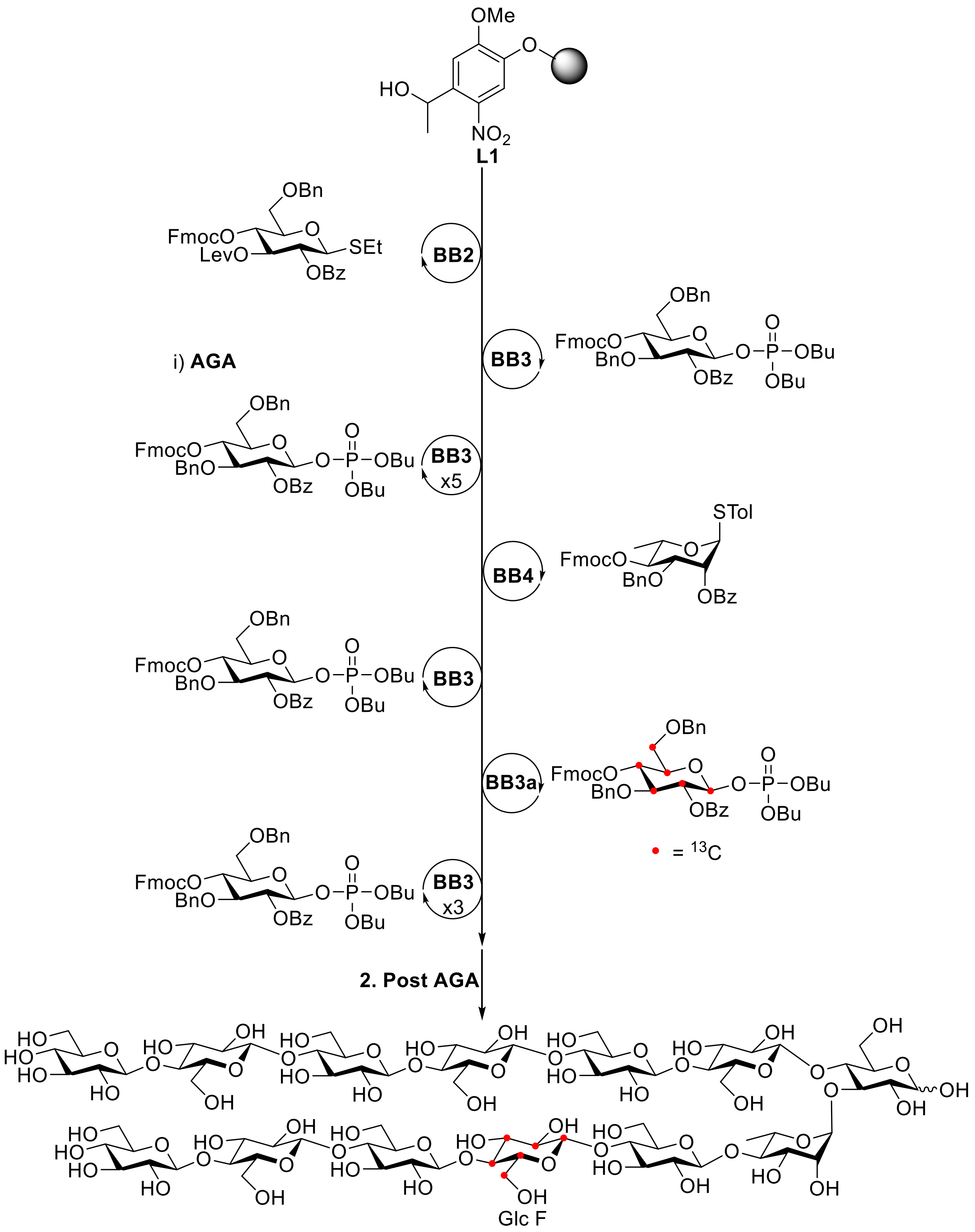

13mer-III-F

Step	BB	Modules	Notes
	-	**A**	**L1** swelling
	**BB2**	**B**, **C1**, **D**, **E1**	**C1**: (**BB2**, -20 °C for 5 min, 0 °C for 20 min)
	**BB3**	**B**, **C3***, **D**, **E1**	**C3***: (**BB3**, -30 °C for 5 min, -10 °C for 40 min) *Double cycle
	(**BB3**)x5	(**B**, **C3**, **D**, **E1**)x5 **D**, **E2**	**C3**: (**BB3**, -30 °C for 5 min, -10 °C for 40 min)
AGA	**BB4**	**B**, **C1**, **D**, **E1**	**C1**: (**BB4**, -20 °C for 5 min, 0 °C for 20 min)
	**BB3**	**B**, **C3***, **D**, **E1**	**C3***: (**BB3**, -30 °C for 5 min, -10 °C for 40 min) *Double cycle
	**^{13}C-BB3a**	**B, C3*, B, C2, D, E1**	**C3***: (**BB3a**, -30 °C for 5 min, -10 °C for 40 min) *Double cycle
	(**BB3**)x3	**(B**, **C3**, **D**, **E1)**x3	**C3**: (**BB3**, -30 °C for 5 min, -10 °C for 40 min)
Post-AGA	-	**G, H1, I1, J**	**G**: (24 h)<br>**I1**: (13 h)<br>**J**: (Method B2: 23.7)

Automated synthesis, global deprotection, and purification afforded **13mer-III-F** as a white solid (5.2 mg, 20% overall yield).

^{1}H NMR (700 MHz, D_2O) δ 5.24 (d, *J* = 1.7 Hz, 0.5H, H-1β Rha), 5.18 (d, *J* = 1.7 Hz, 0.5H, H-1α Rha), 5.14 (d, *J* = 3.7 Hz, 0.4H, H-1α Glc), 4.66 (d, *J* = 7.9 Hz, 1H, H-1 Glc), 4.61 (d, *J* = 7.9 Hz, 0.6H, H-1β Glc), 4.58 (d, *J* = 7.7 Hz, 0.5H, H-1 ^{13}C-Glc), 4.50 – 4.42 (m, 10H, 9x H-1 Glc, H-5 Rha), 4.35 (d, *J* = 7.8 Hz, 0.5H, H-1 ^{13}C-Glc), 4.08 – 4.01 (m, 2.5H), 4.01 – 3.98 (m, 1H), 3.93 (tq, *J* = 14.0, 4.5 Hz, 9H), 3.87 (dd, *J* = 12.5, 2.2 Hz, 2.5), 3.78 (dt, *J* = 12.9, 3.7 Hz, 11H), 3.69 (dd, *J* = 12.4, 5.8 Hz, 4H), 3.65 – 3.56 (m, 23H), 3.55 – 3.50 (m, 3H), 3.48 – 3.42 (m, 6H), 3.38 – 3.35 (m, 2H), 3.34 (d, *J* = 2.6 Hz, 0.5H), 3.33 – 3.25 (m, 10H), 3.21 (d, *J* = 7.0 Hz, 0.5H), 1.29 (dd, *J* = 6.3, 4.1 Hz, 3H, CH_3-6 Rha). ^{13}C NMR (176 MHz, D_2O) δ 103.57, 102.78, 102.39, δ 102.38 (d, *J* = 5.2 Hz), 102.36, δ 102.36 (d, *J* = 46.9 Hz), 102.10, 101.00 (d, *J* = 4.8 Hz), 100.14, 100.02, 95.70, 81.81, 80.26, 80.23 (d, *J* = 10.1 Hz), 80.20, 78.63, 78.33, 78.19 (t, *J* = 40.2 Hz), 77.88, 77.31, 75.88, 75.36, 75.34 (d, *J* = 8.4 Hz), 75.31, 75.15 (d, *J* = 8.1 Hz), 74.93, 74.62 (t, *J* = 42.1 Hz), 74.45, 74.21, 73.98, 73.87 (t, *J* = 39.0 Hz), 73.10 – 72.41 (m), 70.77, 69.86, 69.33, 69.98, 66.86 (d, *J* = 9.7 Hz),60.97, 60.45, 59.83, 59.80 (d, *J* = 42.4 Hz), 59.60, 16.56 (d, *J* = 3.2 Hz). ESI-HRMS m/z 2137.713 $[M+Na]^+$ ($C_{72}{}^{13}C_6H_{132}O_{65}Na$ requires 2137.712).

RP-HPLC of 13mer-III-F (ELSD trace, Method B1, t_R = 24.5 min)

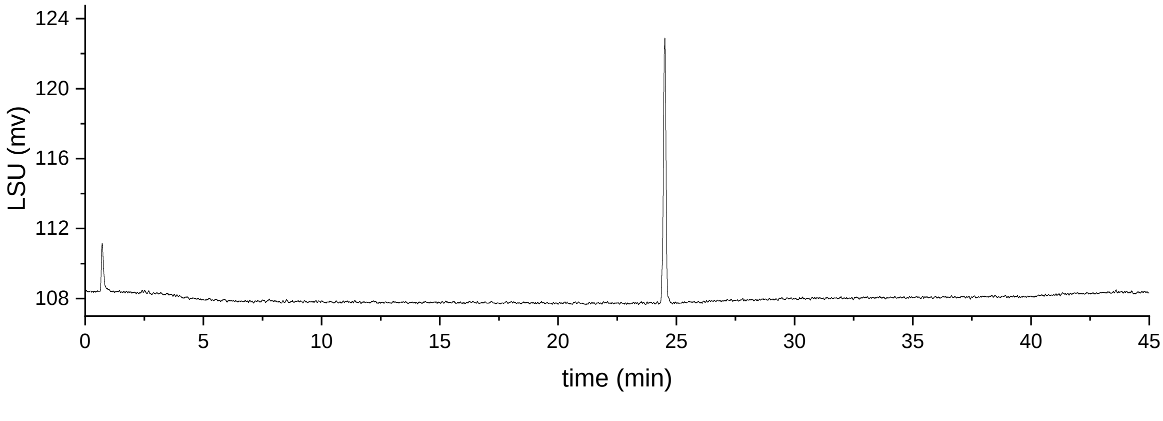

^{1}H NMR of 13mer-III-F (700 MHz, D_2O)

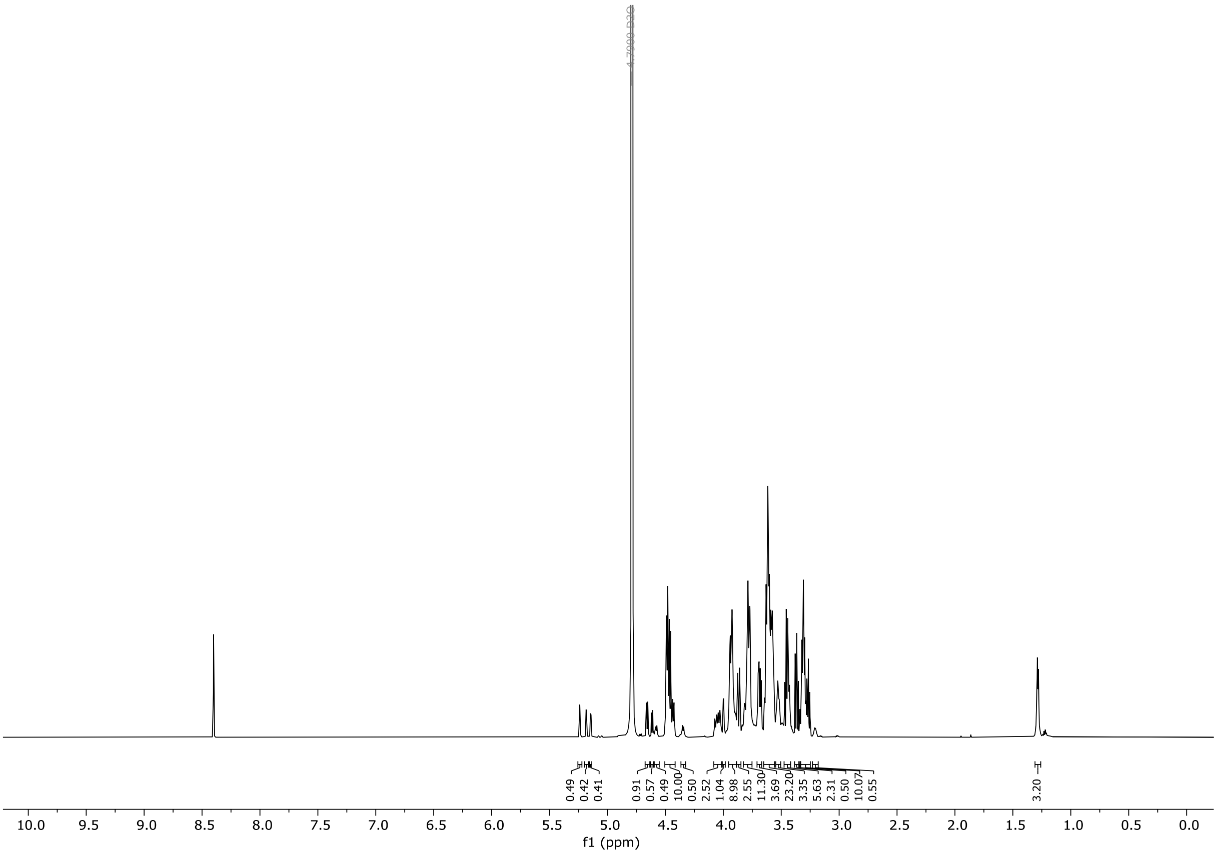

^{13}C NMR of 13mer-III-F (176 MHz, D_2O)

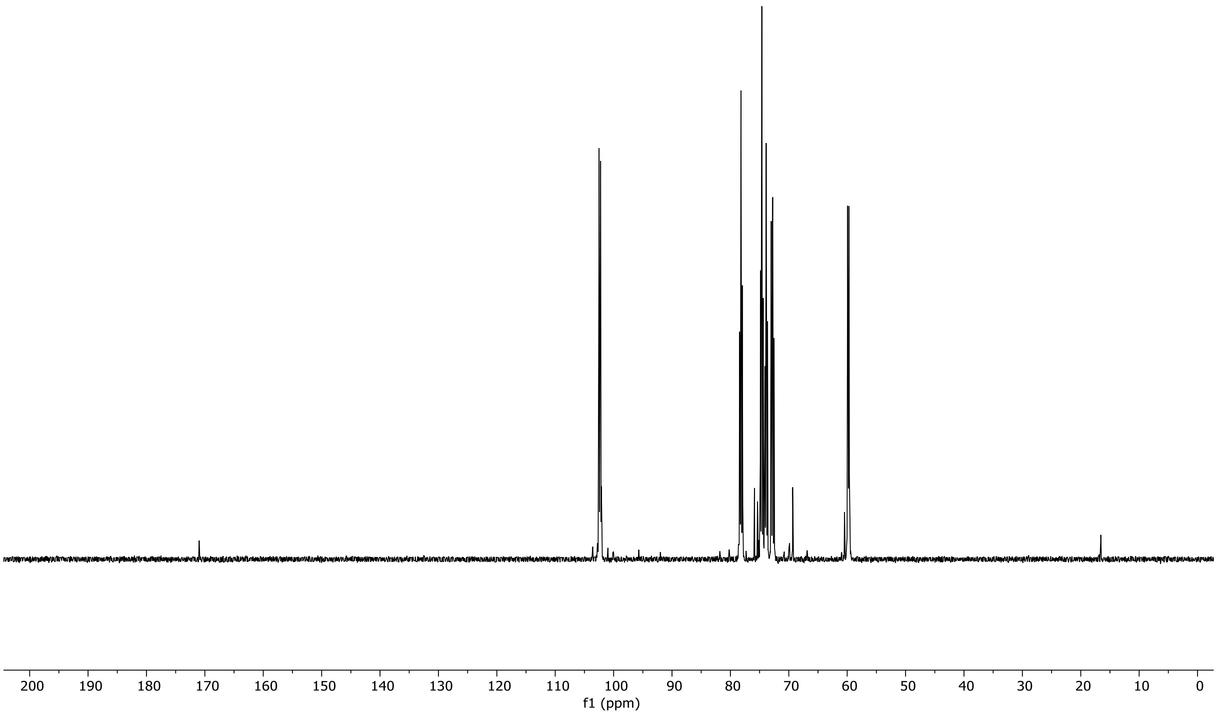

COSY NMR of 13mer-III-F (D_2O)

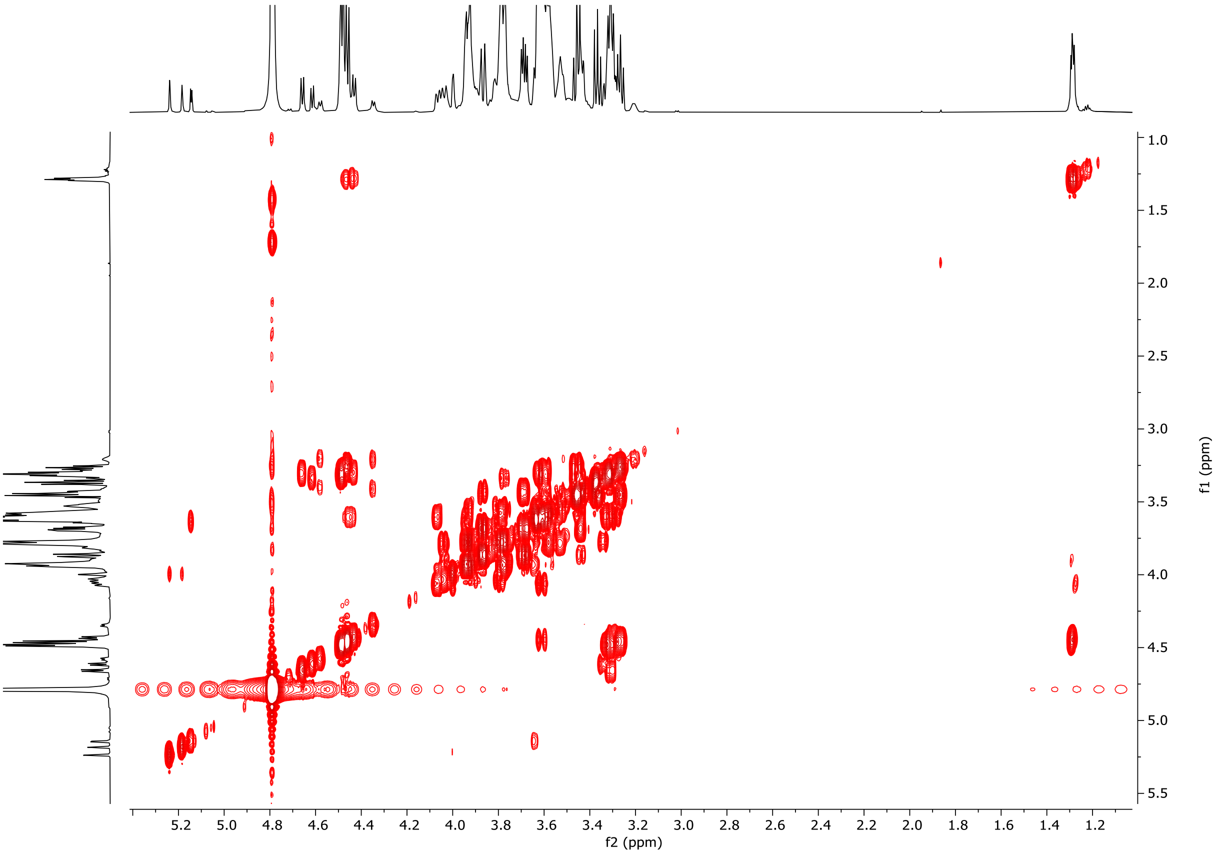

HSQC NMR of 13mer-III-F (D_2O)

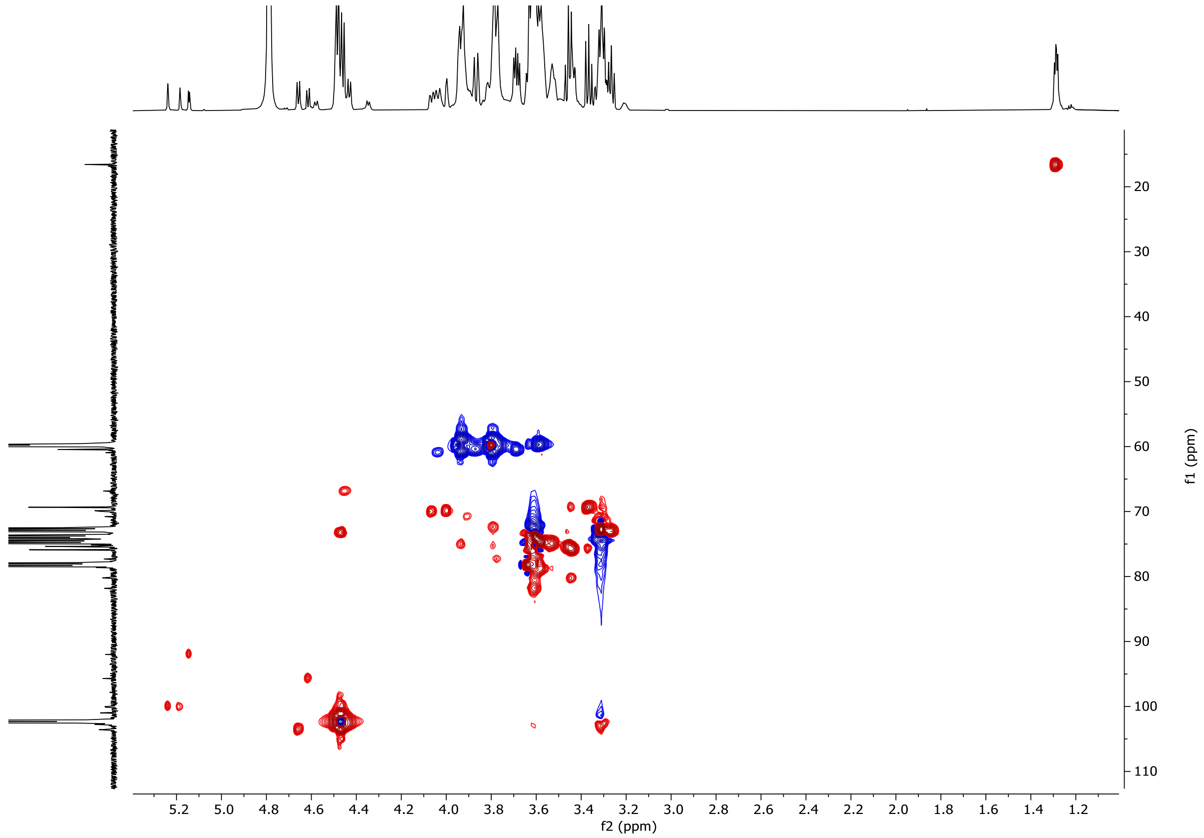

4 Structural analysis

4.1 General materials and methods for Molecular dynamics simulations

For all simulations, the modified version GLYCAM06$_{OSMO,r14}$ force field was used.[12,13]. Initial conformations for single hairpin simulations were constructed with the Glycam Carbohydrate builder and tleap (https://glycam.org/). All compounds with free reducing end were modelled as beta anomers unless specified in the compound name. The topology was subsequently converted using the python script acpype. All simulations were performed in water as solvent using TIP5P as water model.[16] The simulation time for the single molecule experiments was 500 ns unless otherwise specified. Bonds involving hydrogens were constrained using the LINCS to allow a 2 fs time steps. Non-bonded interactions were cut-off at 1.4 nm, long range electrostatics were calculated using the particle mesh Ewald method.[17] After energy minimization (steepest descent algorithm) and before the production run, the systems were equilibrated at 300 K for 50 ns in a canonical (NVT) ensemble (constant number of particles, volume and temperature) and subsequently at 300 K and 1 bar for 50 ns in an isothermal-isobaric (NPT) ensemble. All molecular dynamics simulations were performed using Gromacs 5.1.2.[18] A Nosé-Hoover thermostat[19] kept the constant temperature of 303 K constant while a Parrinello-Rahman barostat[20] ensured a constant pressure of 1 bar. The analysis was visualized using OriginPro 2021b. All hairpins with the free reducing end were modeled as β-anomers unless otherwise specified.

4.2 General materials and methods for NMR

^{1}H, ^{13}C, HSQC, 1D and 2D TOCSY, 1D and 2D ROESY, 2D NOESY NMR spectra were recorded on a Varian 400-MR (400 MHz), Varian 600-NMR (600 MHz), Bruker Biospin AVANCE700 (700 MHz) Bruker AVANCE III 800 (800 MHz) spectrometer. Samples were prepared by dissolving lyophilized samples in D_2O (concentration ≈ 1 - 6 mM). Proton resonances of the oligosaccharides were assigned using a combination of ^{1}H, 2D COSY, HSQC, 1D and 2D TOCSY. Selective 1D TOCSY (HOHAHA, pulse program: seldigpzs) spectra were recorded using different mixing times to assign all the resonances (d9 = 40, 80, 120, 160, and 200 ms). 2D TOCSY (pulse program: mlevphpp) spectra were recorded using different mixing times (d9 = 80, or 120 ms). Selective 1D t-ROESY (pulse program: selrogp.2) spectra were recorded using different mixing times (p15 = 100, 200, or 300 ms). 2D t-ROESY (pulse program: reosyph.2) and 2D NOESY (pulse program: noesygpphpp) spectra were recorded using different mixing times (p15 = 100, 200, or 300 ms for ROESY and d8 = 600, 800, or 1000 ms for NOESY). Monosaccharide were named as follows: D-glucose (Glc), D-*N*-acetyl glucosamine (GlcNAc), L-rhamnose (Rha). Labelling of protons in a monosaccharide is done as follows: e.g. proton attached to C-1 of Rha is named "Rha-1". Resonances of residues at the reducing end are additionally labelled with α or β.

4.3 Molecular dynamics simulations

4.3.1 9mers and 6mer-III

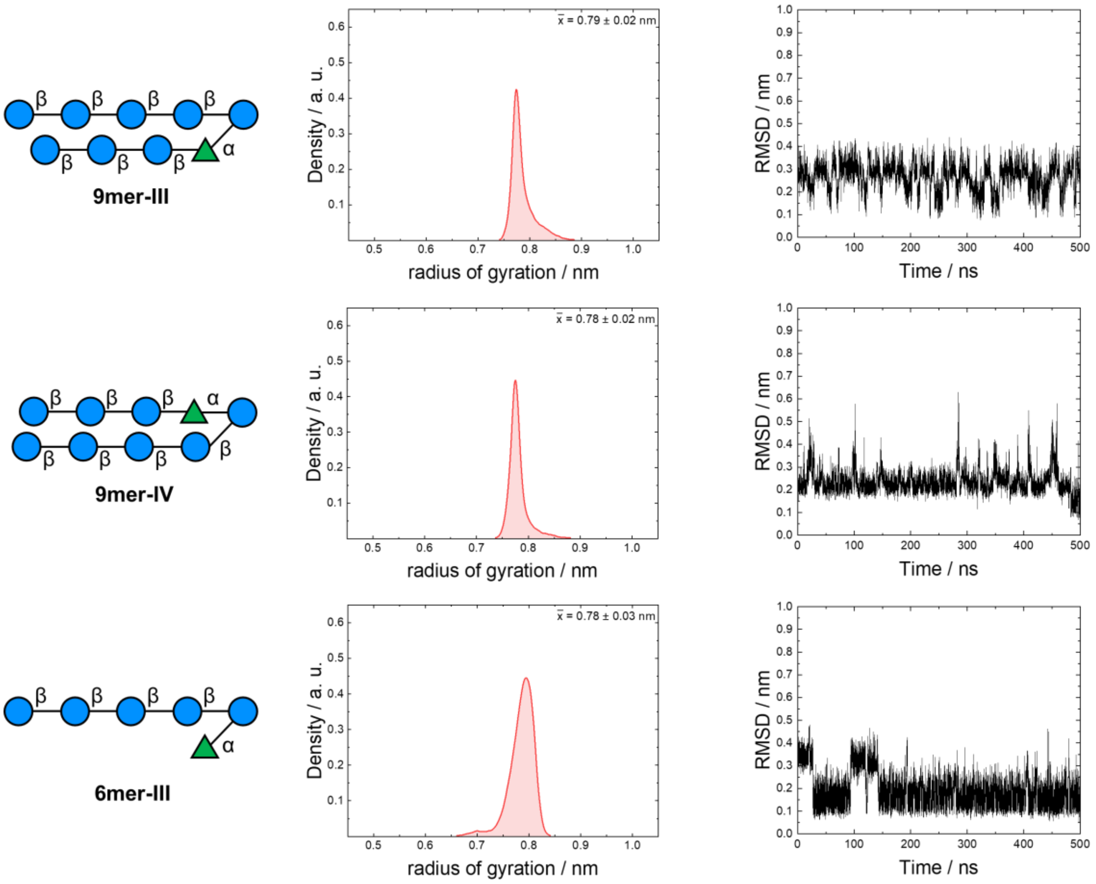

Figure S7 Radius of gyration and root-mean-square deviation (RMSD) analysis of **9mer-III**, **9mer-IV**, and **6mer-III**. The comparison with **9mer-I** and **9mer-II** is reported in previous work.[2]

$Definition\ \psi = C_1 - O_n - C_n - C_{n-1}$

$Definition\ \phi = O_5 - C_1 - O_n - C_n$

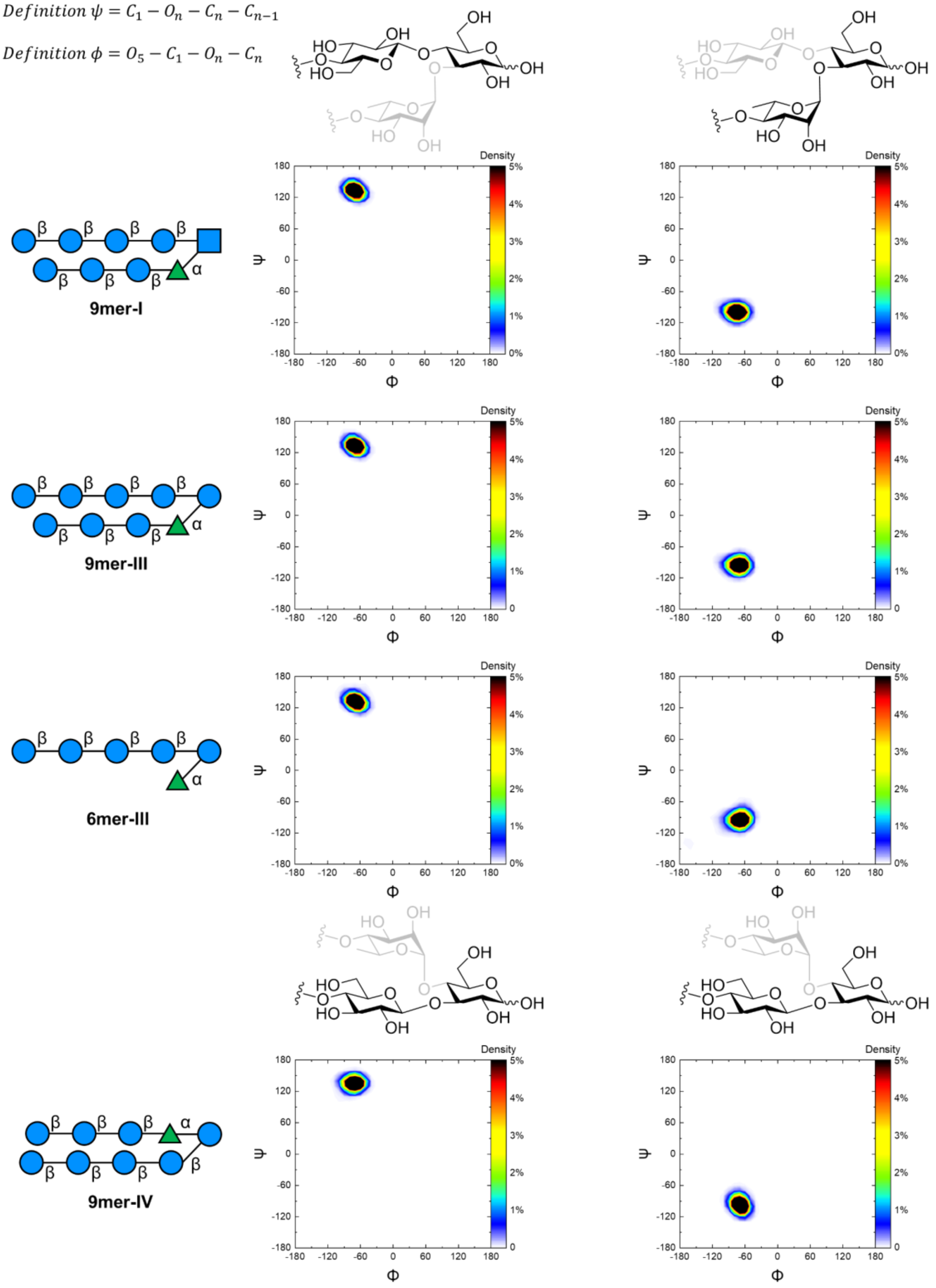

Figure S8 Ramachandran plots of the turn units of **9mer-I**, **9mer-III**, **6mer-III** and **9mer-IV**.

$Definition\ \psi = C_1 - O_n - C_n - H_n$

$Definition\ \phi = H_1 - C_1 - O_n - C_n$

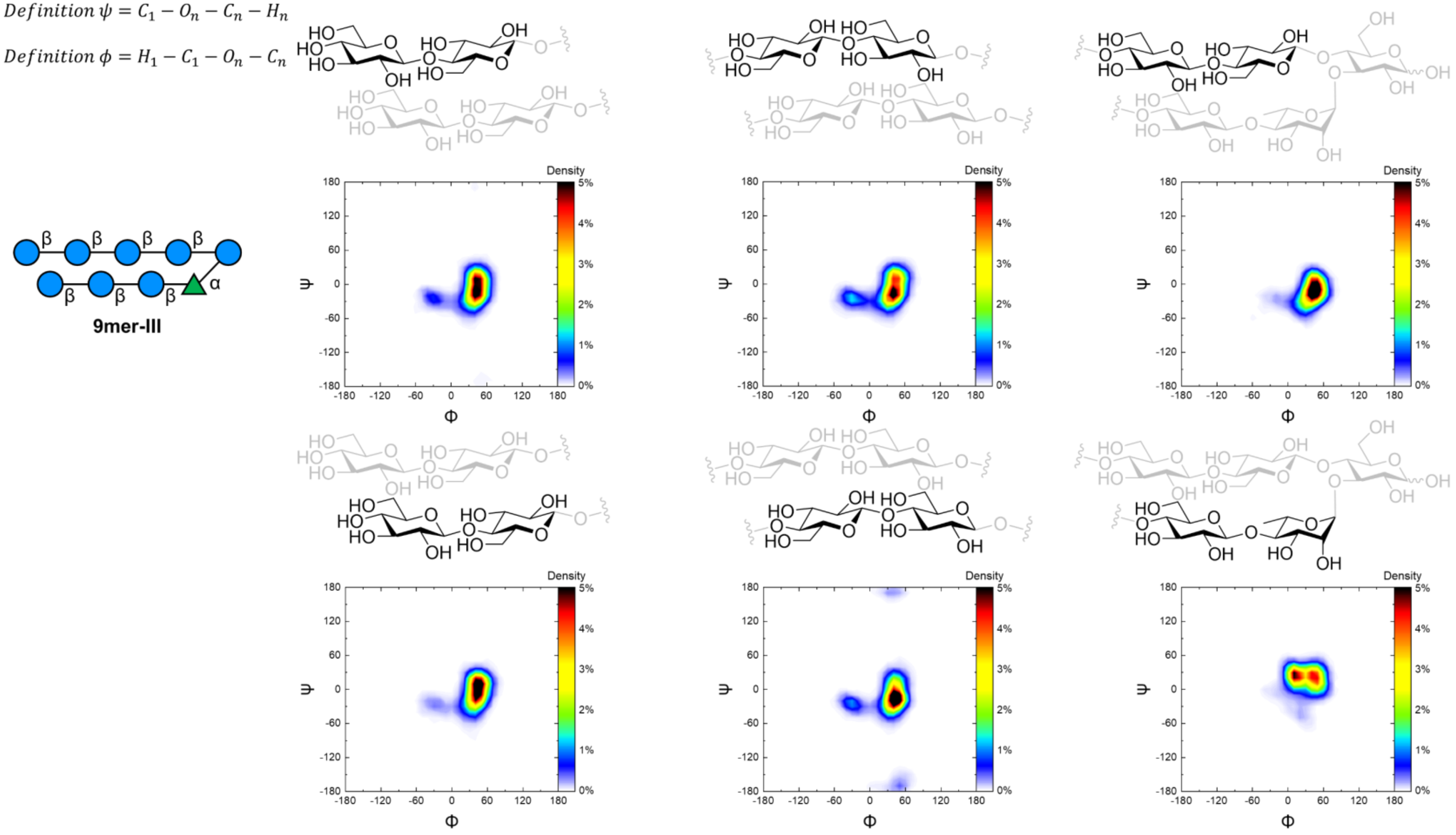

Figure S9 Ramachandran plots of the top and bottom strands of **9mer-III**.

$Definition\ \psi = C_1 - O_n - C_n - H_n$

$Definition\ \phi = H_1 - C_1 - O_n - C_n$

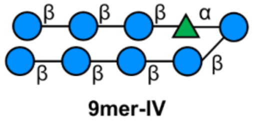

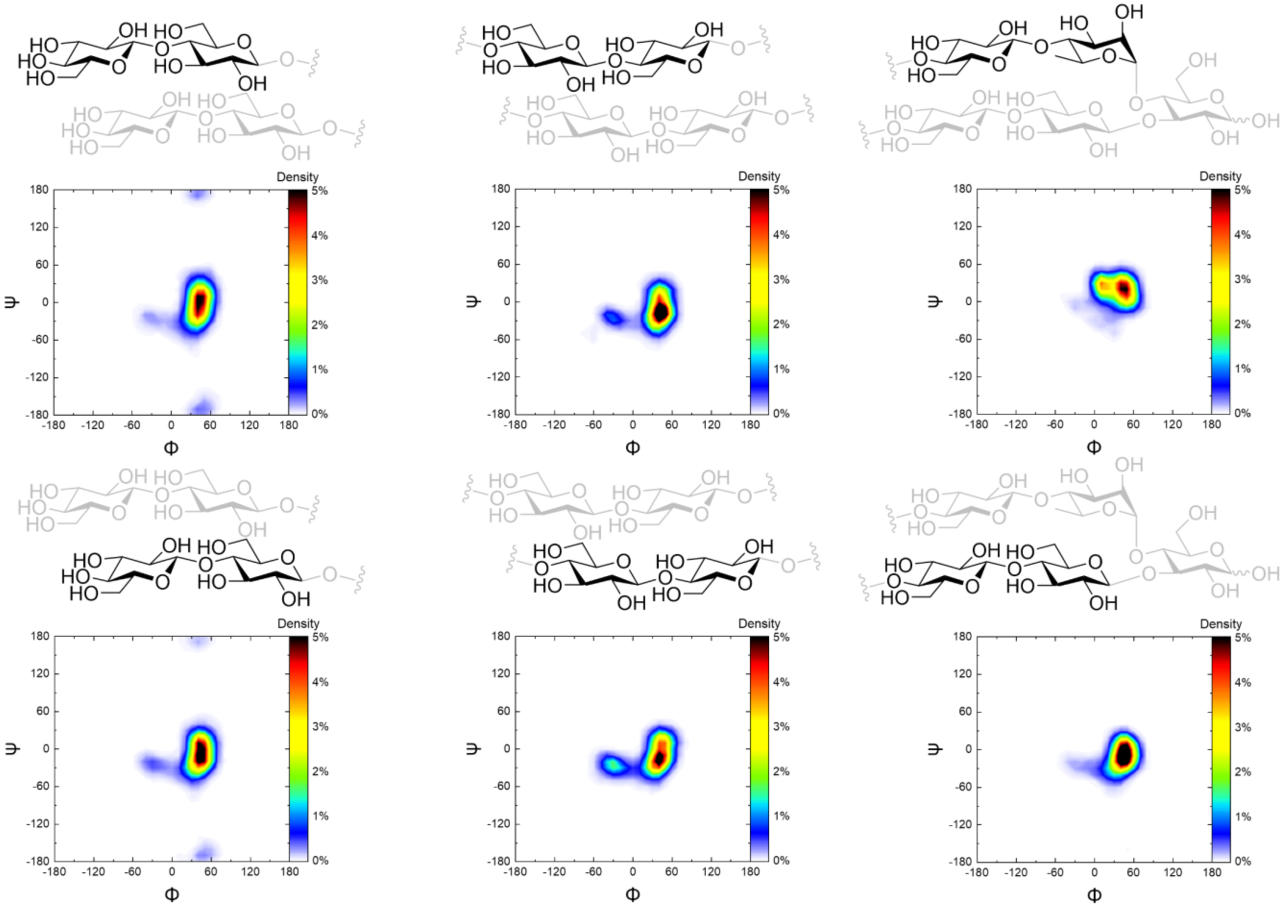

Figure S10 Ramachandran plots of the top and bottom strands of **9mer-IV**.

$Definition\ \psi = C_1 - O_n - C_n - H_n$

$Definition\ \phi = H_1 - C_1 - O_n - C_n$

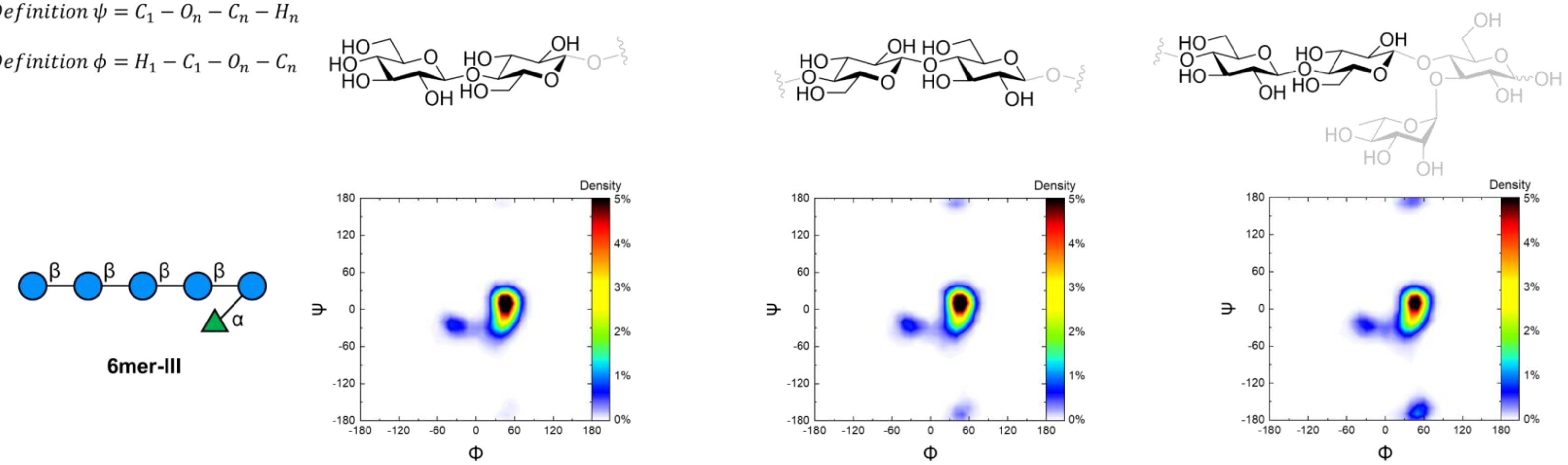

Figure S11 Ramachandran plots of the top strand of **6mer-III**.

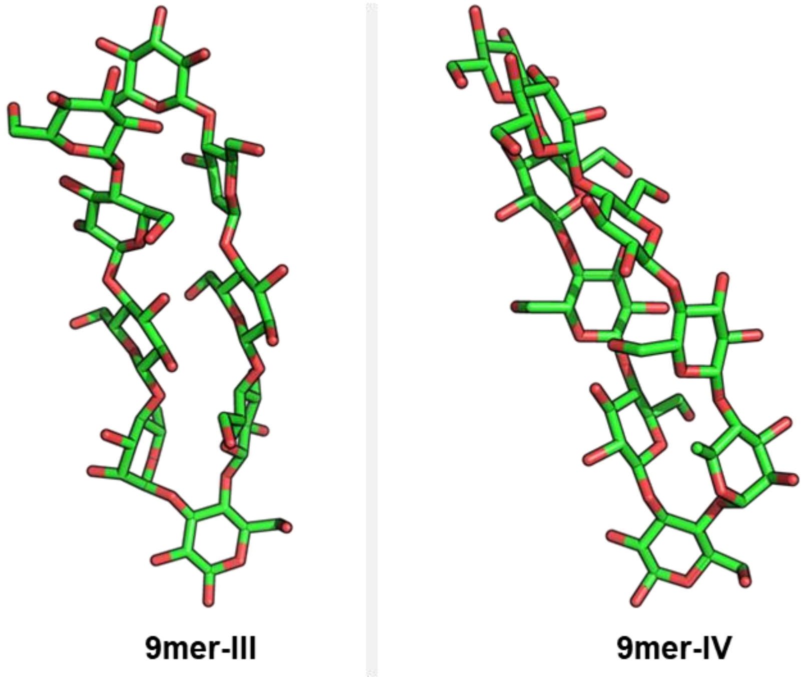

Figure S12 Representative snapshot of **9mer-III** and **9mer-IV**. The twisting of the two strands is correlating with the geometry of the turn unit.

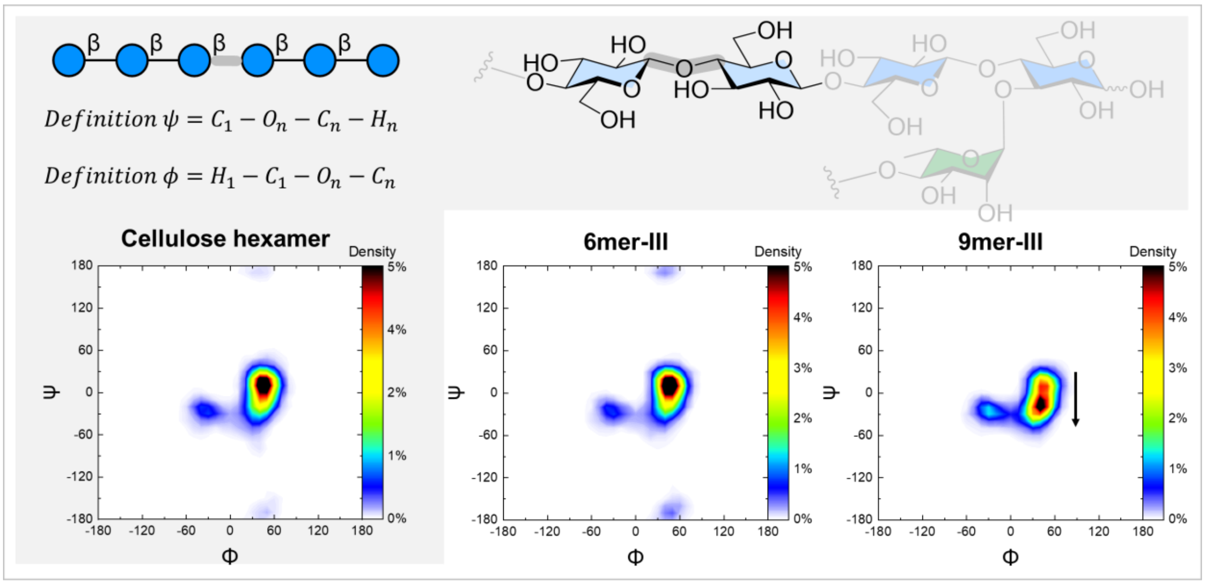

Figure S13 Comparison of Ramachandran plots of highlighted glycosidic linkage of **6mer-III** and **9mer-III** with standard **cellulose hexamer**. In **9mer-III**, a significant shift of Ψ to negative values (indicated by the arrow) suggests an interaction between the two strands.[8]

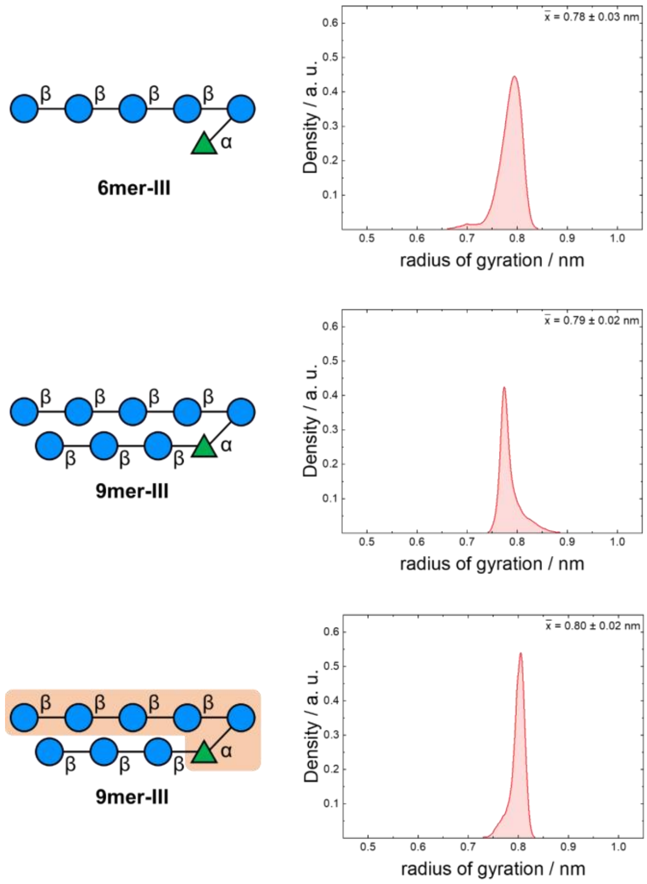

Figure S14 Comparison of the radius of gyration of **6mer-III**, **9mer-III** and the highlighted (orange) part of **9mer-III**. The broader RoG distribution indicates a more flexible structure for the **6mer-III** when compared to **9mer-III**. The average radius is slightly bigger for the **9mer-III** compared to **6mer-III** (when the half hairpin is considered in **9mer-III**, highlighted in orange), indicating that the strand interaction leads to a flattened/elongated conformation.

4.3.2 5mer-III

Dihedral angle analysis as well as the radius of gyration of **5mer-III** showed the appearance of a second population (Figure S15, S16). The root-mean-square deviation indicated a rapid switch between two distinct conformations. Trajectory analysis revealed the opening of the hairpin strands according to a conformational change of the pyranose ring of the first glucose unit (reducing end). The conventional $^{4}C_{1}$ chair conformation (θ = 0°) transitioned to an unconventional $^{3,O}B$ boat conformation (θ = 90°) and ended with the complete inversion of the pyranose ring to a $^{1}C_{4}$ chair conformation (θ = 180°)(Figure S18). The change from an equatorial to axial orientation of the strands resulted in an open conformation of the hairpin. To verify the observed results multiple copies of the simulation with prolonged simulation time were carried out. One copy with restrained puckering coordinates was simulated as reference. The restrained system showed a constant closed conformation along the entire simulation time. Three out of four copies showed the same conformational changes as the initial simulation, resulting in the opening of the hairpin. Longer hairpin structures (e.g., **7mer-III** and longer), do not show any opening events. Previous conformational studies of the natural model **Lewis X** already suggested the occurrence of alternative ring puckering not only in the presents of lectin binding sites (based on data of MD simulations and crystal structures) but also to a small percentage in solution.[9] However, the small percentage of unconventional pyranose conformation made the verification with NMR methods impossible. The comparison of the ^{1}H-NMR spectra of **5mer-I**, **5mer-III** and **9mer-III** showed no significant differences in the coupling constant $J_{H1\text{-}H2}$ of the anomeric proton H1 indicating no difference of the *cis-trans* population of one of the structures (Table S1). Thus, even though we cannot exclude the presence of some unconventional ring conformations, we concluded that those populations could be over emphasized by MD. The presented data of **5mer-III** in the main manuscript (inter residue distances graph) includes the average of all simulations (excluding the restrained reference).

Compound	$J_{H1\alpha-H2}$	$J_{H1\beta-H2}$
**5mer-I**	3.5	8.0
**5mer-III**	3.8	8.0
**9mer-III**	3.7	8.0

Table S1 Comparison of the coupling constant $J_{H1\alpha/\beta-H2}$ of the anomeric proton H-1 extracted from the proton NMR data for **5mer-I** (previous work)[2], **5mer-III** and **9mer-III**.

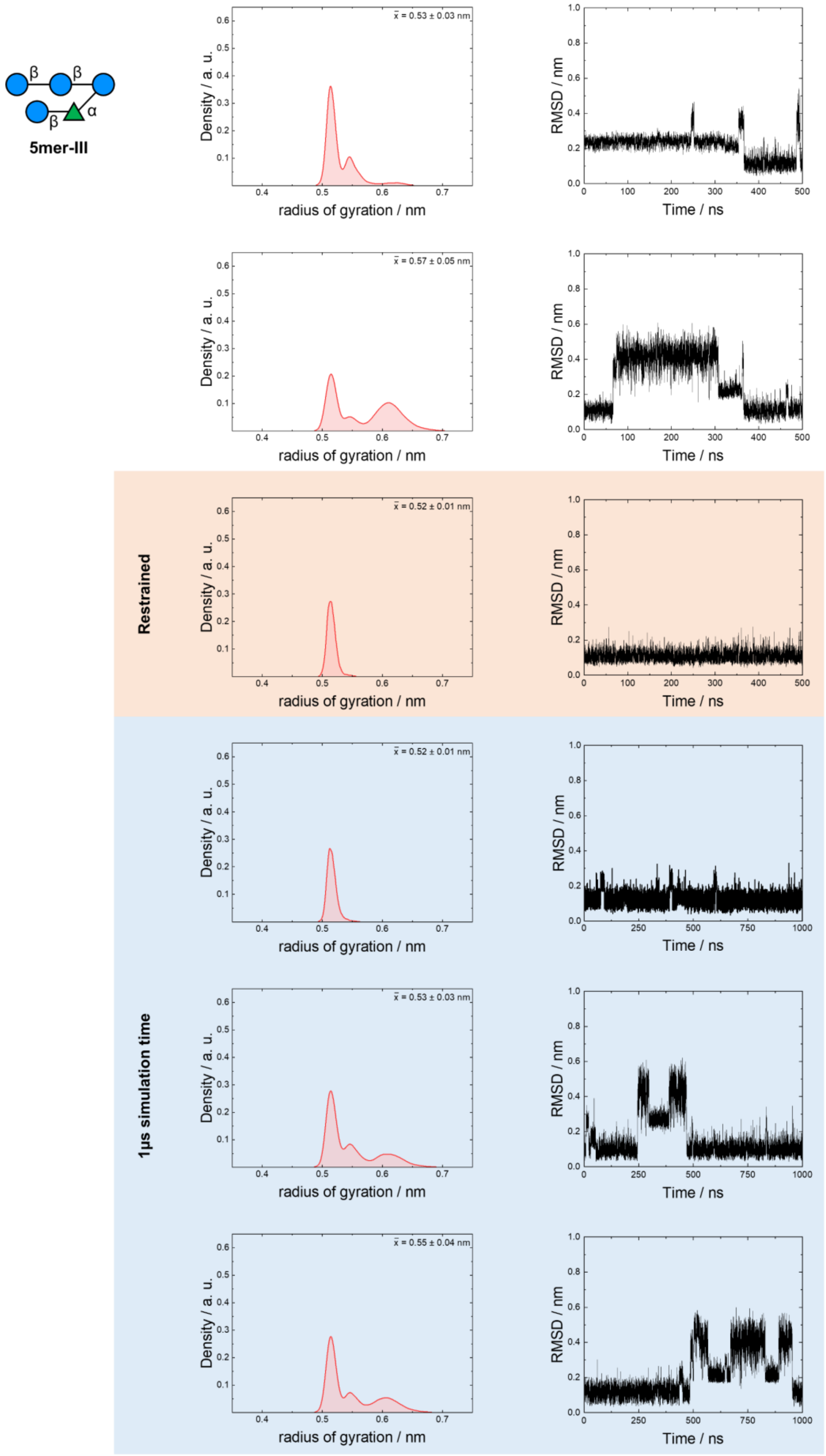

Figure S15 Radius of gyration and root-mean-square deviation (RMSD) analysis of **5mer-III**.

$Definition\ \psi = C_1 - O_n - C_n - C_{n-1}$

$Definition\ \phi = O_5 - C_1 - O_n - C_n$

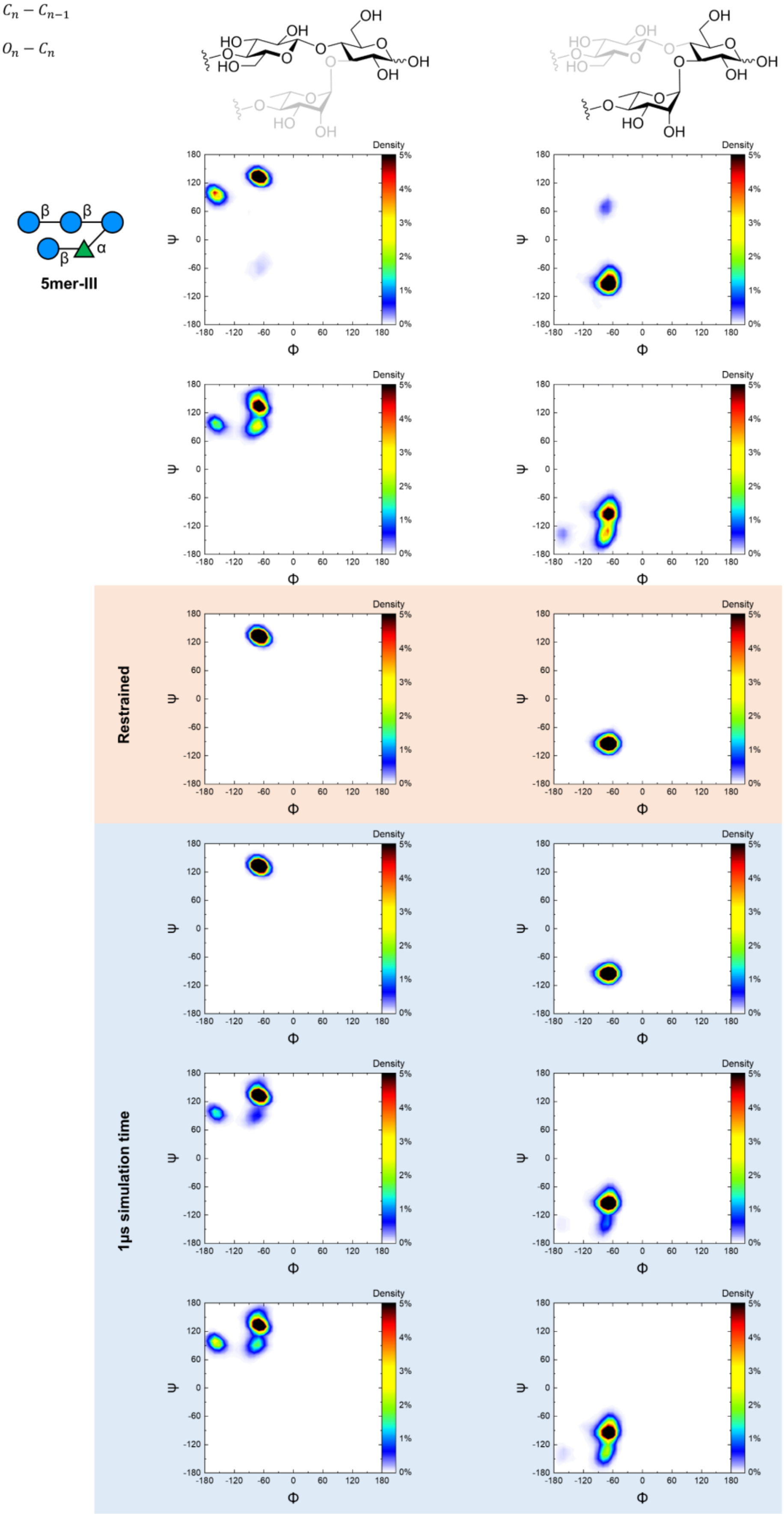

Figure S16 Ramachandran plots of the turn units of **5mer-III**. Multiple simulations were carried out to verify the appearance of unusual conformations.

$Definition\ \psi = C_1 - O_n - C_n - H_n$

$Definition\ \phi = H_1 - C_1 - O_n - C_n$

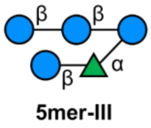

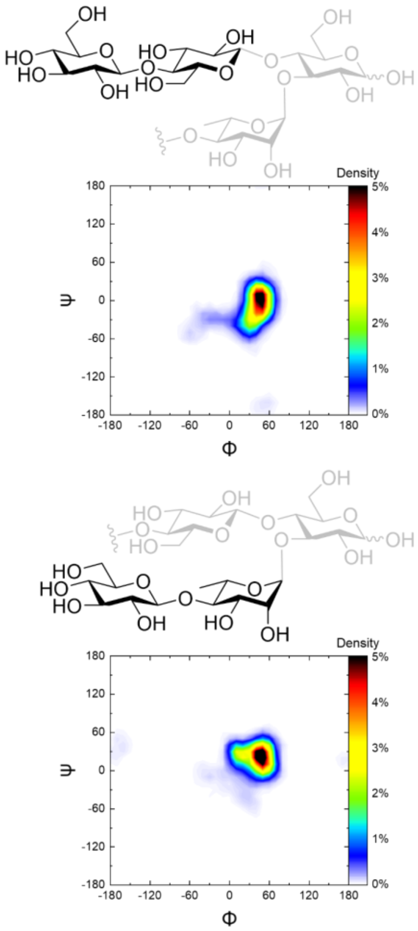

Figure S17 Ramachandran plots of the top and bottom strands of **5mer-III**.

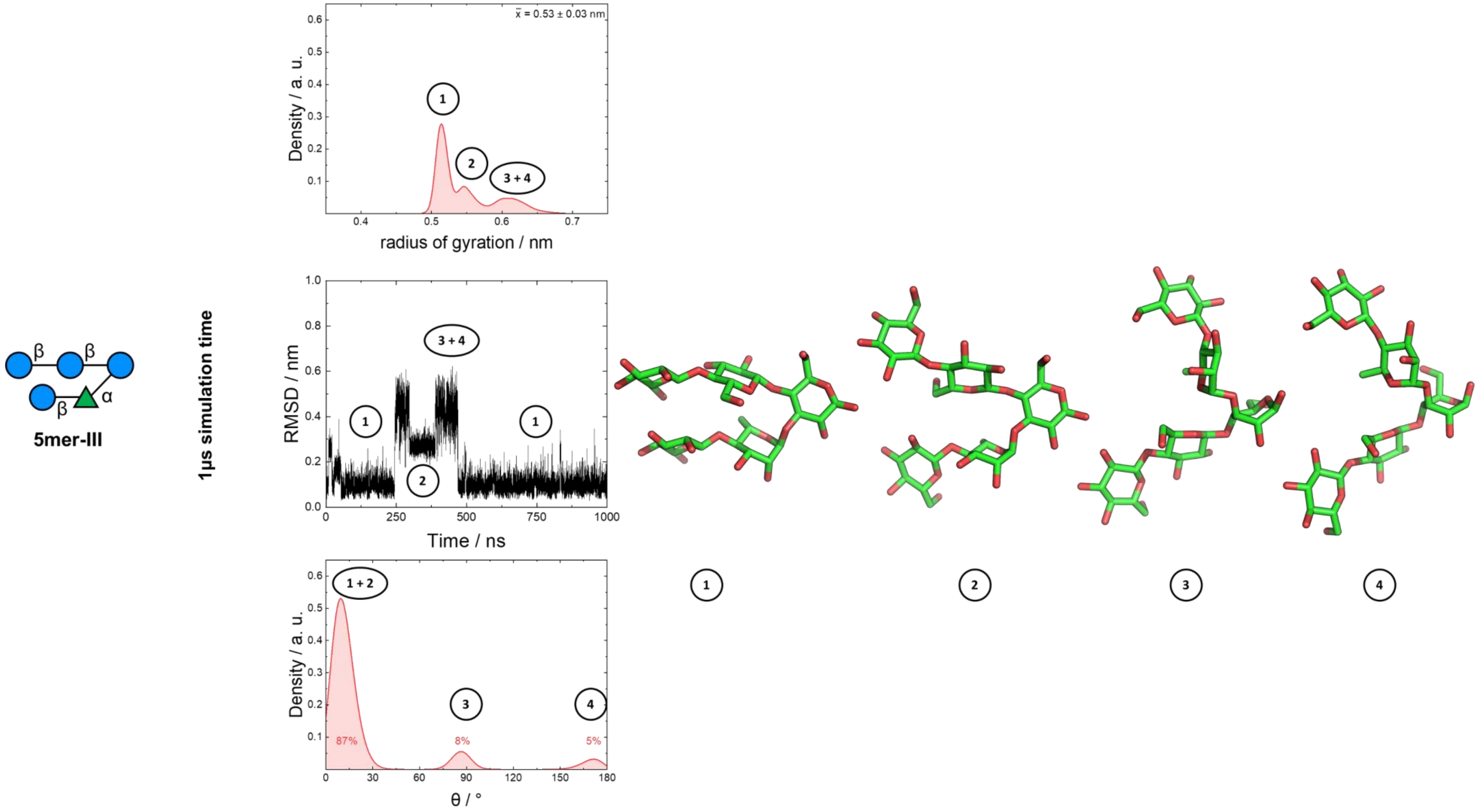

Figure S18 Radius of gyration (top) and root-mean-square deviation (middle) analysis of **5mer-III**. Ring puckering analysis and representative snapshots revealed the correlation between chair inversion and increased flexibility of the system. Conformation **1** and **2** represent the conventional dynamic of the $^{4}C_{1}$ chair, **3** of $^{3,O}B$ boat conformation and **4** of the fully inverted $^{1}C_{4}$ chair.

4.3.3 5mer-III-Closed

$$Definition\ dihedral = O_{top,n} - C_{top,n} - C_{bottom,m} - O_{bottom,m}$$

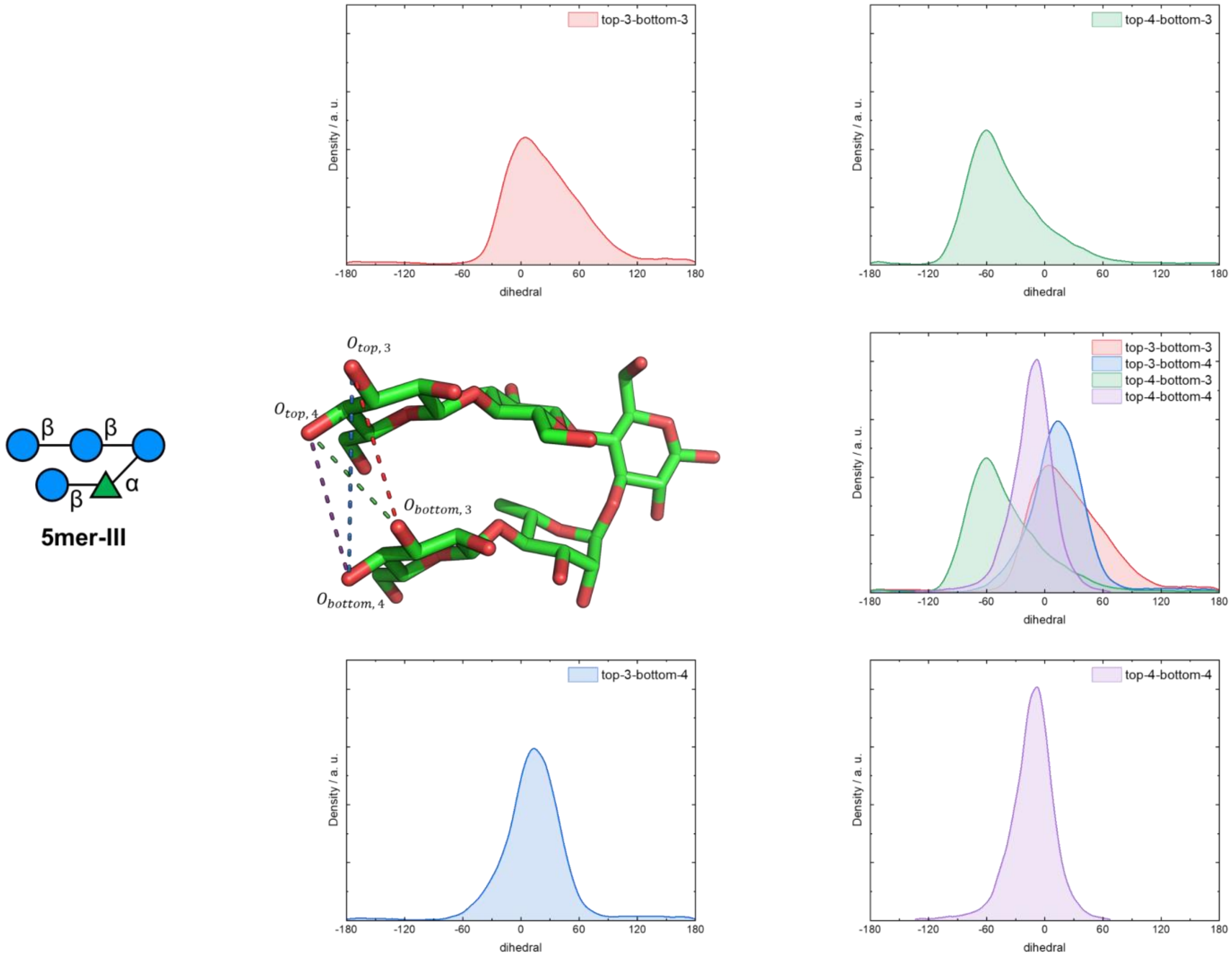

Figure S19 Hairpin closing study of **5mer-III**. The defined dihedral angle revealed the relative orientation of the C-O bonds of the top and bottom strand. Top-4 and bottom-4 showed the sharpest population around 0°, making it the optimal position for the introduction of a linker.

4.3.4 Length comparison

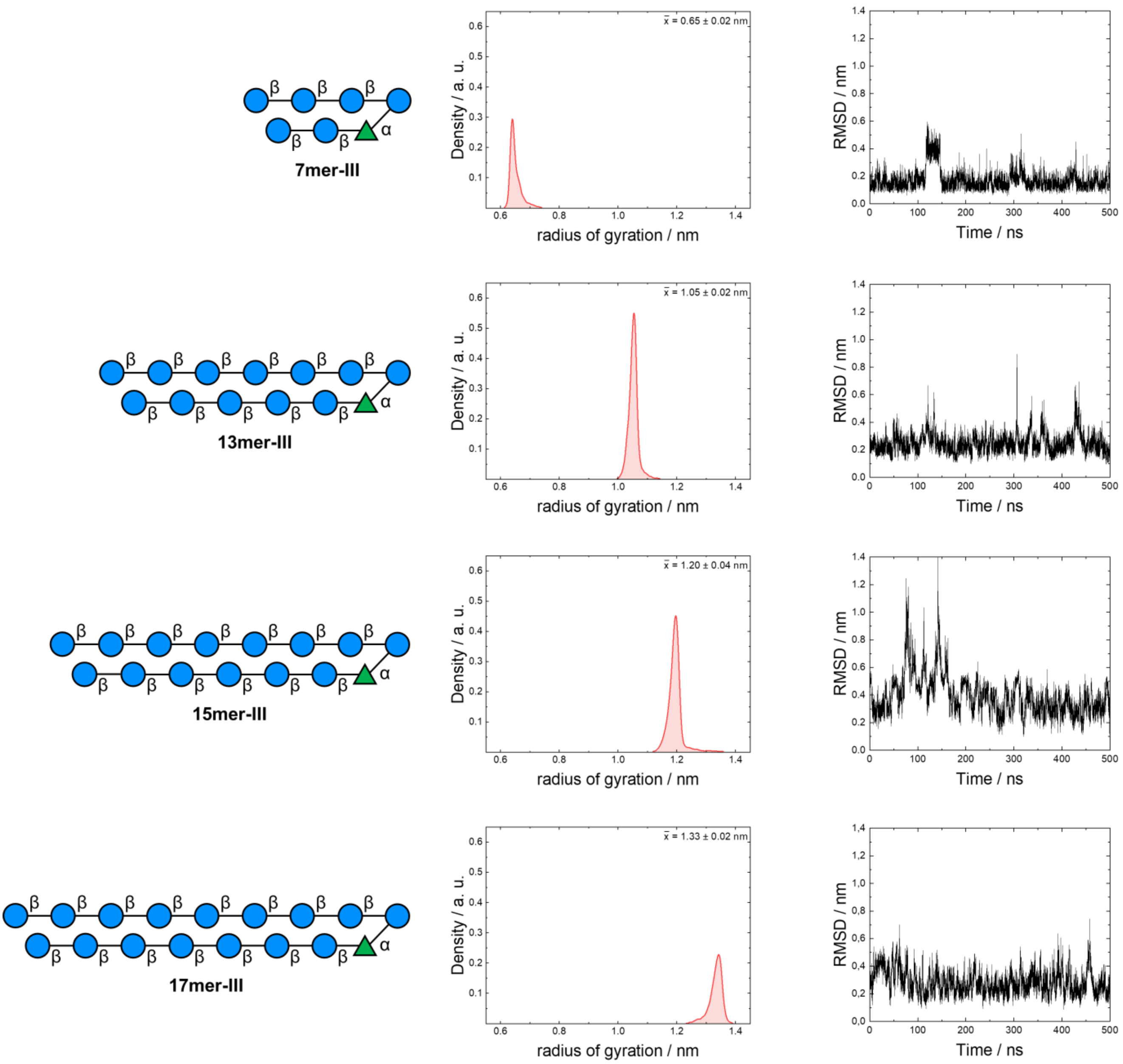

Figure S20 Radius of gyration and root-mean-square deviation (RMSD) analysis of **7mer-III**, **13mer-III**, **15mer-III** and **17mer-III**.

$$Definition\ \psi = C_1 - O_n - C_n - C_{n-1}$$

$$Definition\ \phi = O_5 - C_1 - O_n - C_n$$

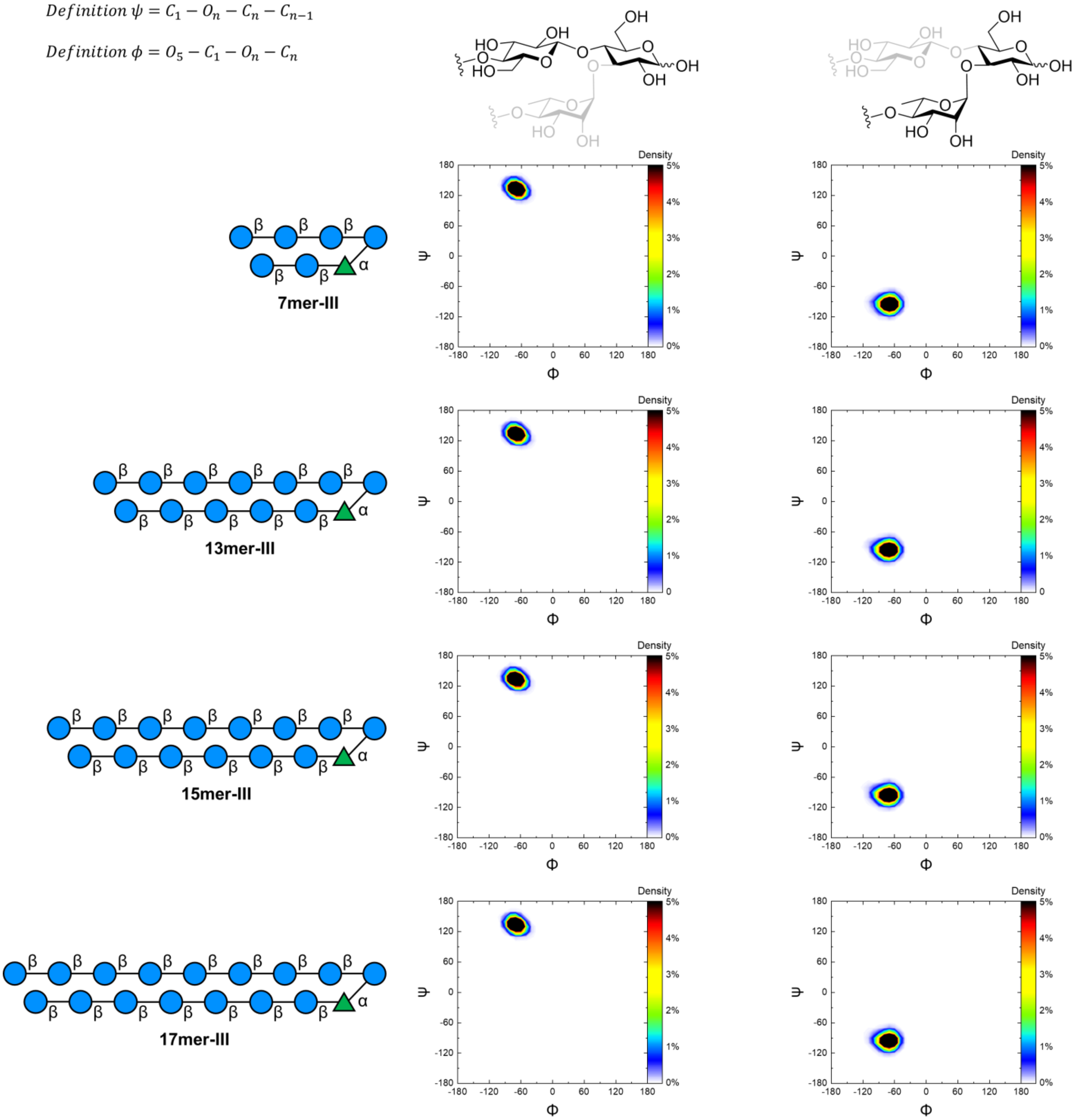

Figure S21 Ramachandran plots of the turn units of **7mer-III**, **13mer-III**, **15mer-III** and **17mer-III**.

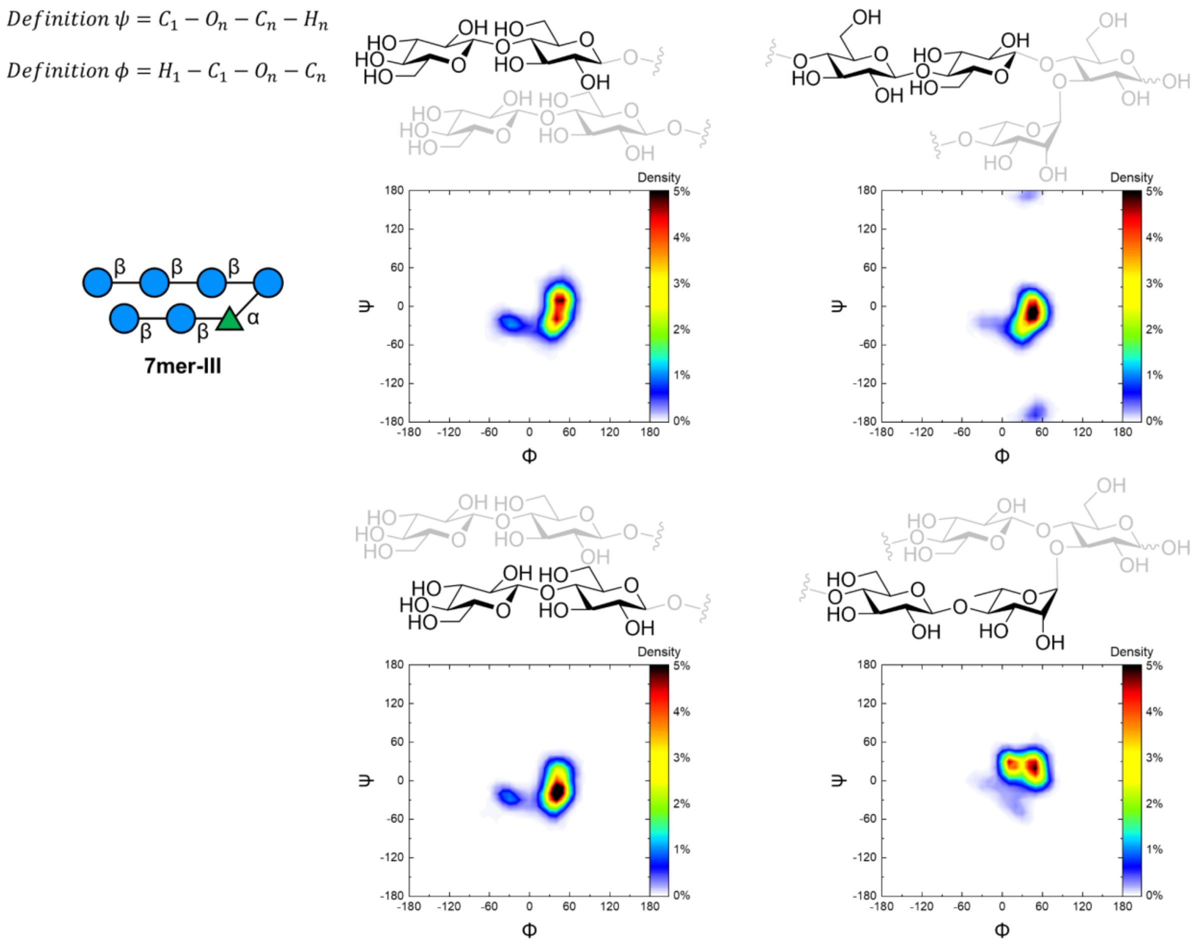

Figure S22 Ramachandran plots of the top and bottom strands of **7mer-III**.

$$Definition\ \psi = C_1 - O_n - C_n - H_n$$

$$Definition\ \phi = H_1 - C_1 - O_n - C_n$$

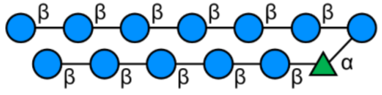

13mer-III

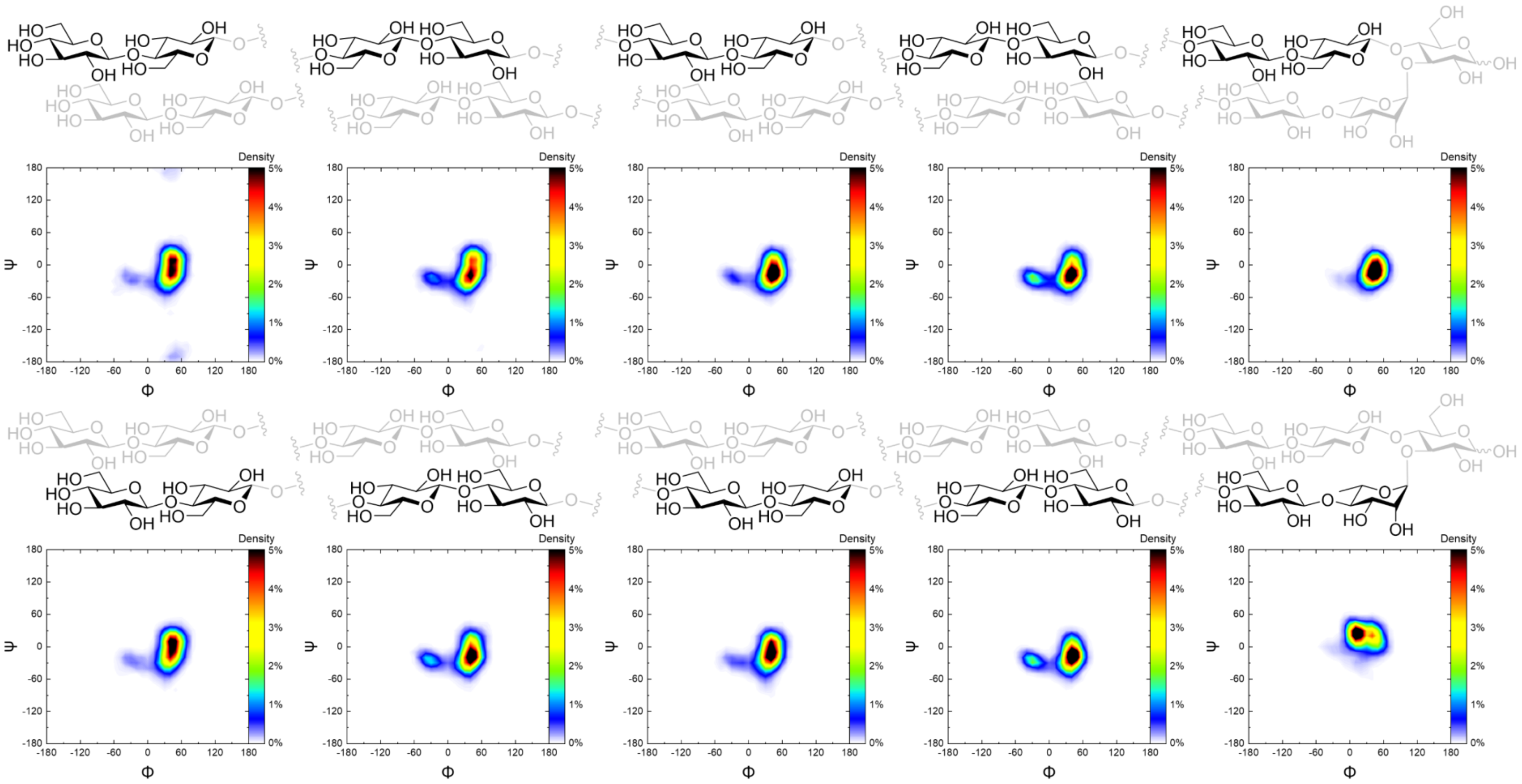

Figure S23 Ramachandran plots of the top and bottom strands of **13mer-III**

4.4 NMR studies

4.4.1 NMR characterization of 5mer-III

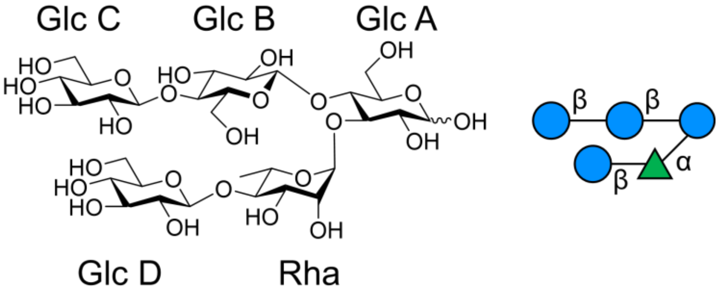

5mer-III

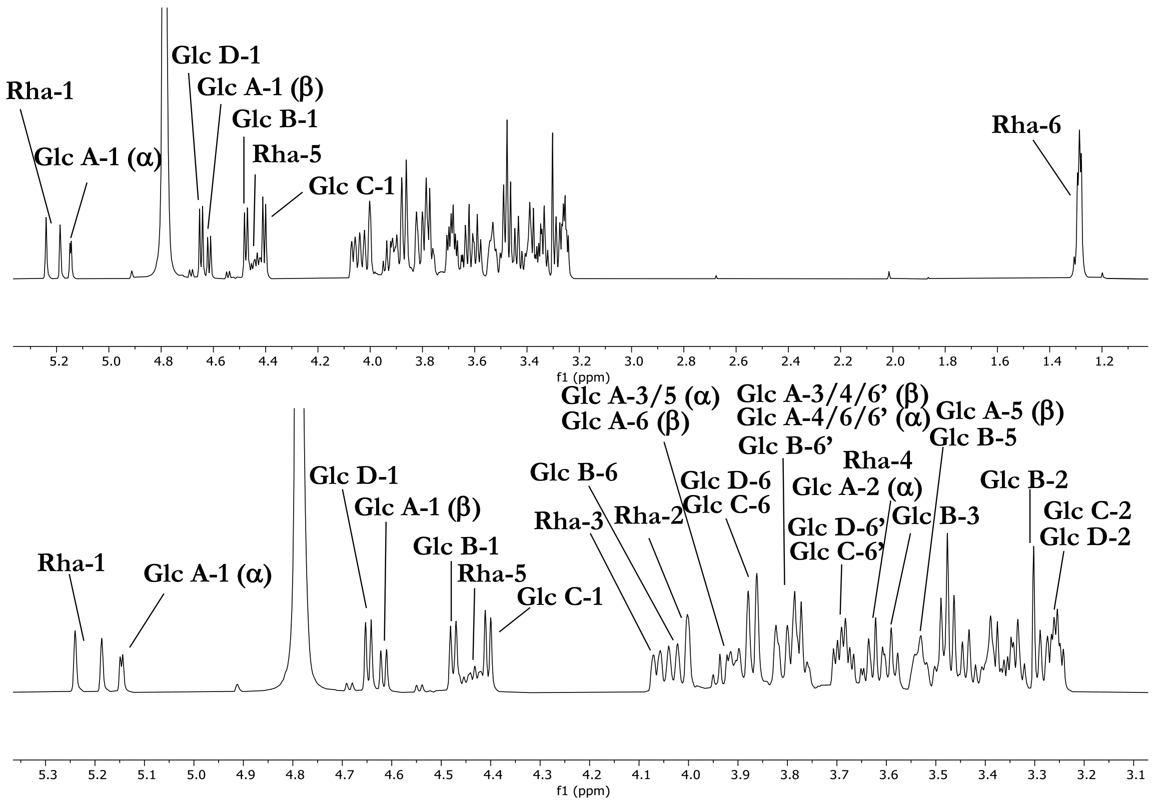

Figure S24 ^{1}H NMR (700 MHz, D_2O) of **5mer-III** with assignments.

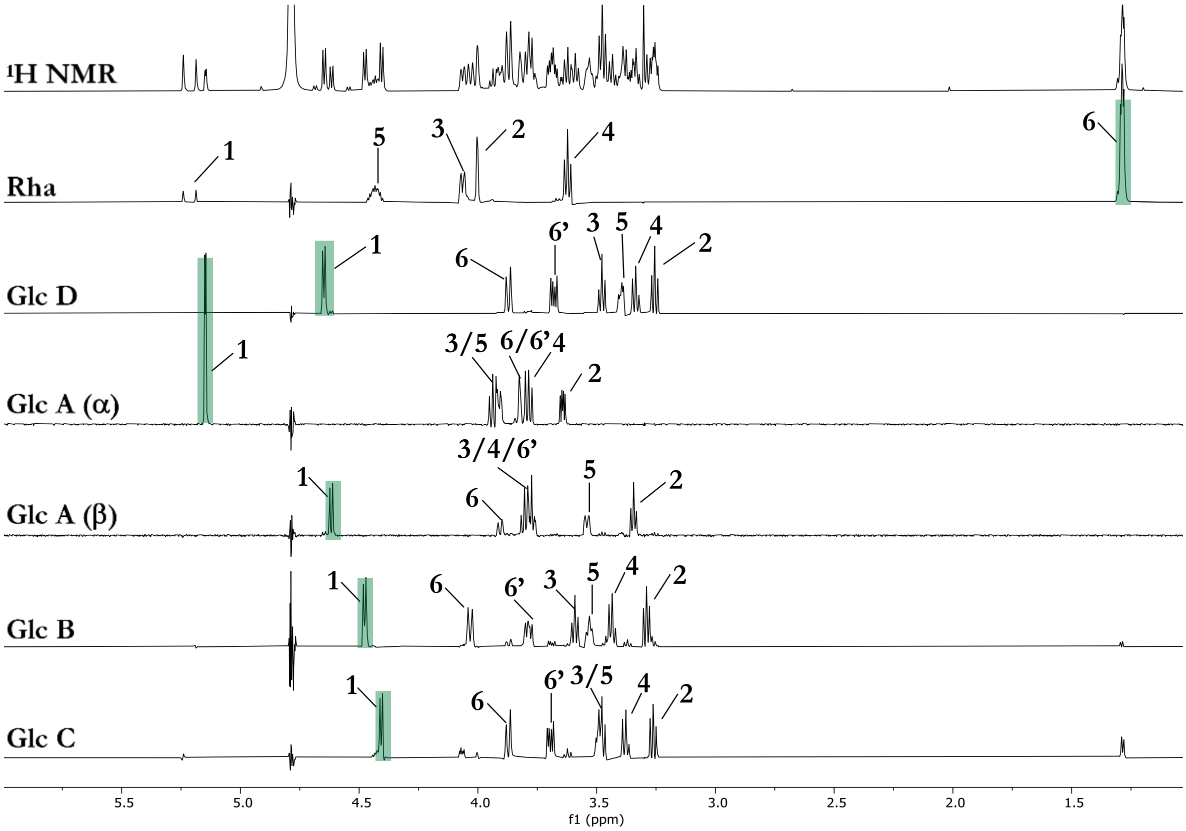

Figure S25 1D TOCSY (700 MHz, d9 200 ms, D_2O) of **5mer-III** with assignments. Resonances chosen for selective excitation are highlighted in green.

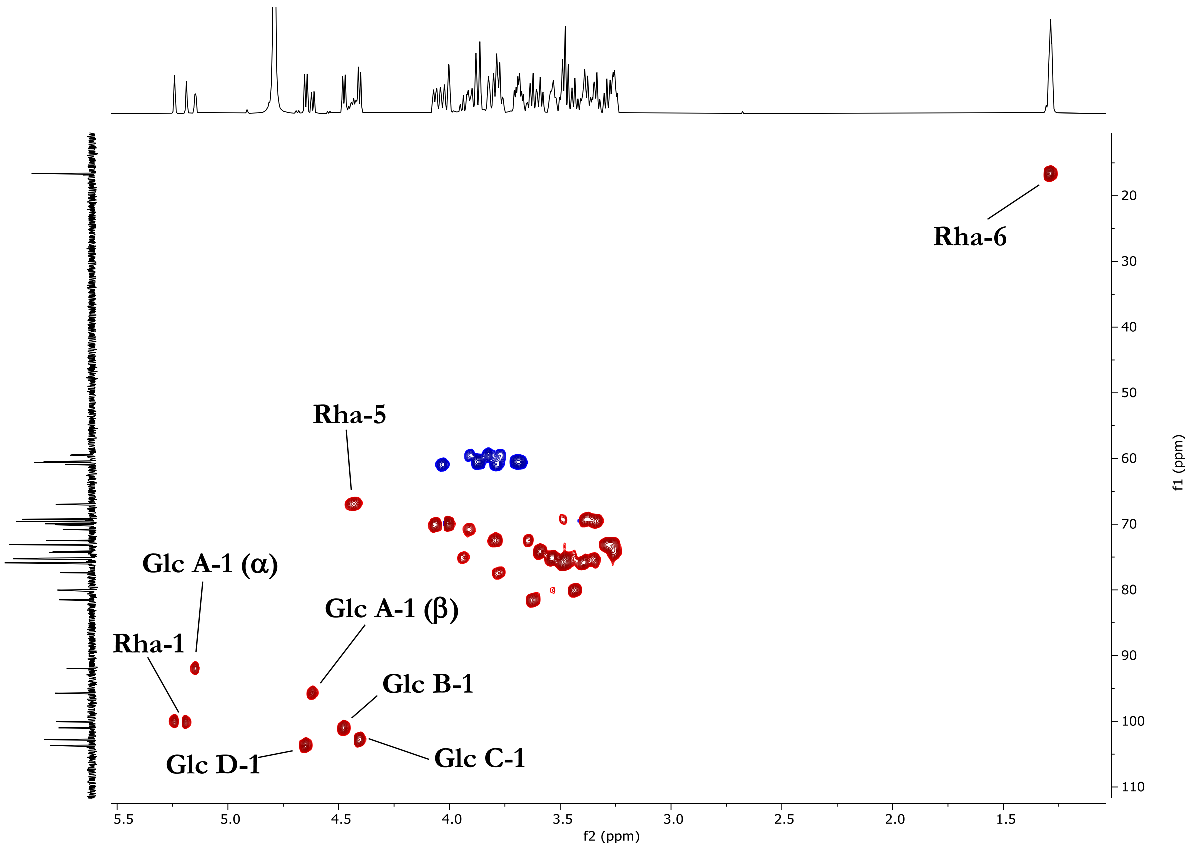

Figure S26 HSQC NMR (D_2O) of **5mer-III** with assignments.

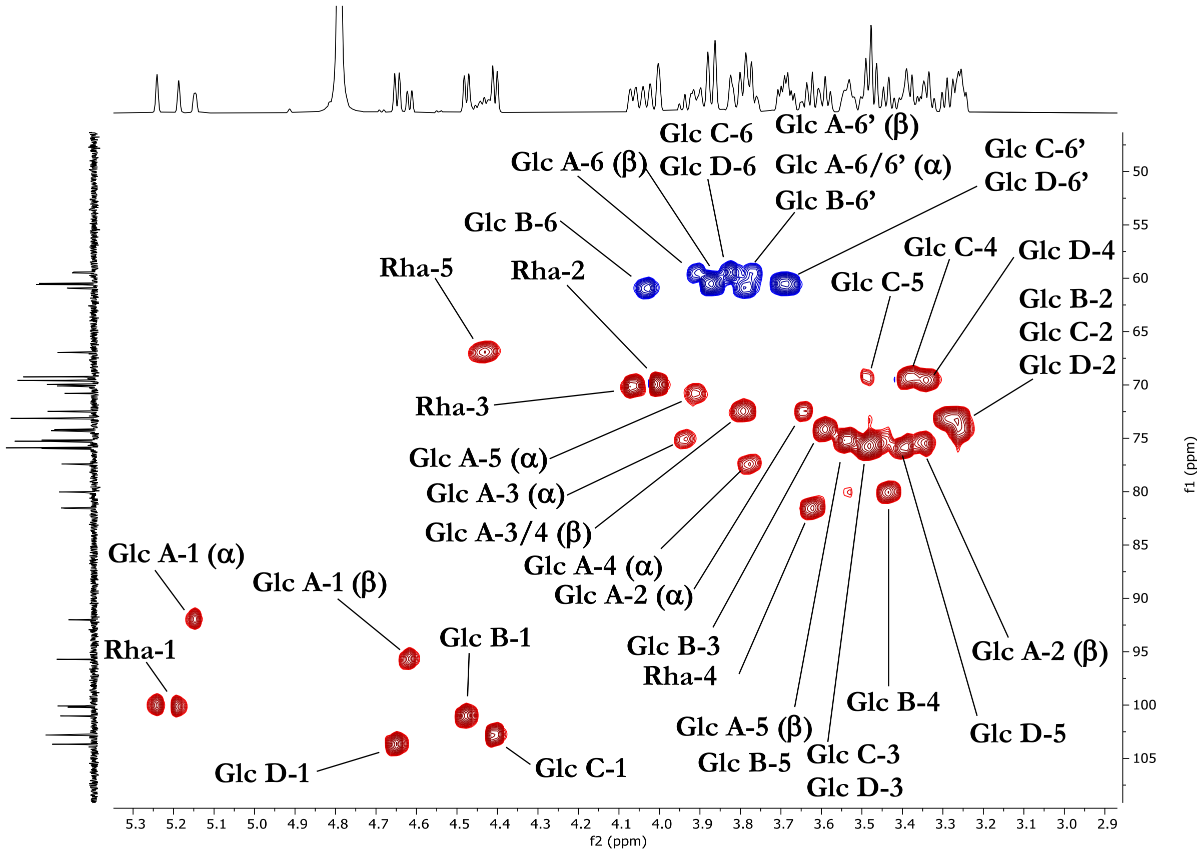

Figure S27 Excerpt of HSQC NMR (D_2O) of **5mer-III** with assignments.

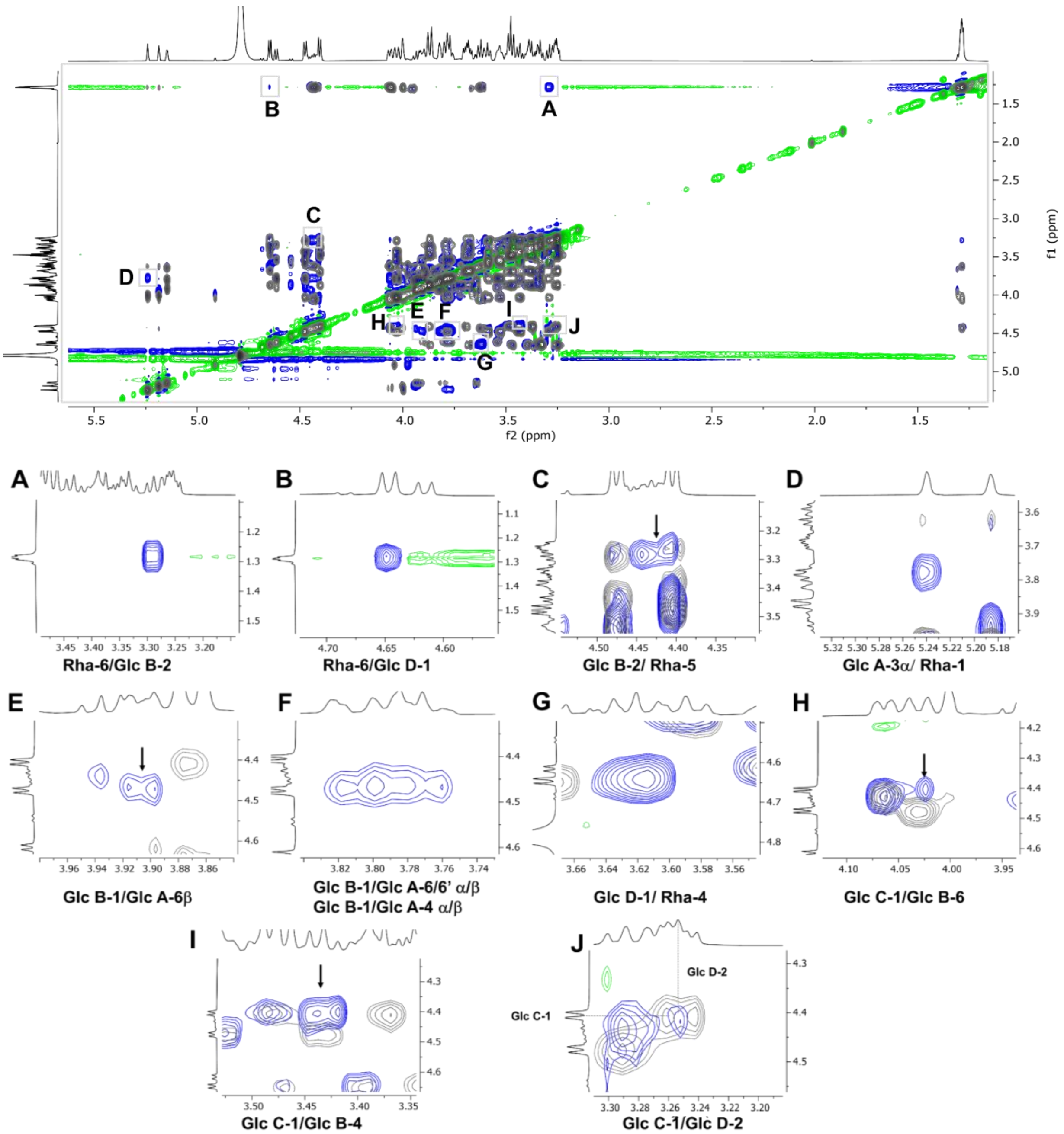

Figure S28 Overimposed 2D ROESY (green-blue, 700 MHz, p15 300 ms, 293 K, D_2O) of **5mer-III** with assignments and 2D TOCSY spectrum (gray, 700 MHz, d9 160 ms, D_2O).

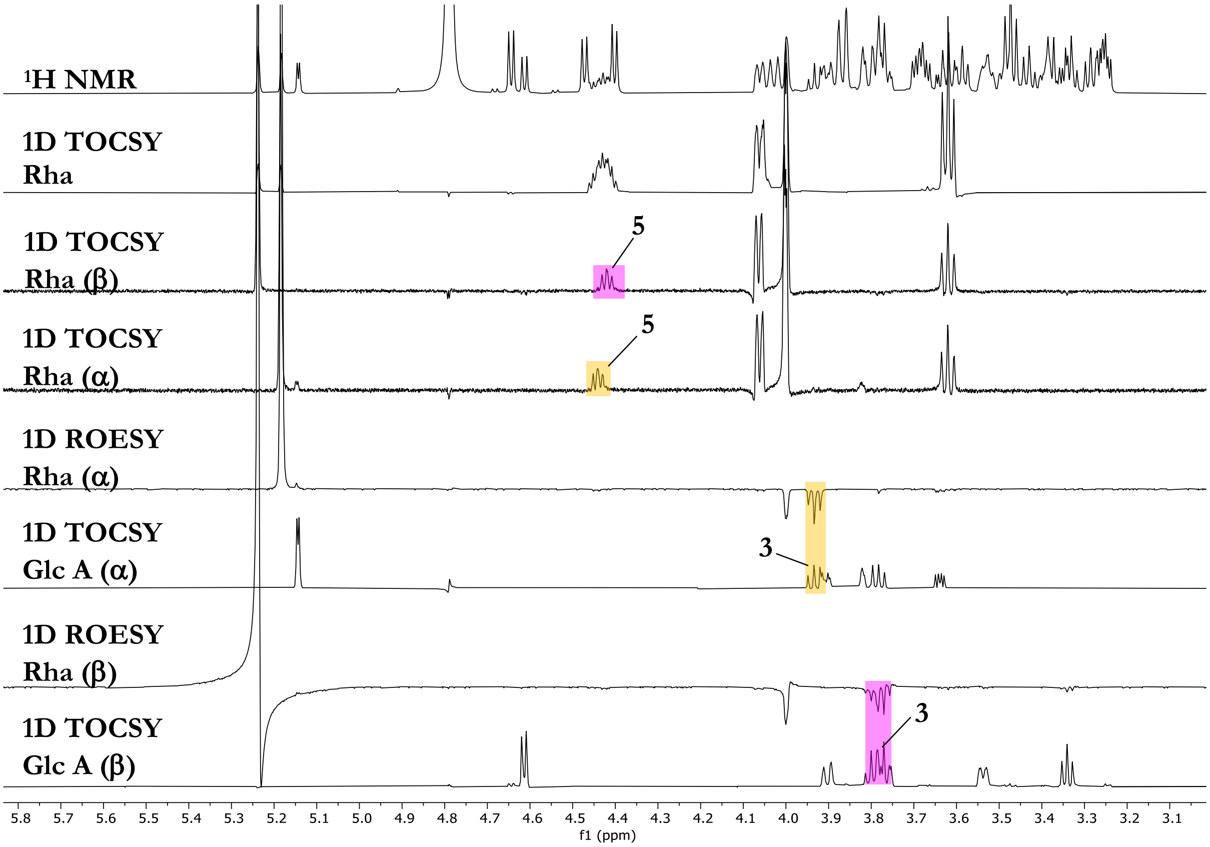

Figure S29 Overlay of 1D ROESY (700 MHz, p15 300 ms, 293 K, D_2O) and 1D TOCSY of **5mer-III**. The 1D ROESY was obtained by selective excitation of the Rha-1 (α) & (β) resonance (δ 5.18 & 5.24 ppm). The NOE between Glc A-3 (α)/Rha-1 (α) is highlighted with a yellow box and between Glc A-3 (β)/Rha-1 (β) is highlighted with a pink box. (α) and (β) refer to the configuration of the reducing end.

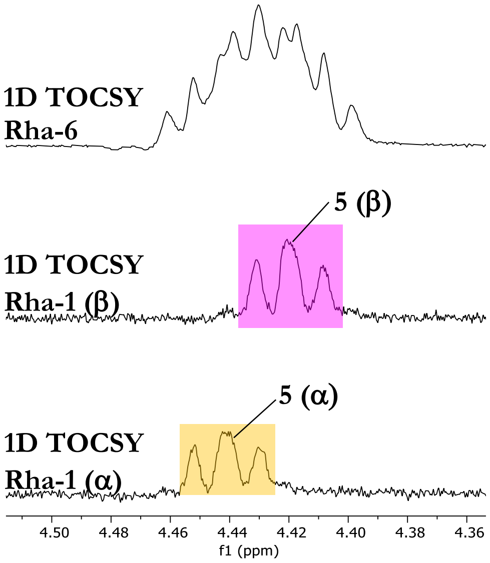

Figure S30 Excerpt of the overlay of 1D TOCSY of Rha-6 (700 MHz, d9 200 ms, D_2O) of **5mer-III** and selective excitation of Rha-1 (α) & (β) (700 MHz, d9 300 ms D_2O) to see Rha-5 (β) (pink box) and Rha-5 (α) (yellow box). (α) and (β) refer to the configuration of the reducing end.

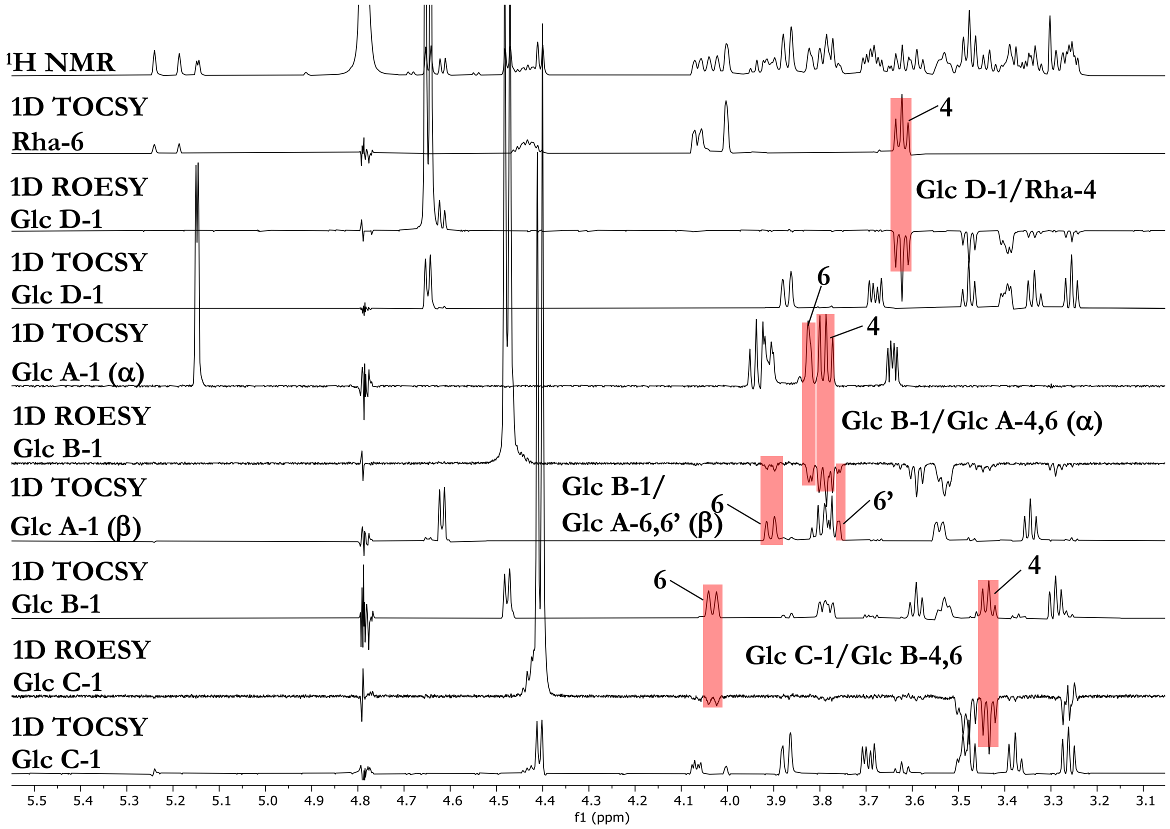

Figure S31 Overlay of 1D ROESY (700 MHz, p15 300 ms, 293 K, D_2O) and 1D TOCSY of **5mer-III**. The ROEs between Glc D-1/Rha-4, Glc B-1/Glc A-4,6 (α) & Glc A-6,6' (β) and Glc C-1/ Glc B-4,6 are highlighted with red boxes.

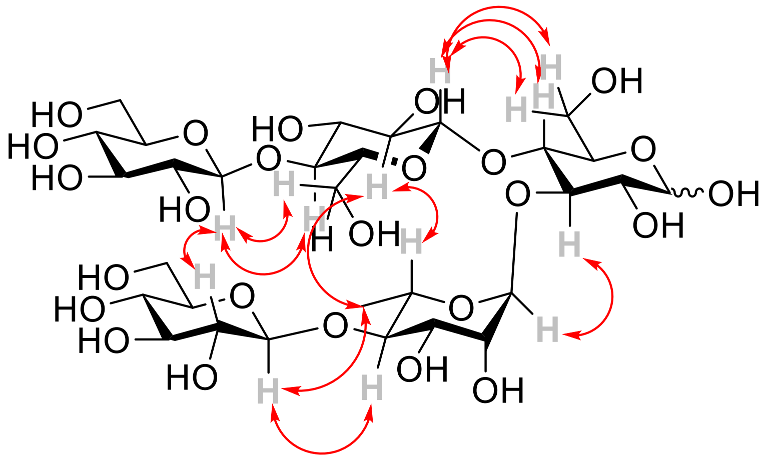

Figure S32 All experimentally observed NOEs (red arrows).

4.4.2 5mer-I *vs* 5mer-III

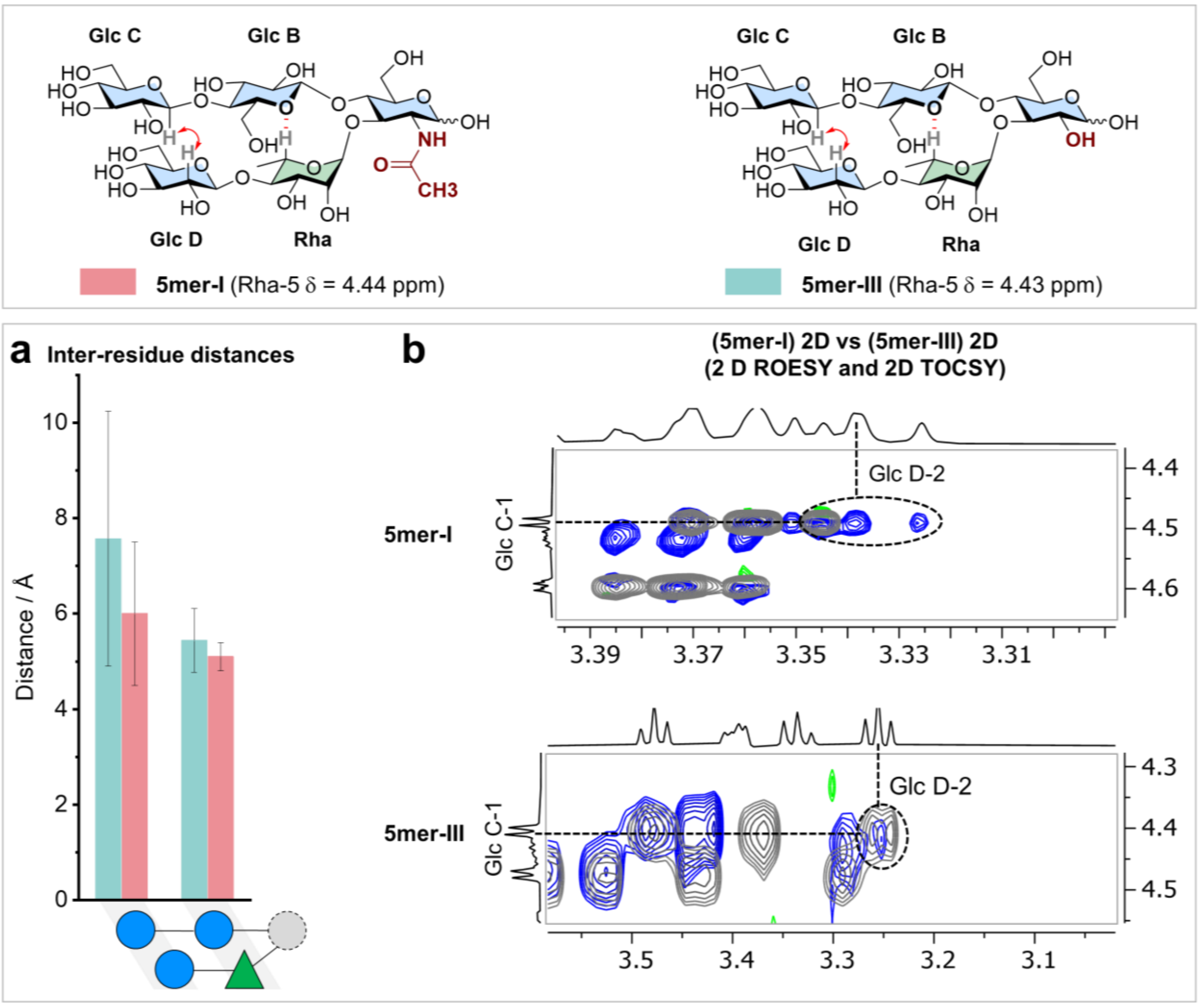

Figure S33 Comparison of **5mer-I** vs **5mer-III** (a) Inter-residue distances by molecular dynamics and (b) Inter-strand key NOE between the two strands (Glc B-1 and Glc C-2 in **5mer-I** vs Glc C-1 and Glc D-2 in **5mer-III**). NMR analysis confirmed the presence of the non-conventional H-bond for both hairpins, supported by the similar chemical shift for Rha-5. Key inter-residue NOEs between Glc B and Rha as well as between Glc B-1 and Glc C-2 were detected for both structures. Still, the inter-strand NOE signal between Glc B-1 and Glc C-2 was weaker in the case of **5mer-III**, suggesting the increased flexibility for **5mer-III**. In this case, the absence of the NAc group seems detrimental for foldamer stability (Fig. SX). Labelling of protons in a monosaccharide is done as follows: e.g. proton attached to C-1 of Glc B is named "Glc B-1".

4.4.3 NMR characterization of 5mer-III-Closed

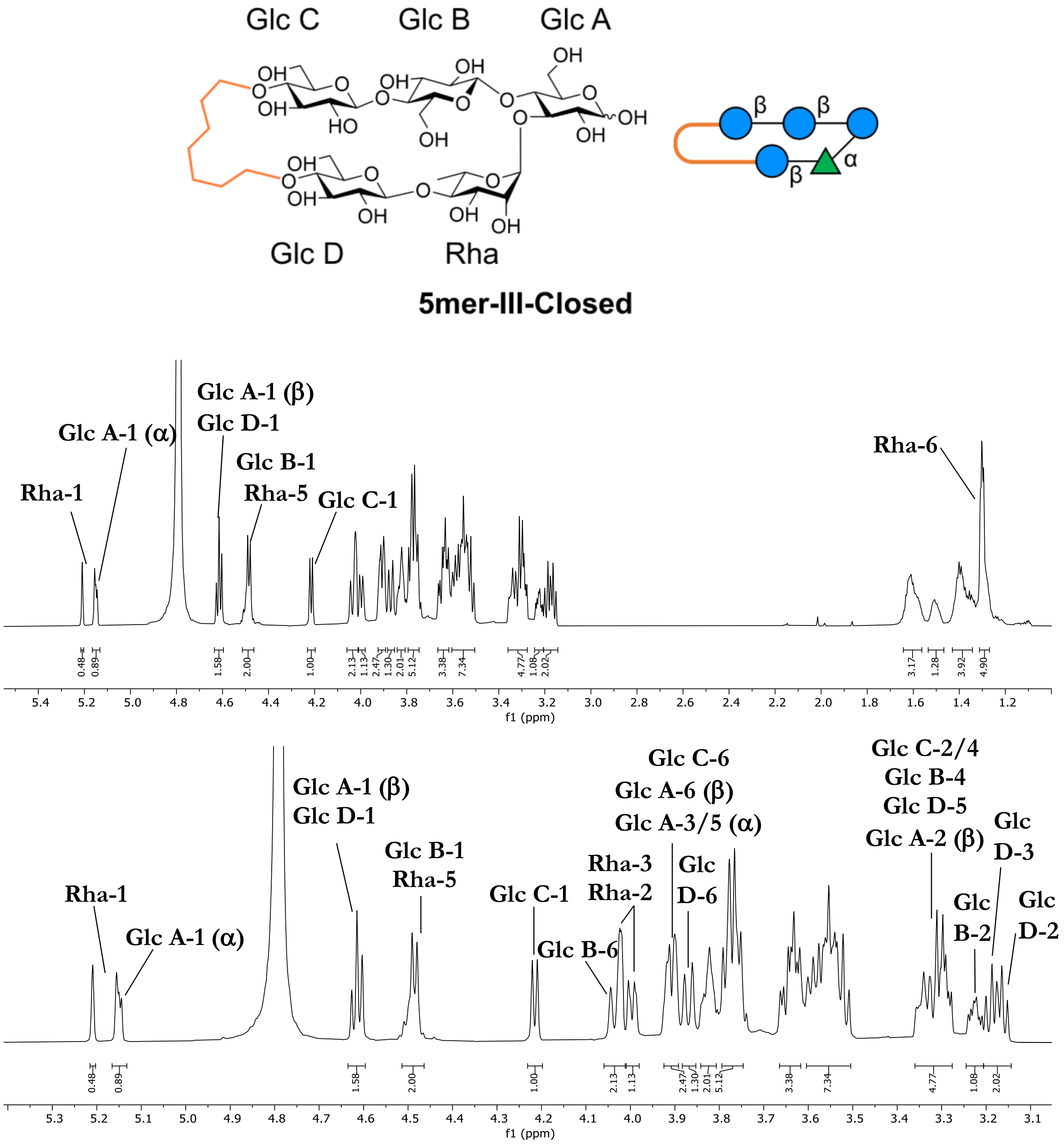

Figure S34 ^{1}H NMR (700 MHz, D_2O) of **5mer-III-Closed** with assignments

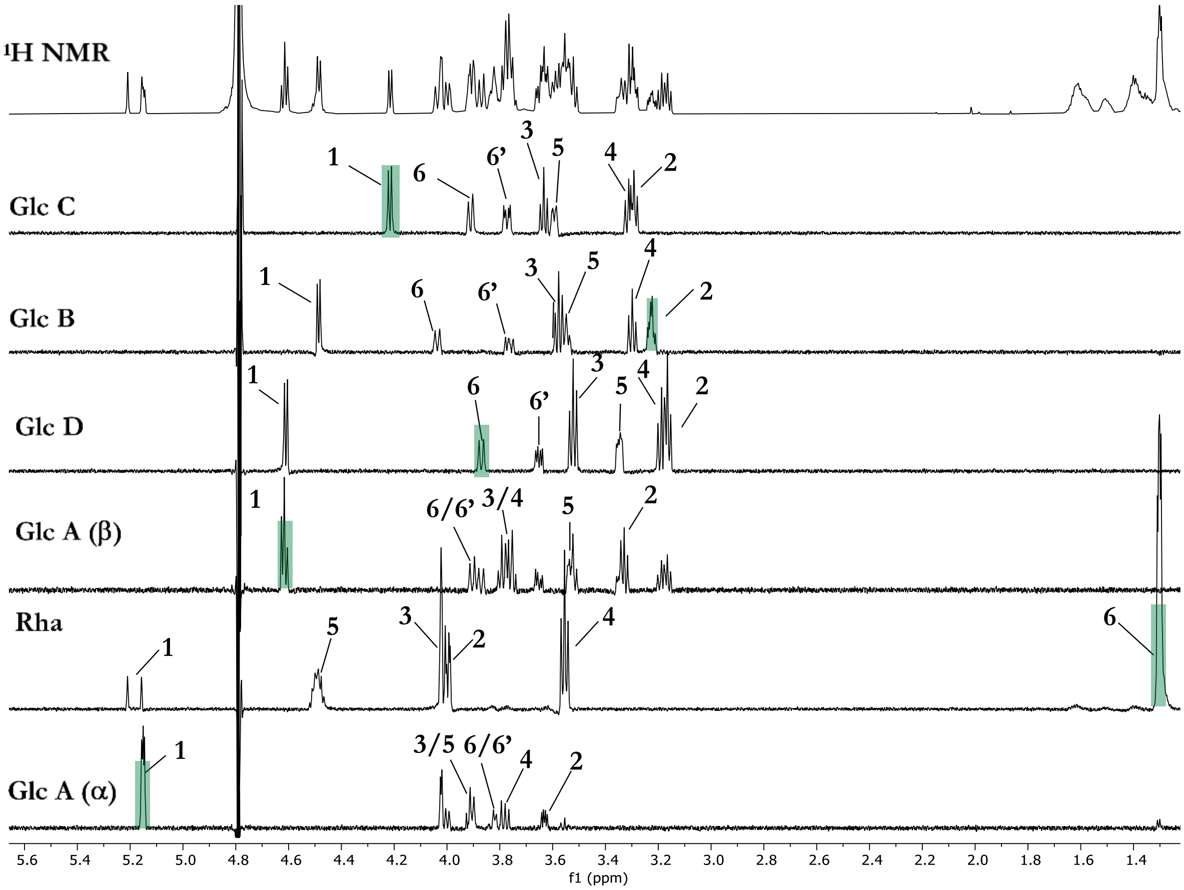

Figure S35 1D TOCSY (700 MHz, d9 200 ms, D_2O) of **5mer-III-Closed** with assignments. Resonances chosen for selective excitation are highlighted in green.

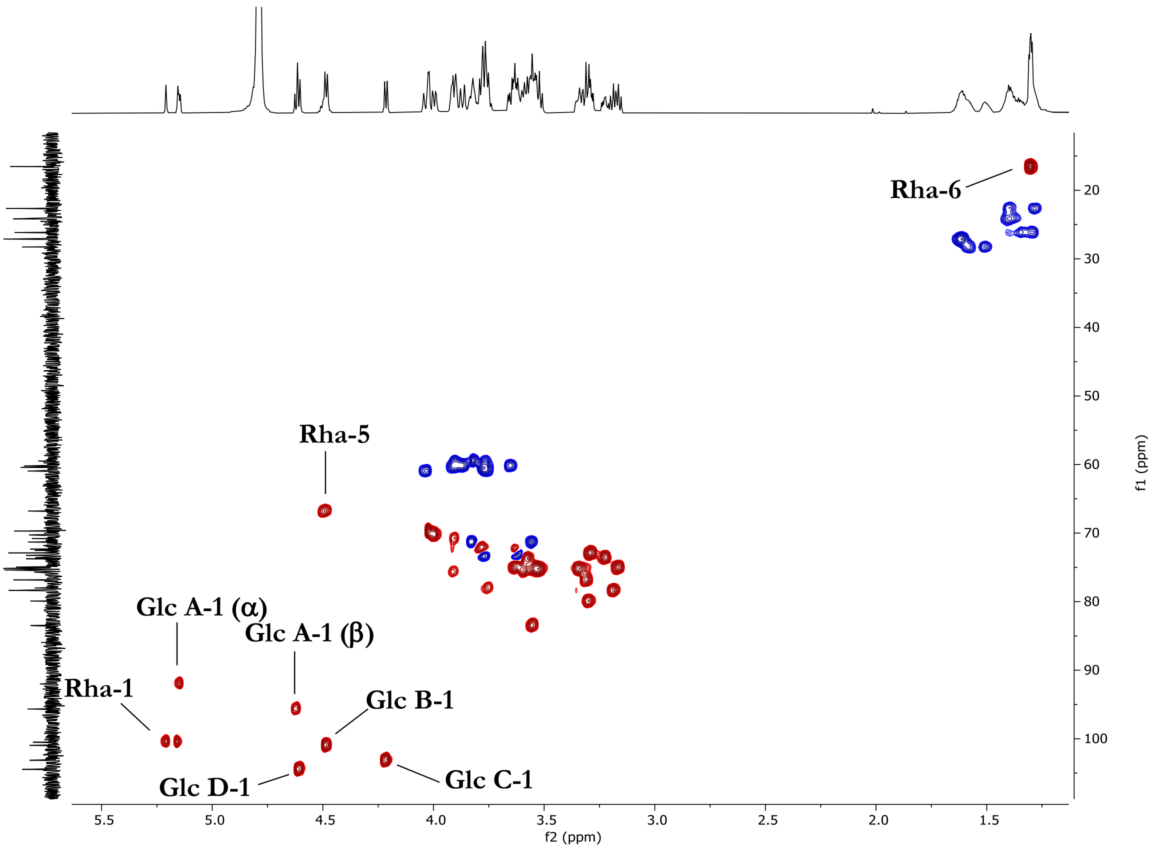

Figure S36 HSQC NMR (D_2O) of **5mer-III-closed** with assignment.

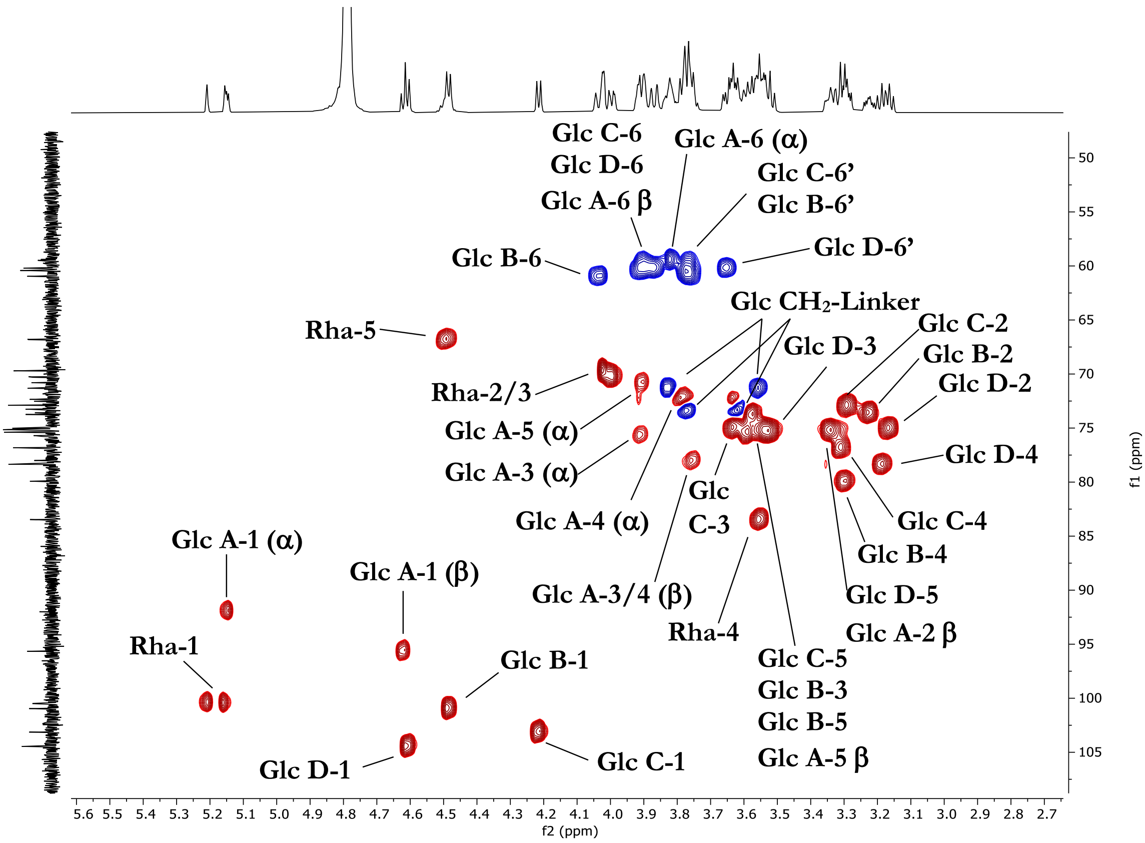

Figure S37 Excerpt of HSQC NMR (D_2O) of **5mer-III-Closed** with assignments.

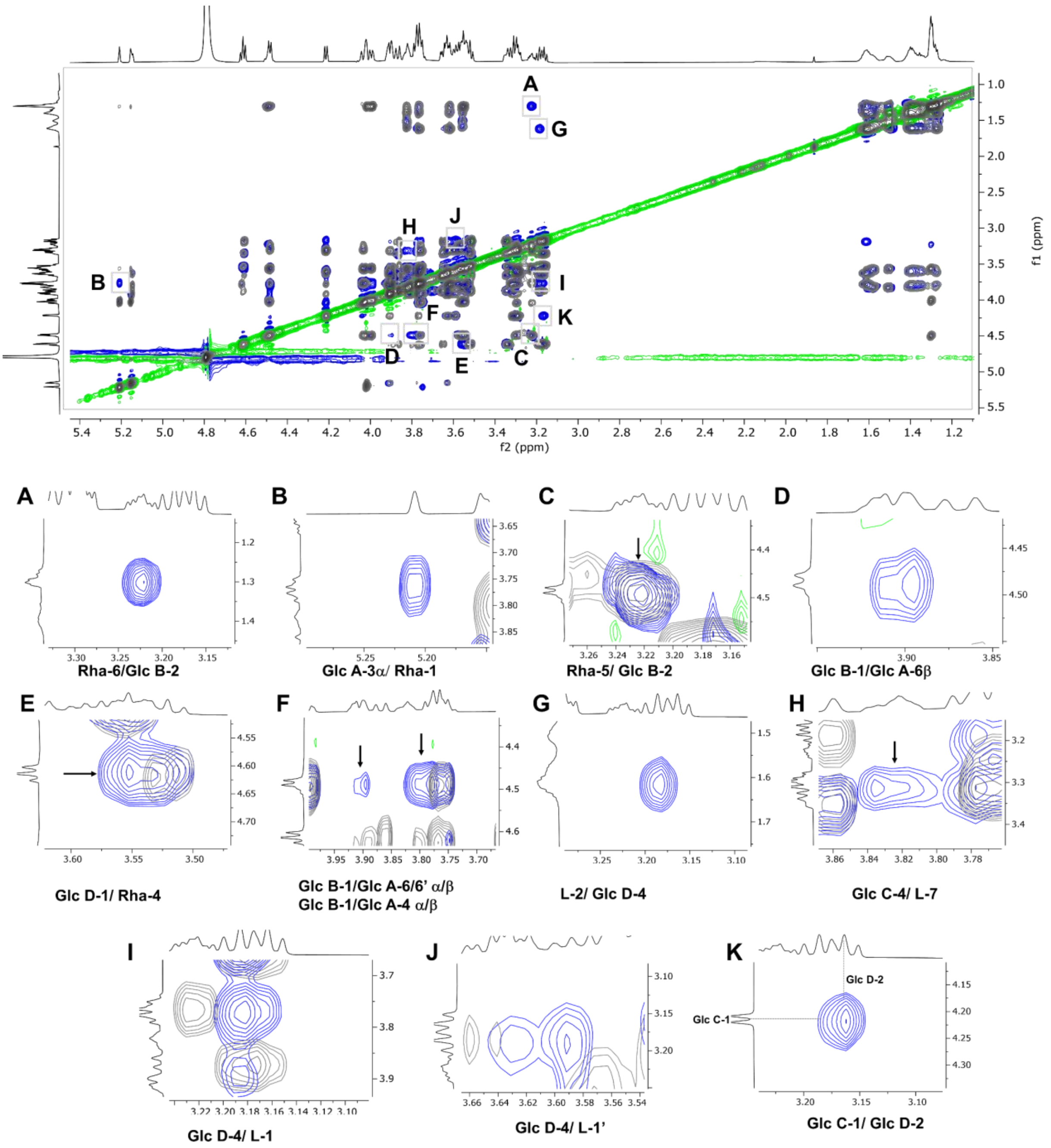

Figure S38 Overimposed 2D ROESY (green-blue, 700 MHz, p15 300 ms, 293 K, D_2O) of **5mer-III-Closed** with assignments and 2D TOCSY spectrum (gray, 700 MHz, d9 150 ms, D_2O).

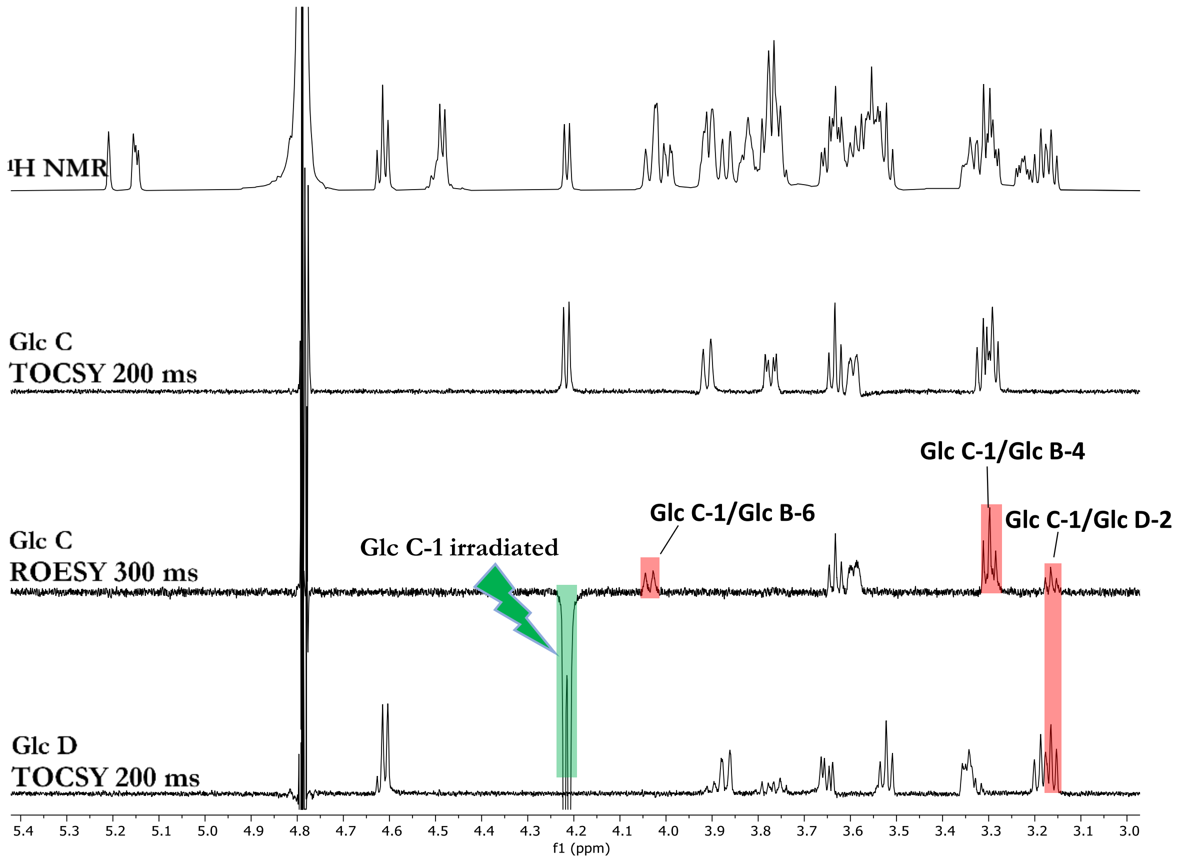

Figure S39: Overlay of 1D ROESY (700 MHz, p15 300 ms, 293 K, D_2O) and 1D TOCSY (700 MHz, d9 200 ms, D_2O) of **5mer-III-Closed.** The ROEs between Glc C-1/Glc B-6, Glc C-1/Glc B-4 and Glc C-1/Glc D-2 are highlighted with red boxes

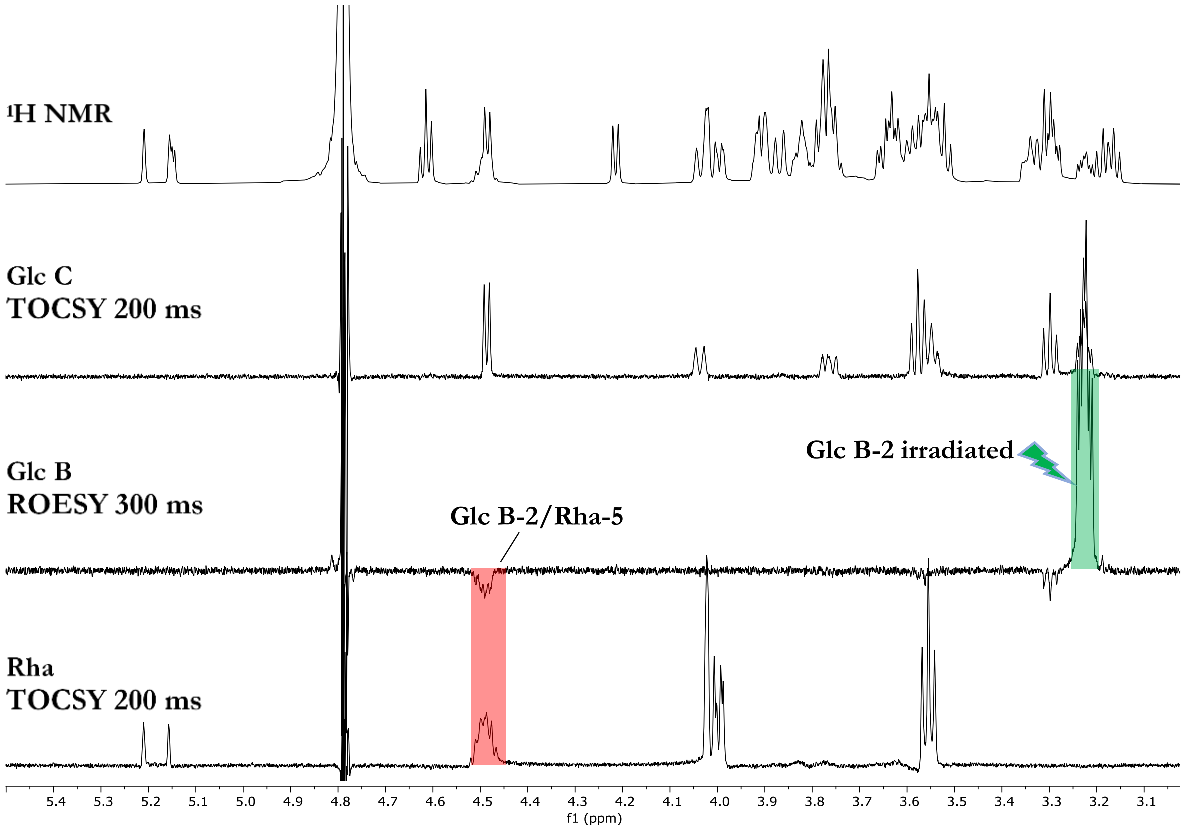

Figure S40 Overlay of 1D ROESY (700 MHz, p15 300 ms, 293 K, D_2O) and 1D TOCSY (700 MHz, d9 200 ms, D_2O) of **5mer-III-Closed.** The ROE between Glc B-1/Rha-5 highlighted with red box.

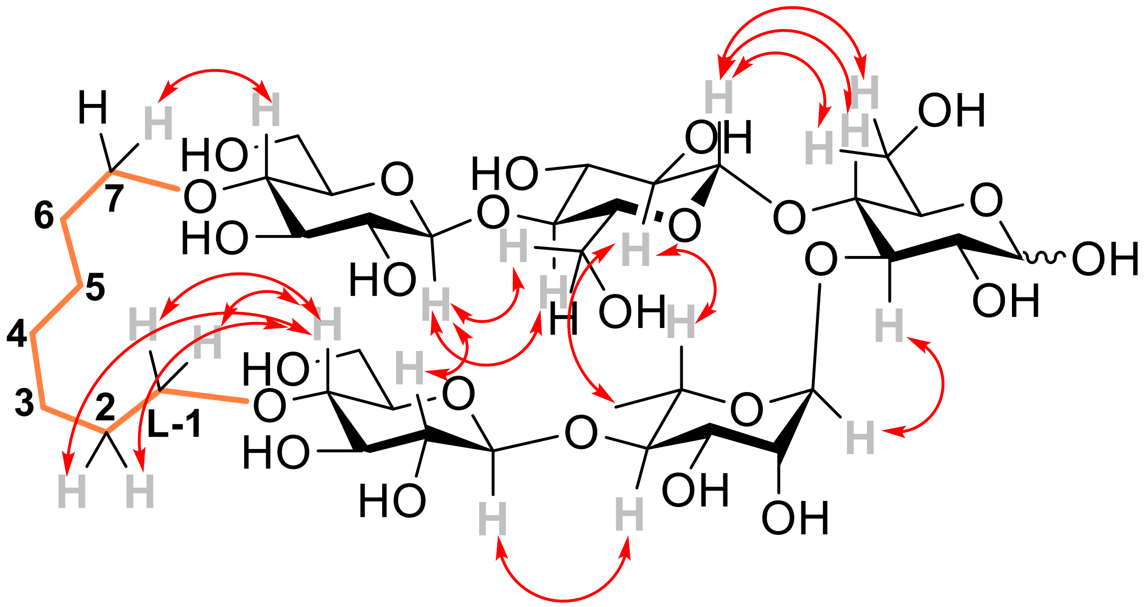

Figure S41: All experimentally observed NOEs (red arrows).

4.4.4 NMR characterization of 9mer-I-Linker

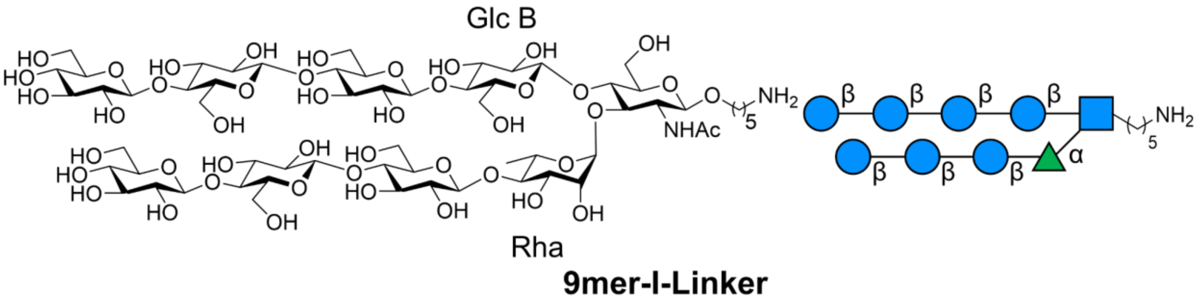

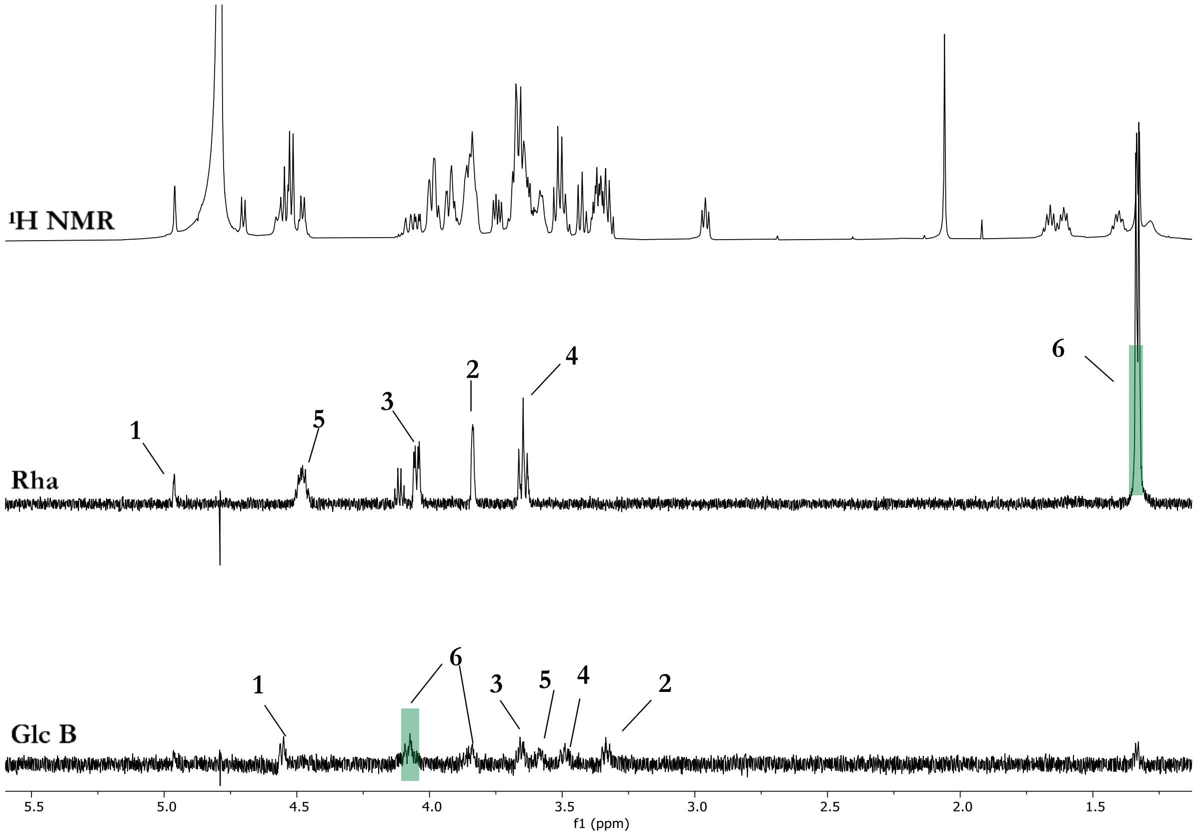

Figure S42 1D TOCSY (600 MHz, d9 200 ms, D_2O) of **9mer-I-Linker** with assignments. Resonances chosen for selective excitation are highlighted in green.

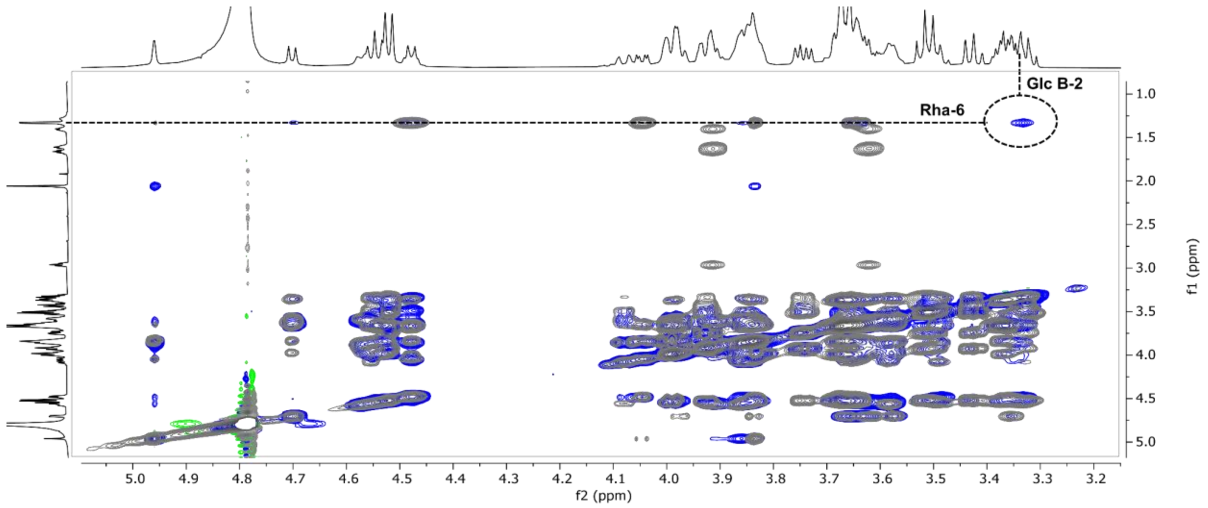

Figure S43 Overimposed 2D NOESY (green-blue, 600 MHz, d8 1000 ms, 298 K, D_2O) of **9mer-I-Linker** with assignments and 2D TOCSY spectrum (gray, 600 MHz, d9 150 ms, D_2O).

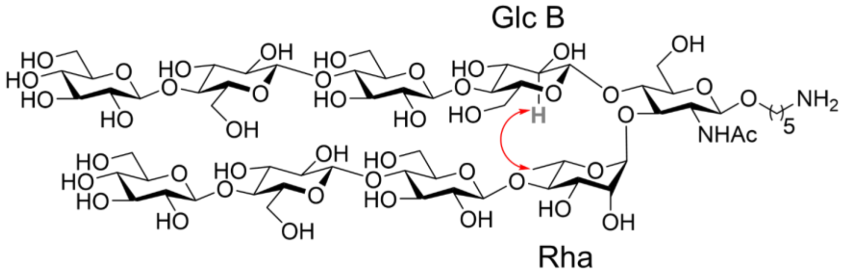

Figure S44 Experimentally observed inter-residue NOE (red arrow) in turn unit derived from Figure S43 for **9mer-I-Linker.**

4.4.5 NMR characterization of 9mer-III

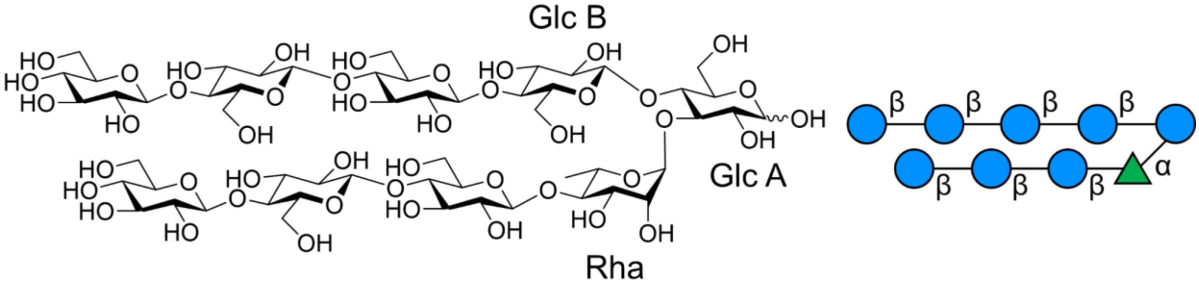

9mer-III

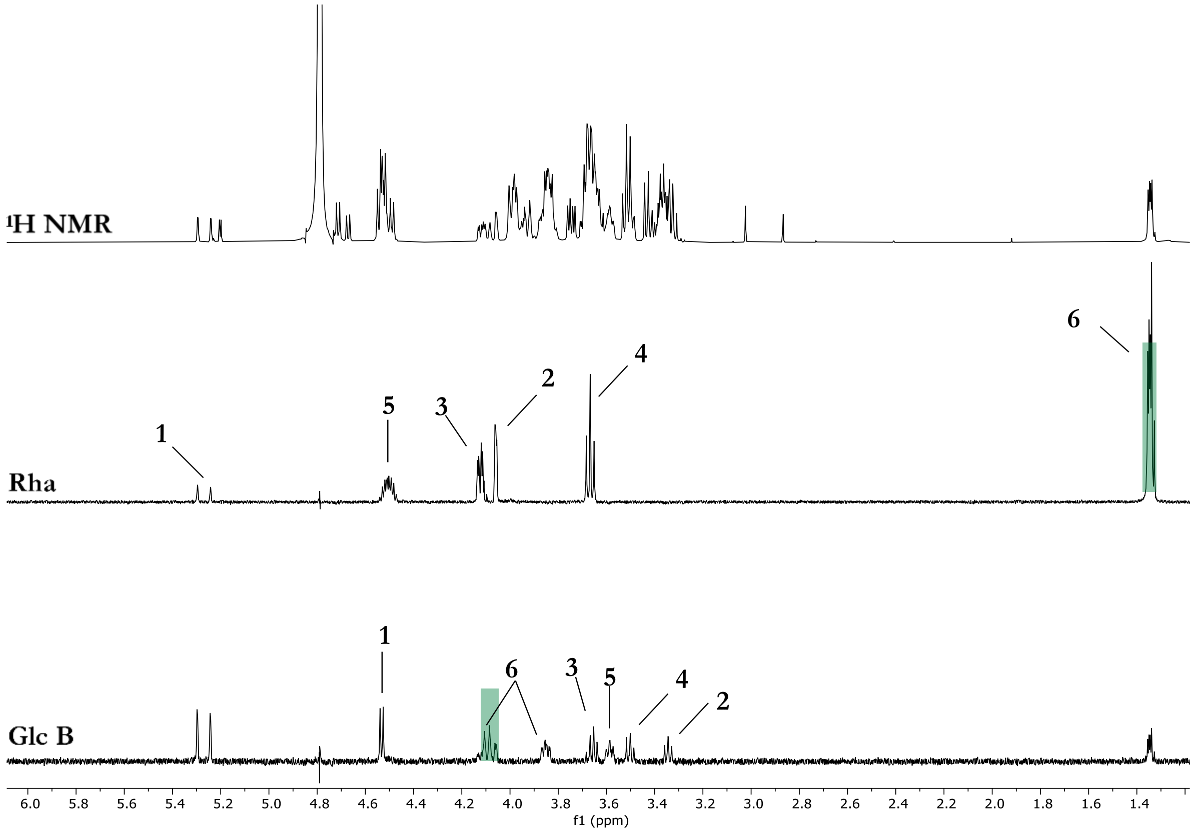

Figure S45 1D TOCSY (600 MHz, d9 200 ms, D_2O) of **9mer-III** with assignments. Resonances chosen for selective excitation are highlighted in green.

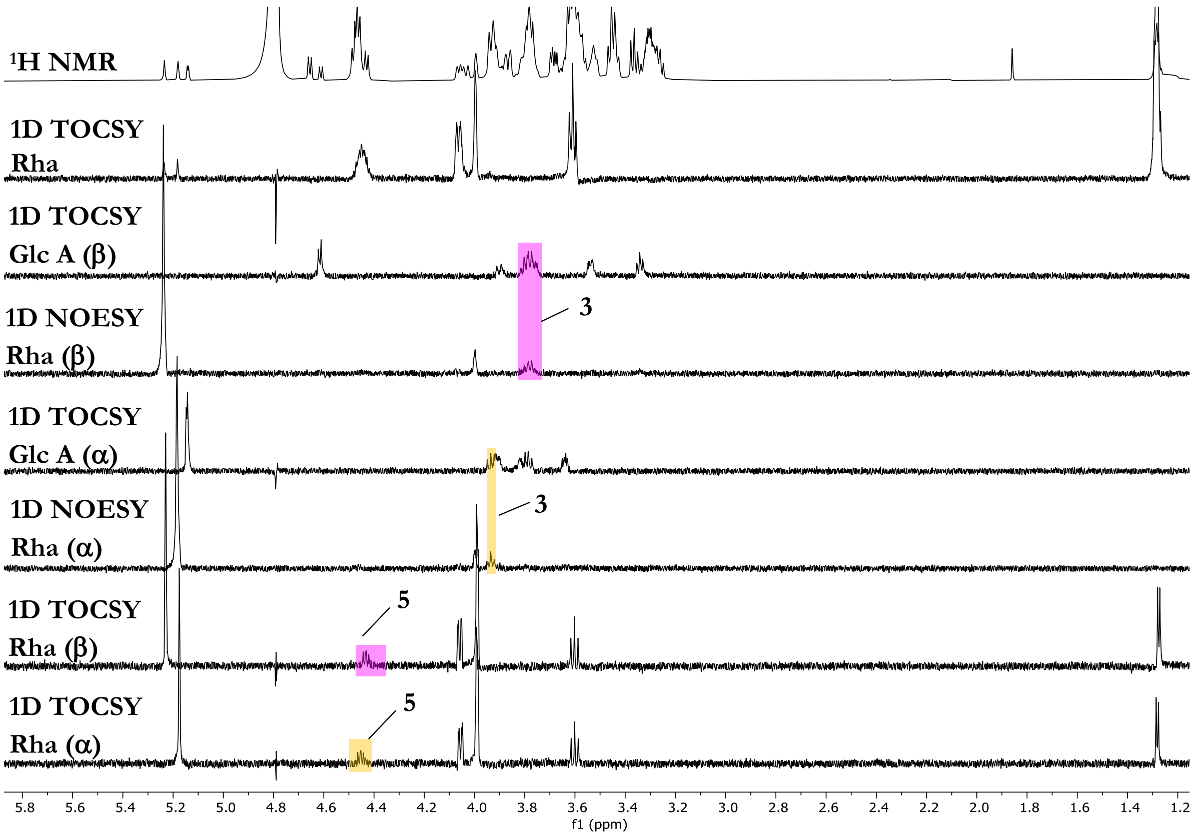

Figure S46 Overlay of 1D NOESY (700 MHz, d8 800 ms, 293 K, D_2O) and 1D TOCSY of **9mer-III**. The 1D NOESY was obtained by selective excitation of the Rha-1 (α) & (β) resonance (δ 5.18 & 5.23 ppm). The NOE between Glc A-3 (α)/Rha-1 (α) is highlighted with a yellow box and between Glc A-3 (β)/Rha-1 (β) is highlighted with a pink box. (α) and (β) refer to the configuration of the reducing end.

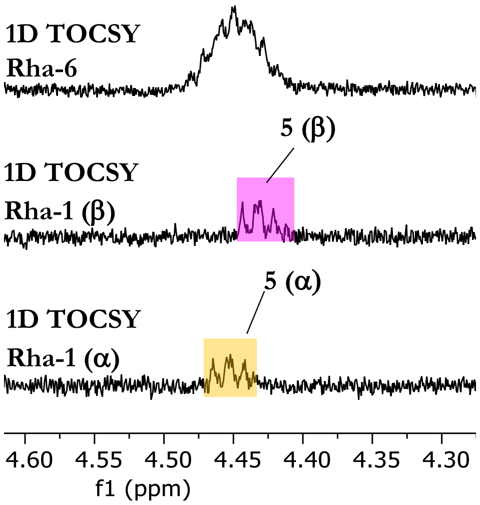

Figure S47 Excerpt of the overlay of 1D TOCSY of Rha-6 (700 MHz, d9 200 ms, D_2O) of **9mer-III** and selective excitation of Rha-1 (α) & (β) (700 MHz, d9 300 ms, D_2O) to see Rha-5 (β) (pink box) and Rha-5 (α) (yellow box). (α) and (β) refer to the configuration of the reducing end.

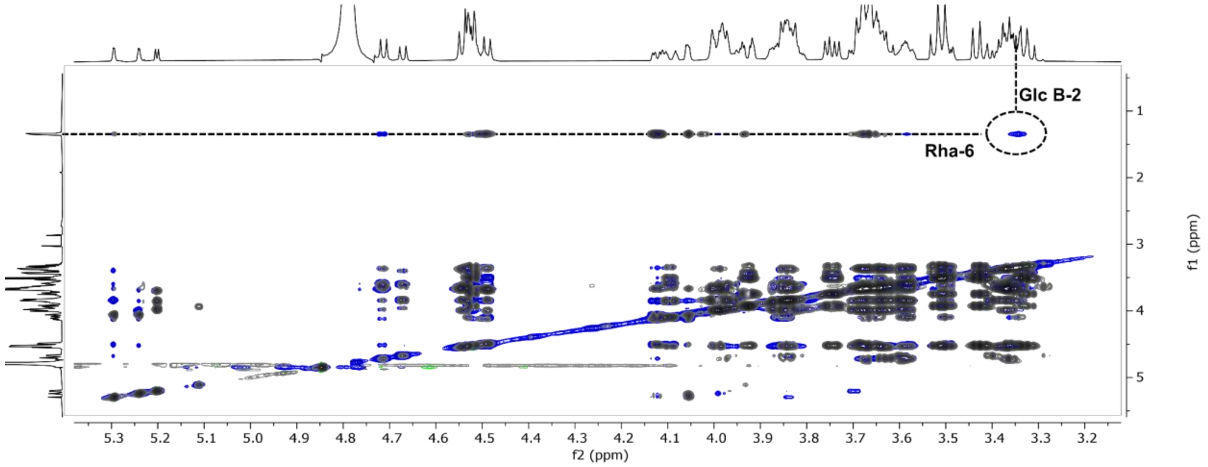

Figure S48 Overimposed 2D NOESY (green-blue, 700 MHz, d8 1000 ms, 293 K, D_2O) of **9mer-III** with assignments and 2D TOCSY spectrum (gray, 700 MHz, d9 150 ms, D_2O).

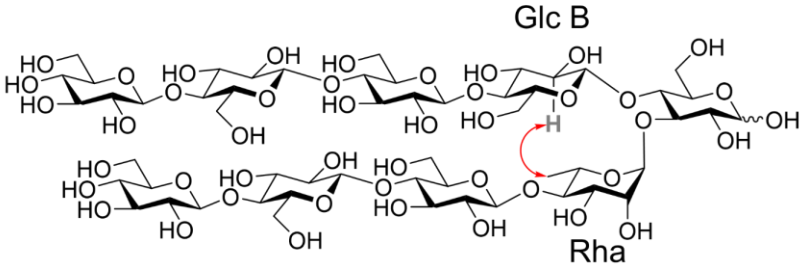

Figure S49 Experimentally observed inter-residue NOE (red arrow) in turn unit derived from Figure S48 for **9mer-III.**

4.4.6 NMR characterization of 9mer-IV

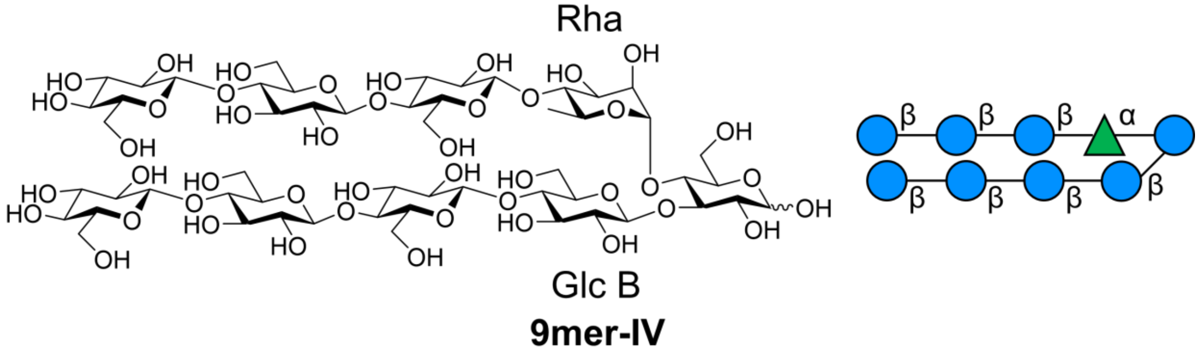

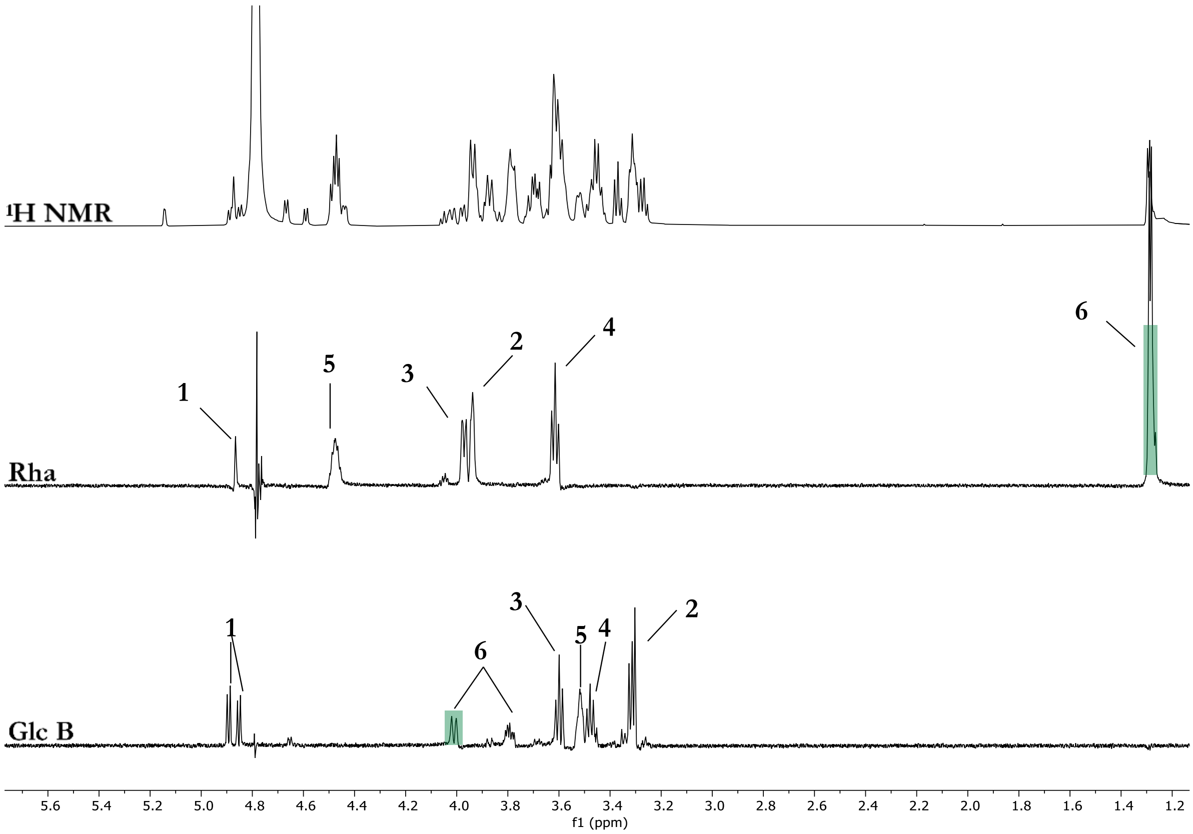

Figure S50 1D TOCSY (700 MHz, d9 200 ms, D_2O) of **9mer-IV** with assignments. Resonances chosen for selective excitation are highlighted in green.

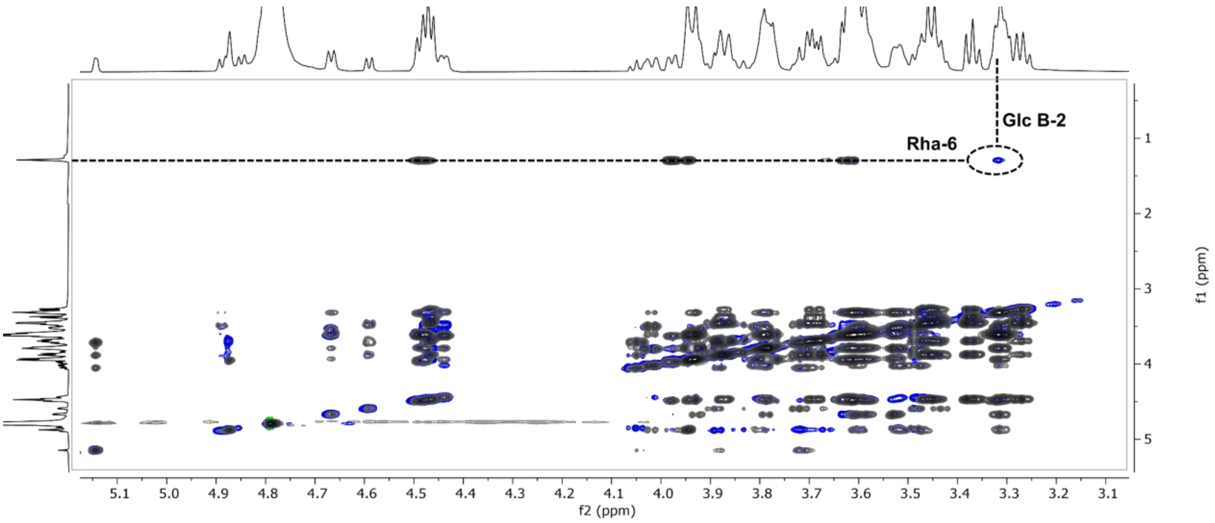

Figure S51 Overimposed 2D NOESY (green-blue, 700 MHz, d8 1000 ms, 293 K, D_2O) of **9mer-IV** with assignments and 2D TOCSY spectrum (gray, 700 MHz, d9 150 ms, D_2O).

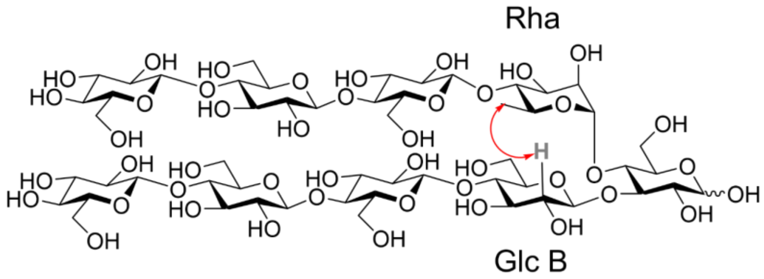

Figure S52 Experimentally observed inter-residue NOE (red arrow) in turn unit derived from Figure S51 for **9mer-IV.**

4.4.7 Effect of temperature, turn unit and strand length on hairpin stability

Compound	δ Rha-5 (ppm) 278 K	δ Rha-5 (ppm) 298 K	δ Rha-5 (ppm) 318 K	Δδ (ppm)* 298 K
3mer-V		4.06		0
9mer-I	4.49	4.48	4.44	0.42
9mer-I-Linker	4.47	4.46	4.42	0.40
9mer-III	4.49	4.48	4.45	0.42
9mer-IV	4.53	4.51	4.47	0.45

Table S2 Comparison of the chemical shifts for Rha-5 at different temperatures (from 278 K to 318 K). *Δδ at room temperature was calculated as follows:

$$\Delta\delta = \delta_{(Rha-5,\boldsymbol{compound})} - \delta_{(Rha-5,\boldsymbol{3mer-V})}$$

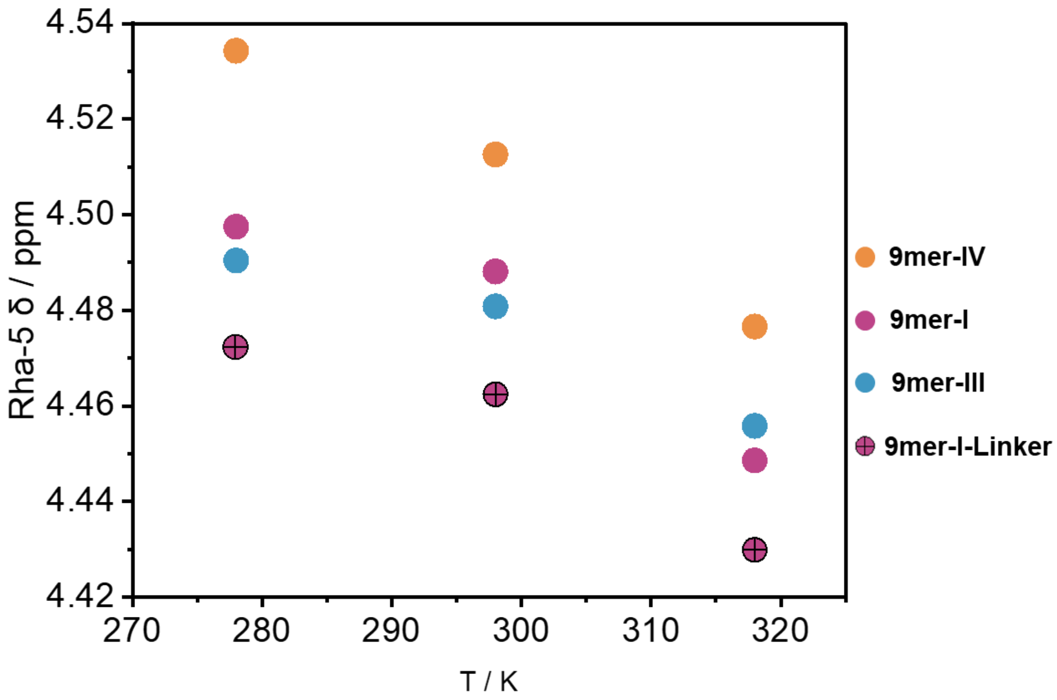

Figure S53 Temperature dependent ^{1}H NMR analysis of **9mer-I, 9mer-I-Linker, 9mer-III** and **9mer-IV**. The chemical shifts for Rha-5 were extracted from selective 1D TOCSY (700 MHz, d9 200 ms, D_2O) at different temperature.

Compound	δ Rha-5 (ppm) 278 K	δ Rha-5 (ppm) 298 K	δ Rha-5 (ppm) 318 K	Δδ (ppm)* 298 K
3mer-V		4.06		0
3mer-III	4.42	4.40	4.37	0.34
5mer-III	4.47	4.46	4.43	0.40
9mer-III	4.49	4.48	4.45	0.42
6mer-III	4.43	4.41	4.33	0.35

Table S3 Comparison of the chemical shifts for Rha-5 at different temperatures (from 278 K to 318 K). *Δδ at room temperature was calculated as follows:

$$\Delta\delta = \delta_{(Rha-5,\boldsymbol{compound})} - \delta_{(Rha-5,\boldsymbol{3mer-V})}$$

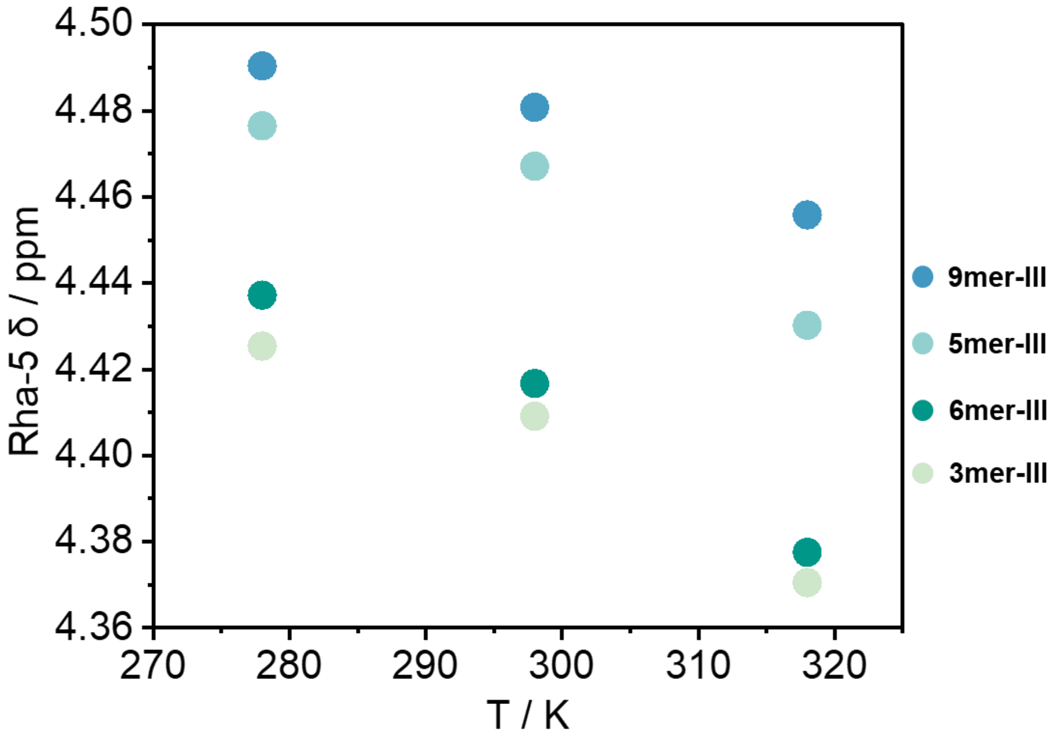

Figure S54 Temperature dependent ^{1}H NMR analysis of **3mer-III, 5mer-III, 9mer-III** and **6mer-III**. The chemical shifts for Rha-5 were extracted from selective 1D TOCSY (700 MHz, d9 200 ms, D_2O) at different temperature.

4.4.8 Determination of the non-conventional H-bond

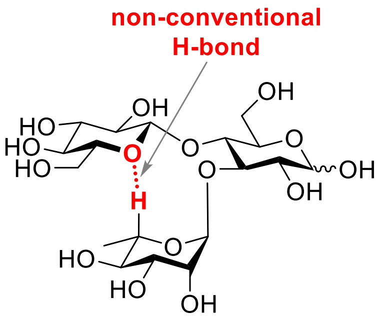

3mer-III

Compound	δ Rha-5 (ppm)	Δδ (ppm)*
**5mer-III**	4.43	0.37
**5mer-III-Closed**	4.49	0.43
**6mer-III**	4.37	0.31
**7mer-III-F**	4.45	0.39
**9mer-I-Linker**	4.46	0.40
**9mer-III**	4.45	0.39
**9mer-IV**	4.51	0.45
**13mer-III**	4.45	0.39
**15mer-III**	4.45	0.39
**17mer-III**	n.d.	-
**3mer-V**	4.06	0

Table S4 Comparison of the chemical shifts of Rha-5 extracted from 2D HSQC spectra for all the compounds synthesized in this work and compared with the **3mer-V** (lacking non-conventional H-bond) synthesized in previous work.[2] *Δδ was calculated as follows:

$$\Delta\delta = \delta_{(Rha-5,\boldsymbol{compound})} - \delta_{(Rha-5,\boldsymbol{3me-V})}$$

All the compounds show a significant downfield shift (Δδ ≈ 0.3-0.5 ppm) of Rha-5 proton compared to 3mer-V, indicating the presence of the non-conventional H-bond.[10]

4.4.9 NMR Characterization of 7mer-III-F

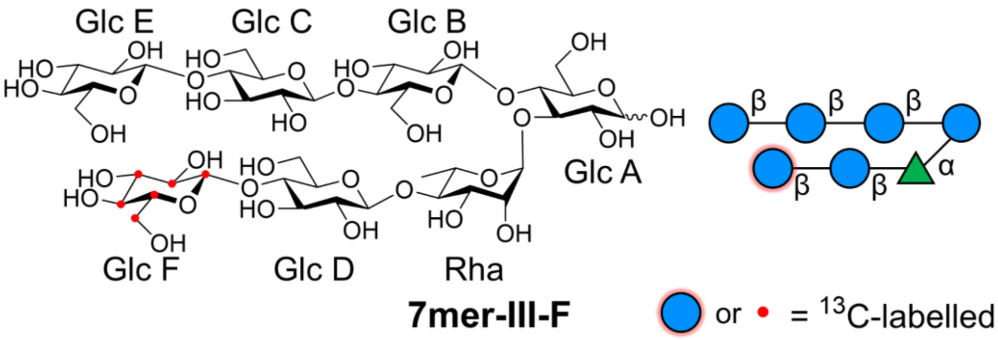

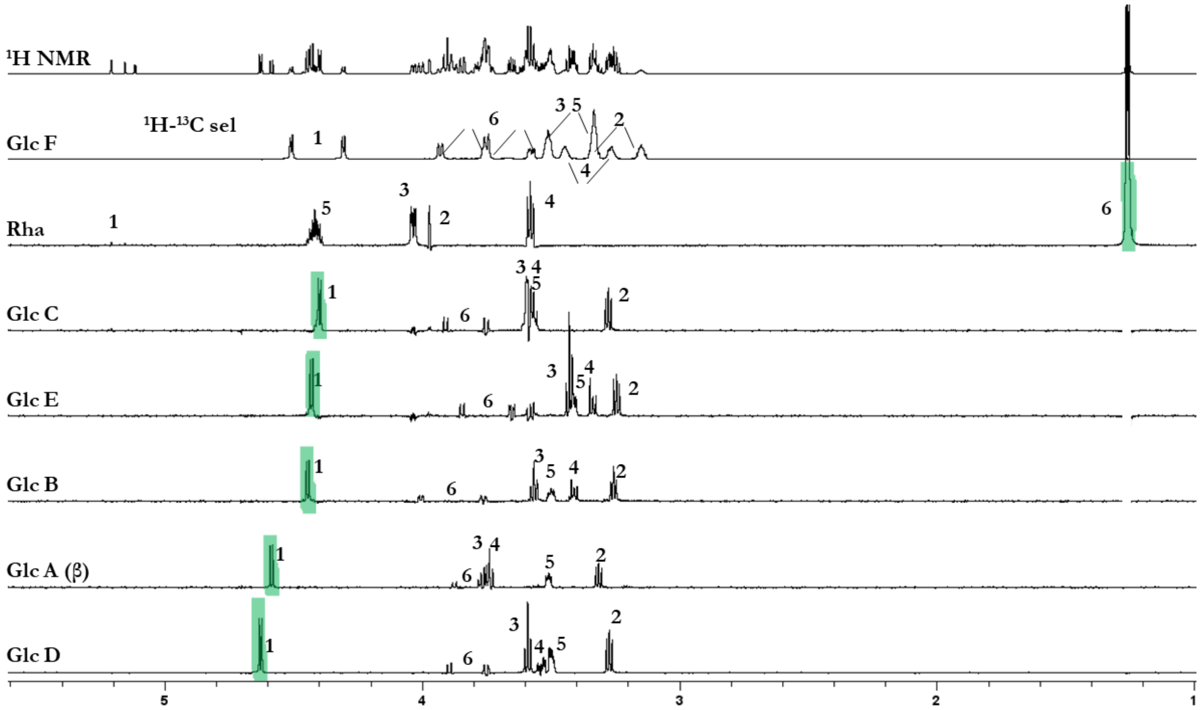

Figure S55 ^{1}H-NMR, ^{1}H-^{13}C selected and 1D TOCSY (800 MHz, d9 80 ms, D_2O) of **7mer-III-F** with assignments. Resonances chosen for selective excitation are highlighted in green.

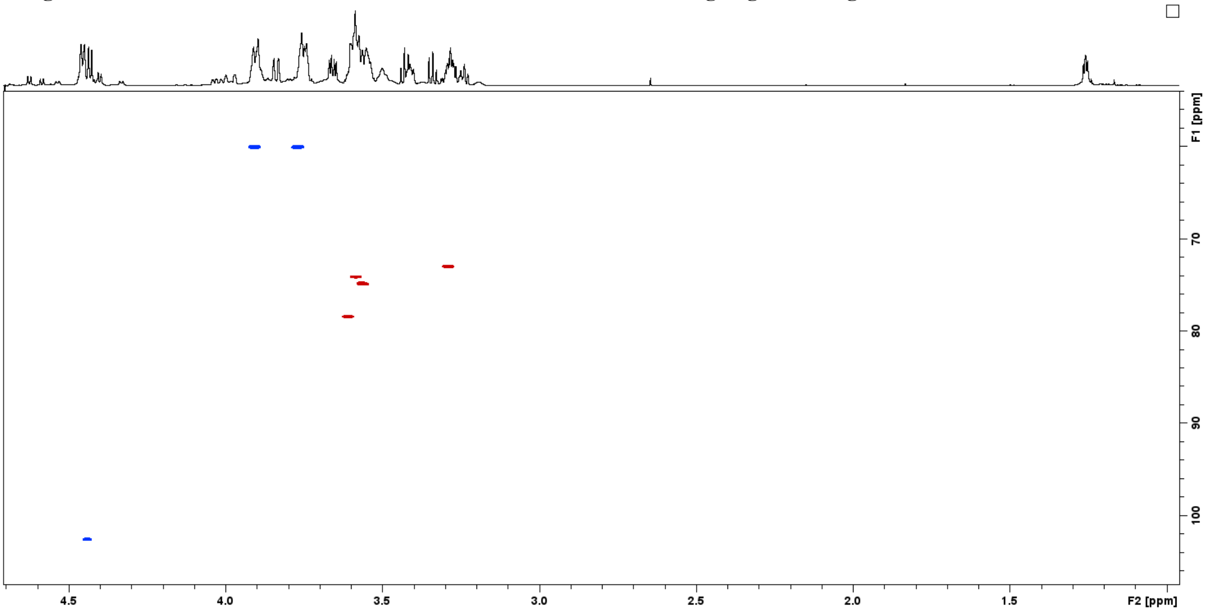

Figure S56 HSQC ^{13}C decoupled of **7mer-III-F**. Only ^{13}C-labelled signals are visible.

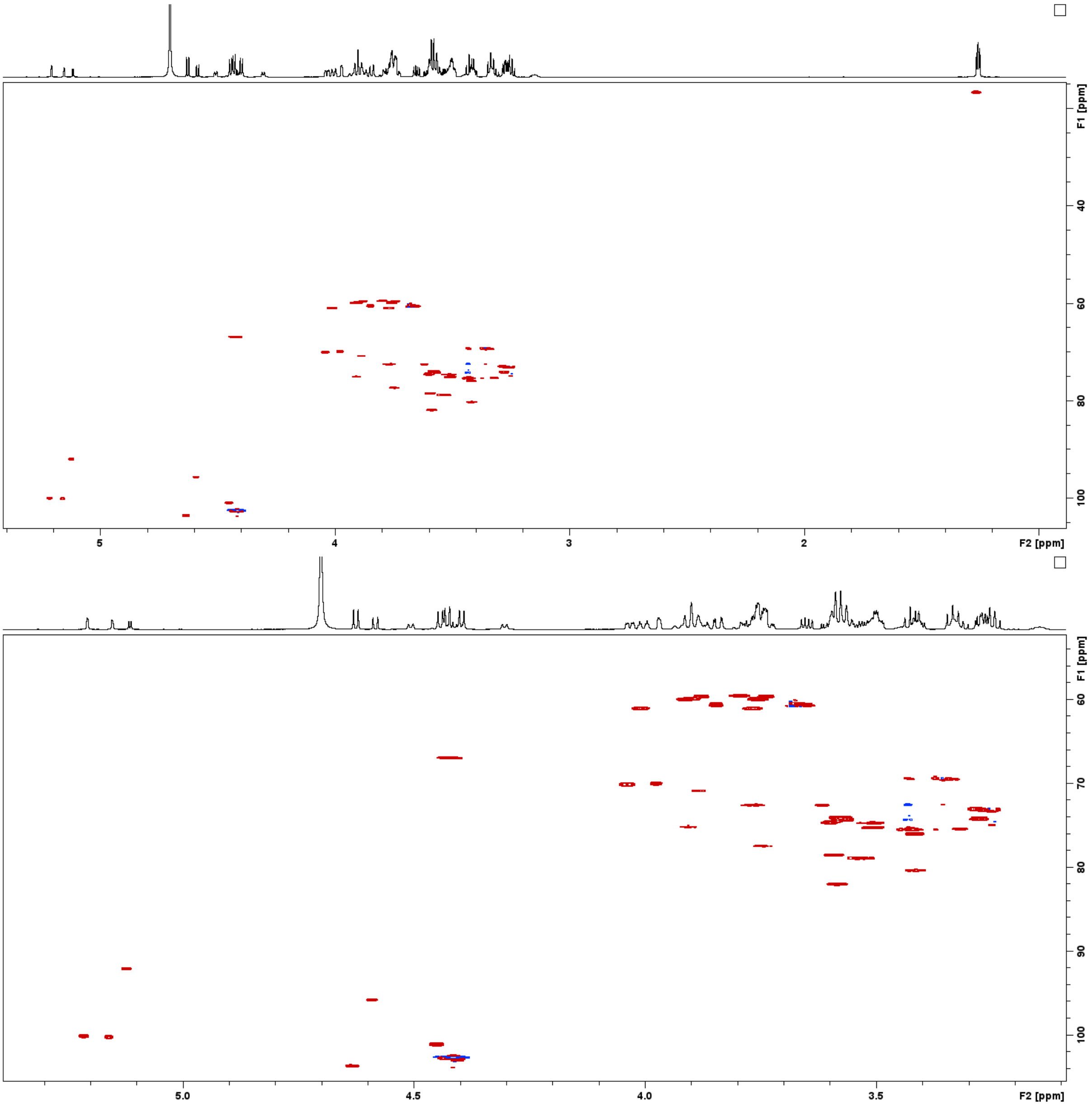

Figure S57 HSQC-ct to suppress the ^{13}C-labelled signals (800 MHz, ct 11.2 ms, D_2O) of **7mer-III-F**.

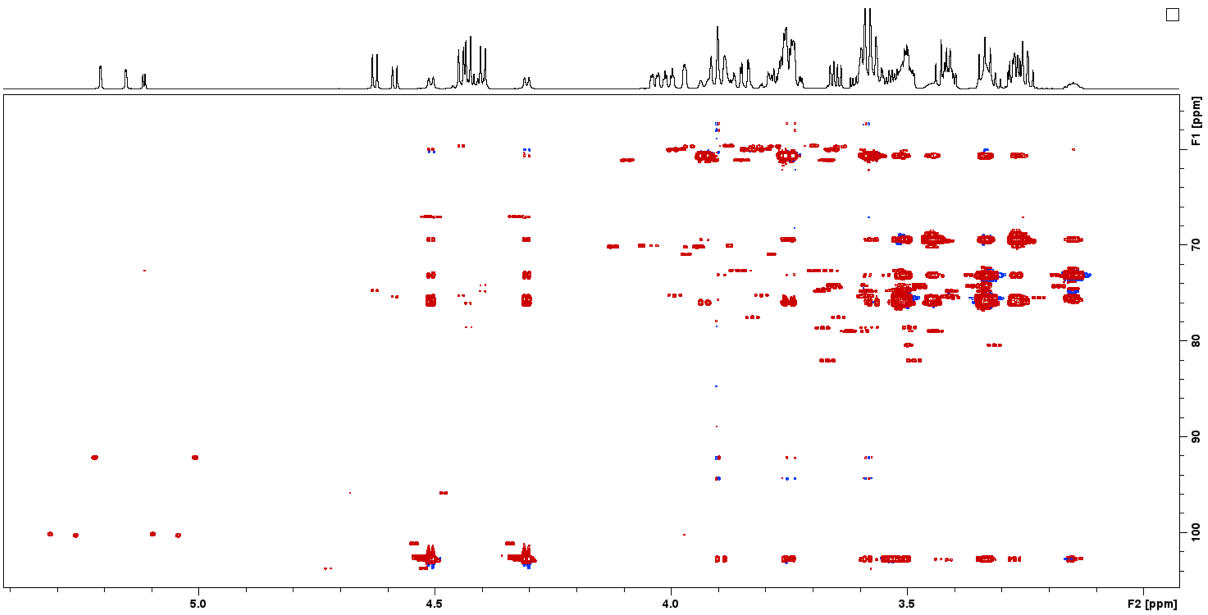

Figure S58 HSQC-NOESY ^{13}C coupled (800 MHz, d8 400 ms, D_2O) for **7mer-III-F**.

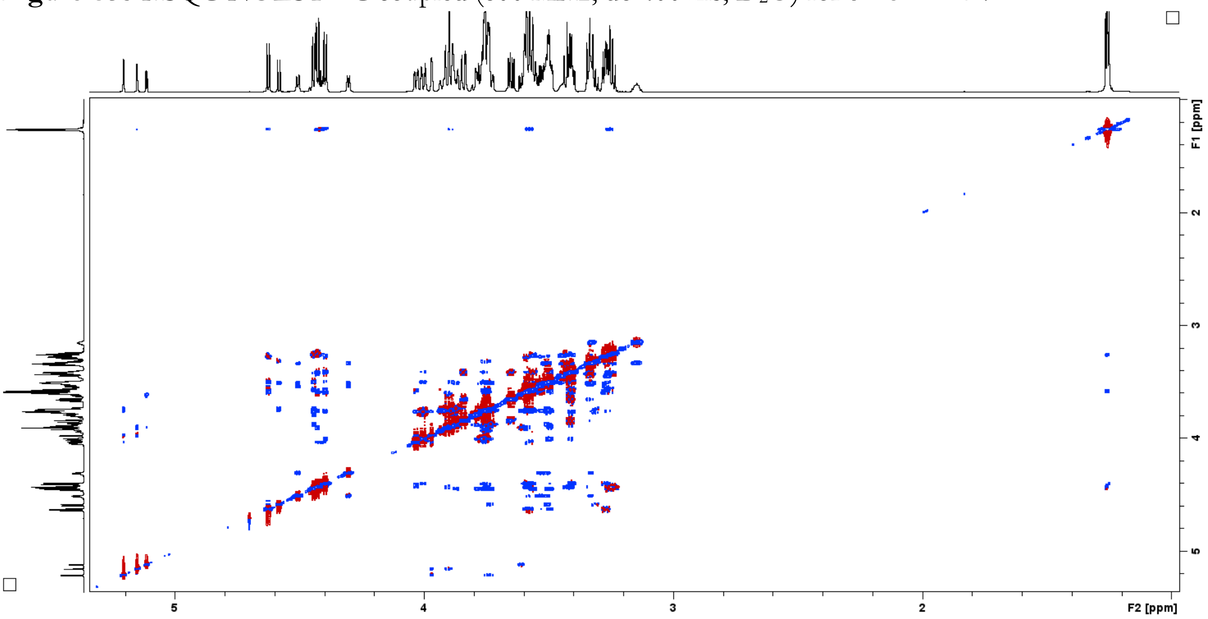

Figure S59 NOESY (800 MHz, d8 400 ms, D_2O) for **7mer-III-F**.

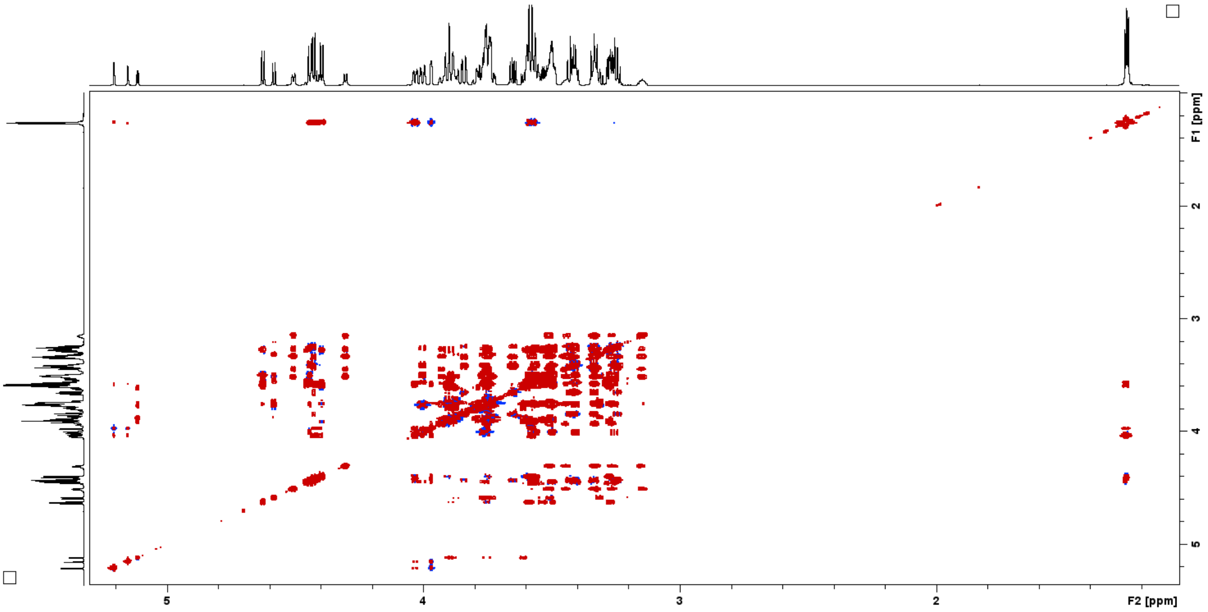

Figure S60 TOCSY (800 MHz, d9 80 ms, D_2O) for **7mer-III-F**.

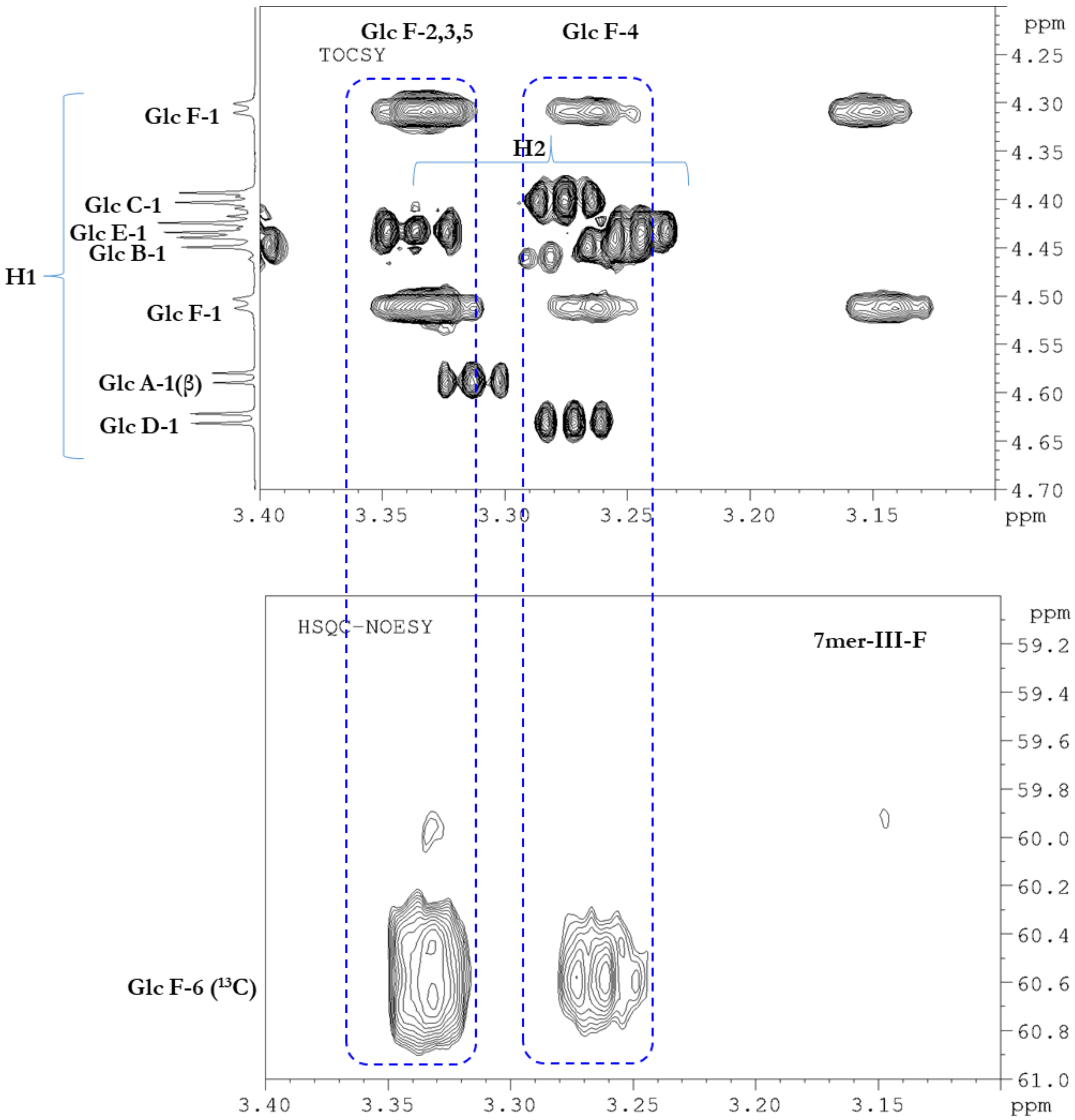

Figure S61 Expansion of the key spectral regions (same spectral width in the f2 ^{1}H-dimension) in the 2D TOCSY and 2D HSQC-NOESY experiments recorded for **7mer-III-F**: for Glc F-6 protons, only the expected intra-residue NOEs are identified: Glc F-4, Glc F-5.

4.4.10 NMR Characterization of 13mer-III-F

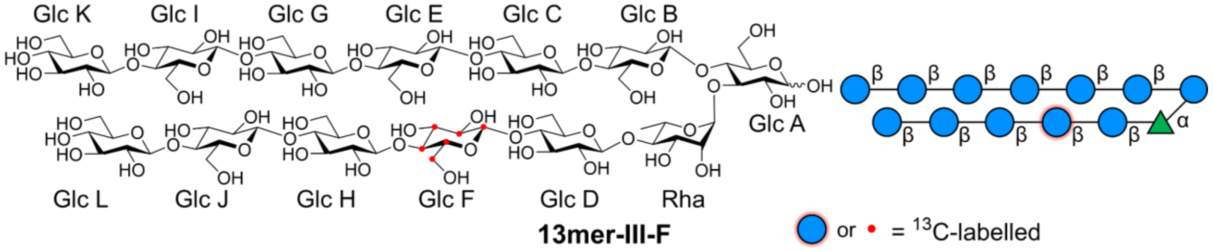

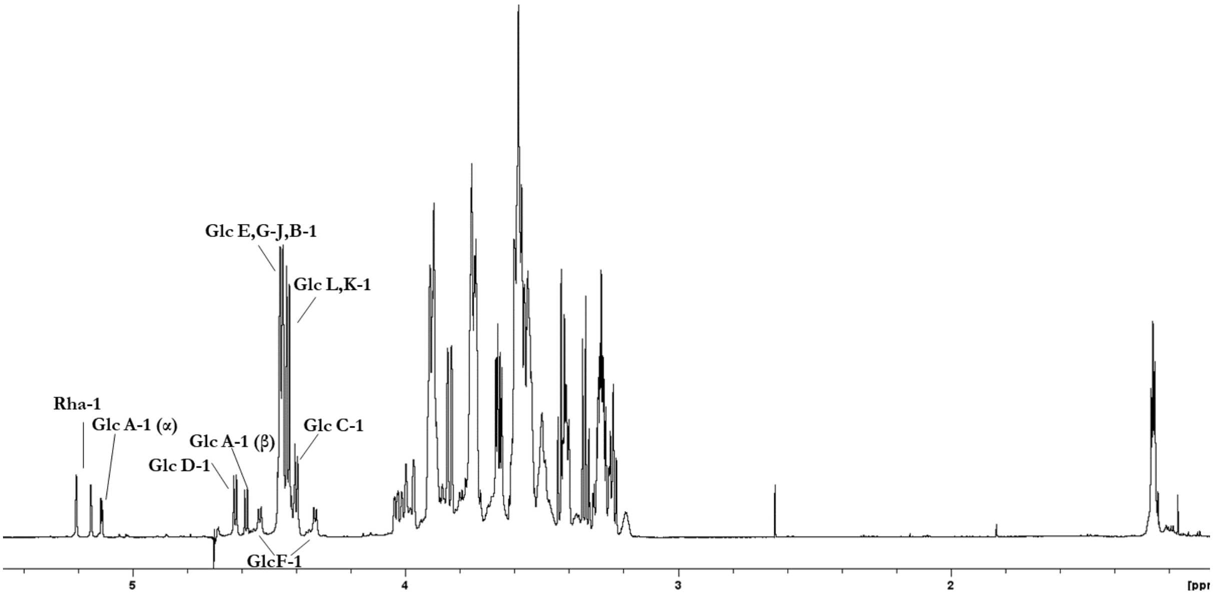

Figure S62 ^{1}H-NMR, (800 MHz, 298K, D_2O) of **13mer-III-F** with assignments of anomeric signals.

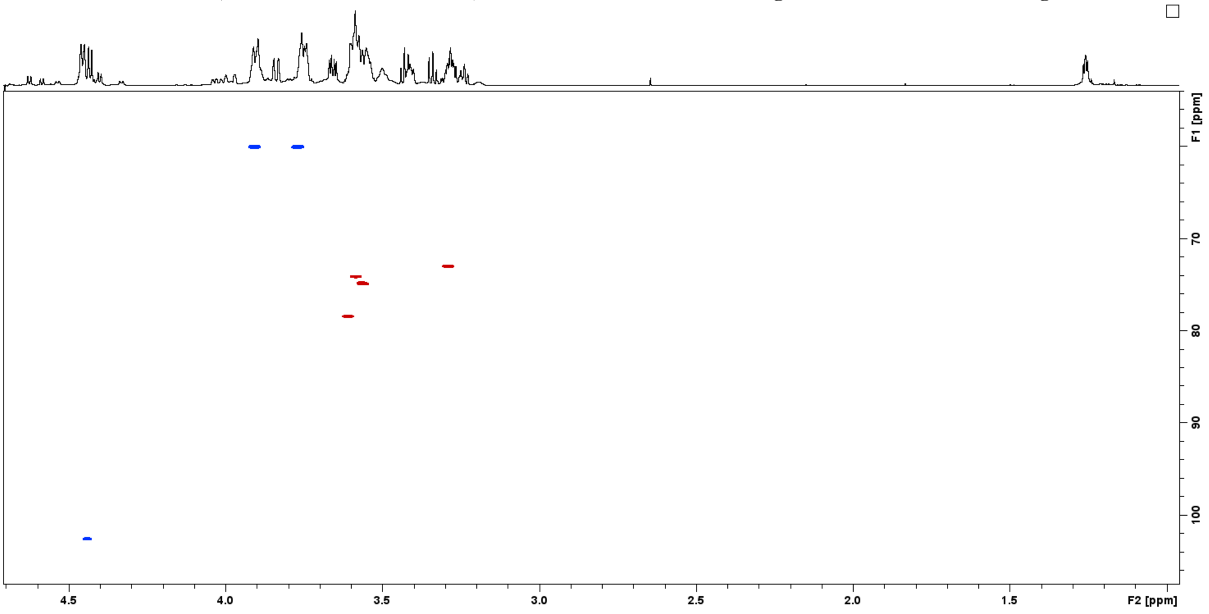

Figure S63 HSQC ^{13}C decoupled of **13mer-III-F**. Only the ^{13}C-labelled signals are visible.

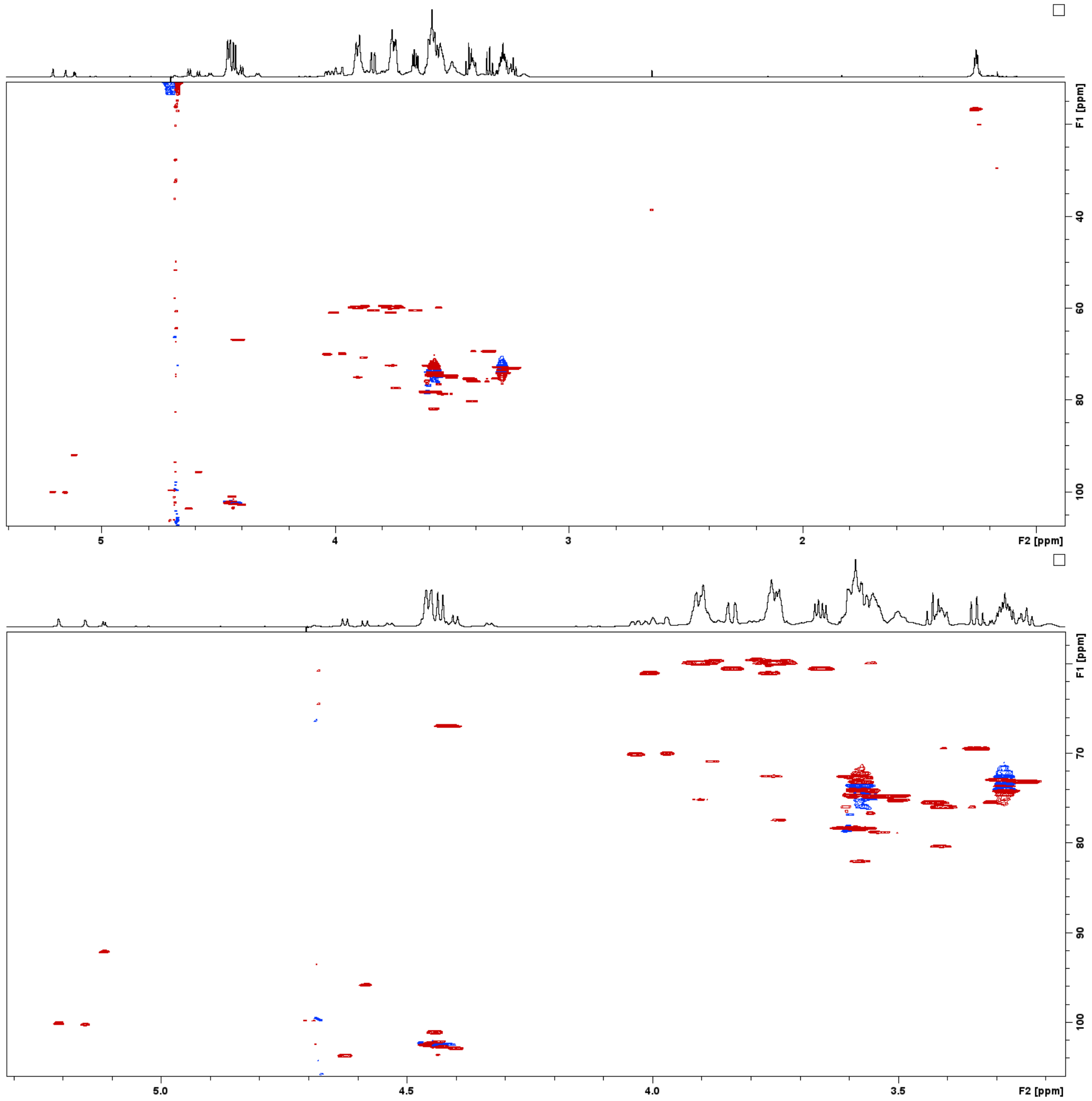

Figure S64 HSQC-ct recorded to suppress the ^{13}C-labelled signals (800 MHz, ct 11.2 ms, D_2O) for **13mer-III-F**.

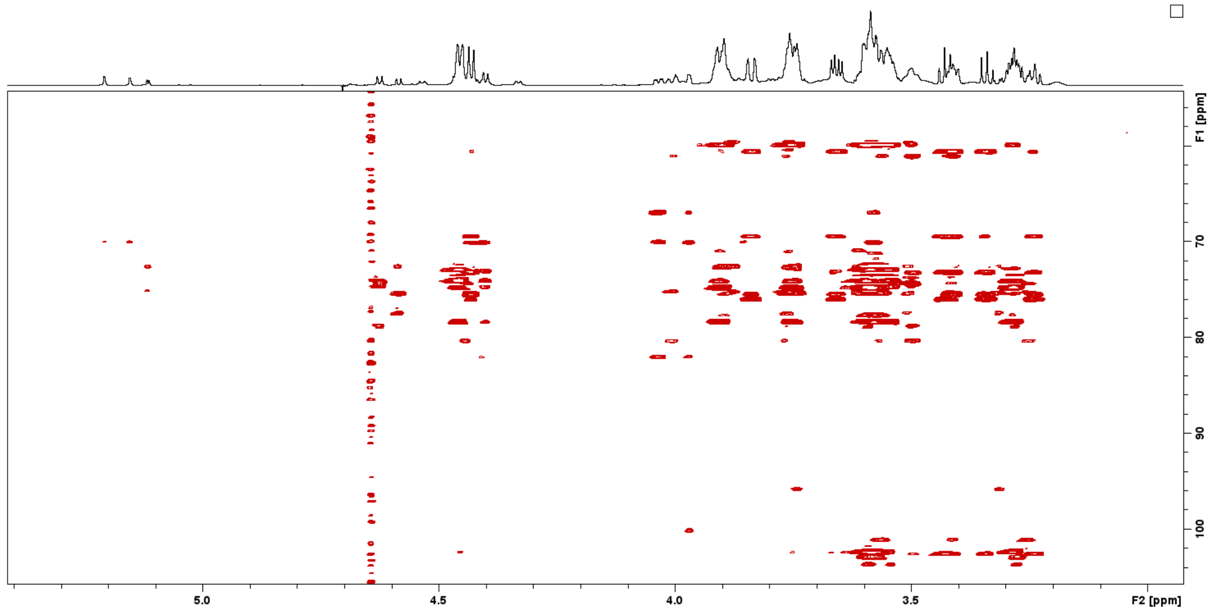

Figure S65 HSQC-TOCSY-ct recorded to suppress the ^{13}C-labelled signals (800 MHz, ct 11.2 ms, d9 80 ms, D_2O) for **13mer-III-F**.

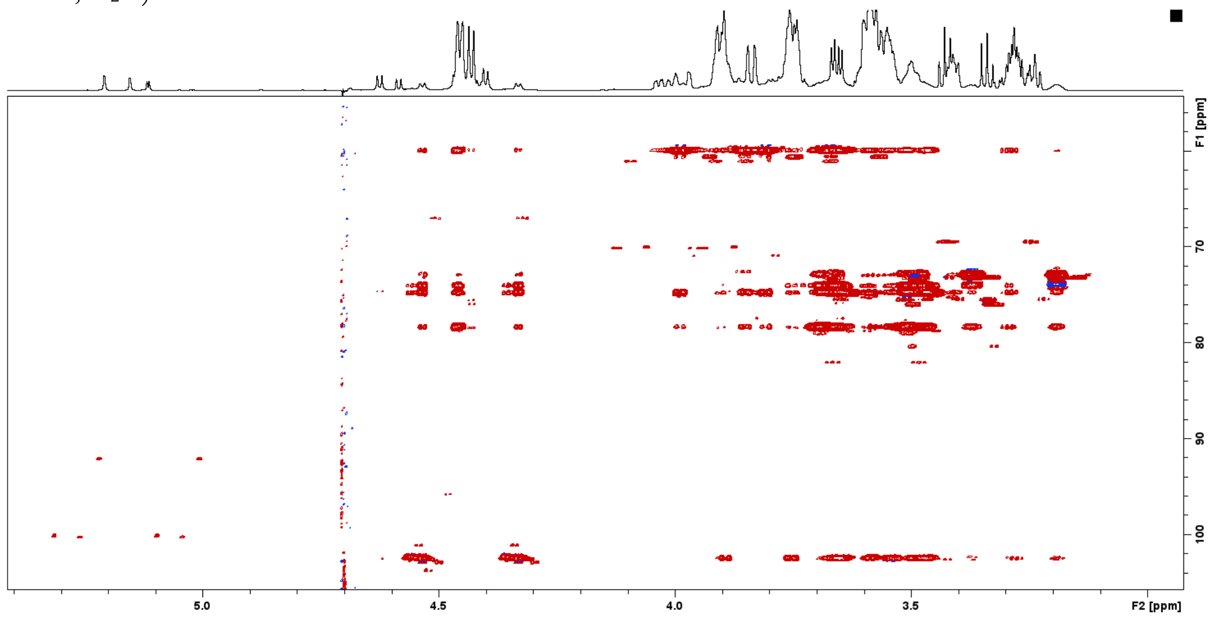

Figure S66 HSQC-NOESY ^{13}C coupled (800 MHz, d8 400 ms, D_2O) for **13mer-III-F**.

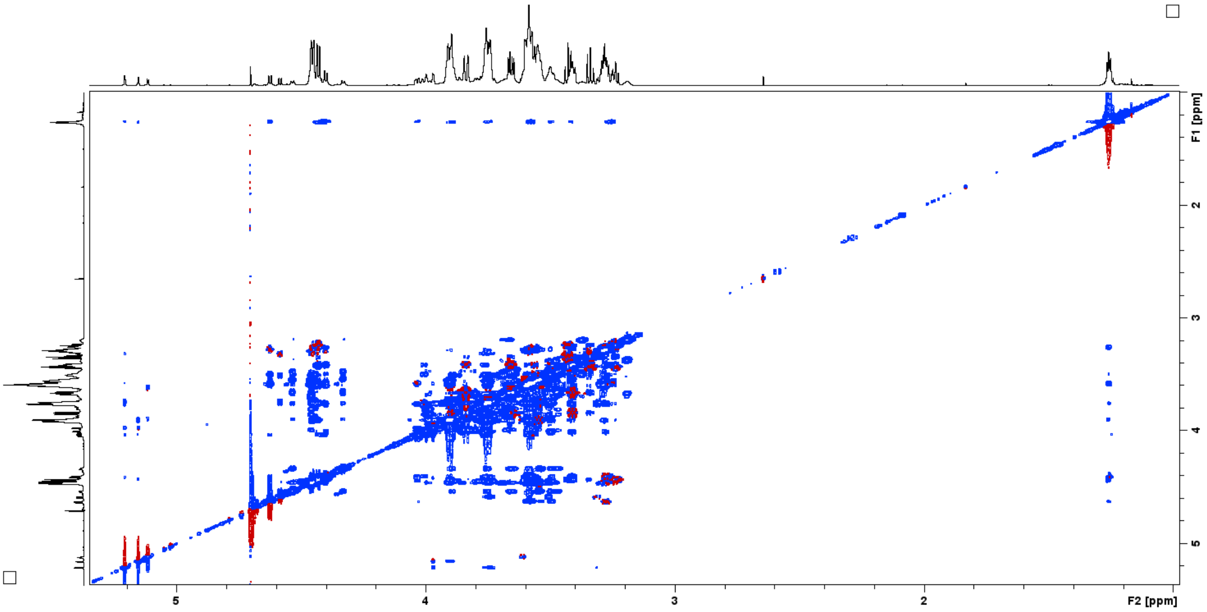

Figure S67 NOESY (800 MHz, d8 400 ms, D_2O) for **13mer-III-F**.

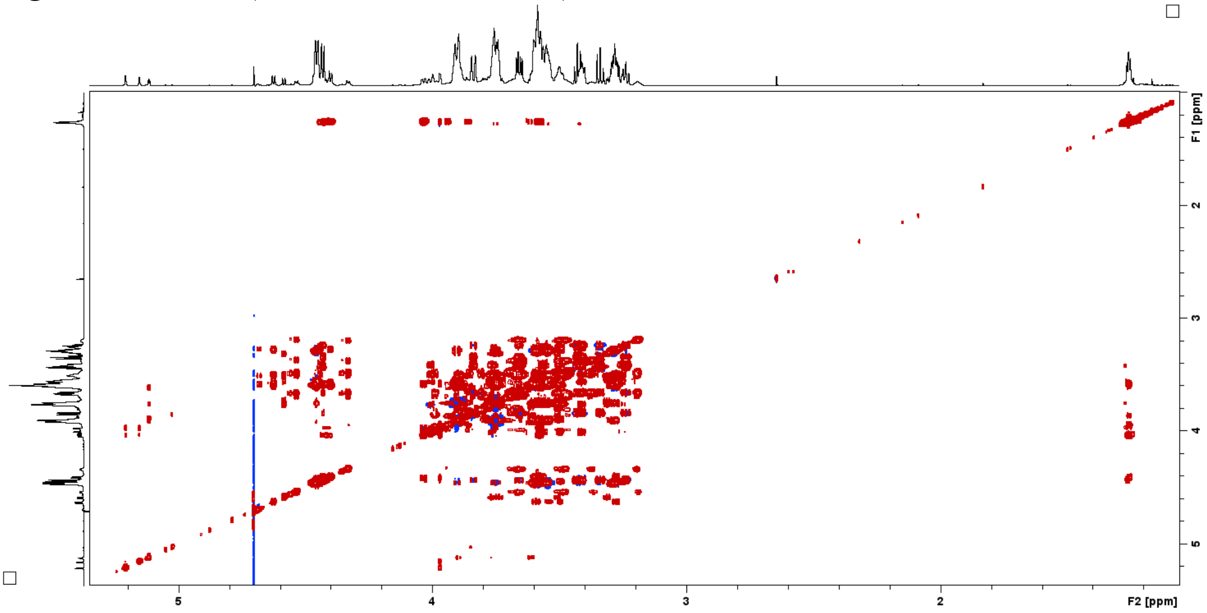

Figure S68 TOCSY (800 MHz, d9 80 ms, D_2O) for **13mer-III-F**.

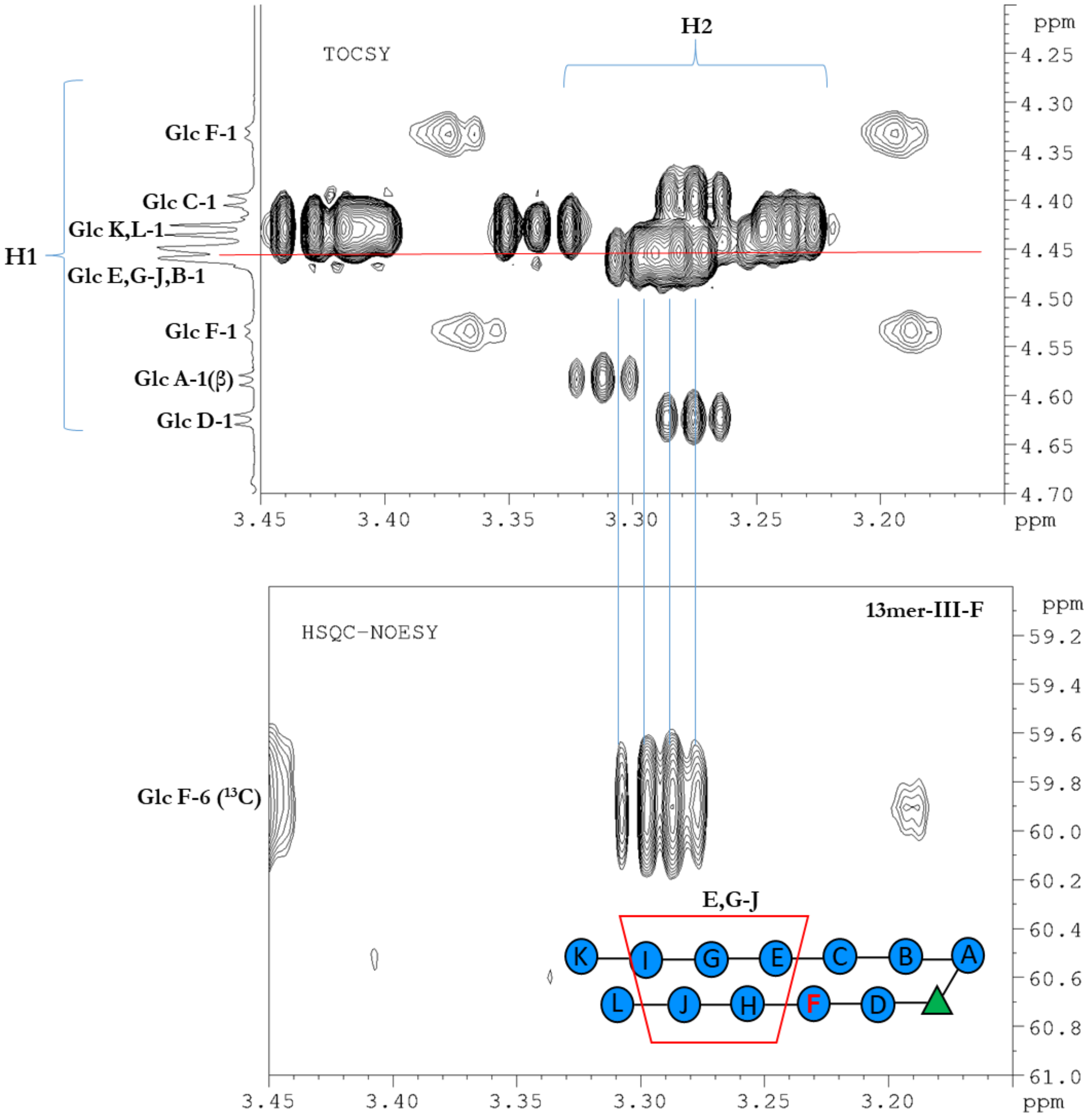

Figure S69 Expansion of the key spectral regions (same spectral width in the f2 ^{1}H-dimension) in the 2D TOCSY and 2D HSQC-NOESY experiments recorded for **13mer-III-F**: for the Glc F-6 protons (attached to ^{13}C-labelled residue), besides the intra-residue cross peaks, key inter-residue NOEs are identified: the inter-strand Glc F-6/Glc E-2 and the intra-strand Glc F-6/Glc H-2.

4.4.11 Comparative analysis of the 6mer-III, 7mer-III-F, 9mer-III and 13mer-III-F analogues

- Determination of the distance Rha-Glc B by STEP-NOESY experiments[11] for **6mer-III, 7mer-III-F, 9mer-III,** and **13mer-III-F.**
- To determine the distance between residues Rha and Glc B and the effect of the non-conventional hydrogen bonding, NOEs from Rha-5 were measured. As this proton shows overlap within the anomeric region, the STEP-NOESY strategy was employed (Figure S73). Isotropic mixing from Rha-6 was optimized to obtain the highest signal at Rha-5 at the possible lower mixing time, which was 30ms. Then, NOE (400ms) from the clean Rha-5 signal was obtained. The results are shown in Figure S73 (B). The absolute NOE intensities increase with the size, (the rotational motion correlation time), but the associated distances estimated for the **7-13mer-III** molecules remain similar, while it is clearly larger for the one-stranded **6mer-III** analogue. The intra-residue Rha-5/Rha-3 distance, estimated for α-methyl Rha using the Macromodel software, was used as internal reference.

(A)

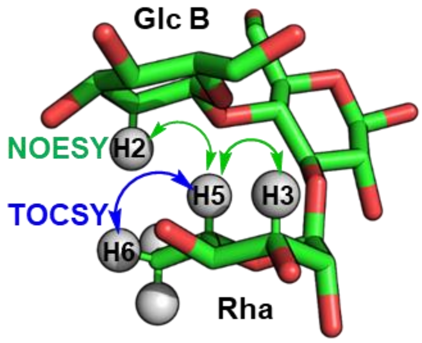

(B)

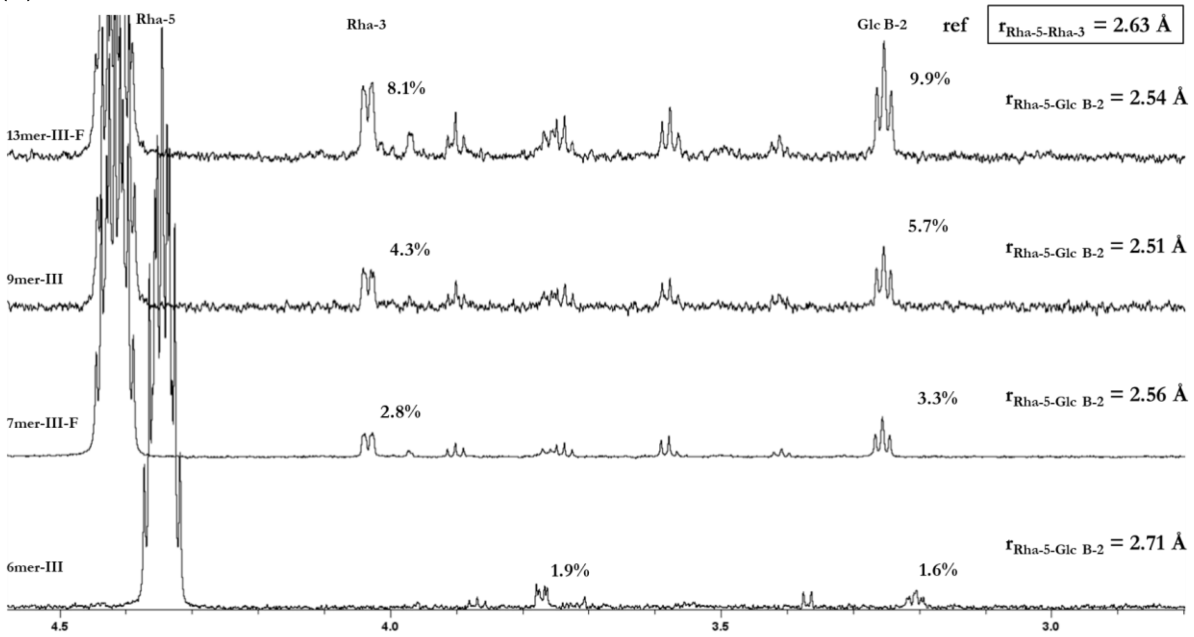

Figure S70 (A) STEP-NOESY selective excitation of Rha-6 followed by isotropic mixing (30ms) transfers the magnetization to Rha-5, which is then selectively inverted, for subsequent cross-relaxation (400ms) to Glc B-2 (inter-residue) and Rha-3 (intra-residue, used as distance reference). **(B)** Experimental data and calculated distances using Isolated Spin Pair Approximation (ISPA).

4.4.12 Diffusion experiments of the 6mer-III, 7mer-III-F, 9mer-III, and 13mer-III-F analogues

Diffusion NMR experiments were measured at 800MHz and 293K in D_2O solutions.

The Bruker standard sequence was used, which employs the stimulated echo, LED and bipolar gradient pulses for diffusion (ledbpgp2s), with d20=150ms, p30=1.2ms.

The apparent molecular weights (Mw) were estimated from the diffusion coefficients by using the MNOVA program, (Table S5 and Figure S71). The deviation between the estimated from the actual Mw is significant higher for the **13mer-III-F** analogue. This fact suggests that certain degree of self-association is taking place.

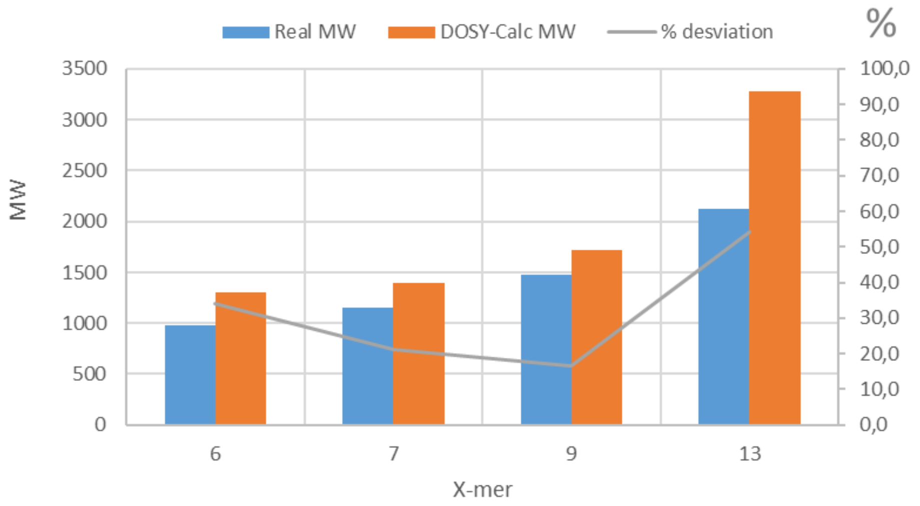

Figure S71 Representation of the real and calculated (DOSY) molecular weights. The % of deviation is indicated for each oligomer.

4.4.13 Chemical shift perturbation (CSP)analysis for Rha-5 and Glc C-1

CSP was analysed for protons Rha-5 and Glc C-1 in **5 to 13mer-III oligomers**.

Rha-5 is involved in the non-conventional Hydrogen bond that stabilizes the turn. It is significantly deshielded when the H-bond is formed. In contrast, the Glc C-1 chemical shift is related to the interaction of Glc C-1 with the apolar β-face of Glc D. Indeed, it is clearly shielded when both residues are close in space. The corresponding values are represented for every oligomer in Figure S75. For the one-stranded **6mer-III**, there is not any D residue. Thus, δGlc C-1 shows the most downfield value and δRha-5, the most upfield value. In syntony with this, the Rha-5 and Glc B-2 estimated distance is the highest one, indicating that turn is less stabilized. In contrast, for the closed **5mer-III** analogue, the behaviour is the opposite for all the parameters, as expected for a closed hairpin, indeed forced to be closed. For the **5, 7, 9, 13mer-III oligomers**, the chemical shift and distance values are fairly similar among them and support the formation of a stable hairpin at those regions.

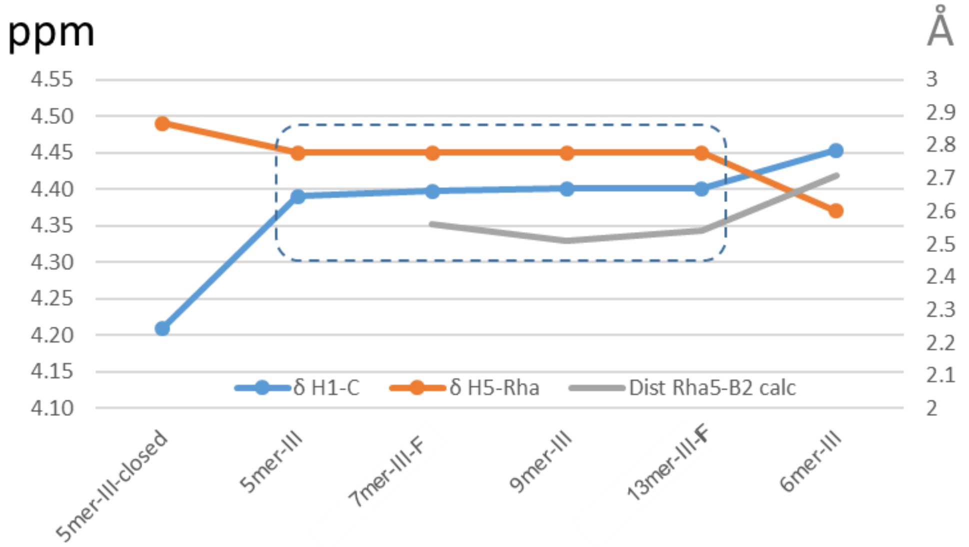

Figure S72 The behaviour of the chemical shift value observed for Glc C-1 and Rha-5 and the NOE-based Rha-5/Glc B-2 distance for the indicated oligomers.

4.4.14 Summary of NMR analysis

		I-Turn	I-Turn	I-Turn	II	II	II	III			
	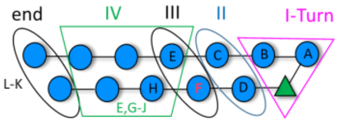	NOE Rha-6/Glc B-2	Dist (Å) Rha-5/Glc B-2[1]	δ (ppm) Rha-5[2]	NOE Glc C-1/Glc D-2	Dist (Å) Glc C-1/Glc D-2[3]	δ (ppm) Glc C-1[4]	NOE Glc F/ Glc E	D x 10^{-10} (m^2/s)	Mw	Mw est (DOSY)[5]
**5mer-III**	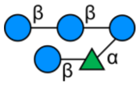	x	*nd*[6]	4.45		2.93-3.02	4.39	-	*nd*	828.6	
**5mer-III-Closed**	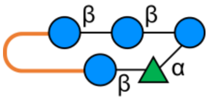	x	*nd*	4.49		2.63-2.74	4.21	-	*nd*	924.9	
**6mer-III (half hairpin)**	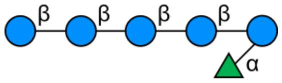	*nd*	2.71	4.37	-	-	4.45	-	2.1	974.9	1305
**7mer-III-F**	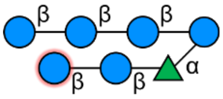	x	2.56	4.45	x	2.90-3.00	4.40	<end> No NOE	2.04	1153.1	1397
**9mer-III**	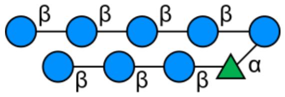	x	2.51	4.45		*nd*	4.40	*nd (found in 9mer-I)*	1.9	1477.3	1724
**13mer-III-F**	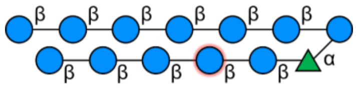	x	2.54	4.45	x	2.88-2.98	4.40	Glc F-6/(Glc E-2+Glc H-2)	1.5	2125.9	3274

Table S5 Summary of the observed NOE values, the corresponding estimated inter-proton distances and the DOSY-based diffusion coefficients obtained for the **5-13mer-III** hairpins.

[1] Calculated from STEP-NOESY experiments (TOCSY: Rha-6/Rha-5, NOESY: Rha-5/Glc B-2)
[2] Related with the non-conventional H-bond
[3] Estimated from NOESY experiments
[4] Related with the interaction of Glc C-1 and the apolar Glc D β-face
[5] Estimated from DOSY experiments in MNOVA program
[6] Not determined

5 References:

(1) Eller, S.; Collot, M.; Yin, J.; Hahm, H. S.; Seeberger, P. H. Automated solid-phase synthesis of chondroitin sulfate glycosaminoglycans. *Angew. Chem. Int. Ed.* **2013**, *52* (22), 5858-5861. https://doi.org/10.1002/anie.201210132.

(2) Fittolani, G.; Tyrikos-Ergas, T.; Poveda, A.; Yu, Y.; Yadav, N.; Seeberger, P. H.; Jiménez-Barbero, J.; Delbianco, M. Synthesis of a glycan hairpin. *Nature Chemistry* **2023**. 10.1038/s41557-023-01255-5. Fittolani, G.; Tyrikos-Ergas, T.; Poveda, A.; Yu, Y.; Yadav, N.; Seeberger, P. H.; Jiménez-Barbero, J.; Delbianco, M. Synthesis of a glycan hairpin. *Nat. Chem.* **2023**, *15* (10), 1461-1469. https://doi.org/10.1038/s41557-023-01255-5.

(3) Dallabernardina, P.; Schuhmacher, F.; Seeberger, P. H.; Pfrengle, F. Mixed-Linkage Glucan Oligosaccharides Produced by Automated Glycan Assembly Serve as Tools To Determine the Substrate Specificity of Lichenase. *Chem. Eur. J.* **2017**, *23* (13), 3191-3196. https://doi.org/10.1002/chem.201605479.

(4) Ricardo, M. G.; Reuber, E. E.; Yao, L.; Danglad-Flores, J.; Delbianco, M.; Seeberger, P. H. Design, Synthesis, and Characterization of Stapled Oligosaccharides. *J. Am. Chem. Soc.* **2022**, *144* (40), 18429-18434. https://doi.org/10.1021/jacs.2c06882.

(5) Le Mai Hoang, K.; Pardo-Vargas, A.; Zhu, Y.; Yu, Y.; Loria, M.; Delbianco, M.; Seeberger, P. H. Traceless Photolabile Linker Expedites the Chemical Synthesis of Complex Oligosaccharides by Automated Glycan Assembly. *J. Am. Chem. Soc.* **2019**, *141* (22), 9079-9086. https://doi.org/10.1021/jacs.9b03769.

(6) Gude, M.; Ryf, J.; White, P. D. An accurate method for the quantitation of Fmoc-derivatized solid phase supports. *Lett. Pept. Sci.* **2002**, *9* (4), 203-206. https://doi.org/10.1023/A:1024148619149.

(7) Hurevich, M.; Kandasamy, J.; Ponnappa, B. M.; Collot, M.; Kopetzki, D.; McQuade, D. T.; Seeberger, P. H. Continuous photochemical cleavage of linkers for solid-phase synthesis. *Org. Lett.* **2014**, *16* (6), 1794-1797. https://doi.org/10.1021/ol500530q.

(8) Yu, Y.; Tyrikos-Ergas, T.; Zhu, Y.; Fittolani, G.; Bordoni, V.; Singhal, A.; Fair, R. J.; Grafmüller, A.; Seeberger, P. H.; Delbianco, M. Systematic Hydrogen-Bond Manipulations To Establish Polysaccharide Structure–Property Correlations. *Angew. Chem. Int. Ed.* **2019**, *58* (37), 13127-13132. https://doi.org/10.1002/anie.201906577.

(9) Topin, J.; Lelimousin, M.; Arnaud, J.; Audfray, A.; Pérez, S.; Varrot, A.; Imberty, A. The Hidden Conformation of Lewis x, a Human Histo-Blood Group Antigen, Is a Determinant for Recognition by Pathogen Lectins. *ACS Chem. Biol.* **2016**, *11* (7), 2011-2020. https://doi.org/10.1021/acschembio.6b00333.

(10) Zhang, Y.; Gómez-Redondo, M.; Jiménez-Osés, G.; Arda, A.; Overkleeft, H. S.; van der Marel, G. A.; Jiménez-Barbero, J.; Codée, J. D. C. Synthesis and Structural Analysis of Aspergillus fumigatus Galactosaminogalactans Featuring α-Galactose, α-Galactosamine and α-N-Acetyl Galactosamine Linkages. *Angew. Chem. Int. Ed.* **2020**, *59* (31), 12746-12750. https://doi.org/10.1002/anie.202003951. Aeschbacher, T.; Zierke, M.; Smieško, M.; Collot, M.; Mallet, J. M.; Ernst, B.; Allain, F. H.; Schubert, M. A Secondary Structural Element in a Wide Range of Fucosylated Glycoepitopes. *Chem. Eur. J.* **2017**, *23* (48), 11598-11610. https://doi.org/10.1002/chem.201701866.

(11) Hu, H.; Bradley, S. A.; Krishnamurthy, K. Extending the limits of the selective 1D NOESY experiment with an improved selective TOCSY edited preparation function. *J. Magn. Reson.* **2004**, *171* (2), 201-206. https://doi.org/10.1016/j.jmr.2004.08.018.
